# Borylation–Reduction–Borylation for the Formation of 1,4-Azaborines

**DOI:** 10.1021/acs.orglett.3c03731

**Published:** 2023-12-06

**Authors:** Shantaram S. Kothavale, Saqib A. Iqbal, Emily L. Hanover, Abhishek K. Gupta, Eli Zysman-Colman, Michael J. Ingleson

**Affiliations:** † EaStCHEM School of Chemistry, The University of Edinburgh, Edinburgh EH9 3FJ, United Kingdom; ‡ Organic Semiconductor Centre and EaStCHEM School of Chemistry, University of St Andrews, St Andrews KY16 9ST, United Kingdom

## Abstract

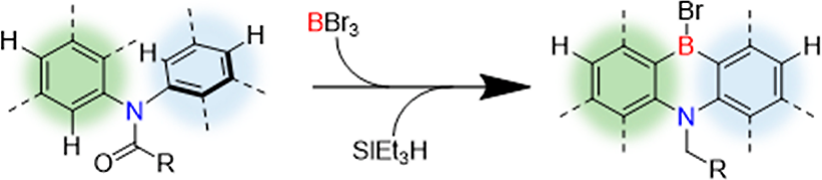

Given the current interest in materials containing 1,4-azaborine units, the development of new routes to these structures is important. Carbonyl directed electrophilic borylation using BBr_3_ is a facile method for the *ortho*-borylation of *N*,*N*-diaryl-amide derivatives. Subsequent addition of Et_3_SiH results in carbonyl reduction and then formation of 1,4-azaborines that can be protected *in situ* using a Grignard reagent. Overall, borylation–reduction–borylation is a one-pot methodology to access 1,4-azaborines from simple precursors.

Aryl-fused 1,4-azaborines are polycyclic aromatic hydrocarbons (PAHs) that contain *ortho* boron and nitrogen centers (e.g., Figure 1).^1−4^ Materials containing these units are of considerable current interest principally due to their attractive photophysical properties, which has led to their use as emitters in OLEDs.^5−7^ However, they are utilized in other areas, e.g. as components of novel ligands in catalysis^8^ and as bioisosteres.^9^ Therefore, the efficient synthesis of 1,4-azaborines is of significant importance.^10−12^ The classic route to these compounds builds on the pioneering work of the groups of Maitlis, Clark and Kawashima.^13−15^ This uses an *ortho*-halogenated diarylamine in a lithium/halogen exchange, with a boron electrophile then added to form the 1,4-azaborine (Figure 1A). While widely used,^16^ the requirement for halogenated precursors adds complexity to this approach. This is particularly true if the halogenated-diarylamine is formed via a Hartwig-Buchwald (HB) coupling reaction, as this necessitates making multihalogenated precursors that undergo a selective HB-coupling.^17,18^ A more efficient route involves the double C–H borylation (one inter- and one intramolecular) of a diarylamine using a boron electrophile. However, the primary product from intermolecular electrophilic borylation of (di)arylamines is the *para* (to *N*) borylated isomer.^19^ Nevertheless, seminal work by Hatakeyama and co-workers demonstrated that 1,4-azaborines can be accessed by sequential electrophilic C–H borylations using BX_3_ (X = Br or I). First, they achieved this by blocking the *para* position, forcing the electrophilic borylation to the *ortho* site (Figure 1B).^20^ Subsequently, they demonstrated that in certain cases under forcing conditions it is possible to form 1,4-azaborines using arylamine precursors that do not contain blocking groups at the *para* position (Figure 1C).^21^ These two approaches, termed “one-shot borylations”, are powerful and efficient routes to form these important materials. The absence of *para*-borylation in the last approach is notable and is presumably due to a combination of (a) the extended PAH structures having a HOMO localized on the *ortho* sites;^22,23^ and (b) reversible *para* C–H borylation under the high temperatures used (generally 170–220 °C). While these developments are impressive, alternative routes to transform diarylamine derivatives into 1,4-azaborines are of interest particularly if they: (i) expand the accessible compound space; (ii) proceed under milder conditions.

**Figure 1:**
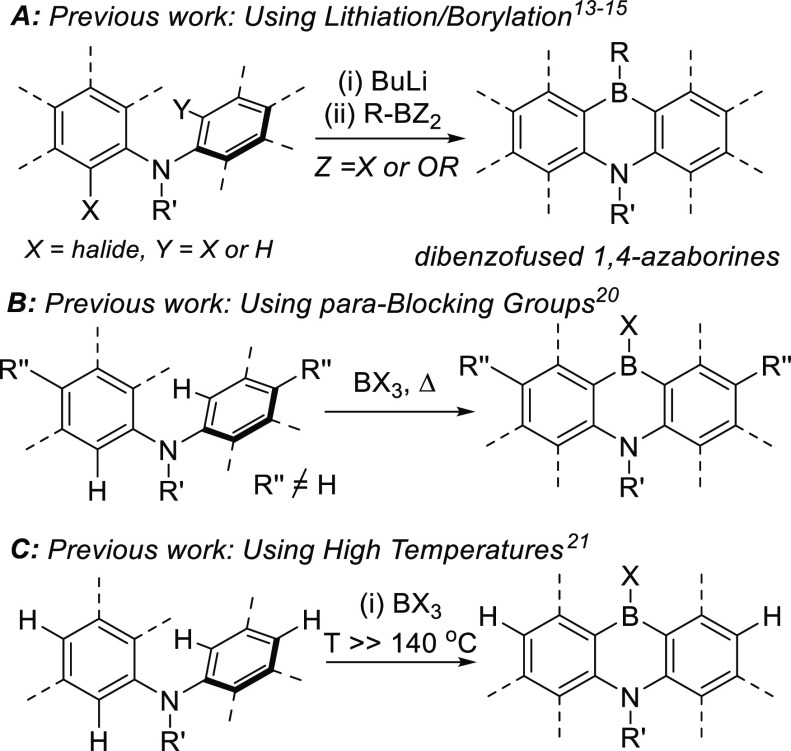
Previous work forming 1,4-azaborines by lithiation/borylation (A) or one-shot borylations (B,C).

One key challenge to form 1,4-azaborines under mild conditions is achieving intermolecular electrophilic borylation with the desired (*ortho*) regiochemistry. One way to affect facile *ortho* electrophilic borylation of aniline derivatives is to install a directing group (DG) at nitrogen and then add BBr_3_.^24−27^ After enabling the *ortho*-borylation, the DG needs to be removed to access an *ortho*-BBr_2_-diarylamine that can then be used for the intramolecular electrophilic borylation to form the 1,4-azaborine. However, the removal of the DGs used to date in directed electrophilic borylation of diaryl amines requires conditions that are not compatible with Aryl-BBr_2_ units.^28^ An alternative approach exploits recent reports of carbonyl directed *ortho*-borylation using BBr_3_.^29^ Post borylation the carbonyl moiety can be reduced using silanes (Figure 2, top).^29b^ This leads to an ArylBBr_2_ unit, as confirmed by isolation of the Lewis adduct with the newly formed amine, which produced a boracycle (e.g., compound **A**). We hypothesized that applying this approach to diarylamine derivatives would lead to a product that does not contain a N → B dative bond (e.g., Figure 2. bottom right). Lewis adduct formation in this case will be disfavored due to the lower Lewis basicity of the diarylamine (relative to the amine in **A**)^30^ coupled with the strained nature of the four-membered boracycle that would be produced on B–N formation. Thus, the ArylBBr_2_ unit will be available to perform the second C–H borylation and form the 1,4-azaborine. Herein we report a borylation–reduction–borylation strategy that forms 1,4-azaborines from simple diarylamine precursors at temperatures <60 °C.

**Figure 2:**
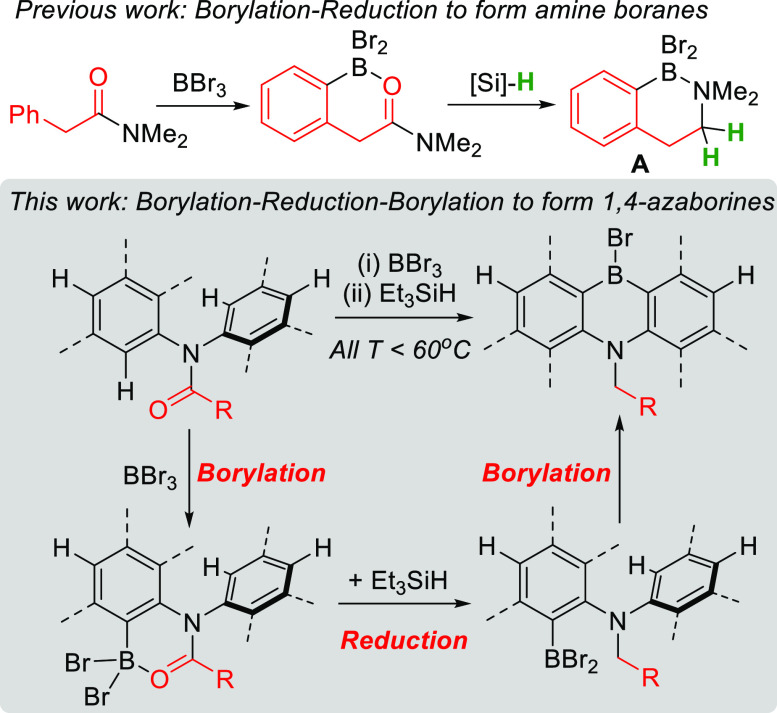
Top, previous borylation-reduction. Bottom, this work.

Based on our previous work,^31^ initial studies used *N,N*,2-triphenylacetamide, **1a**, which contains two inequivalent sites for directed *ortho* C–H borylation, on the PhCH_2_ and on the N–Ph unit. Monitoring the reaction of **1a** with BBr_3_ by *in situ* NMR spectroscopy revealed selective borylation to form **2a-Br**_**2**_ which was in equilibrium with **[2a-Br][BBr**_**4**_**]**, (based on comparable NMR spectra to that reported for related systems).^31^ The mixture of **2a-Br**_**2**_ and **[2a-Br][BBr**_**4**_**]** reacted with ≥2 equiv of Et_3_SiH to ultimately give one major new boron containing product with the ^11^B (δ_11B_ = 50.1) and ^1^H NMR spectra consistent with the formation of **3a-Br**. Addition of water to this compound led to a new ^11^B resonance (δ_11B_ = 38),^32^ consistent with the 1,4-azaborinic acid, **3a–OH** (Figure 3, bottom right). Definitive confirmation of 1,4-azaborine formation was forthcoming from the conversion of **3a-Br** into **4a** by the addition of MesMgBr. Compound **4a** is bench stable and was isolated by column chromatography, enabling its full characterization.

**Figure 3:**
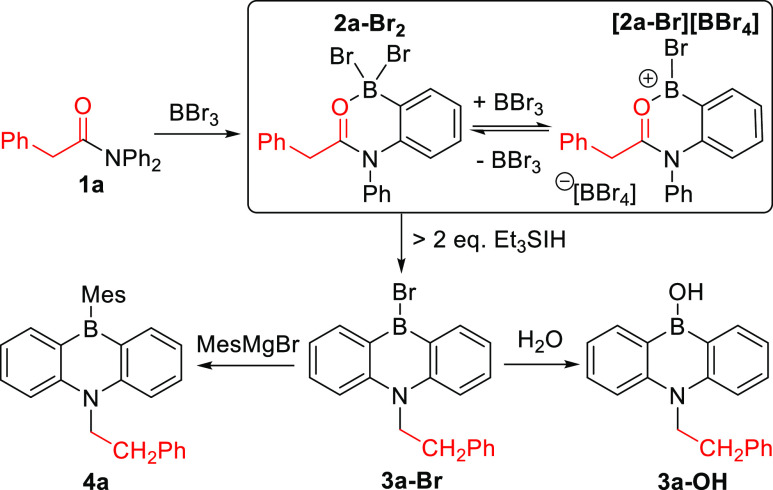
Initial studies into the synthesis of 1,4-azaborines by borylation–reduction–borylation.

With the confirmation of 1,4-azaborine formation by this approach in hand, an optimization study was performed to identify borylation–reduction–borylation conditions applicable to multiple substrates. This revealed that 2.5 equiv of Et_3_SiH was sufficient for full carbonyl reduction, with this step giving optimal outcomes when performed in DCM with heating. Higher yields also were obtained using ≥4 equiv of MesMgBr (as some MesMgBr is consumed by reaction with the Et_3_SiBr byproduct from the reduction). Using these conditions, a number of nitrogen-substituted DGs were explored, including pivaloyl (**1b**), hexanoyl (**1c**) and benzoyl (**1d**), forming **4b**–**4d** (Figure 4) containing *N*-neopentyl, *N*-hexyl, and *N*-benzyl units. respectively (note: homobenzyl in **4a**, and neopentyl in **4b**, have not been used as a *N* substituent in any previously reported 1,4-azaborines to our knowledge). Analogous to the reaction starting from **1a**, monitoring the borylation–reduction–borylation of **1c** by *in situ* NMR spectroscopy revealed that the *B*-Br-1,4-azaborine (**3c-Br**) is the only major boron-containing product formed (by multinuclear NMR spectroscopy; see Figures S1, S2). However, for **1d**, although the initial C–H borylation occurs cleanly, the subsequent reduction-borylation steps are not clean. Instead, products from N–C cleavage are observed (see Figures S3, S4. This is consistent with the lower isolated yield observed for **4d** relative to **4a**–**4c**. Nevertheless, accessing **4d** with a *N*-benzyl group is important as it can be deprotected to form the *N*-H-1,4-azaborine for use in subsequent reactions as a number of us previously have reported.^33^

**Figure 4:**
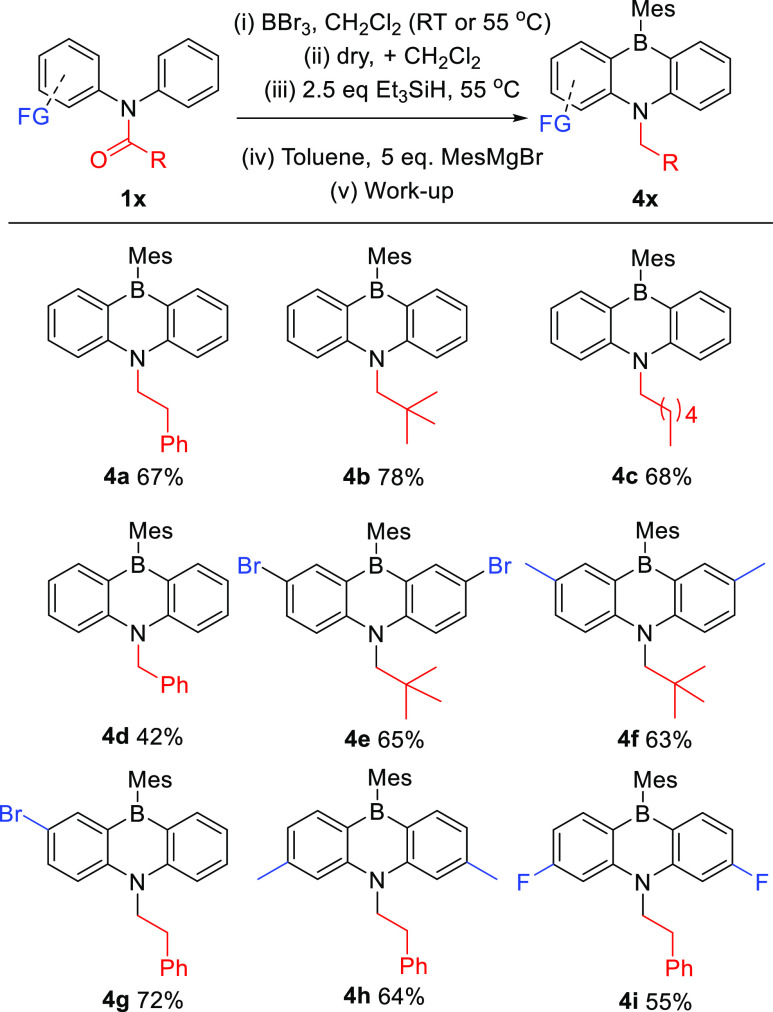
Substrate scope for dibenzofused-1,4-azaborines. Reactions were performed in sealed tubes. **4*x***: Isolated yields.

Looking at electronic effects in this reaction, electron-withdrawing bromines *meta* to the borylation position (Br σ_meta_ = 0.37) were tolerated with **4e** isolated in a yield similar to that of **4f**, which contains electron-donating methyl groups (Me σ_meta_ = −0.06). An unsymmetric monobrominated derivative also was amenable to this process with **4g** isolated in good yield. Next, we looked at the selectivity in the two C–H borylation steps by using diarylamine-substituted *meta* to *N* (**1h** and **1i**). This substitution pattern results in the two *ortho* positions being inequivalent. In both cases, the two C–H borylation steps proceeded with high selectivity for the less sterically hindered position leading to formation of **4h** and **4i.** These contain an electron-donating group (**4h**, Me σ_para_ = −0.14) and an electron-withdrawing group (**4i**, F σ_para_ = 0.15), respectively. While the functional group tolerance of this process is limited, given the use of strong electrophiles (BBr_3_) and reducing conditions, we note that halides are the functional group most widely used in organic materials for further transformations.

The potential to access 1,4-azaborines other than dibenzofused systems by using the same conditions was explored next. Attempts to form a B,N-naphthalene (**4j**, Figure 5) using *N*-vinyl-acetanilide led to no 1,4-azaborine product being isolated and instead produced a complex, intractable mixture. Using naphthyl-containing precursor **1k** led to the isolation of two 1,4-azaborine products (**4k-α,β**) from unselective borylation of the *alpha* and *beta* positions of naphthalene. In contrast, the use of pivaloyl led to the formation of the α product as the major isomer, which could be isolated in 30% yield, with minimal (<5%) β-isomer (**41-β**) isolated. Replacing the naphthalene moiety with benzothiophene led to **1m** being converted into two 1,4-azaborine isomers, **4m-α** and **4m-β**, even when pivaloyl was used as the directing group. Note, these isomeric mixtures can be separated by column chromatography. In contrast to **1k** and **1m**, the *N*-phenyl carbazole derivative, **1n**, produced only a single azaborine isomer, **4n**, from borylation *para* to *N*. Presumably, the N-Ph unit provides sufficient steric shielding of its *ortho* C–H position to prevent any observable borylation at that site. Carbazole-fused 1,4-azaborines are of interest as compounds related to **4n** have been reported previously to have superior photophysical properties and electrochemical stability relative to dibenzofused-1,4-azaborines.^34^ The extended heterocyclic cores of **4m** and **4n** are novel structures to the best of our knowledge;^35^ furthermore, they are accessible in one pot from **1m/1n**, with **1m** and **1n** themselves accessible in two simple steps from commercial precursors (a HB coupling and then an acylation). In contrast, the previously reported route to carbazole-fused 1,4-azaborines required the initial synthesis of a dibrominated dibenzo-fused 1,4-azaborine (related to **4e**), which was used in a HB-coupling reaction with *ortho*-chloro-aniline, followed by a palladium catalyzed C–C bond forming reaction to make the carbazole fused 1,4-azaborine.^34^

**Figure 5:**
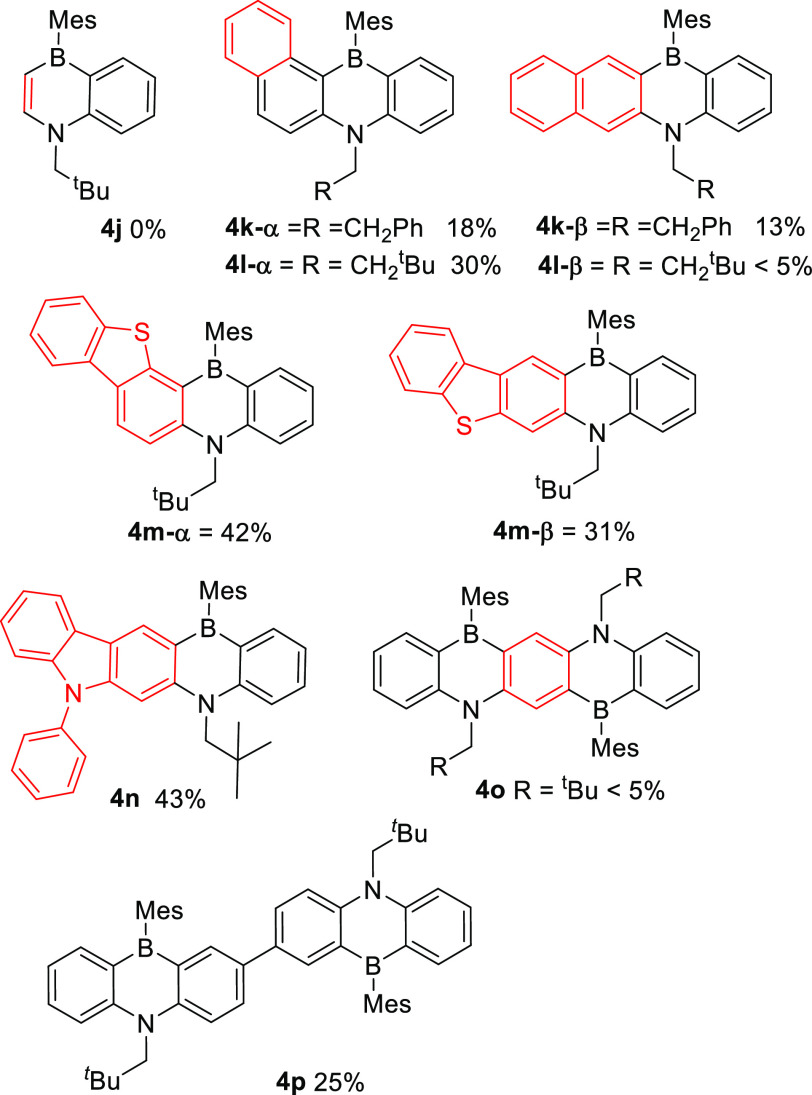
Other fused 1,4-azaborines made through borylation–reduction–borylation. Reactions in sealed tubes; yields are for isolated materials

Next, the construction of multiple 1,4-azaborine units in one PAH via this methodology was explored. However, multiple attempts to form the B_2_N_2_ pentacene **4o** via this methodology proved unsuccessful (with <5% of the desired product isolated), this included using more forcing conditions. In contrast these type of materials can be accessed using lithium/halogen exchange based synthetic routes (as per Figure 1A).^15^ The lack of significant B_2_N_2_ product being formed using this borylation–reduction–borylation method is tentatively attributed to the first C–H borylation on the central phenyl (shown in red in **4o**) electronically deactivating it (due to the π electron withdrawing effect of the boron unit)^36^ towards further C–H borylation (see section S2 for more discussion). This hypothesis also is supported by the successful formation of B_2_N_2_ compound **4p** in 25% isolated yield, with the borylation sites in **1p** more electronically isolated than those on the central phenyl in **1o**.

The functionalization of two of the 1,4-azaborines made through this borylation–reduction–borylation method also was explored. Compound **4g** was found to be compatible with standard HB coupling conditions to form **5** (Figure 6 left). Second, the oxidation of sulfur in **4m-β** was attempted as this is a well-established method to fine-tune optoelectronic properties.^37^ This led to the formation of the sulfone containing azaborine **6**. This enabled comparison of the optoelectronic properties of isomers **4m-α,β** and **6**. This revealed that the two isomers possess very similar optoelectronic properties (e.g., λ_max_ for the lowest energy absorption band = 416 and 409 nm, see Figure S98) with the peak reduction potentials being −2.13 and −2.10 V, respectively (versus Fc/Fc^+^). This was in agreement with DFT calculations (on model compounds containing N-Me groups instead of N–CH_2_^*t*^Bu, Figure S95) that confirmed closely comparable HOMO, LUMO and S_1_ energies for ^Me^**4m-α** and ^Me^**4m-β**. Finally, as expected^36^ sulfone containing **6** has a significantly stabilized LUMO energy (relative to **4m**), with the peak reduction potential observed at −1.67 V (versus Fc/Fc^+^).

**Figure 6:**
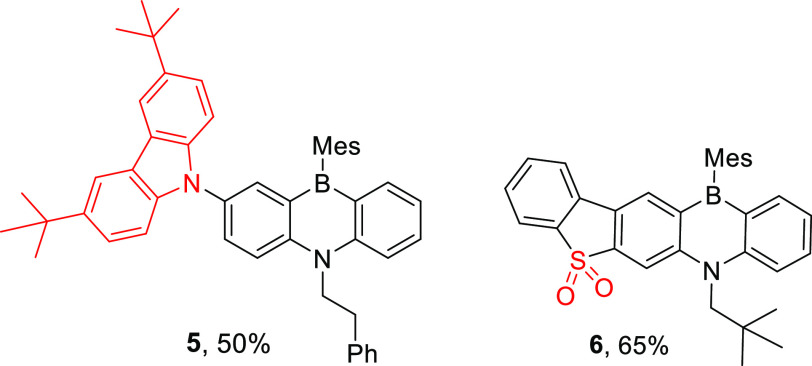
Compounds **5** and **6**.

In summary, borylation–reduction–borylation is a one-pot approach to produce a range of aryl fused 1,4-azaborines using a single set of reaction conditions. This methodology proceeds at a relatively low (≤60 °C) temperature for an inter/intramolecular electrophilic borylation based route to form 1,4-azaborines and enables formation of 1,4-azaborines that would be challenging to access by established methodologies.

## Data Availability

The data underlying this study are available in the published article and its Supporting Information. Some of the research data supporting this publication also can be accessed at https://doi.org/10.17630/d5437e72-5005-4808-9964-dfdef6adc068

## Supporting Information Available

Full experimental details, NMR spectra for all new compounds, and plots of optoelectronic data (PDF)
Cartesian coordinates for 4m-beta (XYZ)
Cartesian coordinates for 4m-alpha (XYZ)
Cartesian coordinates for compound **6** (XYZ)

## Supplementary Material

ol3c03731_si_001.pdf

ol3c03731_si_002.xyz

ol3c03731_si_003.xyz

ol3c03731_si_004.xyz
