# Supplementary material for: Borylation–Reduction–Borylation for the Formation of 1,4-Azaborines
Source: Org Lett. 2023 Dec 6;25(49):8912–6. doi: 10.1021/acs.orglett.3c03731 (PMC10729022; doi:10.1021/acs.orglett.3c03731)
Supplement: Supplementary file 1 — ol3c03731_si_001.pdf [file ol3c03731_si_001.pdf]

Supporting information

Borylation-Reduction-Borylation for the formation of 1,4-azaborines

Shantaram S. Kothavale[a], Saqib A. Iqbal[a], Emily L. Hanover[a,b], Abhishek K. Gupta,[b] Eli Zysman-Colman[b]*, Michael J. Ingleson[a]*

[a] EaStCHEM School of Chemistry, The University of Edinburgh, Edinburgh, EH9 3FJ

[b] Organic Semiconductor Centre and EaStCHEM School of Chemistry, University of St Andrews, KY16 9ST

Table of Contents

S1. General Description……………………………………………………..2

S2. Synthetic Procedure……………………………………………………..4

S3. NMR Spectra……………………………………………………………….33

S4. DFT Calculations…………………………………………………………77

S5. Electrochemical Properties…………………………………………79

S6. Photophysical Properties……………………………………………80

S7. References…………………………………………………………………..81

S1. General description

All experiments were performed under a nitrogen atmosphere in oven-dried glassware. Solvents were obtained from an Inert PureSolv MD5 SPS. All solvents were stored over 3 Å molecular sieves. All chemicals were purchased from commercial sources and used as received. All NMR tube reactions were heated in a metal heating block, all other reactions wer eheated in an oil bath. Column chromatography was performed using a CombiFlash NextGen 300+ AutoColumn or manually (40-63 µm silica). Solution ^{1}H, $^{13}C\{^{1}H\}$ and ^{11}B NMR spectra were recorded on 400 MHz and 500 MHz Bruker Spectrometers. ^{1}H and ^{13}C chemical shifts were referenced to residual solvent signals. ^{11}B and ^{19}F chemical shifts were referenced to external $BF_3{\cdot}OEt_2$ and hexafluorobenzene, respectively. The resonances of carbon atoms directly bound to boron are not always observed in $^{13}C\{^{1}H\}$ NMR spectra due to quadrupolar relaxation leading to signal broadening. Due to slow rotation on the NMR timescale, some of the amide compounds (particularly Ph-CH_2 based amides) exhibited broad NMR peaks in the aromatic region at RT, hence for better resolved NMR spectra the NMR experiments were performed at higher temperature (50 °C) in $CDCl_3$ solvent. High resolution mass spectrometry was performed at the Resource Centre for Advanced Mass Spectrometry based in the School of Chemistry at the University of Edinburgh. High resolution mass spectra were recorded on a VG autospec, or Thermo/Finnigan MAT 900, mass spectrometer. Electron Impact (EI+) spectra were performed at 70 eV using methane as the carrier gas, with either a double focusing sector field (DFSF) or time-of-flight (TOF) mass analyzer. Chemical Ionization (CI+) spectra were performed with methane reagent gas, with either a double focusing sector field (DFSF) or time-of-flight (TOF) mass analyzer. Electrospray Ionization (ESI) spectra were performed using a time-of-flight (TOF) mass analyzer. Data are reported in the form of m/z. All exact masses were calculated using the predominant isotopes, which (for the heteroatoms) are: ^{11}B and ^{79}Br.

Electrochemistry

Cyclic voltammetry (CV) and differential pulse voltammetry (DPV) measurements were performed on Electrochemical Analyzer potentiostat model 620E from CH Instrument. Experiments were conducted using a 1 mM analyte solution with 0.1 M tetra-*n*-butylammonium hexafluorophosphate $[^{n}Bu_4N]PF_6$, as the supporting electrolyte in HPLC grade MeCN with a scan rate of 100 mV s^{-1}, unless otherwise stated. The solutions were degassed by sparging with MeCN-saturated nitrogen gas for 5 minutes prior to measurements.

A glassy carbon electrode with a circular surface (diameter 3 mm) was used as the working electrode, a platinum wire as the counter electrode and an Ag/Ag^+ electrode was used as the reference electrode. The working electrode was polished with alumina slurry, then washed with deionised water, acetone and MeCN and air dried before use. The redox potentials are reported relative to a saturated calomel electrode (SCE) with a ferrocene/ferrocenium/ (F_c/F_c^+) redox couple as the internal standard (0.38 V vs SCE)[1,2]. The HOMO and LUMO energies were determined using $E_{HOMO/LUMO} = -(E^{ox}/E^{red} + 4.8)eV$,[3] where E^{ox} is anodic peak potential and E^{red} is cathodic peak potential determined from DPV relative to F_c/F_c^+.

Photophysical studies

Sample preparation

Solution samples were prepared using HPLC-grade acetonitrile (MeCN) solvent with varying concentrations on the order of 10^{-5} or 10^{-6} M for absorption and emission studies. Quartz cuvettes were used for all solution-state photophysics measurements.

UV/Vis Absorption

Absorption spectra were recorded at RT using a Shimadzu UV-2600 double beam spectrophotometer.

Steady-state emission

Steady-state and time-resolved emission spectra were recorded at 298 K using an Edinburgh Instruments FS5 fluorimeter in an open-air atmosphere. Solution samples for the steady-state measurements were excited at 330 nm for **4m-β** and 360 nm for **6** using a Xenon lamp.

DFT Calculations

The Density functional theory (DFT) calculations, including geometry optimization with a subsequent frequency calculation of the emitters, were implemented using by the Silico software 3.1.0 package manages calculations submitted to Gaussian (2016 + C.01),[4–6] [7]in the gas phase using Density Functional Theory (DFT) with the PBE0 functional[8] and the 6-31G(d,p) basis set, starting with the molecular geometry[9] obtained from ChemDraw structure. Excited singlet and triplet states were calculated by performing time-dependent DFT (TD-DFT) calculations within the Tamm-Dancoff approximation using the same functional and basis set.[10,11]

S2. Synthetic procedures

Synthesis of amides (General Procedure A)

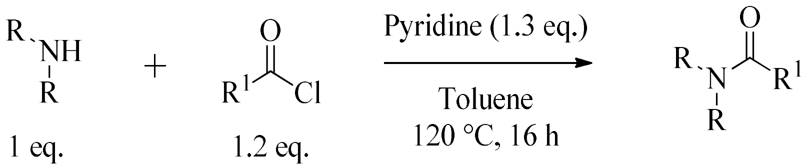

The respective amine (1 eq.) was added to an ampule fitted with a J-Youngs tap. The amine was dissolved in toluene (x mL) and pyridine (1.3 eq.) was added. To a stirring mixture was added the respective acyl chloride (1.2 eq.) dropwise, after which the ampule was sealed and stirred for 16 hours at 120 °C. The reaction mixture was cooled and DCM (50 mL) was added. The dissolved product was washed with water (50 mL) and brine (50 mL) and the organic phase was then dried over $MgSO_4$. The crude product was purified by column chromatography on silica gel (10-90 % dichloromethane in hexane) or (5-70% EtOAc in hexane) to give the pure amide.

Synthesis of amides (General Procedure B)

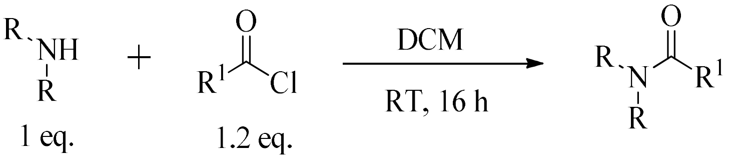

The respective amine (x eq.) was added to an ampule fitted with a J-Youngs tap. The amine was dissolved in DCM (x mL). To a stirring mixture was added the respective acyl chloride (x eq.) dropwise, after which the ampule was sealed and stirred for 16 h at room temperature. Water (40 mL) was added, and the organic phase was removed. The aqueous phase was extracted with DCM (30 mL), washed with brine (40 mL) and the combined organic fractions were washed with sat. aq. $NaHCO_3$ (30 mL) and brine (50 mL) and then dried over $MgSO_4$. The crude product was purified by column chromatography on silica gel (10-90% dichloromethane in hexane) or (5-70 % EtOAc in hexane) to give the pure amide.

Synthesis of *N,N*-2-triphenylacetanamide, 1a

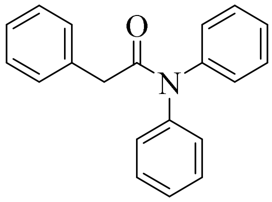

The reaction was performed according to **General procedure B** using phenylacetyl chloride (1.32 mL, 10 mmol), diphenylamine (3.56 g, 21 mmol) in DCM (20 mL). Crude product isolated after work-up was further purified by column chromatography (10-25% EtOAc in hexane) to obtain the product as a white solid. Yield: 2.25 g (78%).

Analytical data is in accordance with the literature[12]

Synthesis of *N,N*-diphenylpivalamide, 1b

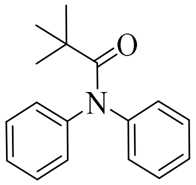

The reaction was performed according to a modified **General procedure B** using pivaloyl chloride (2.17 mL, 17.72 mmol), diphenylamine (2.5 g, 14.77 mmol), NEt_3 (2.49 mL, 17.72 mmol) in DCM (30 mL). Crude product isolated after work-up was further purified by column chromatography (10-30% EtOAc in hexane) to obtain pure product as a white solid. Yield: 2.60 g, (69%).

^{1}H NMR (500 MHz, $CDCl_3$) δ 7.36 – 7.31 (m, 4H), 7.25 – 7.20 (m, 6H), 1.15 (s, 9H). **^{13}C{^{1}H} NMR** (126 MHz, $CDCl_3$) δ 179.8, 144.7, 129.2, 128.5, 127.0, 41.9, 29.8. **[Acc. Mass]** calcd for [M+H] = $C_{17}H_{20}NO$: 254.1539, found 254.1548.

Synthesis of *N,N*-diphenylhexanamide, 1c

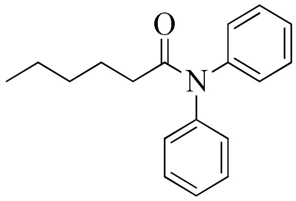

The reaction was performed according to **General procedure B** using hexanoyl chloride (1.40 mL, 10 mmol), diphenylamine (3.56 g, 21 mmol) in DCM (20 mL). Crude product

isolated after work-up was further purified by column chromatography (10-30% EtOAc in hexane) to obtain pure product as a white solid. Yield: 2.05 g, (77%).

^{1}H NMR (500 MHz, $CDCl_3$) δ 7.47 – 7.30 (m, 4H), 7.32 – 7.17 (m, 6H), 2.29 – 2.19 (m, 2H), 1.70 – 1.60 (m, 2H), 1.29 – 1.20 (m, 4H), 0.88 – 0.81 (m, 3H). **^{13}C{^{1}H} NMR** (126 MHz, $CDCl_3$) δ 173.5, 129.2, 127.8, 126.2, 143.2, 35.4, 31.6, 25.4, 22.6, 14.1. **[Acc. Mass]** calcd for [M+Na] = $C_{18}H_{21}NONa$: 290.1515, found 290.1524.

Synthesis of *N,N*-diphenylbenzamide, 1d

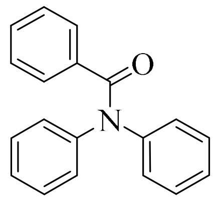

The reaction was performed according to **General procedure A** using benzoyl chloride (1.64 mL, 14.18 mmol), diphenylamine (2 g, 11.81 mmol) and pyridine (1.24 mL, 15.36 mmol) in toluene (40 mL). Crude product isolated after work-up was further purified by column chromatography (20-40% EtOAc in hexane) to obtain pure product as a white solid. Yield: 2.53 g, (78%).

Analytical data is in accordance with the literature[13].

Synthesis of *N,N*-bis(4-bromophenyl)pivalamide, 1e

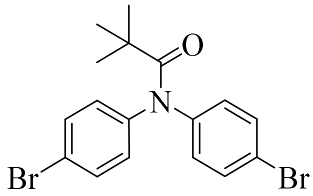

The reaction was performed according to **General procedure A** using pivaloyl chloride (0.44 mL, 3.6 mmol), bis(4-bromophenyl)amine (1.00 g, 3 mmol) and pyridine (0.32 mL, 3.9 mmol) in toluene (5 mL). Crude product isolated after work-up was further purified by column chromatography (10-20% EtOAc in hexane) to obtain pure product as a white solid. Yield: 0.70 g, (57%).

^{1}H NMR (500 MHz, $CDCl_3$) δ 7.48 – 7.49 (d, *J* = 8.5 Hz, 4H), 7.06 – 7.08 (d, *J* = 8.5 Hz, 4H), 1.18 (s, 9H). **^{13}C{^{1}H} NMR** (126 MHz, $CDCl_3$) δ 174.2, 137.8, 127.1, 124.5, 115.4, 36.6, 24.2. **[Acc. Mass]** calcd for [M+H] = $C_{17}H_{18}Br_2NO$: 409.9750, found 409.9743.

Synthesis of *N,N*-bis(4-methylphenyl)pivalamide, 1f

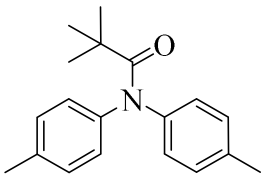

The reaction was performed according to **General procedure A** using pivaloyl chloride (0.78 mL, 6 mmol), di-*p*-tolylamine (0.996 g, 5 mmol) and pyridine (0.52 mL, 6.5 mmol) in toluene (10 mL). Crude product isolated after work-up was further purified by column chromatography (10-20% EtOAc in hexane) to obtain pure product as a white solid. Yield: 0.65 g, (46%).

^{1}H NMR (500 MHz, $CDCl_3$) δ 7.15 – 7.07 (m, 8H), 2.31 (s, 6H), 1.14 (s, 9H). **^{13}C{^{1}H} NMR** (126 MHz, $CDCl_3$) δ 179.6, 142.3, 136.7, 129.8, 128.25, 41.7, 29.8, 21.2. **[Acc. Mass]** calcd for [M+Na] = $C_{19}H_{23}NONa$: 304.1672, found 304.1667.

Synthesis of *N*-(4-bromophenyl)-*N*,2-diphenylacetamide, 1g

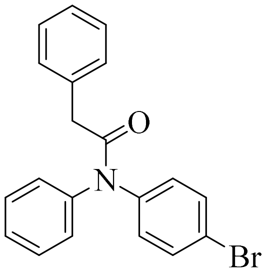

The reaction was performed according to **General procedure A** using phenylacetyl chloride (0.64 mL, 4.83 mmol), 4-bromo-N-phenylaniline (1 g, 4.03 mmol) and pyridine (0.42 mL, 5.24 mmol) in toluene (10 mL). Crude product isolated after work-up was further purified by column chromatography (20-40% EtOAc in hexane) to obtain pure product as a white solid. Yield: 1 g, (68%).

^{1}H NMR (500 MHz, $CDCl_3$) δ 7.45-7.47 (d, *J* = 7.5 Hz, 2H), 7.37-7.40 (t, *J* = 6.5 Hz, 2H), 7.32-7.33 (m, 1H), 7.23-7.29 (m, 3H), 7.19-7.20 (d, *J* = 6.5 Hz, 2H), 7.11-7.13 (t, *J* = 6 Hz, 2H) 3.67 (s, 2H). **^{13}C{^{1}H} NMR** (126 MHz, $CDCl_3$) δ 170.9, 142.3, 141.8, 134.8, 132.0, 129.7, 129.0, 128.5, 127.8, 126.8, 42.2. **[Acc. Mass]** calcd for [M^+] = $C_{20}H_{16}BrNO$: 365.0409, found 365.0394.

Synthesis of 2-phenyl-*N,N*-di-m-tolylacetamide, 1h

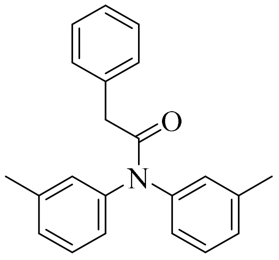

The reaction was performed according to **General procedure A** using phenylacetyl chloride (0.8 mL, 6.08 mmol), di-m-tolylamine (1 g, 5.07 mmol) and pyridine (0.53 mL, 6.59 mmol) in toluene (10 mL). Crude product isolated after work-up was further purified by column chromatography (15-30% EtOAc in hexane) to obtain pure product as a white solid. Yield: 1.2 g, (75%).

^{1}H NMR (500 MHz, $CDCl_3$) δ 7.24 – 7.30 (m, 5H), 7.15-7.16 (d, J = 7 Hz, 2H), 7.04-7.08 (m, 6H) 3.67 (s, 2H), 2.33 (s, 6H). **^{13}C{^{1}H} NMR** (126 MHz, $CDCl_3$) δ 170.9, 142.8, 139.1, 135.4, 129.1, 128.9, 128. 2, 127.8, 126.6, 42.2, 21.2. **[Acc. Mass]** calcd for [M^+] = $C_{22}H_{21}NO$: 315.1617, found 315.1620.

Synthesis of *N,N*-bis(3-fluorophenyl)-2-phenylacetamide, 1i

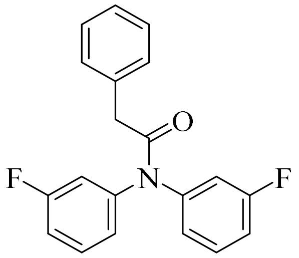

The reaction was performed according to **General procedure A** using phenylacetyl chloride (0.76 mL, 5.84 mmol), di-*m*-tolylamine (1 g, 4.87 mmol) and pyridine (0.5 mL, 6.33 mmol) in toluene (10 mL). Crude product isolated after work-up was further purified by column chromatography (5-20% EtOAc in hexane) to obtain pure product as a sticky solid. Yield: 1.2 g, (76%).

^{1}H NMR (500 MHz, $CDCl_3$) δ 7.24 – 7.36 (m, 7H), 7.00-7.06 (m, 4H), 6.93-6.95 (m, 2H), 3.69 (s, 2H). **^{13}C{^{1}H} NMR** (126 MHz, $CDCl_3$) δ 170.8, 162.9 (d, $^1J_{C\text{-}F}$ = 248.2 Hz), 143.6 (d, $^3J_{C\text{-}F}$ = 9.4 Hz), 134.5, 133.7, 132.8, 129.7 (d, $^2J_{C\text{-}F}$ = 26.3 Hz), 128.9, 128.8 (d, $^3J_{C\text{-}F}$ = 6.9 Hz), 128.6, 127.2, 127.0, 42.4. **^{19}F NMR** (471 MHz, $CDCl_3$) δ -111.85, -111.86, -111.86, -111.87,

-111.88, -111.89, -111.90. **[Acc. Mass]** calcd for [M+H] = $C_{20}H_{16}F_2NO$: 324.1194, found 324.1188.

Synthesis of *N*-phenyl-*N*-vinylacetamide, 1j

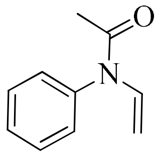

Synthesis of *N*-phenyl-*N*-vinylacetamide was performed according to the reported procedure[14] using N-phenylacetamide (2 g, 14.79 mmol), copper iodide (0.14 g, 0.73 mmol), K_2CO_3 (4.08 g, 29.59 mmol), *N,N*-dimethyl ethylenediamine (0.16 mL, 1.48 mmol) and vinyl bromide (1 M in THF) (29.59 mL, 29.59 mmol). Crude product isolated after work-up was further purified by column chromatography (20-60% EtOAc in hexane) to obtain pure product as a white solid. Yield: 1.3 g, (55%).

Analytical data is in accordance with the literature[14].

Synthesis of *N*-(naphthalen-2-yl)-*N*,2-diphenylacetamide, 1k

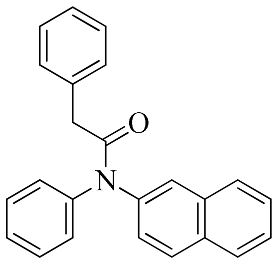

The reaction was performed according to **General procedure A** using phenylacetyl chloride (0.8 mL, 6 mmol), *N*-phenyl-2-naphthylamine (1.1 g, 5 mmol), pyridine (0.52 mL, 6.5 mmol) in toluene (6 mL). Crude product isolated after work-up was further purified by column chromatography (10-30% EtOAc in hexane) to obtain pure product as off-white solid. Yield: 0.67 g, (40%).

^{1}H NMR (500 MHz, $CDCl_3$) δ 7.78 (m, 3H), 7.63 (m, 1H), 7.58 – 7.06 (m, 13H), 3.71 (s, 2H). **^{13}C{^{1}H} NMR** (126 MHz, $CDCl_3$) δ 171.1, 142.9, 140.3, 135.2, 133.6, 132.0, 129.2, 129.1, 128.3, 127.9, 127.6, 126.7, 126.5, 42.3. **[Acc. Mass]** calcd for [M+Na] = $C_{24}H_{19}NONa$: 360.1359, found 360.1356.

Synthesis of *N*-(naphthalen-2-yl)-*N*-phenylpivalamide, 1l

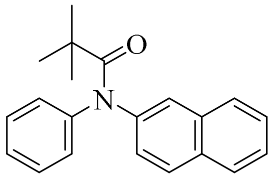

The reaction was performed according to **General procedure A** using pivaloyl chloride (0.73 mL, 6.02 mmol), *N*-phenyl-2-naphthylamine (1.1 g, 5.01 mmol), pyridine (0.52 mL, 6.52 mmol) in toluene (6 mL). Crude product isolated after work-up was further purified by column chromatography (20-30% EtOAc in hexane) to obtain pure product as off-white solid. Yield: 0.72 g, (47%).

^{1}H NMR (500 MHz, $CDCl_3$) δ 7.82-7.84 (m, 2H), 7.77-7.79 (m, 1H), 7.63 (d, J = 2.5 Hz, 1H), 7.48-7.51 (m, 2H), 7.36-7.40 (m, 3H), 7.25-7.31 (m, 3H), 1.21 (s, 9H). **^{13}C{^{1}H} NMR** (126 MHz, $CDCl_3$) δ 180.0, 144.4, 142.0, 133.5, 131.9, 129.1, 128.9, 128.4, 127.8, 127.7, 126.9, 126.8, 126.5, 126.3, 41.8, 29.7. **[Acc. Mass]** calcd for [M+H] = $C_{21}H_{22}NO$: 304.1673, found 304.1679.

Synthesis of *N*-(dibenzo[b,d]thiophen-3-yl)-*N*-phenylpivalamide, 1m

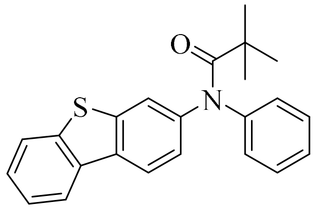

The reaction was performed according to **General procedure A** using pivaloyl chloride (0.1 mL, 0.78 mmol), N-phenyldibenzo[b,d]thiophen-3-amine (0.18 g, 0.65 mmol) and pyridine (0.07 mL, 0.84 mmol) in toluene (5 mL). Crude product isolated after work-up was further purified by column chromatography (40-80% DCM in hexane) to obtain pure product as a white solid. Yield: 0.2 g, (85%).

^{1}H NMR (500 MHz, $CDCl_3$) δ 8.11-8.14 (m, 2H), 7.84-7.86 (m, 1H), 7.69-7.70 (d, J = 1.5 Hz, 1H), 7.46-7.48 (m, 2H), 7.35-7.40 (m, 3H), 7.27-7.32 (m, 3H), 1.21 (s, 9H). **^{13}C{^{1}H} NMR** (126 MHz, $CDCl_3$) δ 179.9, 144.4, 143.2, 139.9, 139.8, 134.9, 134.1, 129.2, 128.5, 127.1, 126.8, 125.0, 124.6, 122.8, 122.3, 121.8, 121.6, 41.9, 29.7. **[Acc. Mass]** calcd for [M+H] = $C_{23}H_{22}NOS$: 360.1416, found 360.1415.

Synthesis of *N*-phenyl-*N*-(9-phenyl-9H-carbazol-2-yl)pivalamide, 1n

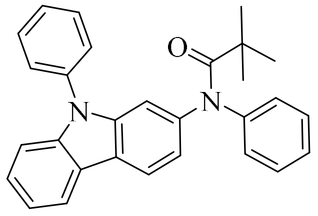

The reaction was performed according to **General procedure A** using pivaloyl chloride (0.39 mL, 3.23 mmol), N-phenyldibenzo[b,d]thiophen-3-amine (0.9 g, 2.69 mmol) and pyridine (0.28 mL, 3.49 mmol) in toluene (20 mL). Crude product isolated after work-up was further purified by column chromatography (50-90% DCM in hexane) to obtain pure product as a white solid. Yield: 0.94 g, (88%).

^{1}**H NMR** (500 MHz, $CDCl_3$) δ 8.11-8.13 (t, *J* = 9 Hz, 2H), 7.62-7.65 (m, 2H), 7.49-7.55 (m, 3H), 7.40-7.45 (m, 2H), 7.30-7.34 (m, 3H), 7.27-7.28 (d, 1.5 Hz, 1H), 7.18-7.25 (m, 4H), 1.18 (s, 9H). ^{13}**C{^{1}H} NMR** (126 MHz, $CDCl_3$) δ 179.9, 145.1, 142.1, 141.6, 141.0, 137.2, 130.0, 128.9, 127.8, 127.0, 126.4, 126.2, 124.8, 122.8, 122.3, 121.1, 120.8, 120.3, 110.3, 109.9, 41.9, 29.7. **[Acc. Mass]** calcd for [M+H] = $C_{29}H_{27}N_2O$: 419.2117, found 419.2120.

Synthesis of *N,N'*-diphenyl-1,4-phenylenedipivalamide, 1o

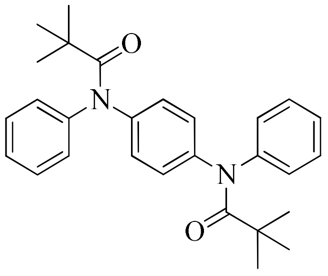

The reaction was performed according to **General procedure A** using pivaloyl chloride (1.47 mL, 12 mmol), *N,N'*-diphenyl-1,4-phenylenediamine (1.302 g, 5 mmol), pyridine (1.05 mL, 13 mmol) in toluene (6 mL). Crude product isolated after work-up was further purified by column chromatography (20-50% DCM in hexane) to obtain pure product as a off-white solid. Yield: 0.62 g, (29%).

^{1}**H NMR** (500 MHz, $CDCl_3$) δ 7.34-7.37 (m, 4H), 7.22-7.28 (m, 6H), 7.16 (s, 4H), 1.13 (s, 18H). ^{13}**C{^{1}H} NMR** (126 MHz, $CDCl_3$) δ 179.7, 143.9, 142.8, 129.2, 128.8, 128.4, 127.3, 41.8, 29.7. **[Acc. Mass]** calcd for [M+H] = $C_{28}H_{33}N_2O_2$: 429.2537, found 429.2536.

Synthesis of *N*-[4-[4-(*N*-acetylanilino)phenyl]phenyl]-*N*-phenylpivalamide, 1p

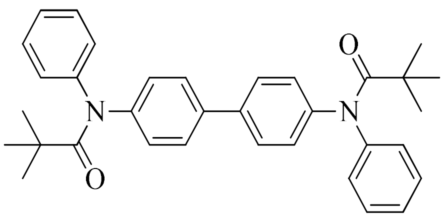

The reaction was performed according to **General procedure A** using pivaloyl chloride (1.4 mL, 12 mmol), *N,N'*-diphenyl-p-phenylenediamine (1.70 g, 5 mmol), pyridine (1.05 mL, 13 mmol.) in toluene (6 mL). Crude product isolated after work-up was further purified by column chromatography (20-50% DCM in hexane) to obtain the product as a white solid. Yield: 0.09 g, (4%).

^{1}H NMR (500 MHz, $CDCl_3$) δ 7.58-7.59 (m, 4H), 7.39-7.42 (m, 4H), 7.29-7.32 (m, 10H), 1.18 (s, 18H). **^{13}C{^{1}H} NMR** (126 MHz, $CDCl_3$) δ 179.3, 144.5, 144.3, 138.5, 129.1, 128.7, 128.6, 127.6, 126.9, 41.6, 29.4. **[Acc. Mass]** calcd for [M+H] = $C_{34}H_{37}N_2O_2$: 505.2850, found 505.2841.

Borylation of *N,N*-2-triphenylacetanamide (1a)

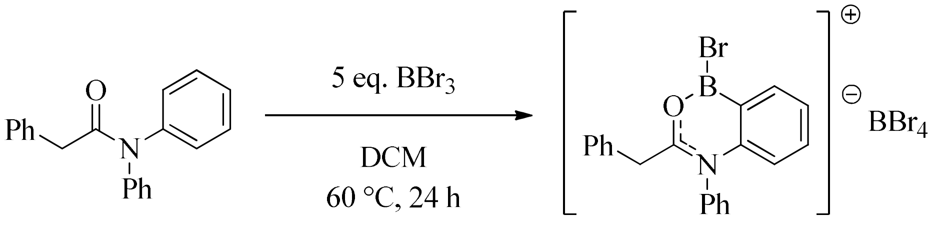

1a **2a**

1a (0.772 g, 2.51 mmol) was added to an ampule fitted with a J-Youngs tap. The amide was dissolved in DCM (10 mL) and BBr_3 (neat, 5 eq., 1.2 mL) was added slowly. The ampule was sealed, and the reaction mixture heated to 60 °C for 24 h. Solvents/volatiles were removed under vacuum and the product dried to obtain **2a** as a grey solid (1.731 g, 97%).

^{1}H NMR (400 MHz, CD_2Cl_2) δ 7.98 (d, J = 7.4 Hz, 1H), 7.65-7.80 (m, 3H), 7.54 (td, J = 7 and 1 Hz, 1H), 7.28-7.43 (m, 6H), 7.06-7.16 (m, 2H), 6.44 (d, J = 8.4 Hz, 1H), 4.02 (s, 2H). **^{11}B NMR** (128 MHz, CD_2Cl_2) δ 21.29, 12.11. **^{13}C{^{1}H} NMR** (126 MHz, $CDCl_3$) δ 170.4, 138.6, 135.5, 135.0, 132.2, 131.9, 131.7, 130.8, 130.3, 129.7, 129.6, 128.9, 128.1, 118.4, 42.2. **Elemental analysis** calculated for $C_{20}H_{16}B_2Br_5NO$: C: 33.95, H: 2.28, N: 1.98, found (average of two runs) C: 34.78, H:2.20, N: 2.03.

In-situ reaction monitoring

N-ethylphenyl

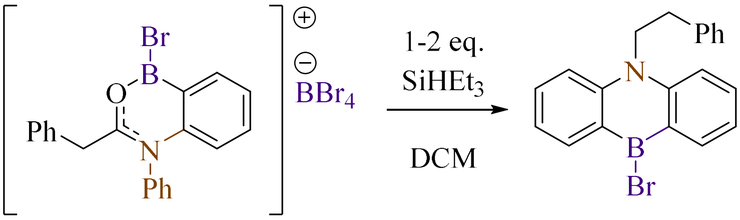

2a-Br$_2$

3a-Br
d$_{11B}$=50.1 ppm

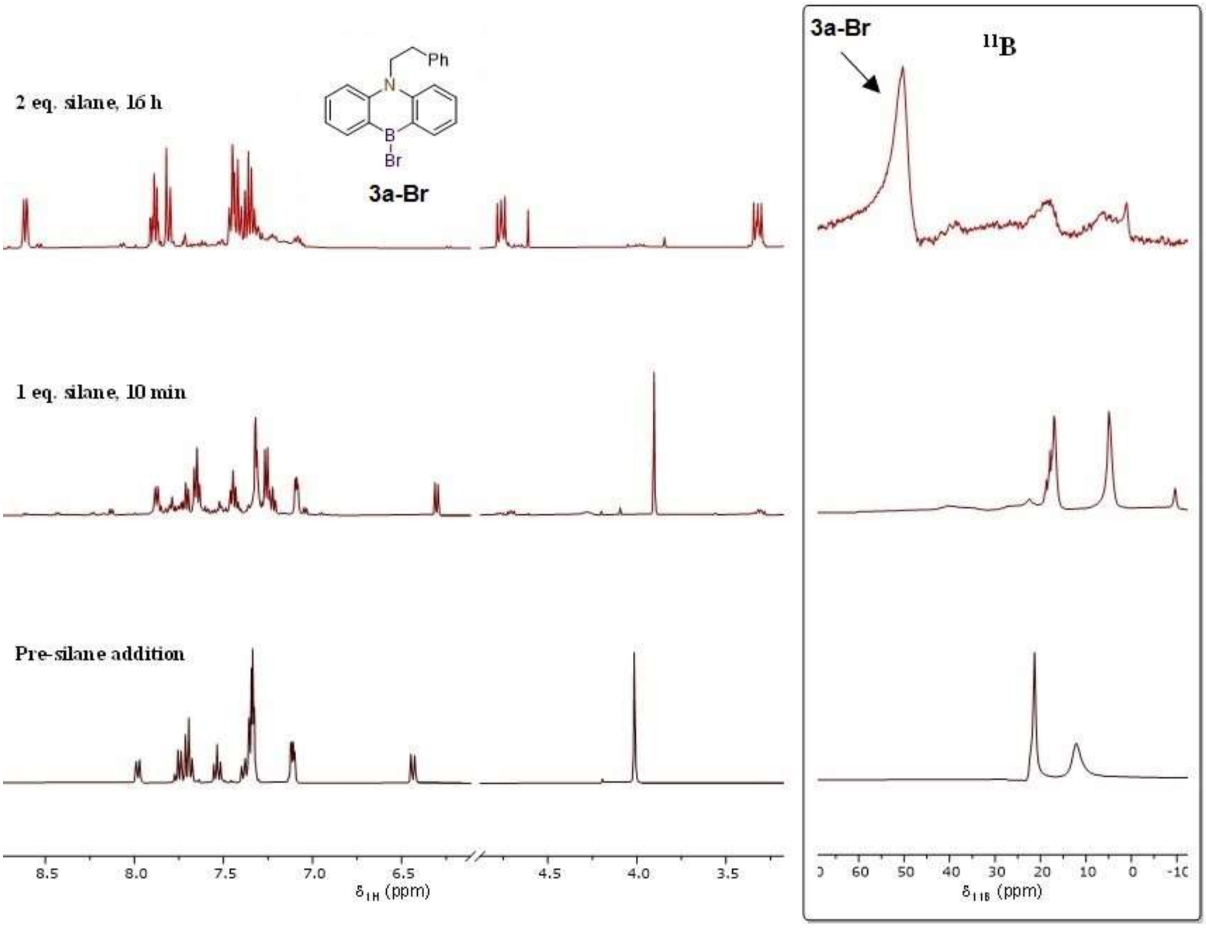

Figure **S1.** Stacked *in-situ* ^{1}H and ^{11}B (inset) NMR spectra (in DCM) showing conversion of **2a-Br$_2$** to **3a-Br**.

N-hexanoyl

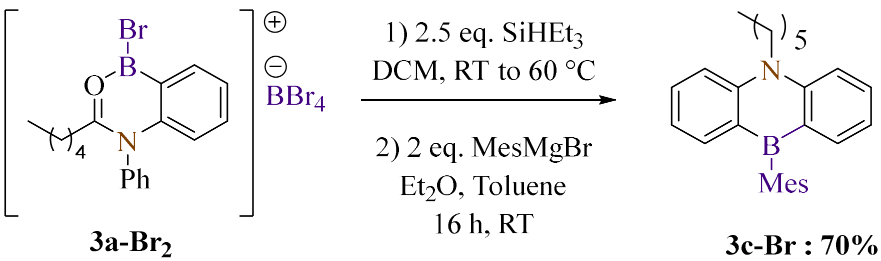

3a-Br$_2$
Formed *in-situ*

3c-Br : 70%

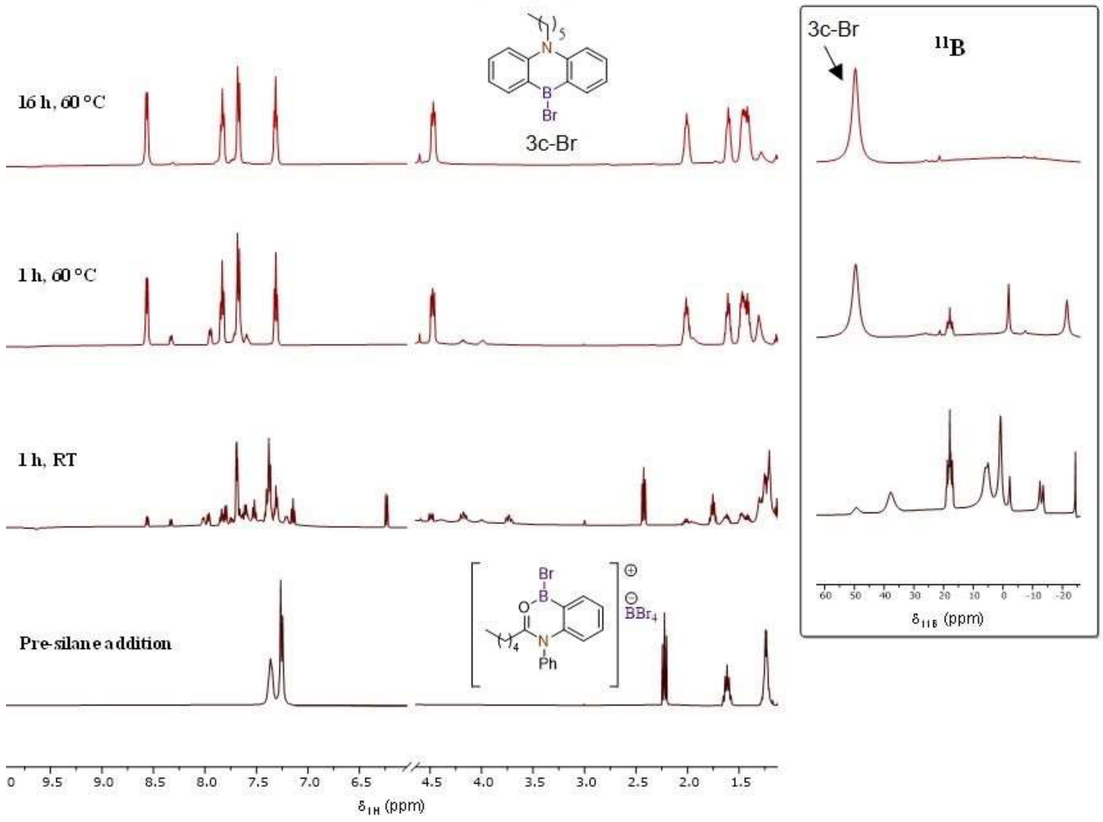

Figure **S2.** Stacked ^{1}H and ^{11}B (inset) NMR spectra (in DCM) showing conversion to **2c-Br$_2$ to 3c-Br**.

N-Benzoyl

After the reduction/borylation process a second species was removed from the column contained two resonances in the ^{11}B NMR spectrum at δ_{11B}=48.3 and 33.3 ppm with the latter in the region for an aryl–B(OH)$_2$ type species. The corresponding ^{1}H NMR spectrum exhibited four resonances in the aliphatic region, two of which were integrated to 3 protons each while the other two were for 6 protons each. Therefore, it was proposed that the species contains two boron atoms, one of which is doubly substituted with mesityl protecting groups. Interestingly, the total number of protons in the aromatic region of the ^{1}H NMR spectrum amounts to 13 and a lack of a singlet at δ_{1H}~5 ppm **(Figure S3)**, suggesting a single C–H borylation and precluding a benzyl substituent in the product, thus it was consistent with reduction and cleavage of the benzoyl/benzyl moiety occurring. Based on this data coupled with literature examples of N–BMes$_2$ compounds (including column stable examples) that resonate at δ_{11B}=47-49 ppm, the product was suggested to be a boroxine derivative of Ar$_2$N=BMes$_2$, with an isolated yield of 35% assuming this formulation is correct **(Figure S3)**.

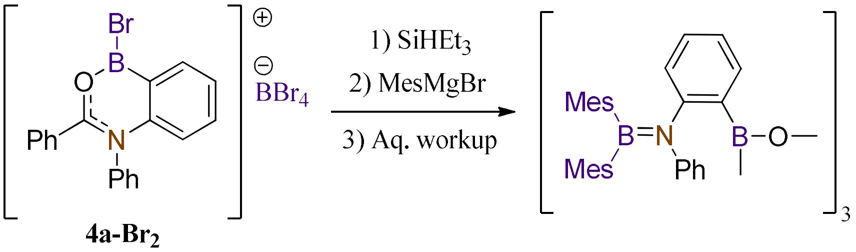

4a-Br$_2$
Formed *in-situ*

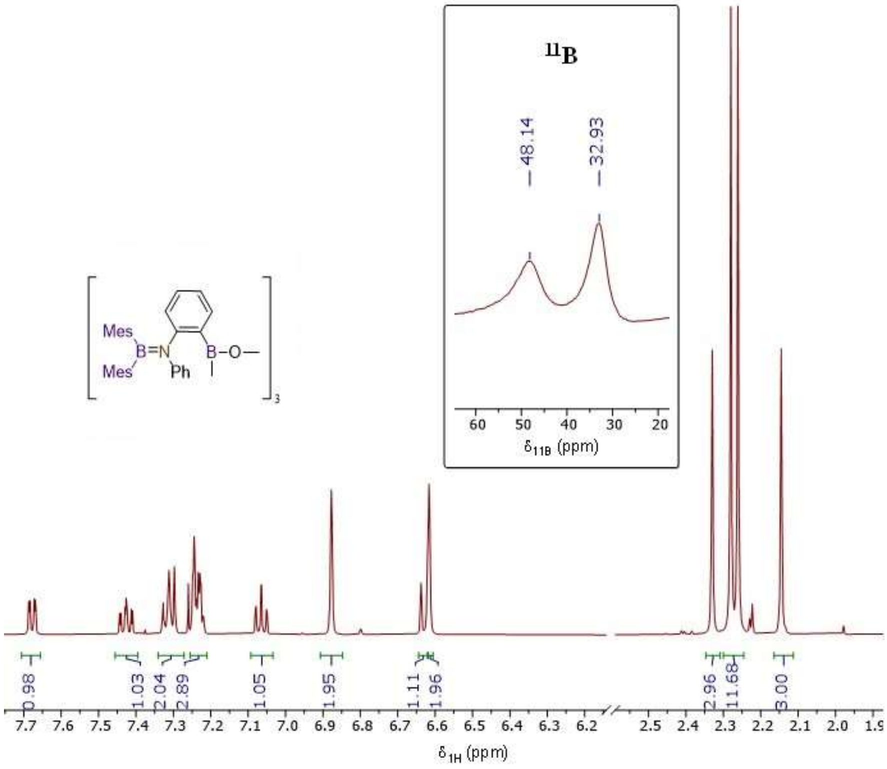

Figure **S3.** ^{1}H and ^{11}B (inset) NMR spectra of **4d-byproduct**.

This suggests the benzyl group is somewhat unstable under forcing reaction conditions in the presence of reductants. A proposed mechanism for the formation of the side-product consists of three hydride transfers – to the amide electrophilic carbon – which results in complete cleavage of the benzyl group. $SiHEt_3$ has been shown to be effective in the cleavage of *N*-benzyl protecting groups at 130 °C in the presence of a base. Furthermore, it is likely that the presence of silyliums and borenium cations *in-situ* activate the carbonyl (and reduction products e.g., by coordination to N) for further reduction by either SiH– or BH– type species. It is possible the second boron incorporation into the product is a result of reduction with (*in-situ* formed) diborane from which the resultant boron species is coordinated to the amine (or coordination prior to reduction to activate the B–H). As <4 equivalents of MesMgBr were employed in this experiment, complete arylation of all boron centres does not occur and thus the species is assumed to exist as a boroxine, with the other boron stabilised by double-mesityl substitution **(**Figure **S4)**.

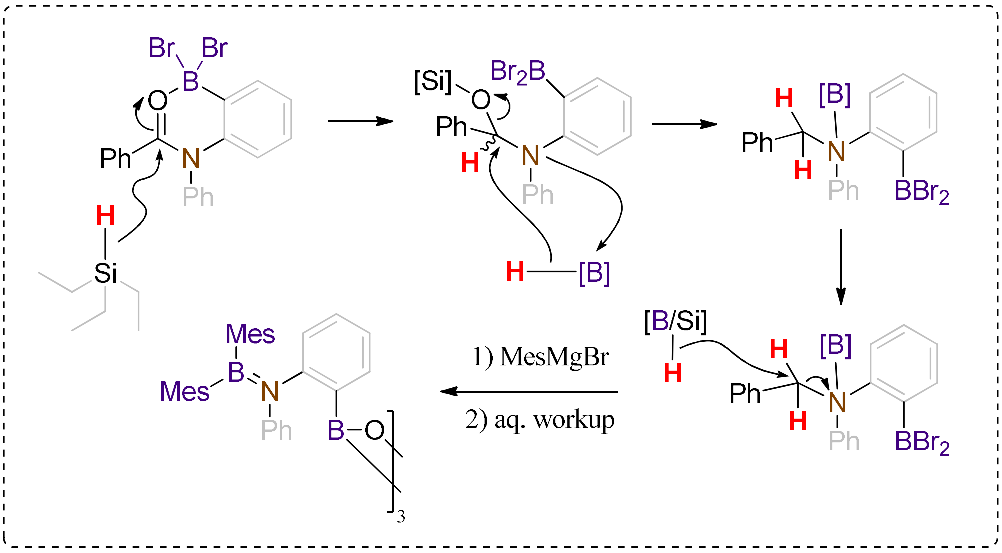

Figure **S4.** Proposed mechanism for the synthesis of **4d-byproduct** by N-benzyl cleavage.

Synthesis of 1,4-azaborines (General Procedure C)

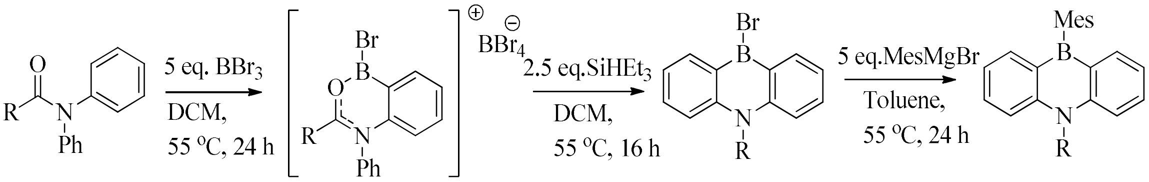

The corresponding diarylamide (1 eq.) was added to a sealable reaction vessel. The amide was dissolved in DCM (x mL), after which BBr_3 (5 eq) was added, the vessel was sealed with a J-Youngs tap and the reaction mixture stirred at 55 °C for 24 hrs. The solvents/volatiles were removed under vacuum and the crude borylated product was dried for 1 h under vacuum to remove residual BBr_3. The borylated product was again dissolved in DCM (x mL), after which $SiHEt_3$ (2.5 eq.) was added, the vessel was sealed and stirred at 55 °C for up to 16 hrs. The solvents/volatiles were removed under vacuum and the crude azaborine dried. The product was suspended in toluene (x mL) and MesMgBr (1M in Et_2O, 5 eq.) was added, the vessel was sealed and stirred at 55 °C for 24 hrs. The reaction mixture was quenched with water and extracted with ethyl acetate and the crude product was purified by column chromatography on silica-gel (5-40 % dichloromethane in hexane).

Synthesis of 10-mesityl-5-phenethyl-5,10-dihydrodibenzo[b,e][1,4]azaborinine (4a)

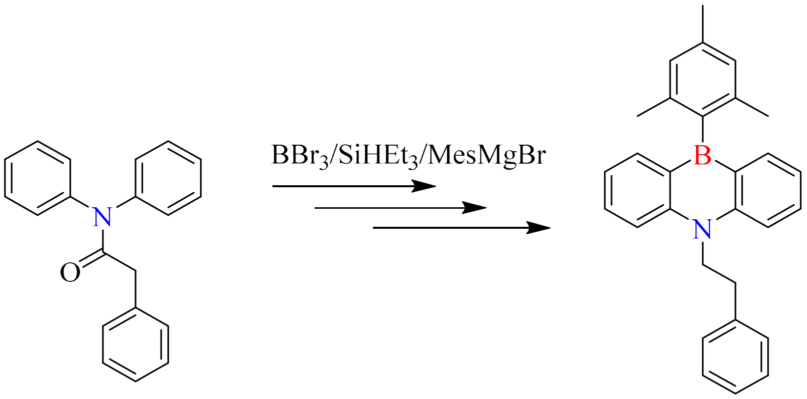

The synthesis was performed according to the **General Procedure C** using *N,N*-2-triphenylacetanamide (1g, 3.48 mmol), BBr_3 (1.65 mL, 17.39 mmol), $SiHEt_3$ (1.34 mL, 8.39 mmol), and MesMgBr (17.26 mL, 17.26 mmol, 1M in diethyl ether) in DCM (20 mL), and toluene (20 mL). The crude product was purified by column chromatography (5-40 % dichloromethane in hexane) followed by washing with hexane to obtain the product as white solid. Yield: 0.94 g (67%).

^{1}H NMR (500 MHz, $CDCl_3$) δ 7.92-7.94 (m, 2H), 7.81-7.84 (m, 4H), 7.48-7.53 (m, 4H), 7.36-7.38 (m, 1H), 7.19-22 (m, 2H), 7.00 (s, 2H), 4.81-4.85 (t, J = 8.5 Hz, 2H), 3.39-3.43 (t, J = 8.5 Hz,, 2H), 2.45 (s, 3H), 2.01 (s, 6H). **^{11}B NMR** (160 MHz, $CDCl_3$) δ 53.58. **^{13}C{^{1}H} NMR** (126 MHz, $CDCl_3$) δ 145.2, 139.3, 138.3, 137.9, 136.4, 133.7, 129.0, 128.7, 127.0, 126.8, 119.8, 114.3, 48.7, 33.5, 23.3, 21.3. **[Acc. Mass]** calcd for [M+H] = $C_{29}H_{29}BN$: 402.2388, found 402.2380.

Synthesis of 10-mesityl-5-neopentyl-5,10-dihydrodibenzo[b,e][1,4]azaborinine (4b)

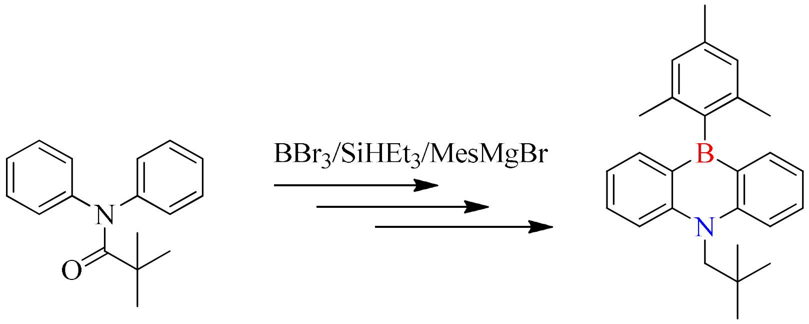

The synthesis was performed according to the **General Procedure C** using *N,N*-diphenylpivalamide (0.1 g, 0.39 mmol), BBr_3 (0.18 mL, 1.97 mmol), $SiHEt_3$ (0.15 mL, 0.98 mmol), and MesMgBr (1.96 mL, 1.96 mmol, 1M in diethyl ether) in DCM (2 mL), and toluene (2 mL). The crude product was purified by column chromatography (5-10 % dichloromethane in hexane) followed by washing with pentane to obtain the product as white solid. Yield: 0.11 g (78%).

^{1}H NMR (500 MHz, $CDCl_3$) δ 7.73-7.91 (m, 4H), 7.69-7.72 (m, 2H), 7.11-7.14 (m, 2H), 7.00 (s, 1H), 6.97 (s, 1H), 4.75 (s, 2H), 2.44 (s, 3H), 2.03 (s, 3H), 1.95 (s, 3H), 1.01 (s, 9H). **^{11}B NMR** (160 MHz, $CDCl_3$) δ 53.26. **^{13}C{^{1}H} NMR** (126 MHz, $CDCl_3$) δ 146.9, 139.2, 139.1, 137.5, 136.2, 132.5, 126.8, 126.7, 119.4, 116.8, 53.5, 35.6, 29.9, 23.1, 23.0, 21.3. **[Acc. Mass]** calcd for [M+H] = $C_{26}H_{31}BN$: 368.2779, found 368.2791.

Synthesis of 5-hexyl-10-mesityl-5,10-dihydrodibenzo[b,e][1,4]azaborinine (4c)

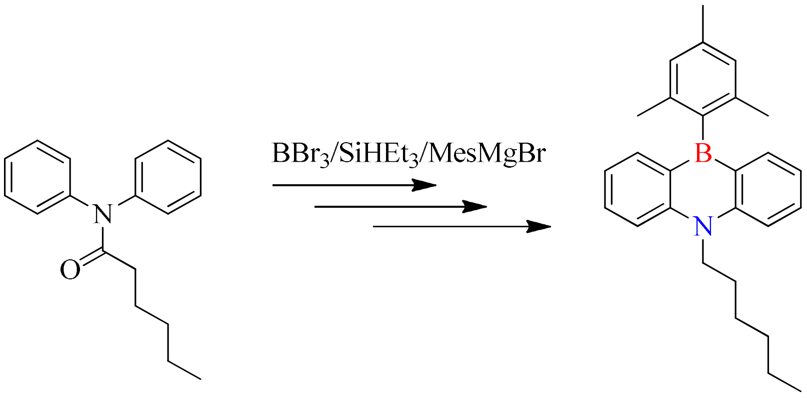

The synthesis was performed according to the **General Procedure C** using *N,N*-diphenylhexanamide (0.5 g, 1.87 mmol), BBr_3 (0.88 mL, 9.35 mmol), $SiHEt_3$ (0.74 mL, 4.64 mmol), and MesMgBr (7.34 mL, 7.36 mmol, 1M in diethyl ether) in DCM (5 mL), and toluene (5 mL). The crude product was purified by column chromatography (5-30 % dichloromethane in hexane) followed by washing with hexane to obtain the product as white solid. Yield: 0.48 g (68%).

^{1}H NMR (500 MHz, $CDCl_3$) δ 7.88-7.90 (dd, J = 7.5 and 2 Hz, 2H), 7.77-7.88 (m, 2H), 7.67-7.69 (d, J = 8.5 Hz, 2H), 7.14-7.17 (t, J = 7 Hz, 2H), 6.98 (s, 2H), 4.53-4.55 (t, J = 7 Hz, 2H), 2.43 (s, 3H), 2.12-2.17 (m, 2H), 1.68 (s, 6H), 1.51-1.56 (m, 2H), 1.45-1.49 (m, 4H), 1.02 (t, J = 7 Hz, 3H). **^{11}B NMR** (160 MHz, $CDCl_3$) δ 53.60. **^{13}C{^{1}H} NMR** (126 MHz, $CDCl_3$) δ 145.4, 139.3, 137.7, 136.3, 133.5, 126.8, 119.4, 114.6, 47.9, 31.6, 27.3, 26.7, 23.2, 22.7, 21.3, 14.1. **[Acc. Mass]** calcd for [M+H] = $C_{27}H_{33}BN$: 382.2701, found 382.2693.

Synthesis of 5-benzyl-10-mesityl-5,10-dihydrodibenzo[b,e][1,4]azaborinine (4d)

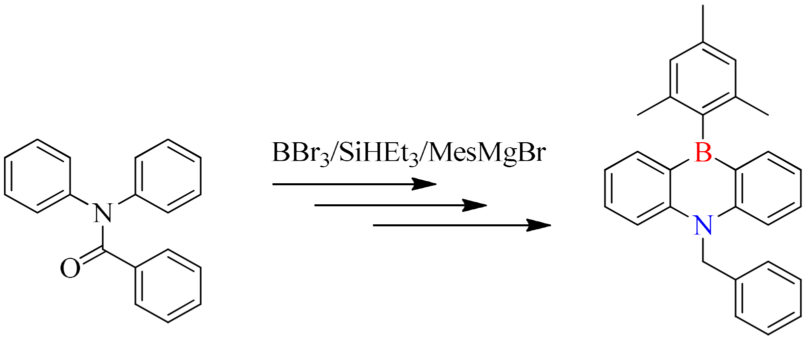

The synthesis was performed according to the **General Procedure C** using *N,N*-diphenylbenzamide (0.25 g, 0.91 mmol), BBr_3 (1.65 mL, 17.39 mmol), $SiHEt_3$ (0.36 mL, 2.28

mmol), and MesMgBr (4.56 mL, 4.55 mmol, 1M in diethyl ether) in DCM (2.5 mL), and toluene (2.5 mL). The crude product was purified by column chromatography (5-40 % dichloromethane in hexane) followed by washing with hexane to obtain the product as white solid. Yield: 0.15 g (42%).

^{1}H NMR (500 MHz, $CDCl_3$) δ 7.91-7.93 (dd, *J* = 7.5 and 2 Hz, 2H), 7.66-7.69 (m, 2H), 7.50-7.66 (d, *J* = 8.5 Hz, 2H), 7.36-7.51 (m, 2H), 7.30-7.35 (m, 3H), 7.15-7.18 (t, *J* = 7.5 Hz, 2H), 7.00 (s, 2H), 5.81 (s, 2H), 2.45 (s, 3H), 2.05 (s, 6H). **^{11}B NMR** (160 MHz, $CDCl_3$) δ 53.29. **^{13}C{^{1}H} NMR** (126 MHz, $CDCl_3$) δ 146.3, 139.3, 137.5, 136.8, 136.4, 129.1, 127.5, 126.9, 125.9, 125.9, 119.9, 115.5, 52.7, 23.3, 21.3. **[Acc. Mass]** calcd for [M^+] = $C_{28}H_{26}BN$: 387.2153, found 387.2143.

Synthesis of 2,8-dibromo-10-mesityl-5-neopentyl-5,10-dihydrodibenzo[b,e][1,4]azaborinine (4e)

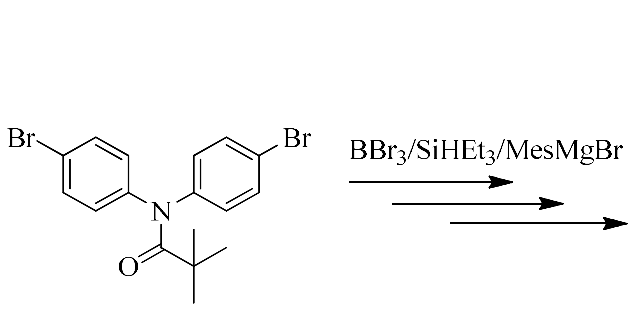

The synthesis was performed according to the **General Procedure C** using *N,N*-bis(4-bromophenyl)pivalamide (0.5 g, 1.21 mmol), BBr_3 (0.57 mL, 6.08 mmol), $SiHEt_3$ (0.48 mL, 3.02 mmol), and MesMgBr (5.96 mL, 5.96 mmol, 1M in diethyl ether) in DCM (5 mL), and toluene (5 mL). The crude product was purified by column chromatography (5-30 % dichloromethane in hexane) followed by washing with hexane to obtain the product as yellow solid. Yield: 0.41 g (65%).

^{1}H NMR (500 MHz, $CDCl_3$) δ 7.90-7.91 (d, *J* = 2.5 Hz, 2H), 7.74-7.79 (m, 4H), 6.99 (s, 1H), 6.96 (s, 1H), 4.67 (s, 2H), 2.44 (s, 3H), 2.00 (s, 3H), 1.91 (s, 3H), 0.93 (s, 9H). **^{11}B NMR** (160 MHz, $CDCl_3$) δ 53.53. **^{13}C{^{1}H} NMR** (126 MHz, $CDCl_3$) δ 145.5, 139.0, 136.8, 135.6, 127.1, 119.1, 113.4, 54.1, 35.8, 29.8, 23.2, 21.3. **[Acc. Mass]** calcd for [M+H] = $C_{26}H_{29}BBr_2N$: 524.0753, found 524.0765.

Synthesis of 10-mesityl-2,8-dimethyl-5-neopentyl-5,10-dihydrodibenzo[b,e][1,4]azaborinine (4f)

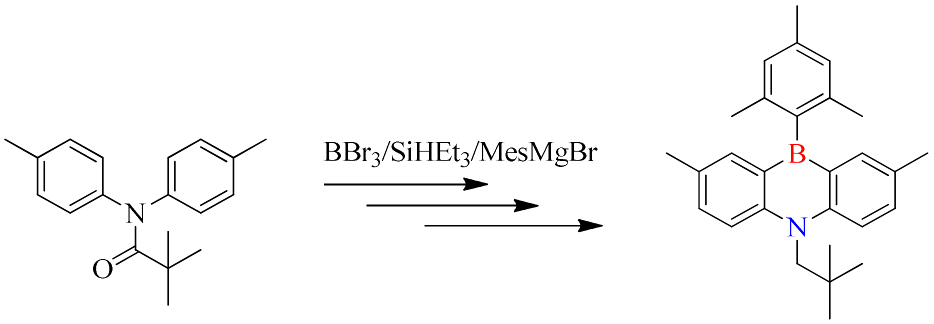

The synthesis was performed according to the **General Procedure C** using *N,N*-di-p-tolylpivalamide (0.25 g, 0.88 mmol), BBr_3 (0.42 mL, 4.44 mmol), $SiHEt_3$ (0.35 mL, 2.22 mmol), and MesMgBr (4.35 mL, 4.35 mmol, 1M in diethyl ether) in DCM (2.5 mL), and toluene (2.5 mL). The crude product was purified by column chromatography (5-40 % dichloromethane in hexane) followed by washing with hexane to obtain the product as white solid. Yield: 0.23 g (63%).

^{1}H NMR (601 MHz, $CDCl_3$) δ 7.77-7.79 (d, *J* = 9.0 Hz, 2H), 7.59-7.60 (d, *J* = 1.5 Hz, 2H), 7.49-7.52 (dd, *J* = 9 and 2.5 Hz, 2H), 7.00 (s, 1H), 6.97 (s, 1H), 4.69 (s, 2H), 2.45 (s, 3H), 2.36 (s, 6H), 2.03 (s, 3H), 1.95 (s, 3H), 0.99 (s, 9H). **^{11}B NMR** (160 MHz, $CDCl_3$) δ 52.10. **^{13}C{^{1}H} NMR** (151 MHz, $CDCl_3$) δ 145.0, 139.2, 136.5, 135.9, 133.9, 128.1, 126.7, 126.6, 116.6, 53.5, 35.6, 29.9, 23.2, 21.4, 20.4. **[Acc. Mass]** calcd for [M^+] = $C_{28}H_{34}BN$: 395.2779, found 395.2791.

Synthesis of 2-Br-10-mesityl-5-phenethyl-5,10-dihydrodibenzo[b,e][1,4]azaborinine (4g)

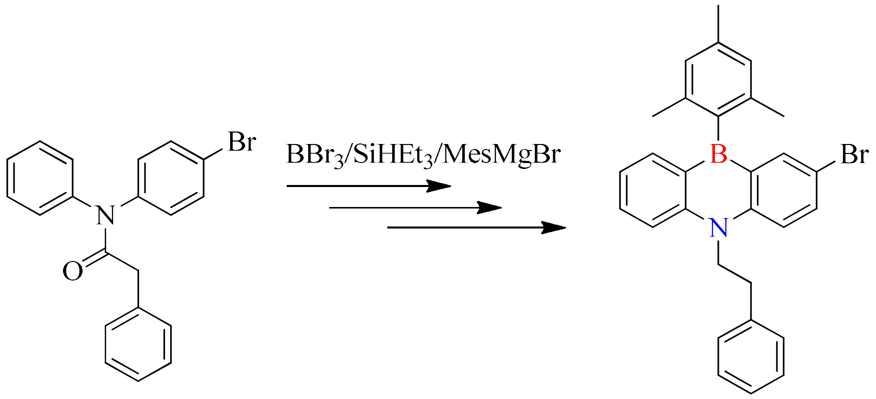

The synthesis was performed according to the **General Procedure C** using *N*-(4-bromophenyl)-*N*,2-diphenylacetamide (0.5 g, 1.36 mmol), BBr_3 (0.65 mL, 6.82 mmol), $SiHEt_3$ (0.54 mL, 3.41 mmol), and MesMgBr (6.8 mL, 6.82 mmol, 1M in diethyl ether) in DCM (5

mL), and toluene (5 mL). The crude product was purified by column chromatography (5-10 % dichloromethane in hexane) followed by washing with hexane to obtain the product as yellow solid. Yield: 0.47 g (72%).

^{1}H NMR (500 MHz, $CDCl_3$) δ 7.98-7.99 (d, *J* = 3 Hz, 1H), 7.79-7.90 (m, 4H), 7.67-7.69 (d, *J* = 9 Hz, 1H), 7.44-7.47 (m, 4H), 7.35-7.39 (m, 1H), 7.19-7.22 (t, *J* = 6.5 Hz, 1H), 6.99 (s, 2H), 4.78-4.81 (t, *J* = 9 Hz, 2H), 3.36 – 3.39 (t, *J* = 8 Hz, 2H), 2.45 (s, 3H), 1.99 (s, 6H). **^{11}B NMR** (160 MHz, $CDCl_3$) δ 53.79. **^{13}C{^{1}H} NMR** (126 MHz, $CDCl_3$) δ 145.0, 143.9, 139.3, 139.2, 138.1, 138.0, 136.6, 136.3, 134.1, 129.1, 128.6, 127.1, 126.9, 120.1, 116.6, 114.4, 113.1, 48.8, 33.5, 23.3, 21.3. **[Acc. Mass]** calcd for [M+H] = $C_{29}H_{28}BBrN$: 480.1492, found 480.1476.

Synthesis of 10-mesityl-3,7-dimethyl-5-phenethyl-5,10-dihydrodibenzo[b,e][1,4]azaborinine (4h)

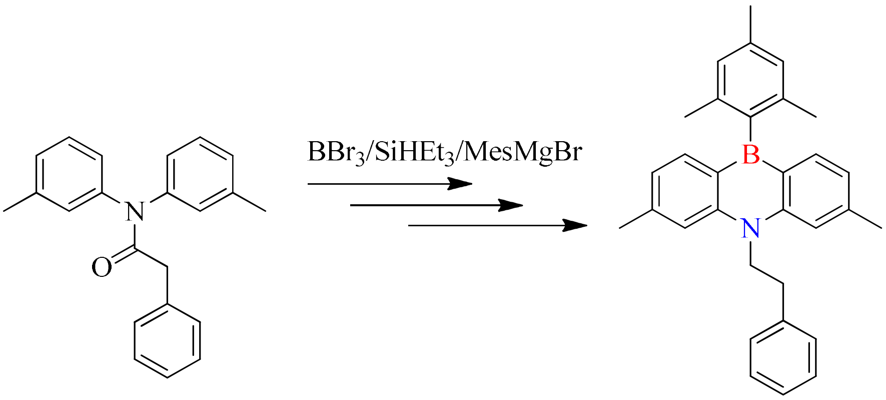

The synthesis was performed according to the **General Procedure C** using 2-phenyl-*N,N*-di-*m*-tolylacetamide (0.3 g, 0.95 mmol), BBr_3 (0.45 mL, 4.75 mmol), $SiHEt_3$ (0.38 mL, 2.37 mmol), and MesMgBr (4.75 mL, 4.75 mmol, 1M in diethyl ether) in DCM (3 mL), and toluene (3 mL). The crude product was purified by column chromatography (5-30 % dichloromethane in hexane) followed by washing with hexane to obtain the product as off-white solid. Yield: 0.26 g (64%).

^{1}H NMR (500 MHz, $CDCl_3$) δ 7.78-7.79 (d, *J* = 7.5 Hz, 2H), 7.56 (s, 2H), 7.47-7.56 (m, 4H) 7.37-7.40 (m, 1H), 7.01-7.03 (d, *J* = 7.5 Hz, 2H), 6.98 (s, 2H), 4.78-4.81 (t, *J* = 9 Hz, 2H), 3.38 – 3.42 (t, *J* = 8 Hz, 2H), 2.62 (s, 6H), 2.44 (s, 3H), 2.01 (s, 6H). **^{11}B NMR** (160 MHz, $CDCl_3$) δ 53.02. **^{13}C{^{1}H} NMR** (126 MHz, $CDCl_3$) δ 145.6, 143.9, 139.4, 138.6, 137.8, 136.2, 129.0, 128.7, 126.9, 126.7, 121.2, 114.5, 48.4, 33.6, 23.2, 22.9, 21.3. **[Acc. Mass]** calcd for [M+H] = $C_{31}H_{33}BN$: 430.2700, found 430.2706.

Synthesis of 3,7-difluoro-10-mesityl-5-phenethyl-5,10-dihydrodibenzo[b,e][1,4]azaborinine (4i)

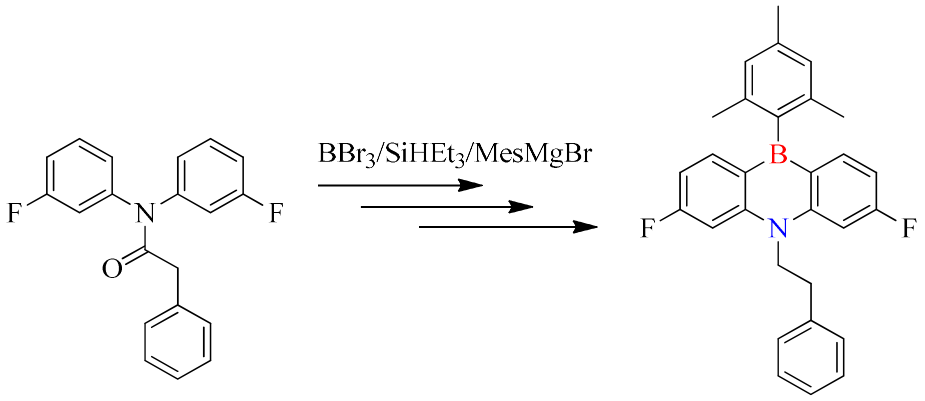

The synthesis was performed according to the **General Procedure C** using 2-phenyl-*N,N*-bis(3-fluorophenyl)-2-phenylacetamide (0.3 g, 0.92 mmol), BBr_3 (0.44 mL, 4.63 mmol), $SiHEt_3$ (0.37 mL, 2.32 mmol), and MesMgBr (4.5 mL, 4.57 mmol, 1M in diethyl ether) in DCM (3 mL), and toluene (3 mL). The crude product was purified by column chromatography (5-10 % dichloromethane in hexane) followed by washing with pentane to obtain the product as sticky solid. Yield: 0.22 g (55%).

^{1}H NMR (500 MHz, $CDCl_3$) δ 7.86-7.89 (t, J = 8 Hz, 2H), 7.45-7.47 (m, 4H), 7.36-7.41 (m, 3H), 6.98 (s, 2H), 6.91-6.95 (td, J = 8 and 2 Hz, 2H), 4.64-4.68 (t, J = 8.5 Hz, 2H), 3.33-3.36 (t, J = 8.5 Hz, 2H), 2.43 (s, 3H), 1.98 (s, 6H). **^{11}B NMR** (160 MHz, $CDCl_3$) δ 52.46. **^{13}C{^{1}H} NMR** (126 MHz, $CDCl_3$) δ 166.67 (d, $^1J_{C-F}$ = 250.2 Hz), 147.0 (d, $^3J_{C-F}$ = 10.4 Hz), 140.6 (d, $^3J_{C-F}$ = 10.4 Hz), 139.2, 137.6, 136.7, 129.1, 128.7, 127.2, 126.9, 108.8 (d, $^2J_{C-F}$ = 21.3 Hz), 100.8 (d, $^2J_{C-F}$ = 26.3 Hz), 49.2, 33.1, 23.1, 21.3. **^{19}F NMR** (471 MHz, $CDCl_3$) δ -103.29, -103.30, -103.32, -103.32, -103.35. **[Acc. Mass]** calcd for [M+H] = $C_{29}H_{27}BF_2N$: 438.2199, found 438.2182.

Synthesis of 1-ethyl-4-mesityl-1,4-dihydrobenzo[b][1,4]azaborinine (4j)

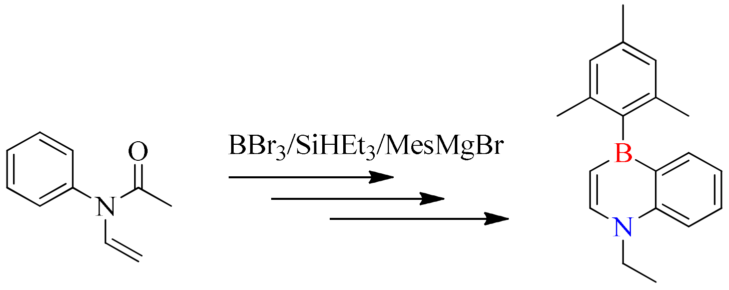

The synthesis was performed according to the **General Procedure C** using *N*-phenyl-*N*-vinylacetamide (0.3 g, 1.86 mmol), BBr_3 (0.9 mL, 9.30 mmol), $SiHEt_3$ (0.74 mL, 4.65 mmol), and MesMgBr (9.3 mL, 9.30 mmol, 1M in diethyl ether) in DCM (3 mL), and toluene (3 mL). Purification of the crude product was attempted by column chromatography (5-40 % dichloromethane in hexane); however, no fraction from these columns contained the desired product by NMR/Mass spec. analysis.

Synthesis of 12-mesityl-7-phenethyl-7,12-dihydrobenzo[b]naphtho[1,2-e][1,4]azaborinine (4k-α) and 12-mesityl-5-phenethyl-5,12-dihydrobenzo[b]naphtho[2,3-e][1,4]azaborinine (4k-β)

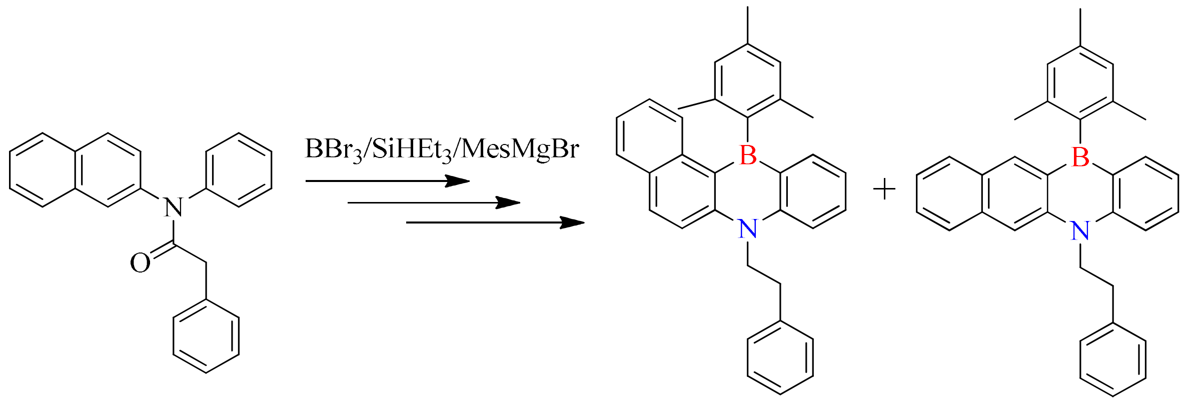

The synthesis was performed according to the **General Procedure C** using *N*-(naphthalen-2-yl)-*N*,2-diphenylacetamide (0.2 g, 0.59 mmol), BBr_3 (0.28 mL, 1.48 mmol), $SiHEt_3$ (0.23 mL, 1.48 mmol), and MesMgBr (2.9 mL, 2.91 mmol, 1M in diethyl ether) in DCM (2 mL), and toluene (2 mL). The crude product was purified by column chromatography (5-20 % dichloromethane in hexane) followed by washing with pentane to obtain the product as yellow solid. Yield: 0.05 g (18%, **4k-α**) and 0.03 g (13%, **4k-β**)

NMR data for **(4k-α)** ^{1}**H NMR** (500 MHz, $CDCl_3$) δ 8.42 (d, *J* = 8.7 Hz, 1H), 8.22 (d, *J* = 9.6 Hz, 1H), 8.04 (d, *J* = 9.5 Hz, 1H), 7.90 – 7.85 (m, 1H), 7.84-7.77 (m, 2H), 7.53 (d, *J* = 7 Hz, 2H), 7.48 (t, *J* = 7.5 Hz, 2H), 7.44-7.35 (m, 2H), 7.33-7.29 (m, 1H), 7.24-7.18 (m, 2H), 7.01

(s, 2H), 5.02 – 4.88 (m, 2H), 3.58-3.44 (m, 2H), 2.48 (s, 3H), 1.95 (s, 6H). **^{11}B NMR** (160 MHz, $CDCl_3$) δ 51.98. **^{13}C{^{1}H} NMR** (126 MHz, $CDCl_3$) δ 145.9, 143.9, 139.1, 138.3, 138.0, 137.4, 136.1, 135.4, 133.0, 129.2, 128.8, 128.2, 128.1, 127.8, 127.6, 127.6, 127.3, 124.3, 120.5, 116.2, 114.5, 50.3, 34.4, 23.0, 21.5. **[Acc. Mass]** calcd for [M+H] = $C_{33}H_{32}BN$: 452.2544, found 452.2542.

NMR data for **(4k-β)** **^{1}H NMR** (500 MHz, $CDCl_3$) δ 8.56 (s, 1H), 8.12 (s, 1H), 8.02-8.03 (d, J = 9.5 Hz, 1H), 7.95-7.96 (m, 1H), 7.87-7.91 (m, 2H), 7.77-7.85 (d, J = 7 Hz, 2H), 7.58-7.62 (t, J = 7.6 Hz, 2H), 7.50-7.53 (m, 2H), 7.39-7.42 (m, 1H), 7.16-7.19 (m, 2H), 7.05 (s, 2H), 5.02 – 4.88 (m, 2H), 3.58-3.44 (m, 2H), 2.48 (s, 3H), 1.95 (s, 6H). **^{11}B NMR** (160 MHz, $CDCl_3$) δ 55.89. **^{13}C{^{1}H} NMR** (126 MHz, $CDCl_3$) δ 146.5, 142.5, 139.8, 139.4, 138.7, 138.4, 136.9, 136.6, 134.6, 129.1, 129.0, 128.7, 128.0, 127.5, 127.3, 127.0, 126.9, 123.5, 119.3, 114.0, 109.8, 48.9, 33.0, 23.3, 21.4. **[Acc. Mass]** calcd for [M+H] = $C_{33}H_{32}BN$: 452.2544, found 452.2542.

Synthesis of 12-mesityl-7-neopentyl-7,12-dihydrobenzo[b]naphtho[1,2-e][1,4]azaborinine(4l-α) and 12-mesityl-5-neopentyl-5,12-dihydrobenzo[b]naphtho[2,3-e][1,4]azaborinine (4l-β)

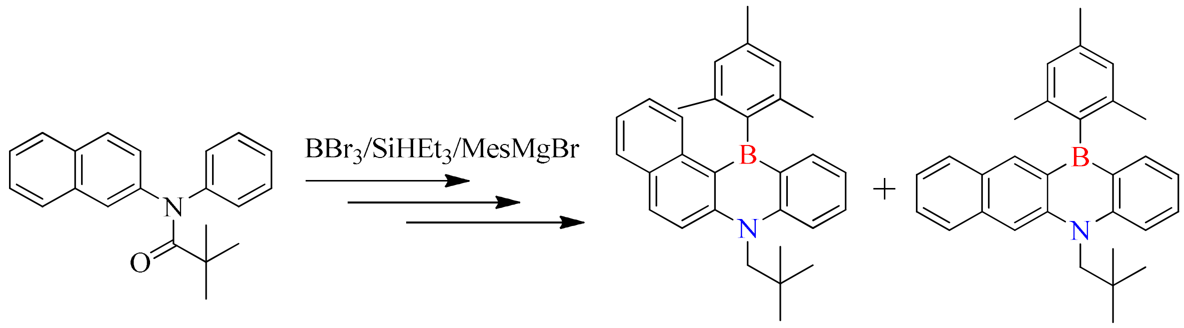

The synthesis was performed according to the **General Procedure C** using *N*-(naphthalen-2-yl)-*N*-phenylpivalamide (0.3 g, 0.98 mmol), BBr_3 (0.47 mL, 4.94 mmol), $SiHEt_3$ (0.39 mL, 2.47 mmol), and MesMgBr (4.9 mL, 4.92 mmol, 1M in diethyl ether) in DCM (3 mL), and toluene (3 mL). The crude product was purified by column chromatography (5-10 % dichloromethane in hexane) followed by washing with pentane to obtain the product as yellow solid. Yield: 0.12 g (30%, **4l-α**). Another product isomer (**4l-β**), was collected in negligble amount (<< 5%) which was not sufficient for its analysis.

^{1}H NMR (500 MHz, $CDCl_3$) δ 8.77-8.79 (dd, J = 7.5 and 2 Hz, 1H), 8.25-8.27 (d, J = 9.5 Hz, 1H), 8.19-8.21 (dd, J = 8 and 1.5 Hz, 1H), 8.11-8.14 (m, 2H), 8.01-8.02 (d, J = 9 Hz, 1H), 7.77-

7.80 (m, 1H), 7.60-7.63 (t, J = 7 Hz, 1H), 7.37-7.40 (t, J = 7.5 Hz, 1H), 7.01 (s, 2H), 4.85 (s, 2H), 2.44 (s, 3H), 2.33 (s, 3H), 2.31 (s, 3H), 1.05 (s, 9H). **^{11}B NMR** (160 MHz, $CDCl_3$) δ 50.56. **^{13}C{^{1}H} NMR** (126 MHz, $CDCl_3$) δ 149.0, 148.4, 140.1, 139.9, 139.9, 138.5, 137.6, 132.9, 132.5, 131.8, 131.3, 127.2, 127.1, 125.7, 124.2, 120.3, 117.8, 117.1, 53.5, 35.7, 29.8, 22.7, 22.6, 21.4. **[Acc. Mass]** calcd for [M+H] = $C_{28}H_{36}BN$: 418.2642, found 418.2649.

Synthesis of 13-mesityl-5-neopentyl-5,13-dihydrobenzo[b]benzo[4',5']thieno[3',2':5,6]benzo[1,2-e][1,4]azaborinine (4m-α) and 12-mesityl-7-neopentyl-7,12-dihydrobenzo[b]benzo[4',5']thieno[2',3':4,5]benzo[1,2-e][1,4]azaborinine (4m-β)

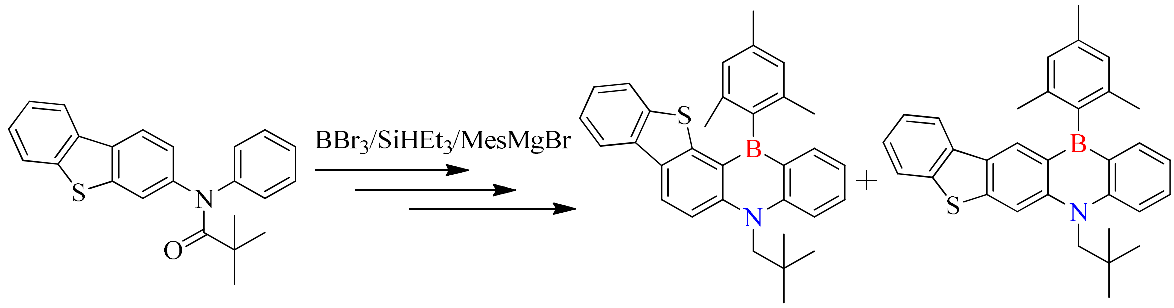

The synthesis was performed according to the **General Procedure C** using *N*-(dibenzo[b,d]thiophen-3-yl)-*N*-phenylpivalamide (0.1 g, 0.28 mmol), BBr_3 (0.13 mL, 1.39 mmol), $SiHEt_3$ (0.1 mL, 0.67 mmol), and MesMgBr (1.35 mL, 1.35 mmol, 1M in diethyl ether) in DCM (2 mL), and toluene (2 mL). The crude product was purified by column chromatography (5-15 % dichloromethane in hexane) followed by washing with pentane to obtain the product as off-white solid. Yield: 0.05 g (42%, **4m-α**) and 0.04 g (31%, **4m-β**).

4m-α analytical data: **^{1}H NMR** (500 MHz, $CDCl_3$) δ 8.41-8.43 (d, J = 9 Hz, 1H), 8.12-8.13 (d, J = 8 Hz, 1H), 8.06-8.08 (d, J = 9 Hz, 1H), 7.94-7.96 (d, J = 9 Hz, 1H), 7.86-7.88 (dd, J = 7.5 and 2 Hz, 1H), 7.71-7.76 (m, 2H), 7.41-7.45 (td, J = 7 and 1 Hz, 1H), 7.33-7.37 (td, J = 7 and 1 Hz, 1H), 7.33-7.37 (t, J = 7 Hz, 1H), 7.09 (s, 1H), 7.05 (s, 1H), 4.90 (s, 2H), 2.53 (s, 3H), 2.04 (s, 3H), 1.95 (s, 3H), 0.97 (s, 9H). **^{11}B NMR** (160 MHz, $CDCl_3$) δ 52.30. **^{13}C{^{1}H} NMR** (126 MHz, $CDCl_3$) δ 149.7, 147.1, 146.4, 139.4, 139.1, 137.4, 137.2, 134.8, 132.4, 127.6, 127.5, 127.4, 125.3, 125.0, 123.9, 122.6, 120.1, 120.0, 116.9, 115.3, 54.7, 35.9, 29.8, 22.7, 22.4, 22.3, 21.6, 14.1, **[Acc. Mass]** calcd for [M^+] = $C_{32}H_{32}BNS$: 474.2421, found 474.2417.

4m-β analytical data: **^{1}H NMR** (500 MHz, $CDCl_3$) δ 8.59 (s, 1H), 8.30 (s, 1H), 8.07-8.09 (m, 1H), 7.90-7.91 (d, J = 9 Hz, 1H), 7.86-7.88 (dd, J = 7.5 and 2 Hz, 1H), 7.81-7.82 (m, 1H), 7.71-7.74 (m, 1H), 7.39-7.43 (m, 2H), 7.12-7.15 (t, J = 7.5 Hz, 1H), 7.09 (s, 1H), 7.05 (s, 1H), 4.76-

4.84 (q, 2H), 2.49 (s, 3H), 2.07 (s, 3H), 1.98 (s, 3H), 1.02 (s, 9H). **^{11}B NMR** (160 MHz, $CDCl_3$) δ 54.13. **^{13}C{^{1}H} NMR** (126 MHz, $CDCl_3$) δ 147.1, 146.1, 145.4, 139.2, 138.4, 137.6, 136.4, 135.5, 132.8, 130.1, 128.8, 126.9, 126.9, 126.8, 126.3, 124.6, 122.5, 121.6, 119.6, 116.7, 109.4, 54.0, 35.7, 29.9, 23.2, 23.1, 21.4, 6.7, 4.4. **[Acc. Mass]** calcd for [M^+] = $C_{32}H_{32}BNS$: 474.2421, found 474.2413.

Synthesis of 1-(12-mesityl-5-phenyl-5H-benzo[5,6][1,4]azaborinino[2,3-b]carbazol-7(12H)-yl)-2,2-dimethylpropan-1-one (4n)

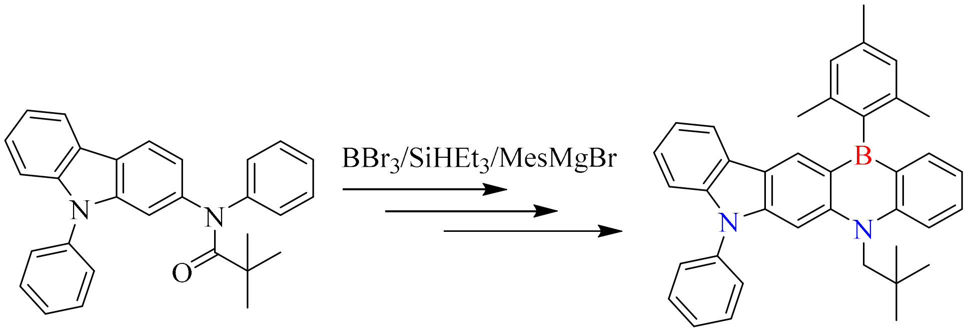

The synthesis was performed according to the **General Procedure C** using *N*-phenyl-*N*-(9-phenyl-9H-carbazol-2-yl)pivalamide (0.3 g, 0.71 mmol), BBr_3 (0.33 mL, 3.58 mmol), $SiHEt_3$ (0.3 mL, 1.79 mmol), and MesMgBr (3.6 mL, 3.58 mmol, 1M in diethyl ether) in DCM (3 mL), and toluene (3 mL). The crude product was purified by column chromatography (5-40 % dichloromethane in hexane) followed by washing with hexane to obtain the product as white solid. Yield: 0.16 g (43%).

^{1}H NMR (500 MHz, $CDCl_3$) δ 8.60 (s, 1H), 8.12-8.14 (d, J = 8 Hz, 1H), 7.82-7.85 (m, 2H), 7.65-7.73 (m, 6H), 7.57-7.60 (t, J = 7 Hz, 1H), 7.32-7.40 (m, 2H), 7.26-7.27 (m, 1H), 7.09-7.12 (t, J = 7 Hz, 1H), 7.06 (s, 1H), 7.04 (s, 1H), 4.68-4.71 (d, J = 16 Hz, 1H), 4.53-4.56 (d, J = 16 Hz, 1H), 2.50 (s, 3H), 2.10 (s, 3H), 2.03(s, 3H), 0.98 (s, 9H). **^{11}B NMR** (160 MHz, $CDCl_3$) δ 53.73. **^{13}C{^{1}H} NMR** (126 MHz, $CDCl_3$) δ 147.2, 146.7, 145.7, 142.5, 139.4, 137.5, 137.4, 136.1, 132.2, 130.1, 129.9, 128.0, 127.5, 126.9, 126.8, 125.7, 123.6, 120.6, 120.4, 119.1, 118.4, 116.3, 109.2, 95.4, 54.2, 35.2, 30.0, 23.3, 23.2, 21.4. **[Acc. Mass]** calcd for [M+H] = $C_{38}H_{38}BN_2$: 533.3122, found 533.3119.

Attempted borylations to form B_2N_2 pentacene analogues.

Problems occur when attempting to form fused 1,4-azaborine systems that share a benzene unit in doubly B, N-doped pentacene derivatives. The 1,4-dianiline derived substrate, **1o**, was borylated with BBr_3 (using a range of conditions), in this case analysis of the compound post-borylation was difficult due to poor solubility of the products, however multiple species are expected which should converge to a single 1,4-azaborine species upon reduction of the pivaloyl directing group.

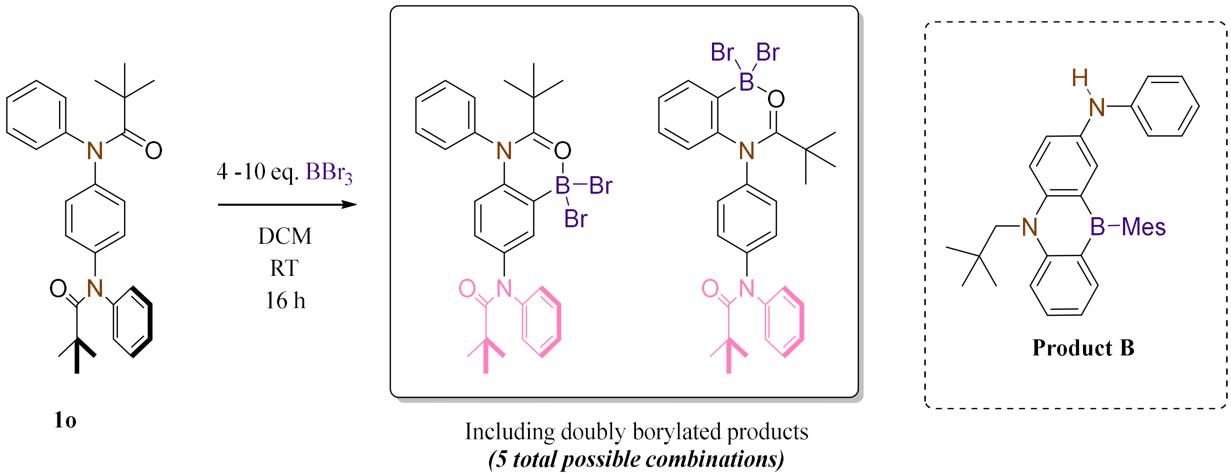

1o

Including doubly borylated products
(5 total possible combinations)

Figure **S5**. Plausible isomer structures and **byproduct B** after reacting amide 1o with BBr_3.

Addition of 5-10 equivalents $SiHEt_3$ (in separate reactions in attempts to form **4o**) to these reaction mixtures resulted in multiple species by ^{11}B NMR spectroscopy including large amounts of diborane (δ_{11B}=18.0 ppm) and the azaborine product as a minor peak at δ_{11B}=50.0 ppm. However, multiple attempts to purify the corresponding –BMes product, failed, with the product contaminated with a species exhibiting broad resonances in the ^{1}H NMR spectrum that could not be resolved **(Figure S6)**. Mass spectroscopic analysis of the impure product mixture only showed evidence of the mono-azaborine product, **B**, (m/z=459.2951, M+H) thus the side species can be assigned to that product resulting from incomplete borylation and complete cleavage of one of the neopentyl groups. However, these suggestions are only tentative as neither product could be obtained purely. Notably, the species that can be clearly resolved in the ^{1}H NMR spectrum, contains two mesityl groups as evidenced by three singlets attributed to 6 protons each in the aliphatic region, thus this product is likely to be the desired linear doubly B, N-doped azaborine, (Figure S6), additionally the corresponding ^{11}B NMR spectrum contained a single resonance at δ_{11B}=54.1 ppm consistent with an Ar_2BMes and thus precluding the existence of a $ArBMes_2$ species.

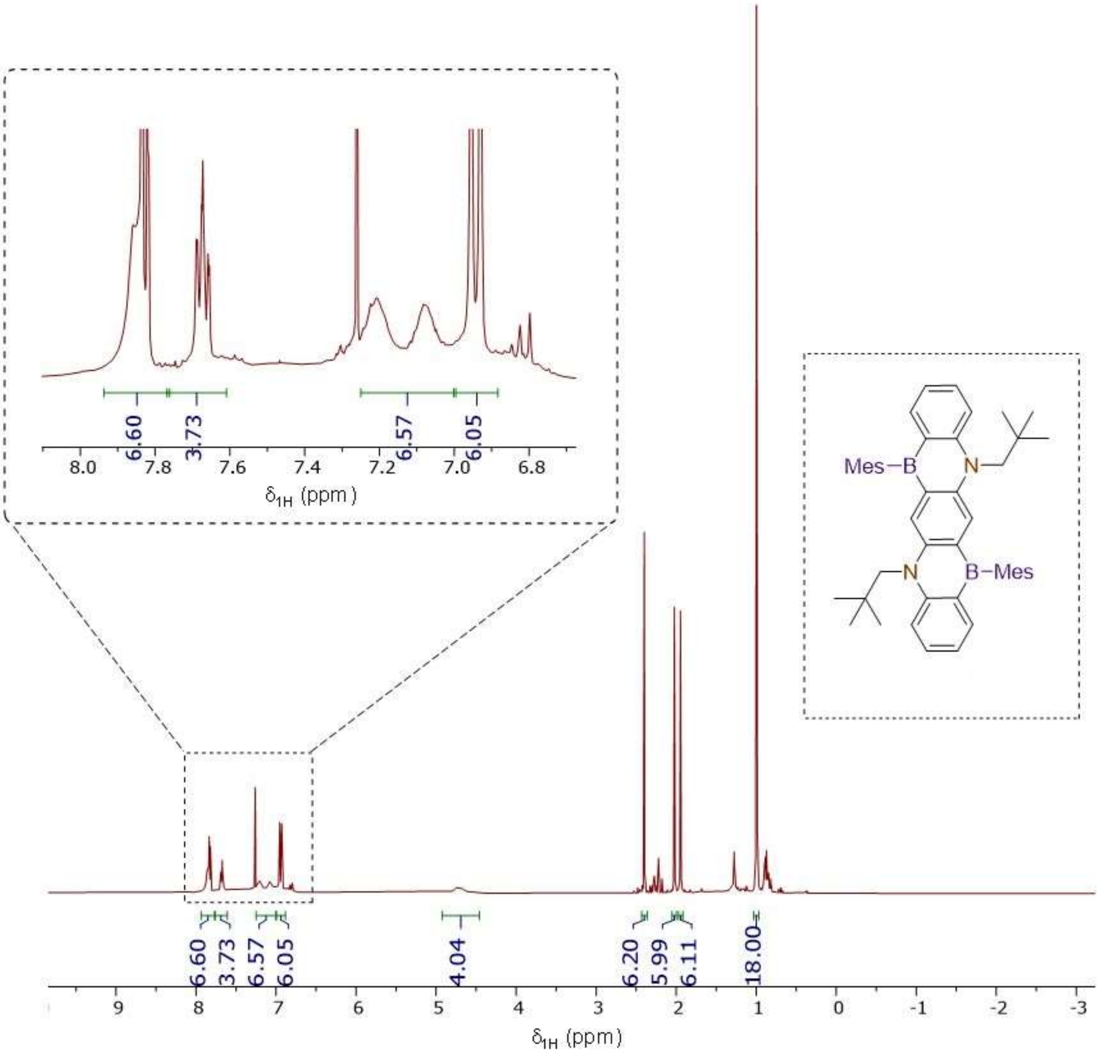

Figure **S6.** ^{1}H NMR spectrum of **4o**. Zoom in of aromatic region showing broad impurity resonances.

Attempted synthesis of (4o)

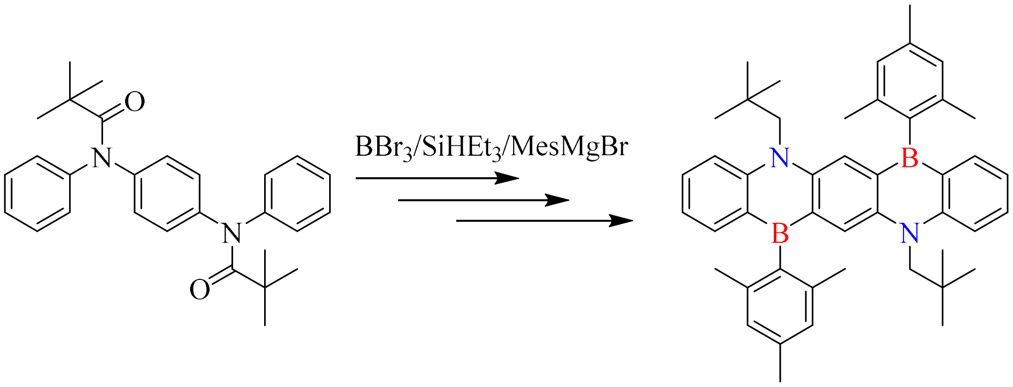

The synthesis was performed according to the **General Procedure C** using *N,N'*-(1,4-phenylene)bis(2,2-dimethyl-*N*-phenylpropanamide) (0.04 g, 0.09 mmol), BBr_3 (0.04 mL, 0.46

mmol), $SiHEt_3$ (0.04 mL, 0.23 mmol), and MesMgBr (0.46 mL, 0.46 mmol, 1M in diethyl ether) in DCM (1 mL), and toluene (1 mL). The crude product was purified by column chromatography (5-40 % dichloromethane in hexane). A small amount of desired product was formed, with its identity supported by mass spectrometry. **[Acc. Mass]** calcd for [M^+] = $C_{46}H_{54}B_2N_2$: 656.4538, found 656.4531.

Synthesis of 10,10'-dimesityl-5,5'-dineopentyl-5,5',10,10'-tetrahydro-2,2'-bidibenzo[b,e][1,4]azaborinine (4p)

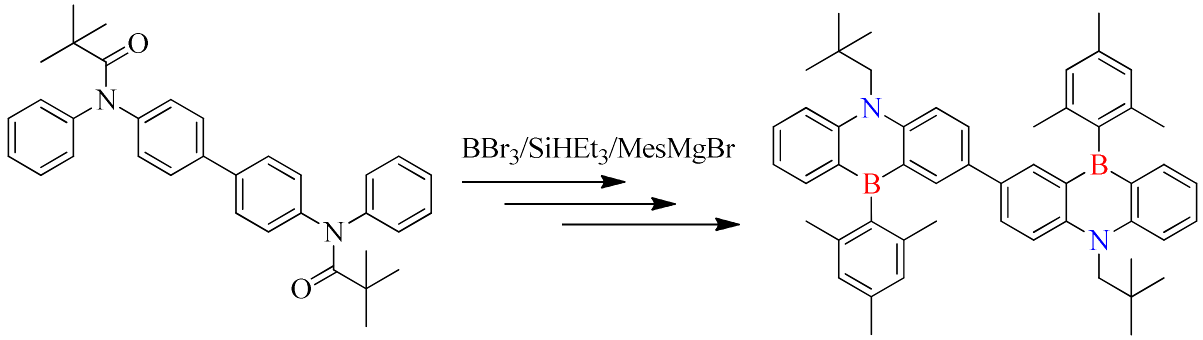

The synthesis was performed according to the **General Procedure C** using *N,N'*-([1,1'-biphenyl]-4,4'-diyl)bis(2,2-dimethyl-*N*-phenylpropanamide) (0.1 g, 0.19 mmol), BBr_3 (0.09 mL, 0.19 mmol), $SiHEt_3$ (0.08 mL, 0.49 mmol), and MesMgBr (0.96 mL, 0.96 mmol, 1M in diethyl ether) in DCM (2 mL), and toluene (2 mL). The crude product was purified by column chromatography (5-30 % dichloromethane in hexane) followed by washing with hexane to obtain pure product as yellow solid. Yield: 0.036 g (25%).

^{1}H NMR (500 MHz, $CDCl_3$) δ 8.02-8.03 (*J* = 5 Hz, 2H), 7.88-7.93 (m, 8H), 7.69-7.72 (td, *J* = 7 and 2 Hz, 2H), 7.11-7.14 (t, *J* = 7 Hz, 2H), 7.03 (s, 1H), 6.99 (s, 2H), 6.96 (s, 1H), 4.75 (s, 4H), 2.48 (s, 6H), 2.01 (s, 3H), 1.99 (s, 3H), 1.95 (s, 3H), 1.93 (s, 3H), 1.03 (s, 18H). **^{11}B NMR** (160 MHz, $CDCl_3$) δ 53.77. **^{13}C{^{1}H} NMR** (126 MHz, $CDCl_3$) δ 146.8, 145.9, 140.2, 139.2, 139.1, 137.6, 137.5, 135.9, 135.1, 135.0, 132.4, 131.6, 131.5, 131.4, 131.3, 127.7, 127.5, 126.9, 126.8, 119.4, 117.2, 117.1, 116.7, 53.6, 35.6, 29.9, 23.1, 23.0, 23.0, 21.4. **[Acc. Mass]** calcd for [M^+] = $C_{52}H_{58}B_2N_2$: 732.4797, found 732.4804.

Synthesis of 2-(3,6-di-tert-butyl-9H-carbazol-9-yl)-10-mesityl-5-phenethyl-5,10-dihydrodibenzo[b,e][1,4]azaborinine (5)

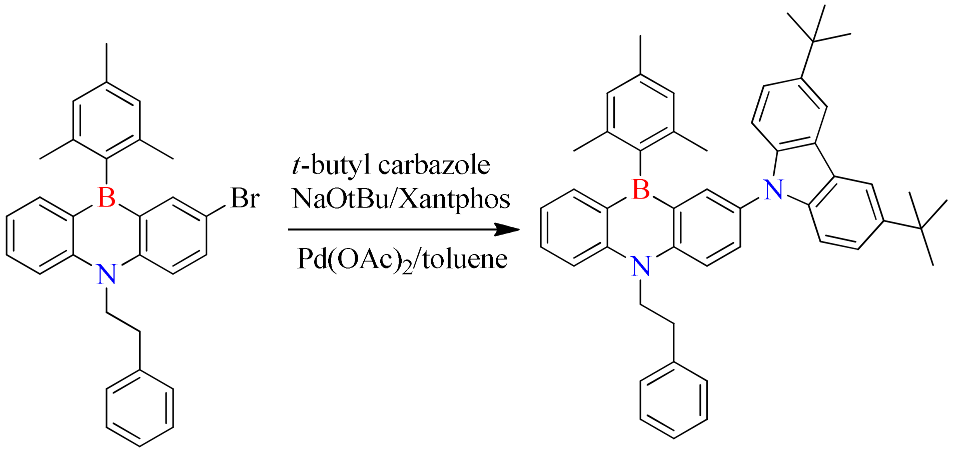

To a 100 mL round bottom flask, a mixture of 2-bromo-10-mesityl-5-phenethyl-5,10-dihydrodibenzo[b,e][1,4]azaborinine (**4g**) (0.2 g, 0.41 mmol), di-*t*-butyl carbazole (0.14 g, 0.49 mmol), NaO*t*Bu (0.08 g, 0.83 mmol), Pd(OAc)$_2$ (0.03 g, 0.13 mmol), Xant-Phos (0.04 g, 0.09 mmol) was dissolved in anhydrous toluene (10 mL) and was refluxed for 15 hours. The reaction was then quenched with water and extracted with ethyl acetate (3 x 100 mL). The organic layer was separated and dried over Na_2SO_4. The crude product was then purified by column chromatography (10-30% DCM in hexane) to obtain the product as yellow solid. Yield: 0.14 g (50%).

^{1}H NMR (500 MHz, $CDCl_3$) δ 8.15-8.16 (d, *J* = 1.5 Hz, 2H), 8.06-8.07 (d, *J* = 2.5 Hz, 1H), 7.88-7.99 (m, 5H), 7.37-7.53 (m, 7H), 7.24-7.27 (m, 3H), 6.90 (s, 2H), 4.90-4.93 (t, *J* = 8.5 Hz, 2H), 3.47-3.51 (t, *J* = 8.5 Hz, 2H), 2.33 (s, 3H), 2.05 (s, 6H), 1.49 (s, 18H). **^{11}B NMR** (160 MHz, $CDCl_3$) δ 53.95. **^{13}C{^{1}H} NMR** (126 MHz, $CDCl_3$) δ 145.2, 144.1, 142.5, 139.9, 138.9, 138.1, 136.5, 135.5, 134.0, 132.6, 129.7, 129.0, 128.7, 127.1, 126.9, 126.8, 123.5, 123.1, 120.1, 116.2, 116.1, 114.5, 108.9, 49.0, 34.7, 33.6, 32.0, 23.3, 21.2. **[Acc. Mass]** calcd for [M^+] = $C_{49}H_{51}B_1N_2$: 679.4218, found 679.4208.

Synthesis of 12-mesityl-7-neopentyl-7,12-dihydrobenzo[b]benzo[4',5']thieno[2',3':4,5]benzo[1,2-e][1,4]azaborinine 5,5-dioxide (6)

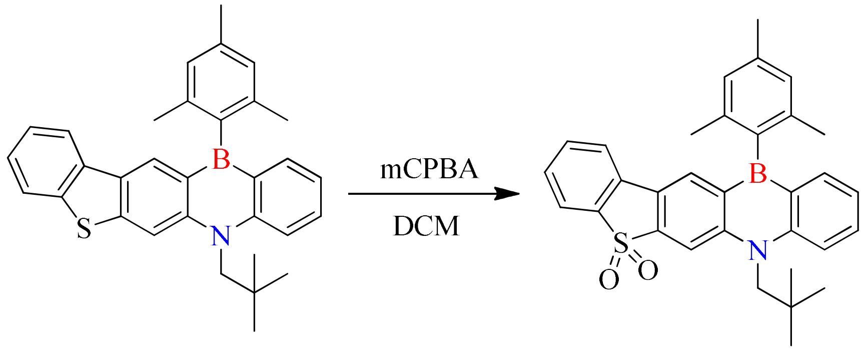

To a 50 mL round bottom flask, 12-mesityl-7-neopentyl-7,12-dihydrobenzo[b]benzo[4',5']thieno[2',3':4,5]benzo[1,2-e][1,4]azaborinine (**4m-β**) (0.025 g, 0.05 mmol), was dissolved in dichloromethane (10 mL) and the mixture was cooled at 0 °C. mCPBA (0.03 g, 0.16 mmol) was added and the reaction mixture was stirred at room temperature for 2 hrs. The reaction was then quenched with water and extracted with dichloromethane (3 x 30 mL). The organic layer was separated and dried over Na_2SO_4. The crude product was then purified by column chromatography (30-60% DCM in hexane) to obtain the product as yellow solid. Yield: 0.017 g (65%).

^{1}H NMR (500 MHz, $CDCl_3$) δ 8.38 (s, 1H), 8.22 (s, 1H), 7.94-7.96 (d, J = 9 Hz, 1H), 7.87-7.89 (dd, J = 7.5 and 2 Hz, 1H), 7.84-7.85 (d, J = 7.5 Hz, 1H), 7.75-7.80 (m, 2H), 7.57-7.60 (t, J = 7.5 Hz, 1H), 7.44-7.47 (t, J = 7.5 Hz, 1H), 7.20-7.23 (t, J = 7.5 Hz, 1H), 7.03 (s, 1H), 7.00 (s, 1H), 4.75-4.89 (m, 2H), 2.47 (s, 3H), 2.02 (s, 3H), 1.94 (s, 3H), 1.01 (s, 9H). **^{11}B NMR** (160 MHz, $CDCl_3$) δ 54.10. **^{13}C{^{1}H} NMR** (126 MHz, $CDCl_3$) δ 147.7, 146.9, 141.7, 139.1, 137.7, 137.4, 136.9, 133.9, 133.6, 132.5, 130.4, 128.9, 127.2, 127.1, 122.1, 121.6, 121.2, 121.0, 117.2, 111.3, 54.4, 35.9, 29.7, 23.2, 23.0, 21.4. **[Acc. Mass]** calcd for [M^+] = $C_{32}H_{32}BNSO_2$: 506.2319, found 506.2319.

S3. NMR Spectra

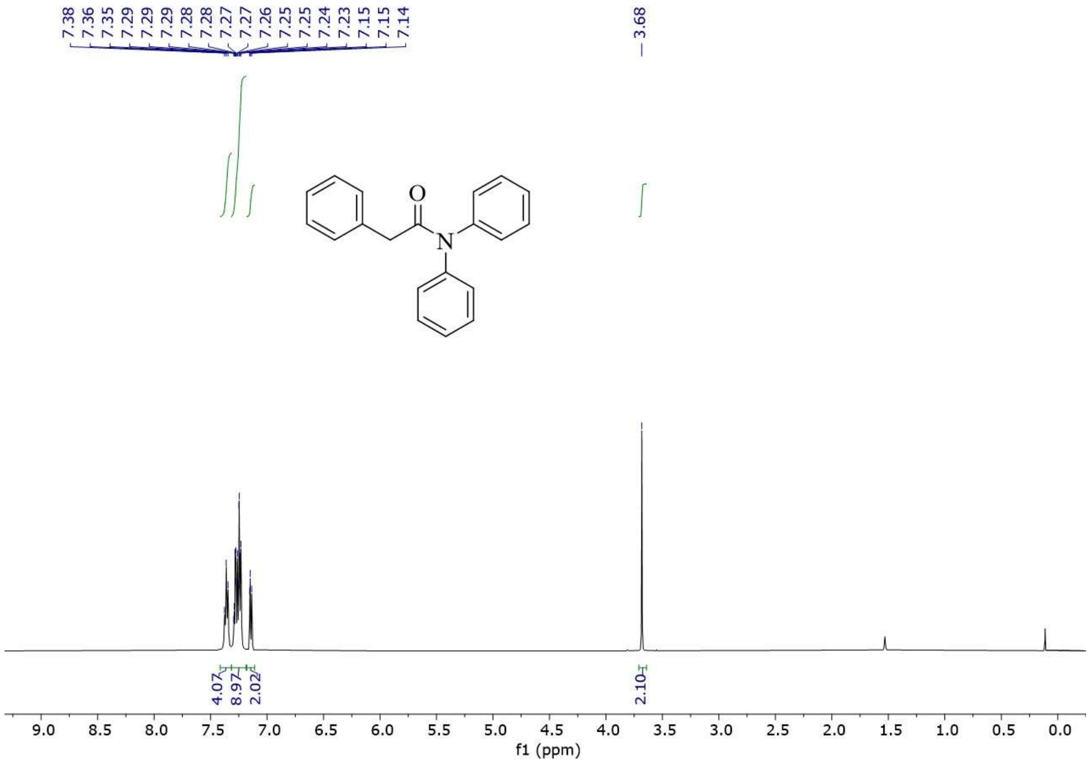

Figure **S7** ^{1}H NMR spectrum of ***N,N*-2-triphenylacetanamide, 1a** in $CDCl_3$ (500 MHz) at 50 °C.

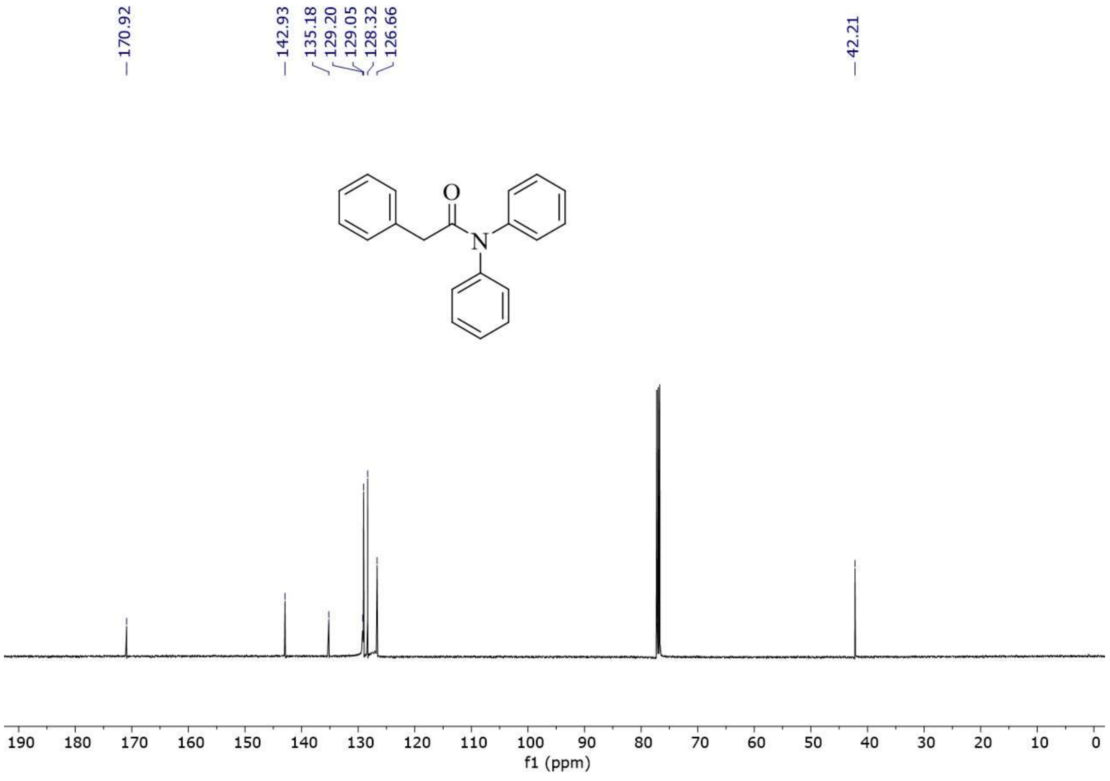

Figure **S8**. ^{13}C{^{1}H} NMR spectrum of ***N,N*-2-triphenylacetanamide, 1a** in $CDCl_3$ (126 MHz) at 50 °C.

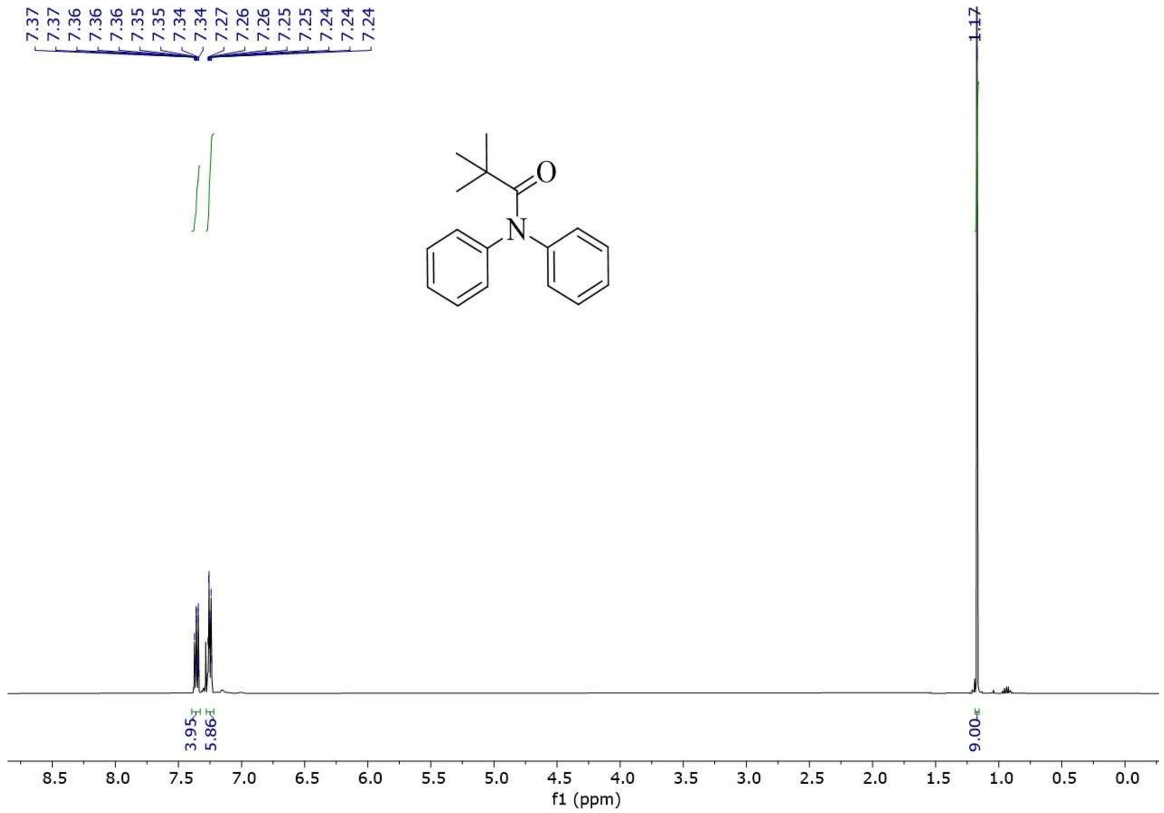

Figure **S9**. ¹H NMR spectrum of ***N,N*-diphenylpivalamide,1b** in $CDCl_3$ (500 MHz) at 50 °C.

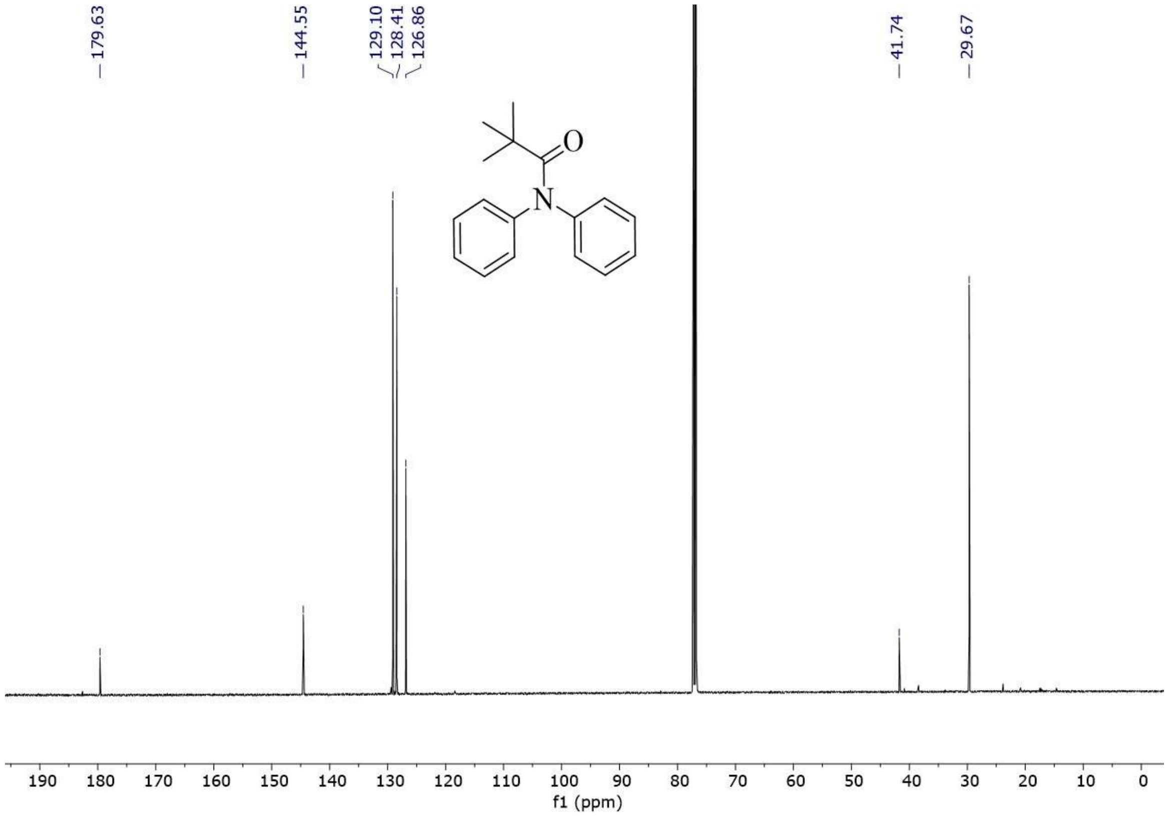

Figure **S10**. $^{13}C\{^1H\}$ NMR spectrum of ***N,N*-diphenylpivalamide, 1b** in $CDCl_3$ (126 MHz) at 50 °C

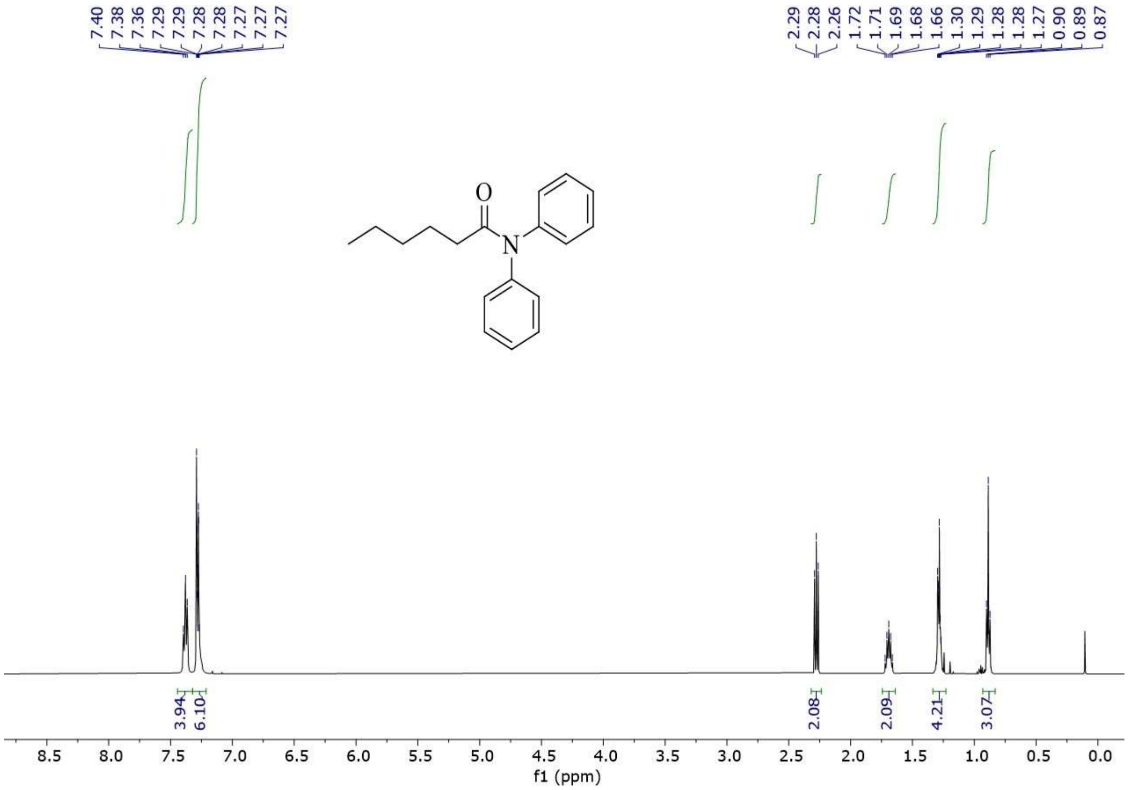

Figure **S11**. ^{1}H NMR spectrum of ***N,N*-diphenylhexanamide, 1c** in $CDCl_3$ (500 MHz) at 50 °C.

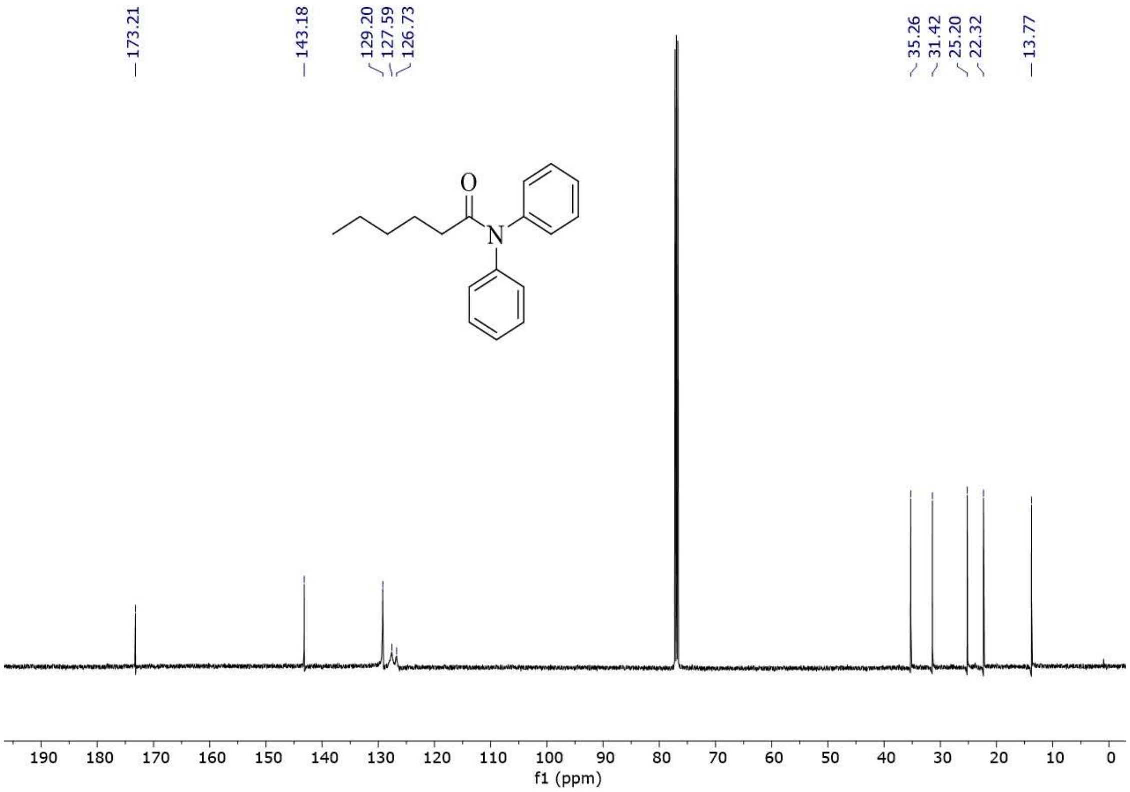

Figure **S12**. $^{13}C\{^{1}H\}$ NMR spectrum of ***N,N*-diphenylhexanamide, 1c** in $CDCl_3$ (126 MHz) at 50 °C.

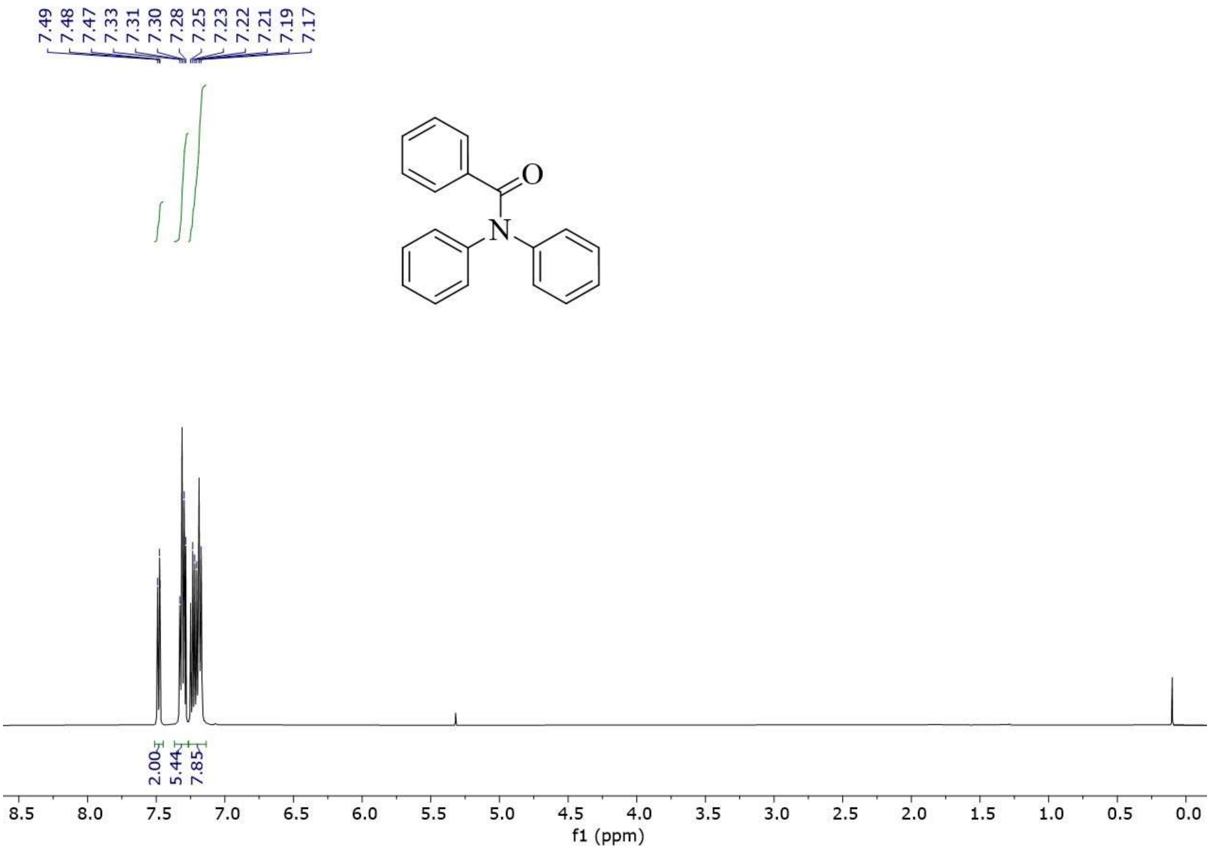

Figure **S13**. ^{1}H NMR spectrum of ***N,N*-diphenylbenzamide, 1d** in $CDCl_3$ (500 MHz).

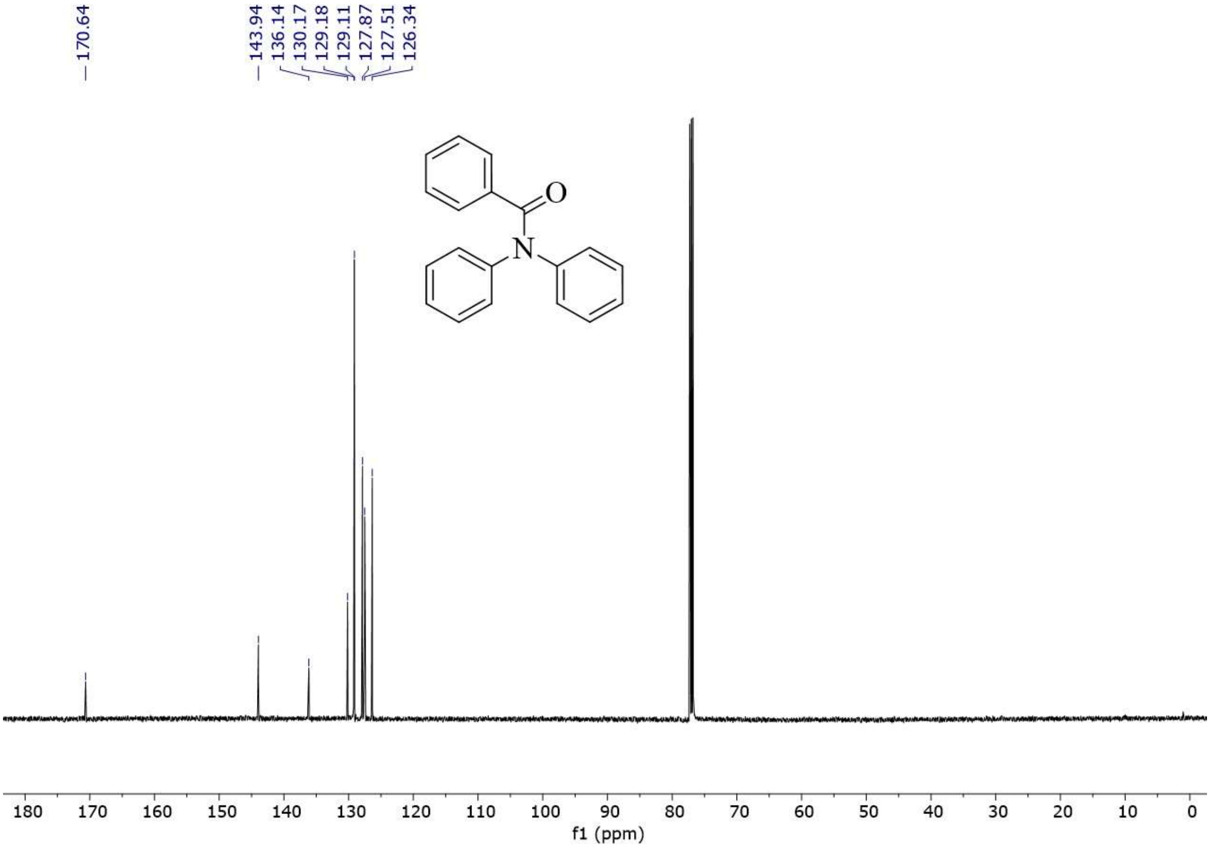

Figure **S14**. $^{13}C\{^1H\}$ NMR spectrum of ***N,N*-diphenylbenzamide, 1d** in $CDCl_3$ (126 MHz).

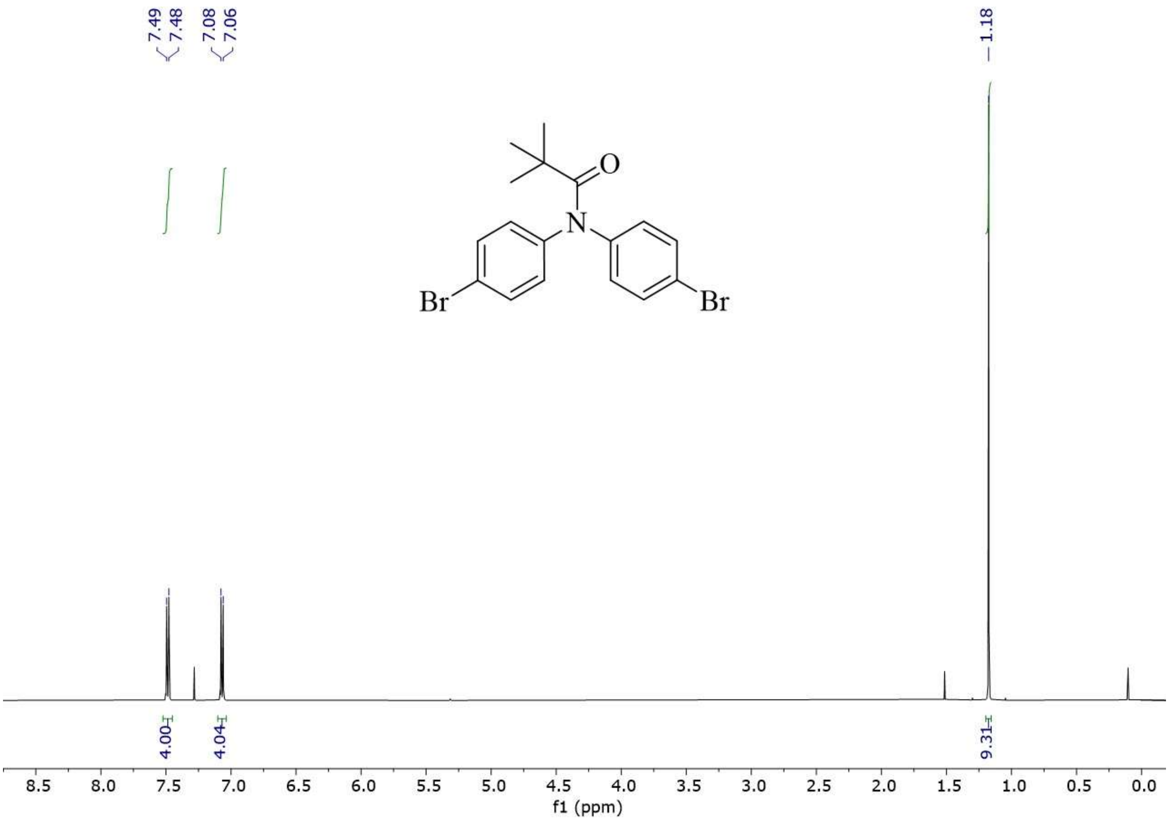

Figure **S15**. ¹H NMR spectrum of ***N,N*-bis(4-bromophenyl)pivalamide, 1e** in $CDCl_3$ (500 MHz).

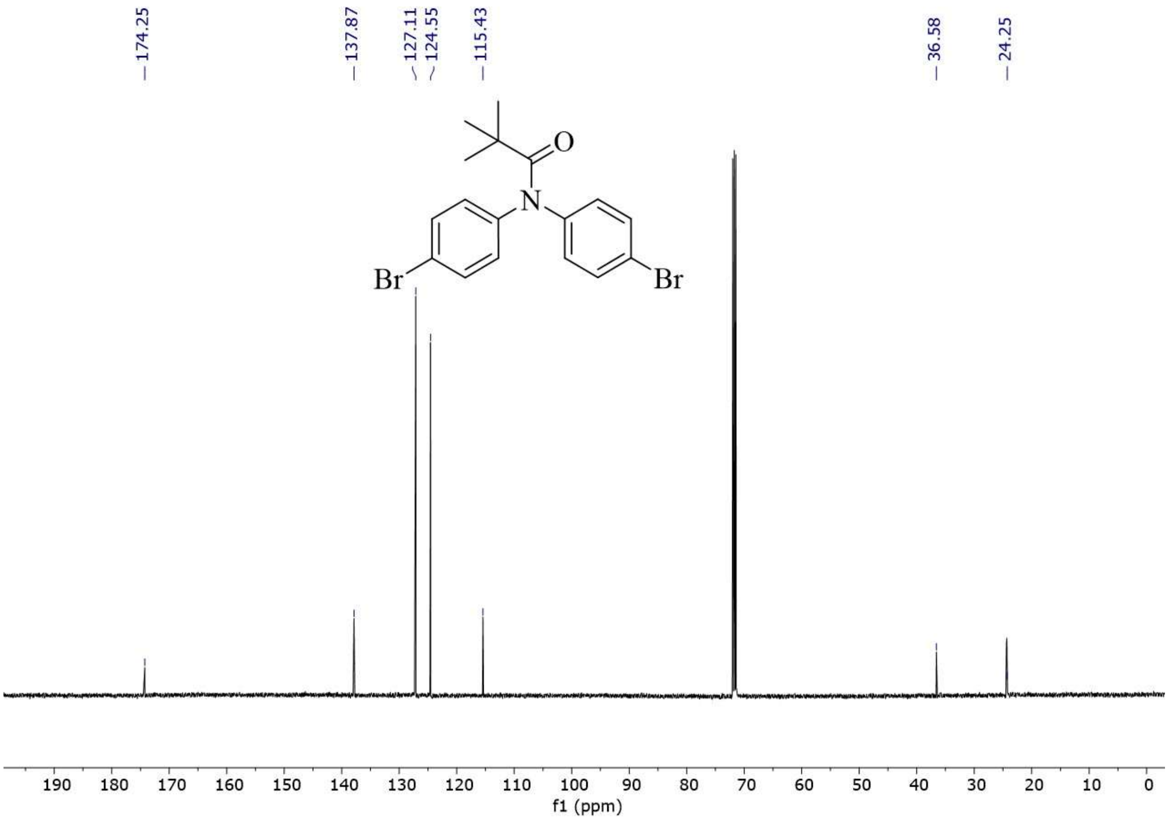

Figure **S16**. $^{13}C\{^1H\}$ NMR spectrum of ***N,N*-bis(4-bromophenyl)pivalamide, 1e** in $CDCl_3$ (126 MHz).

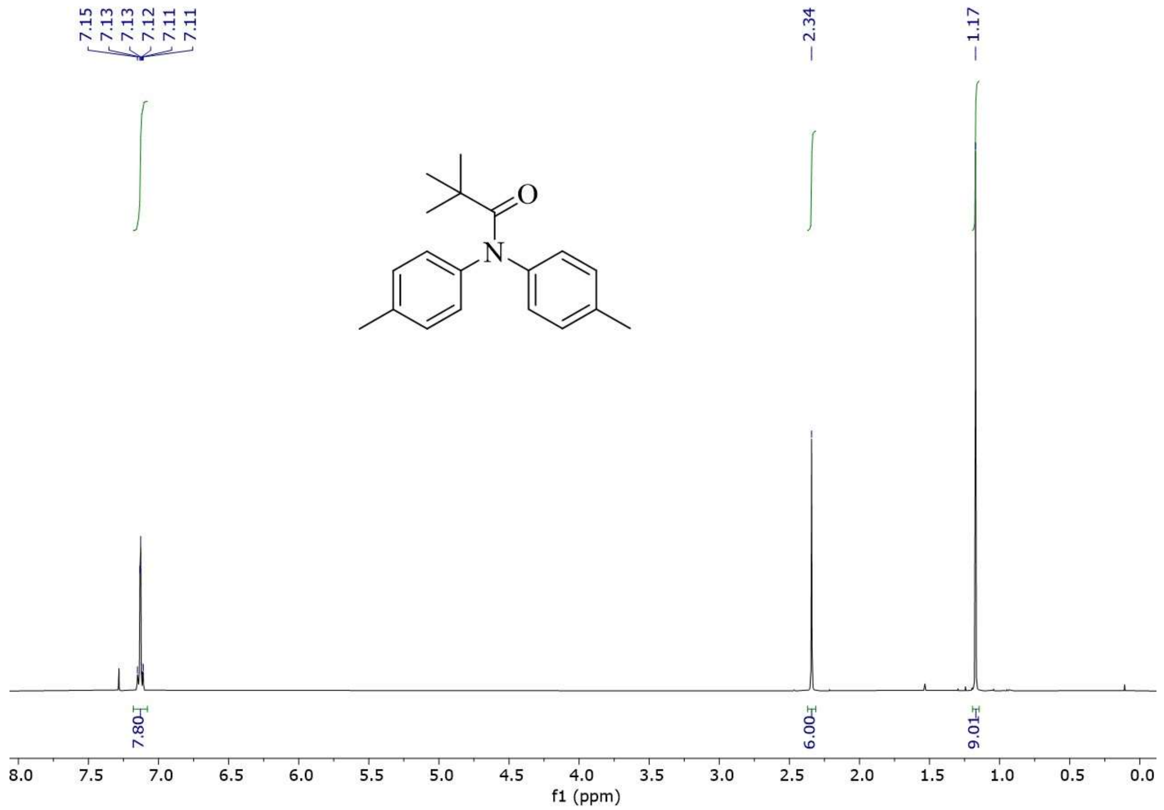

Figure **S17**. ^{1}H NMR spectrum of ***N*,*N*-bis(4-methylphenyl)pivalamide, 1f** in $CDCl_3$ (500 MHz) at 50 °C.

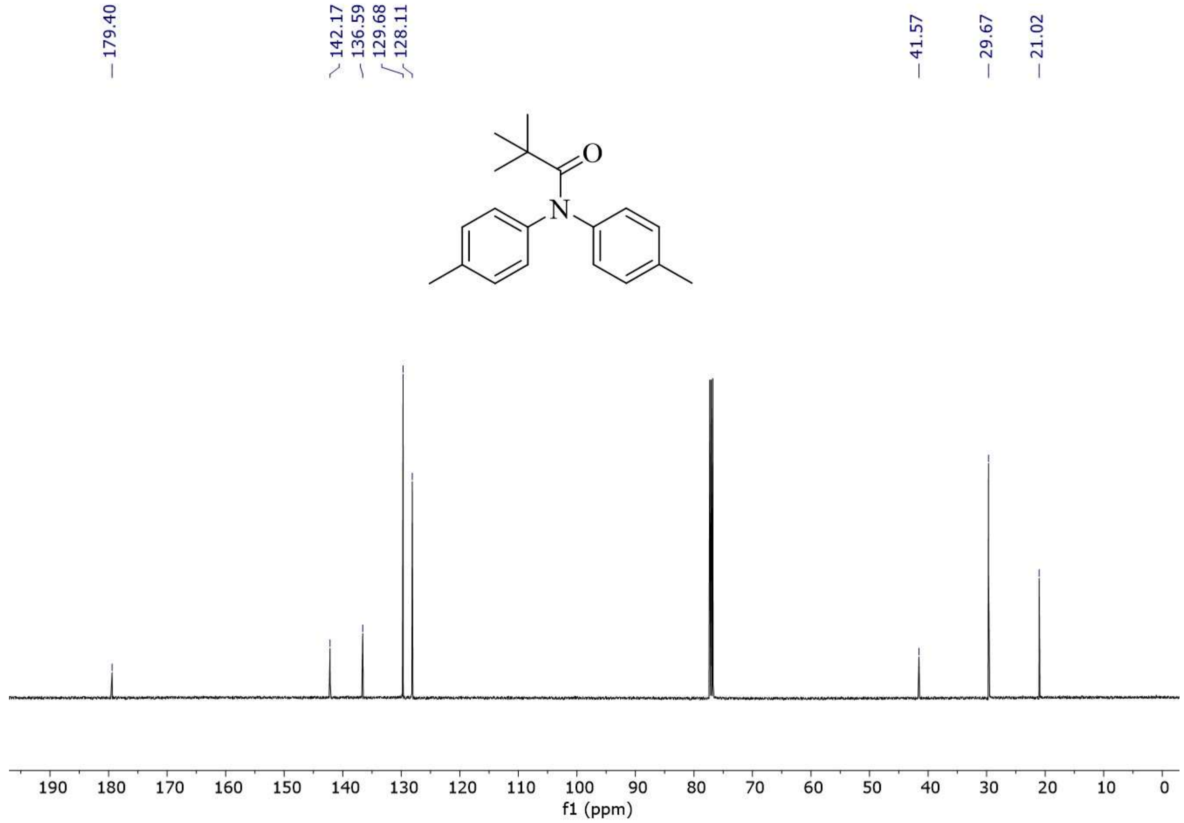

Figure **S18**. $^{13}C\{^1H\}$ NMR spectrum of ***N*,*N*-bis(4-methylphenyl)pivalamide, 1f** in $CDCl_3$ (126 MHz) at 50 °C.

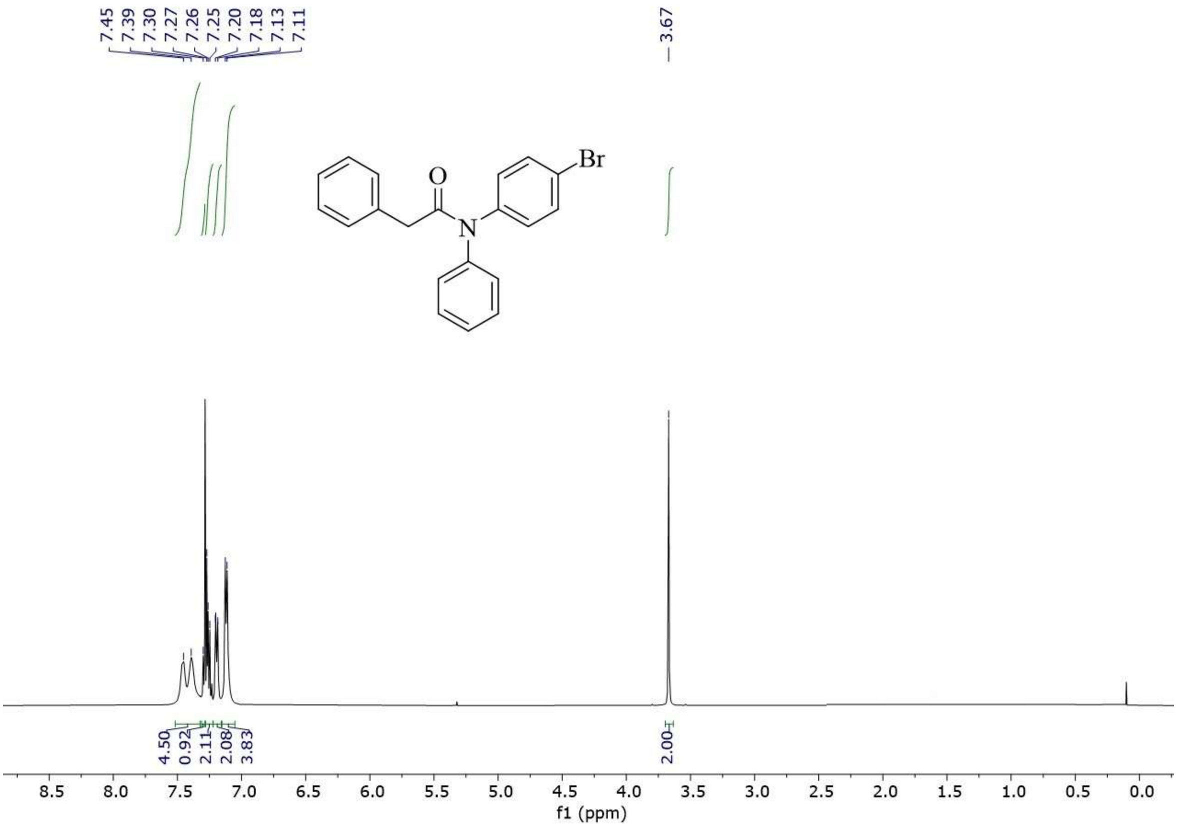

Figure **S19**. ^{1}H NMR spectrum of ***N*-(4-bromophenyl)-N,2-diphenylacetamide, 1g** in $CDCl_3$ (500 MHz) at 50 °C.

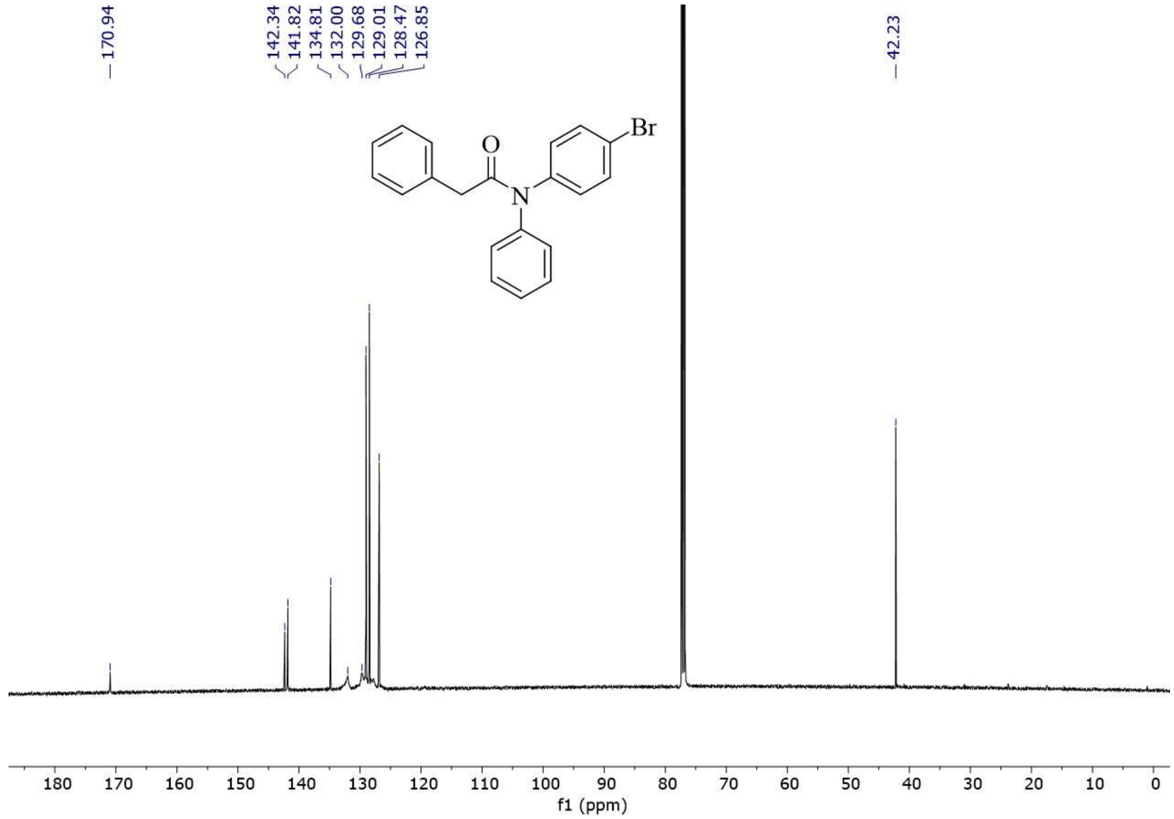

Figure **S20**. $^{13}C\{^{1}H\}$ NMR spectrum of ***N*-(4-bromophenyl)-N,2-diphenylacetamide, 1g** in $CDCl_3$ (126 MHz) at 50 °C.

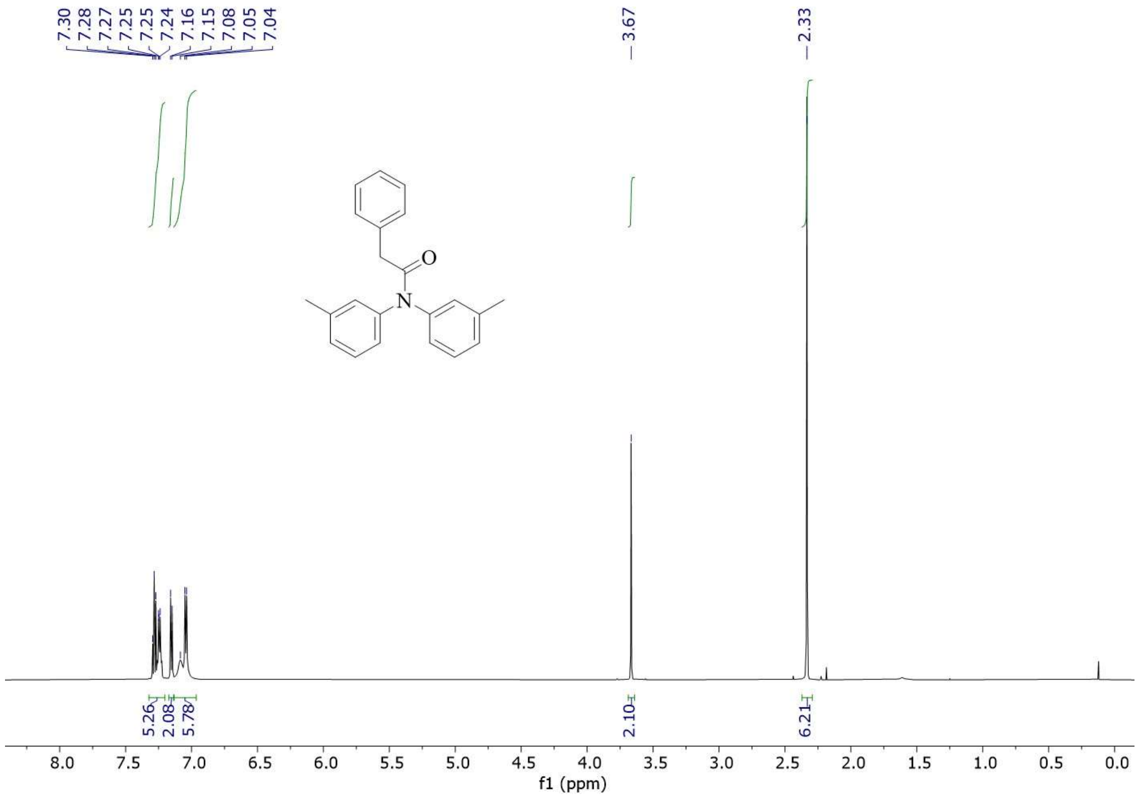

Figure **S21**. ^{1}H NMR spectrum of **2-phenyl-*N,N*-di-m-tolylacetamide, 1h** in $CDCl_3$ (500 MHz) at 50 °C.

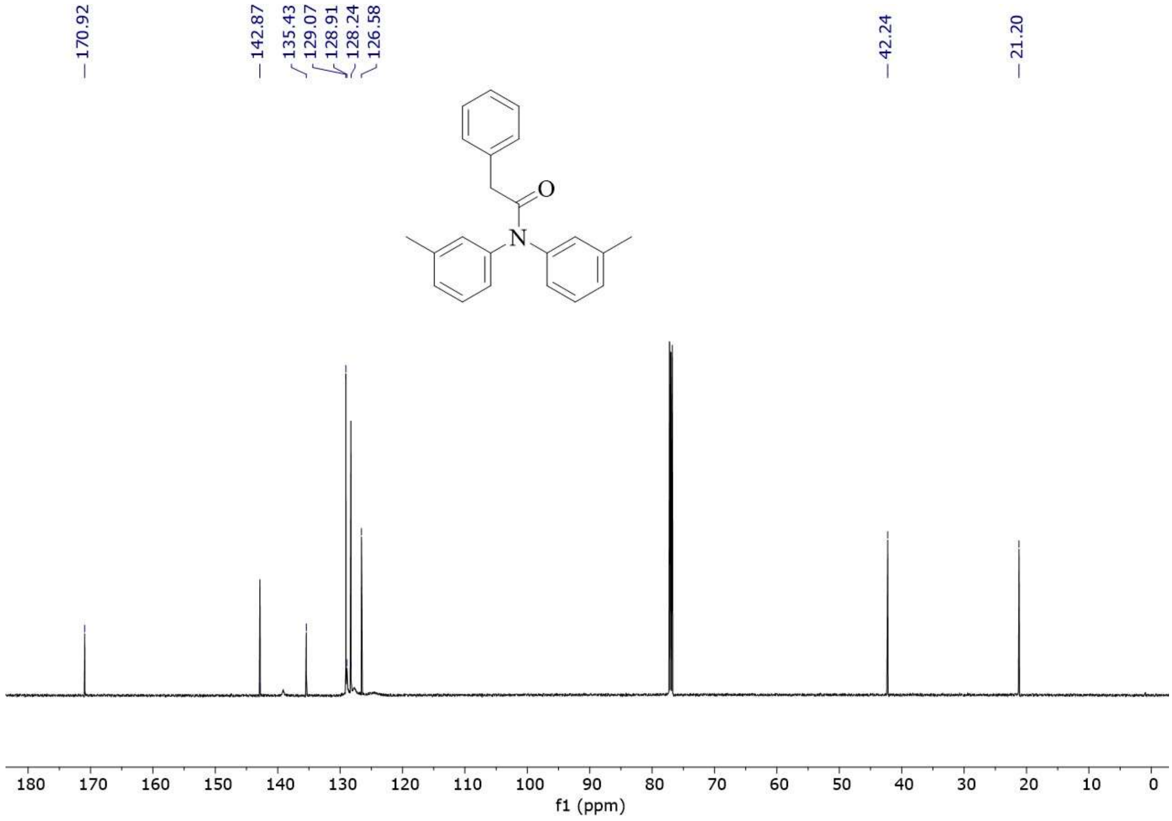

Figure **S22**. $^{13}C\{^{1}H\}$ NMR spectrum of **2-phenyl-*N,N*-di-m-tolylacetamide, 1h** in $CDCl_3$ (126 MHz) at 50 °C.

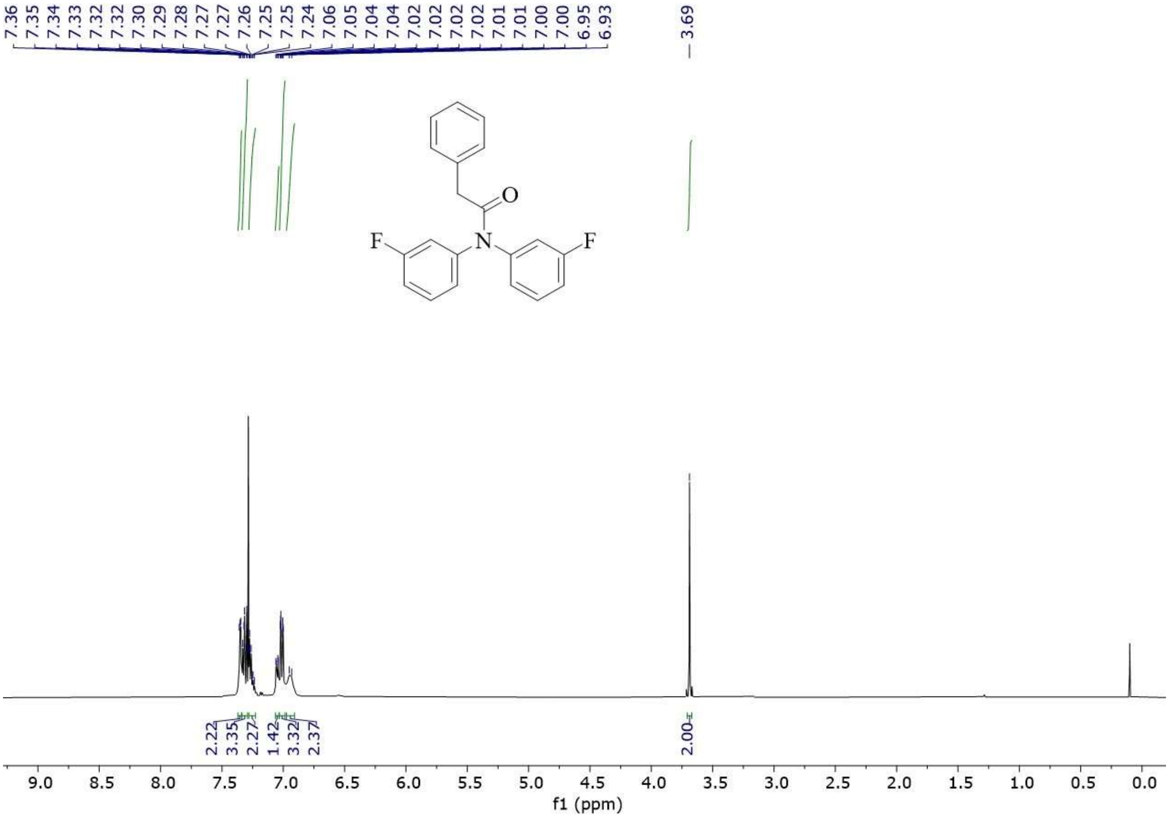

Figure **S23**. ^{1}H NMR spectrum of ***N,N*-bis(3-fluorophenyl)-2-phenylacetamide, 1i** in $CDCl_3$ (500 MHz) at 50 °C.

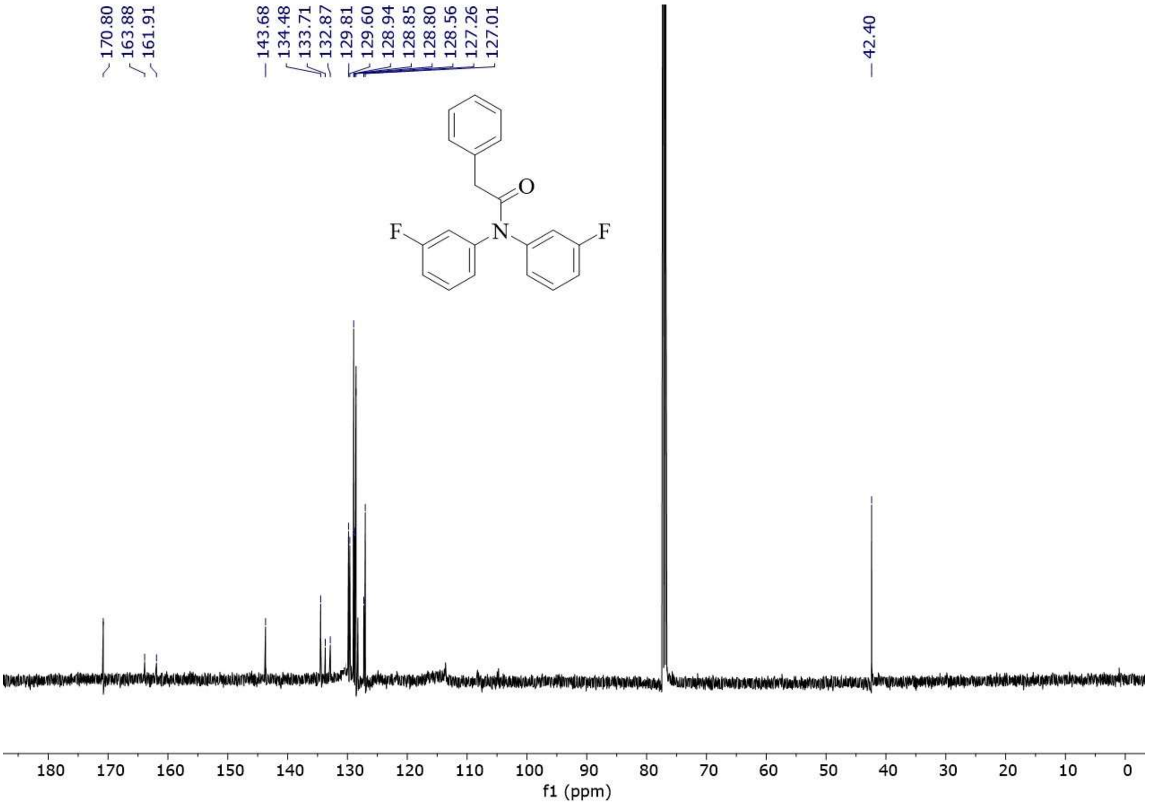

Figure **S24**. $^{13}C\{^1H\}$ NMR spectrum of ***N,N*-bis(3-fluorophenyl)-2-phenylacetamide, 1i** in $CDCl_3$ (126 MHz) at 50 °C.

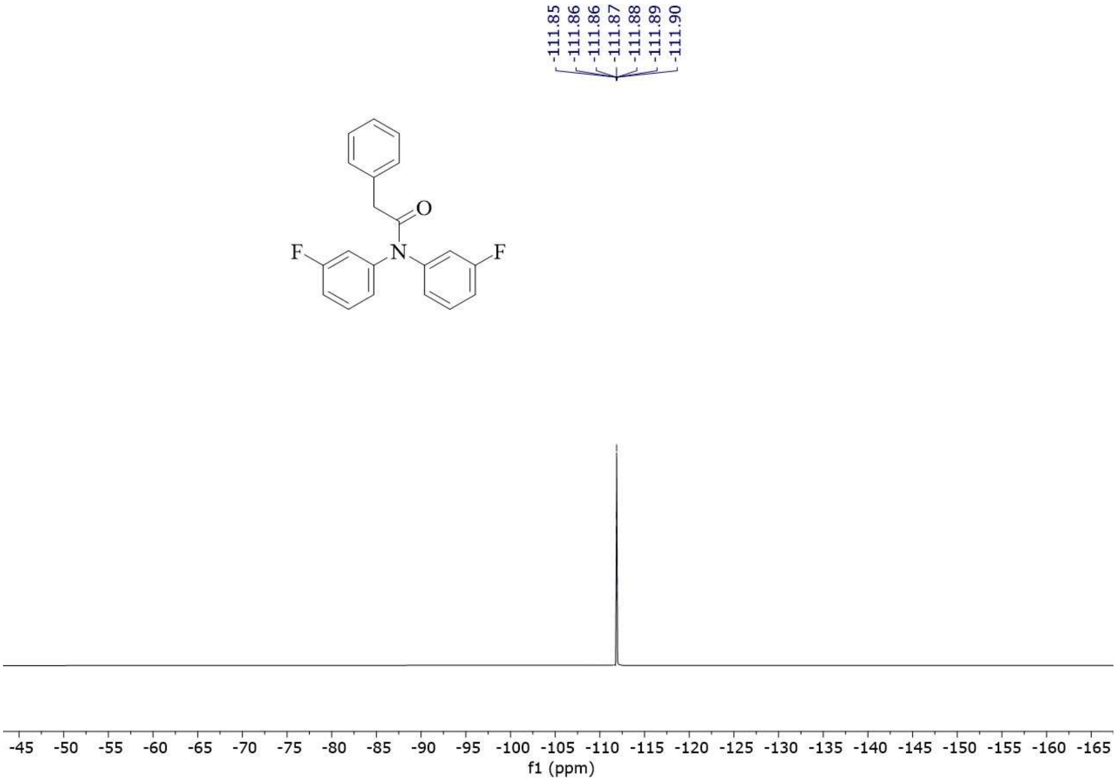

Figure **S25**. ^{19}F NMR spectrum of ***N,N*-bis(3-fluorophenyl)-2-phenylacetamide, 1i** in $CDCl_3$ (471 MHz).

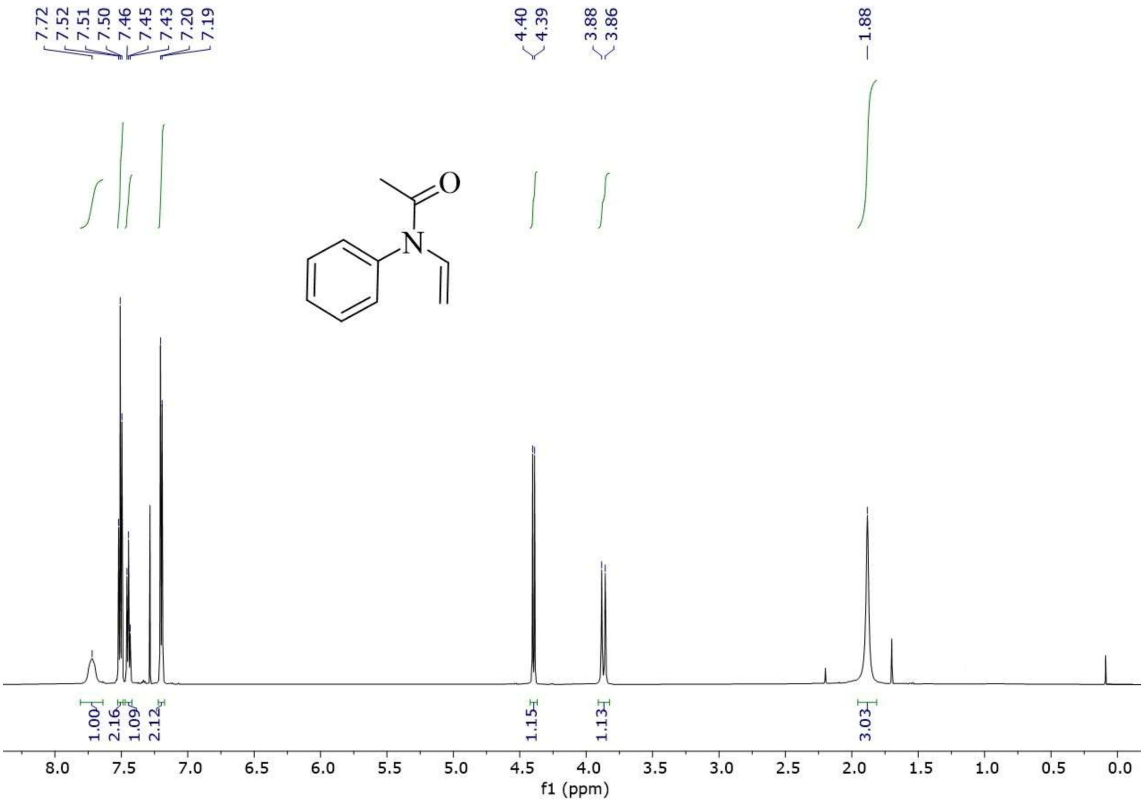

Figure **S26**. ^{1}H NMR spectrum of ***N*-phenyl-*N*-vinylacetamide, 1j** in $CDCl_3$ (500 MHz).

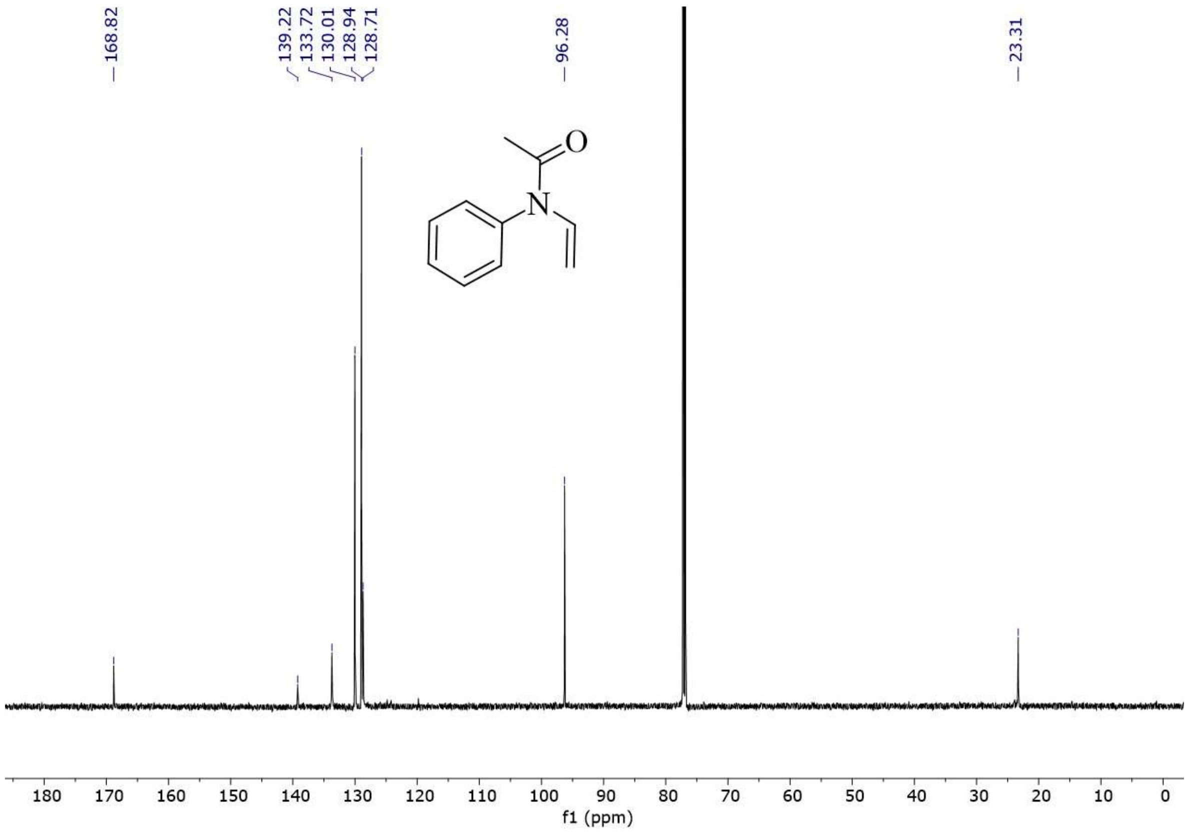

Figure **S27**. $^{13}C\{^{1}H\}$ NMR spectrum of ***N*-phenyl-*N*-vinylacetamide, 1j** in $CDCl_3$ (126 MHz).

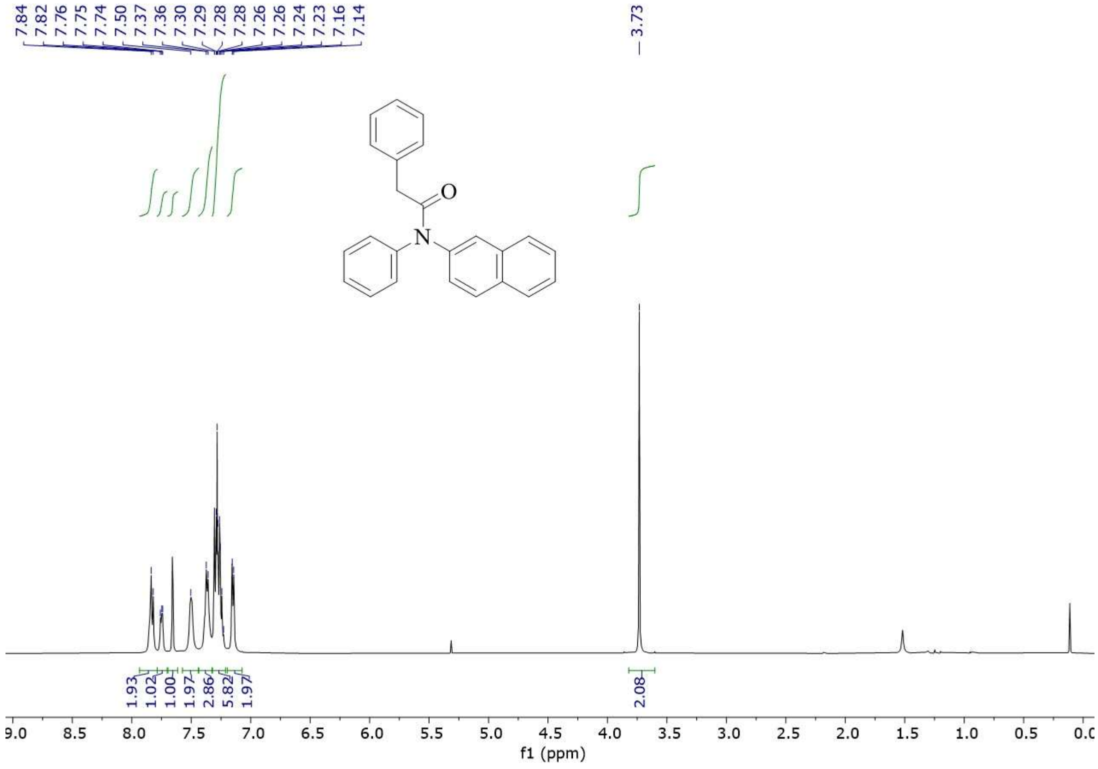

Figure **S28**. ^{1}H NMR spectrum of ***N*-(naphthalen-2-yl)-*N*,2-diphenylacetamide, 1k** in $CDCl_3$ (500 MHz) at 50 ºC

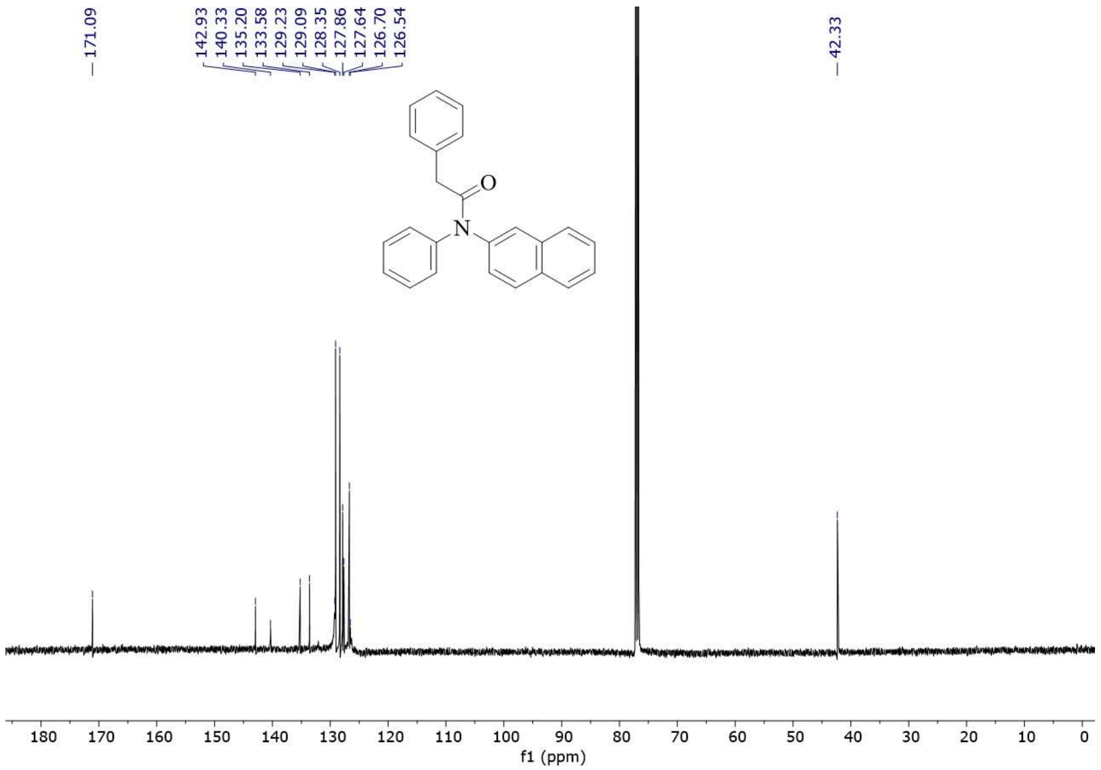

Figure **S29**. $^{13}C\{^{1}H\}$ NMR spectrum of ***N*-(naphthalen-2-yl)-*N*,2-diphenylacetamide, 1k** in $CDCl_3$ (126 MHz) at 50 ºC.

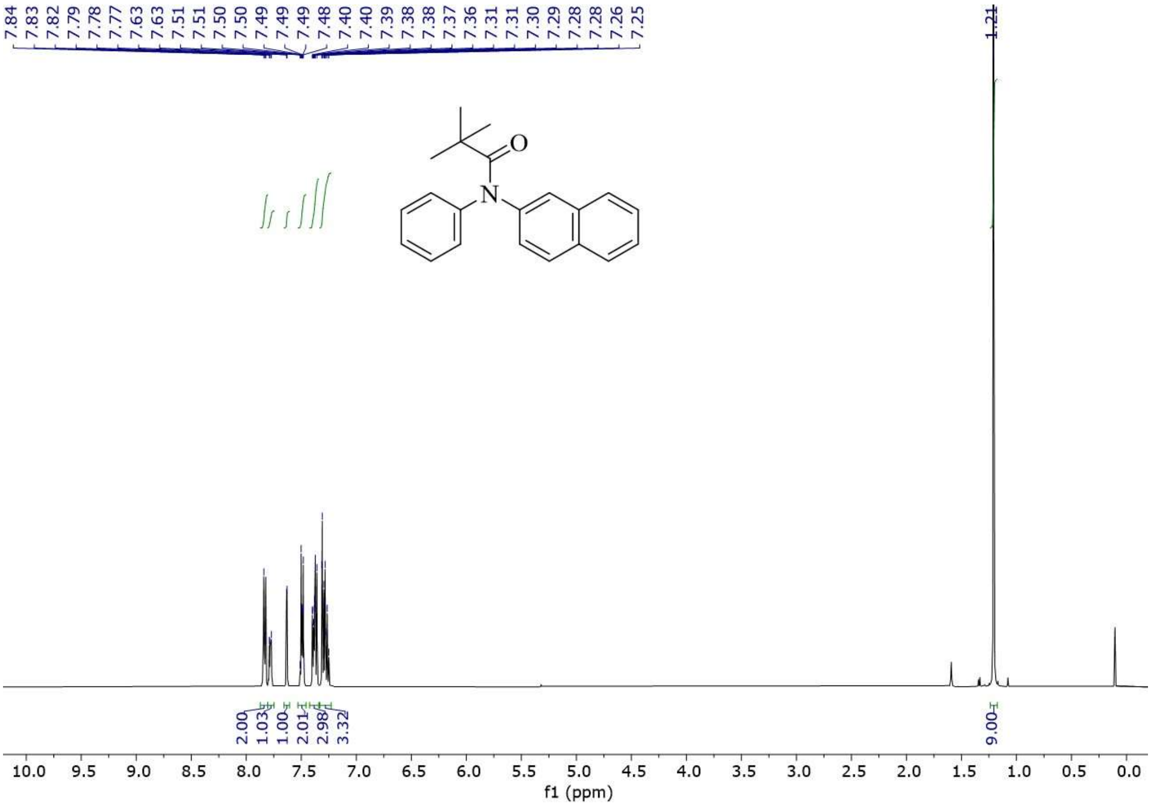

Figure **S30**. ^{1}H NMR spectrum of ***N*-(naphthalen-2-yl)-*N*-phenylpivalamide, 1l** in $CDCl_3$ (500 MHz).

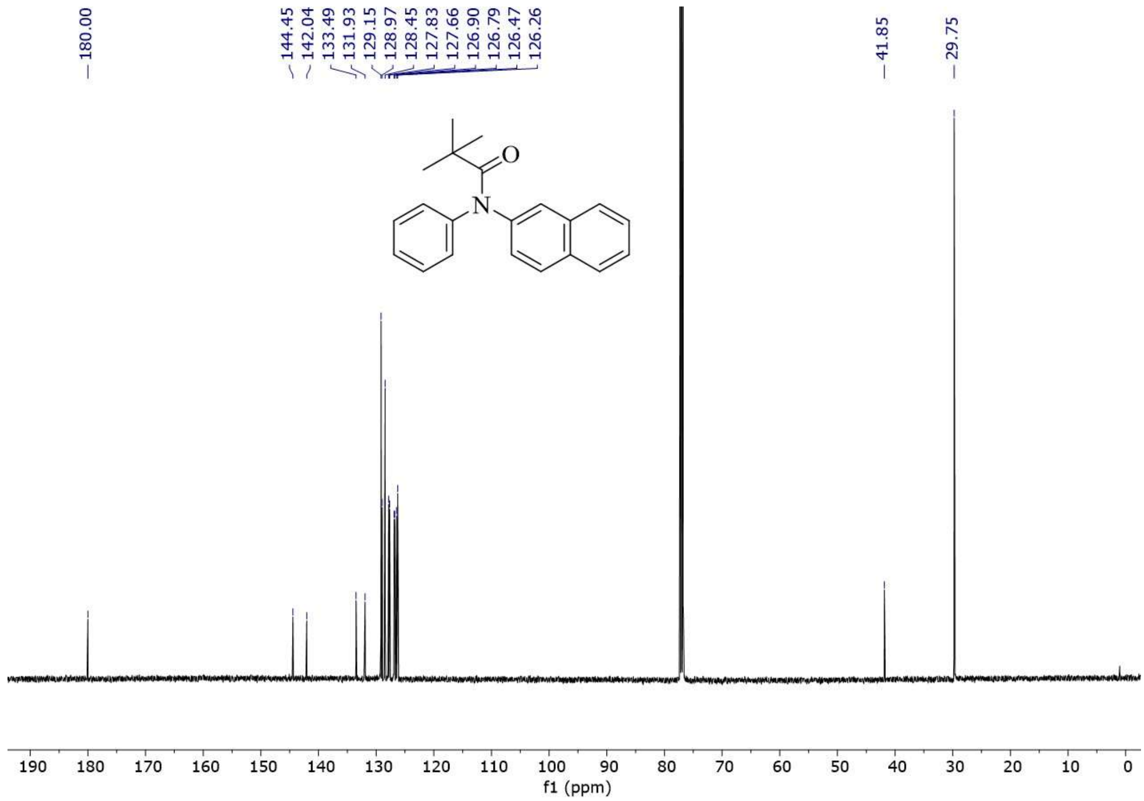

Figure **S31**. $^{13}C\{^{1}H\}$ NMR spectrum of ***N*-(naphthalen-2-yl)-*N*-phenylpivalamide, 1l** in $CDCl_3$ (126 MHz).

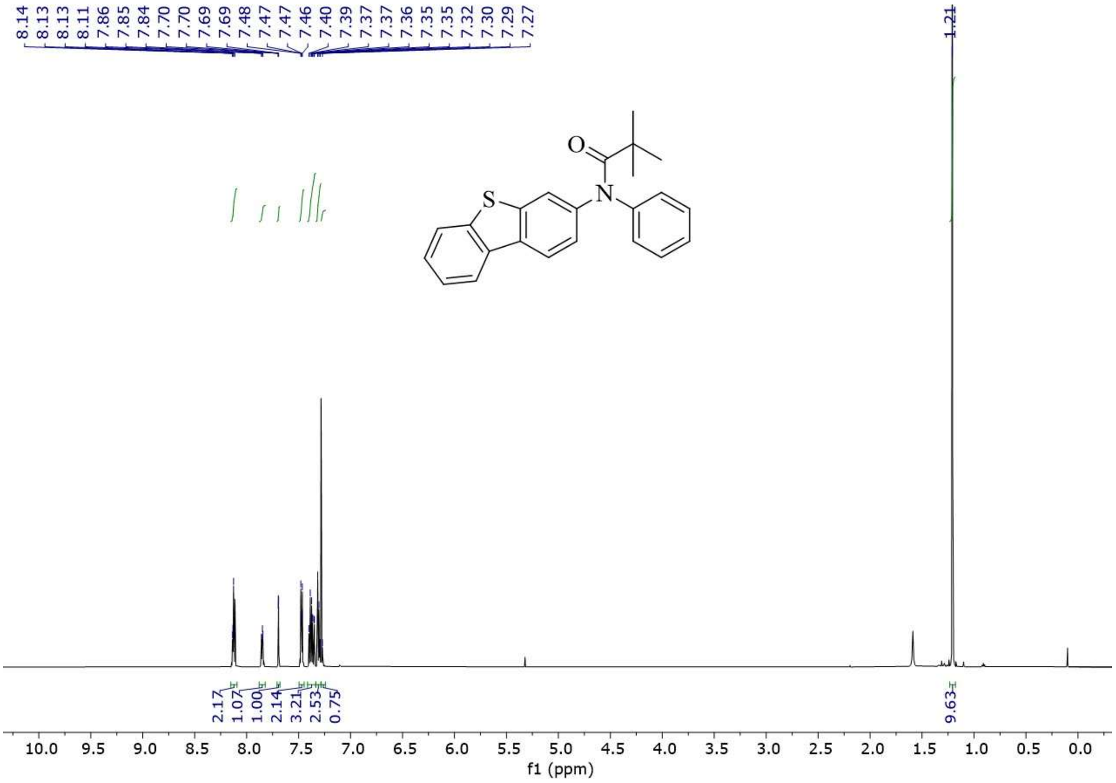

Figure **S32**. ^{1}H NMR spectrum of ***N*-(dibenzo[b,d]thiophen-3-yl)-*N*-phenylpivalamide, 1m** in $CDCl_3$ (500 MHz).

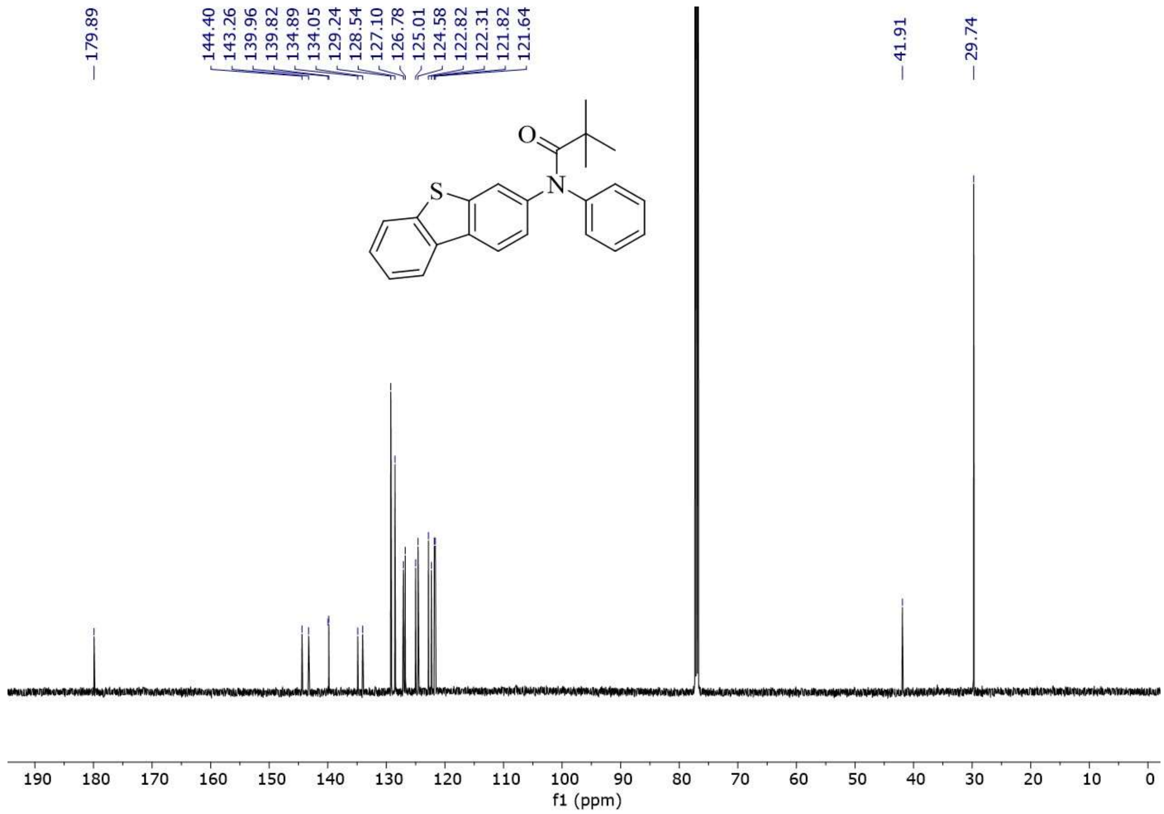

Figure **S33**. $^{13}C\{^{1}H\}$ NMR spectrum of ***N*-(naphthalen-2-yl)-*N*-phenylpivalamide, 1m** in $CDCl_3$ (126 MHz).

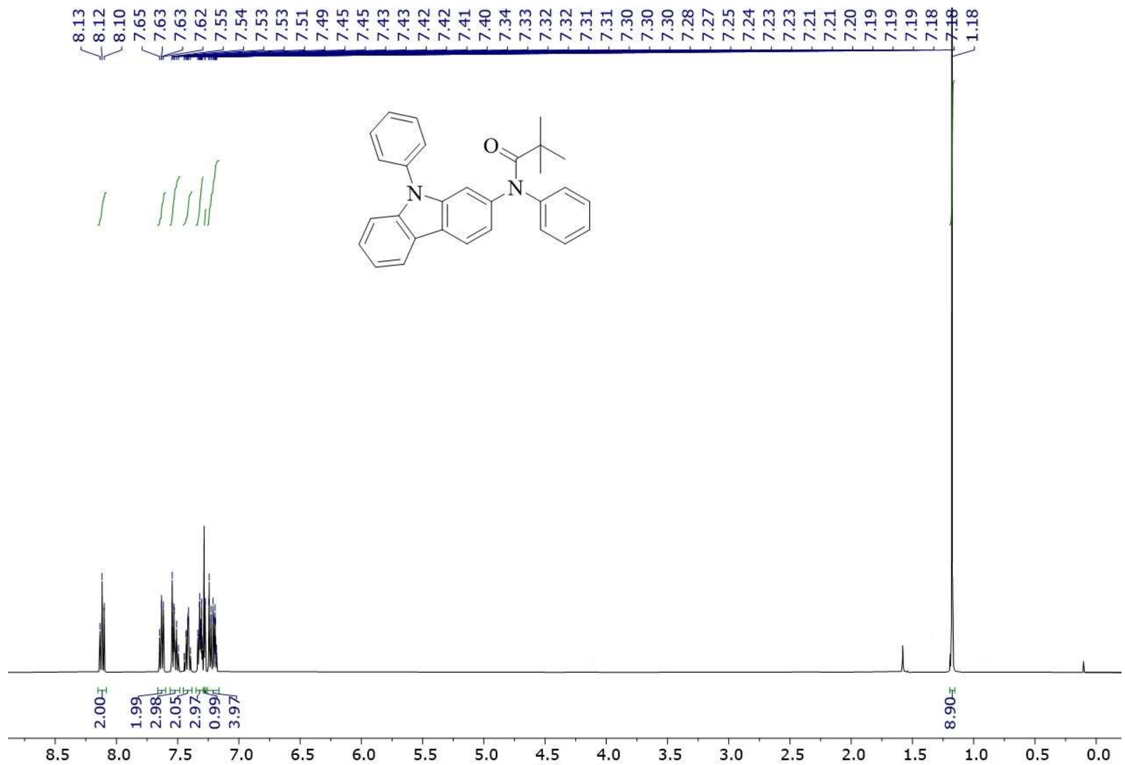

Figure **S34**. ^{1}H NMR spectrum of ***N*-phenyl-N-(9-phenyl-9H-carbazol-2-yl)pivalamide, 1n** in $CDCl_3$ (500 MHz).

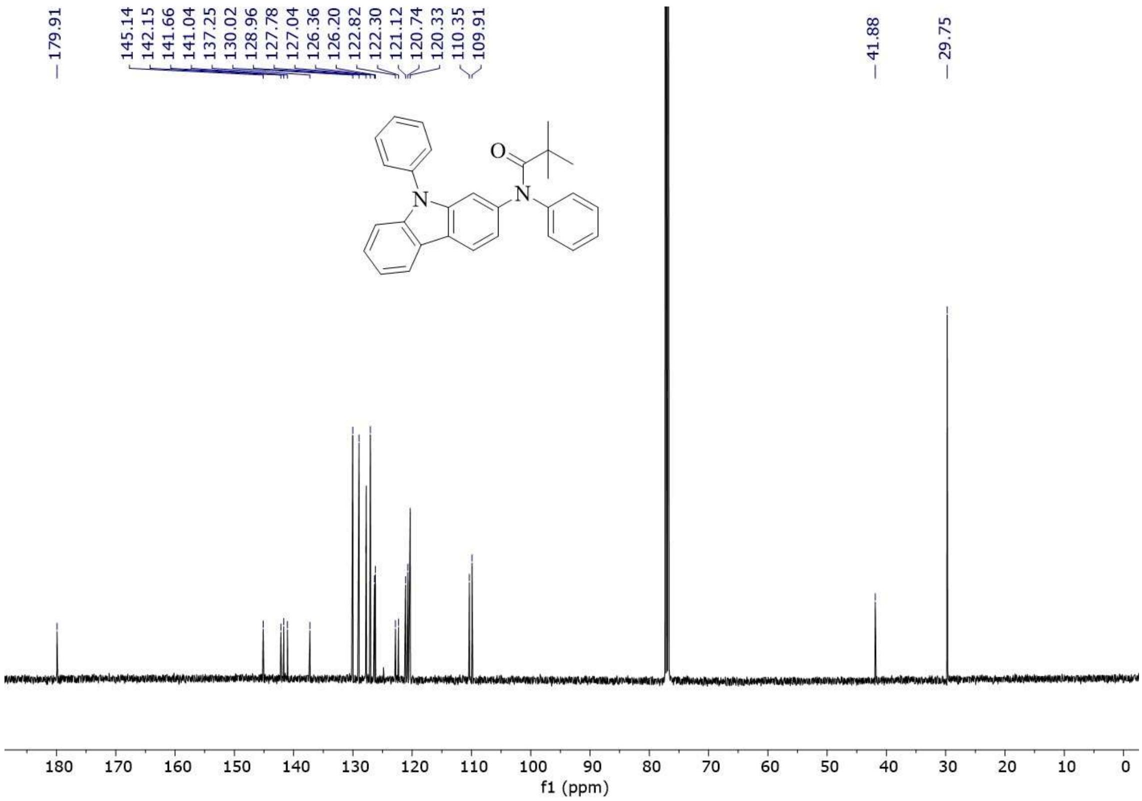

Figure **S35**. $^{13}C\{^{1}H\}$ NMR spectrum of ***N*-phenyl-N-(9-phenyl-9H-carbazol-2-yl)pivalamide, 1n** in $CDCl_3$ (126 MHz).

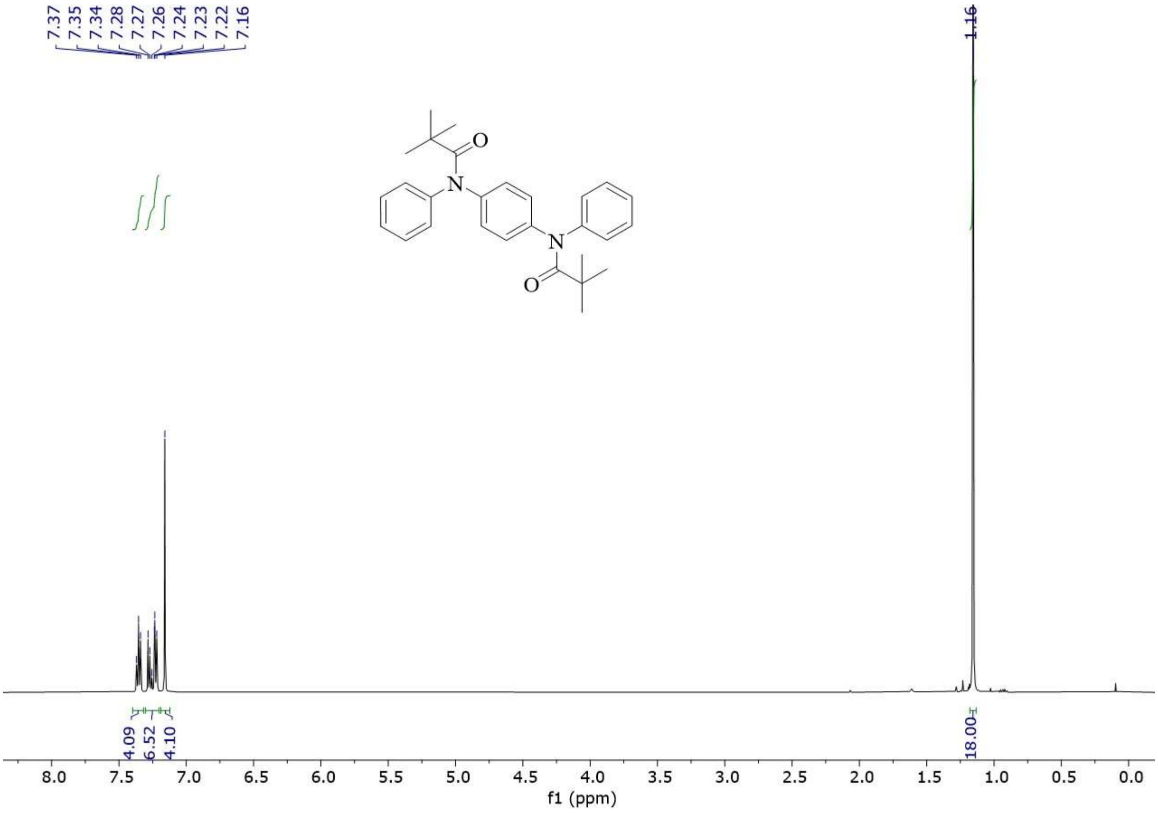

Figure **S36**. ^{1}H NMR spectrum of ***N,N′*-diphenyl-1,4-phenylenedipivalamide, 1o** in $CDCl_3$ (500 MHz).

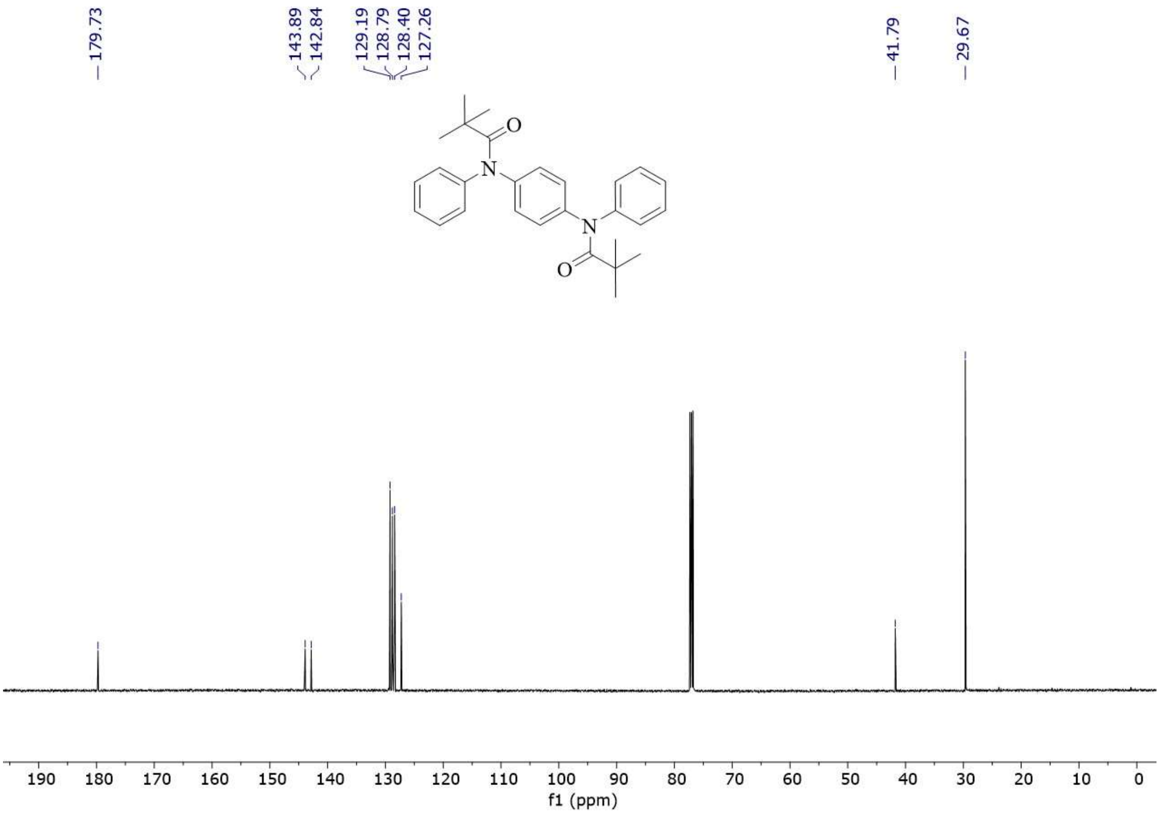

Figure **S37**. $^{13}C\{^{1}H\}$ NMR spectrum of ***N,N′*-diphenyl-1,4-phenylenedipivalamide, 1o** in $CDCl_3$ (126 MHz).

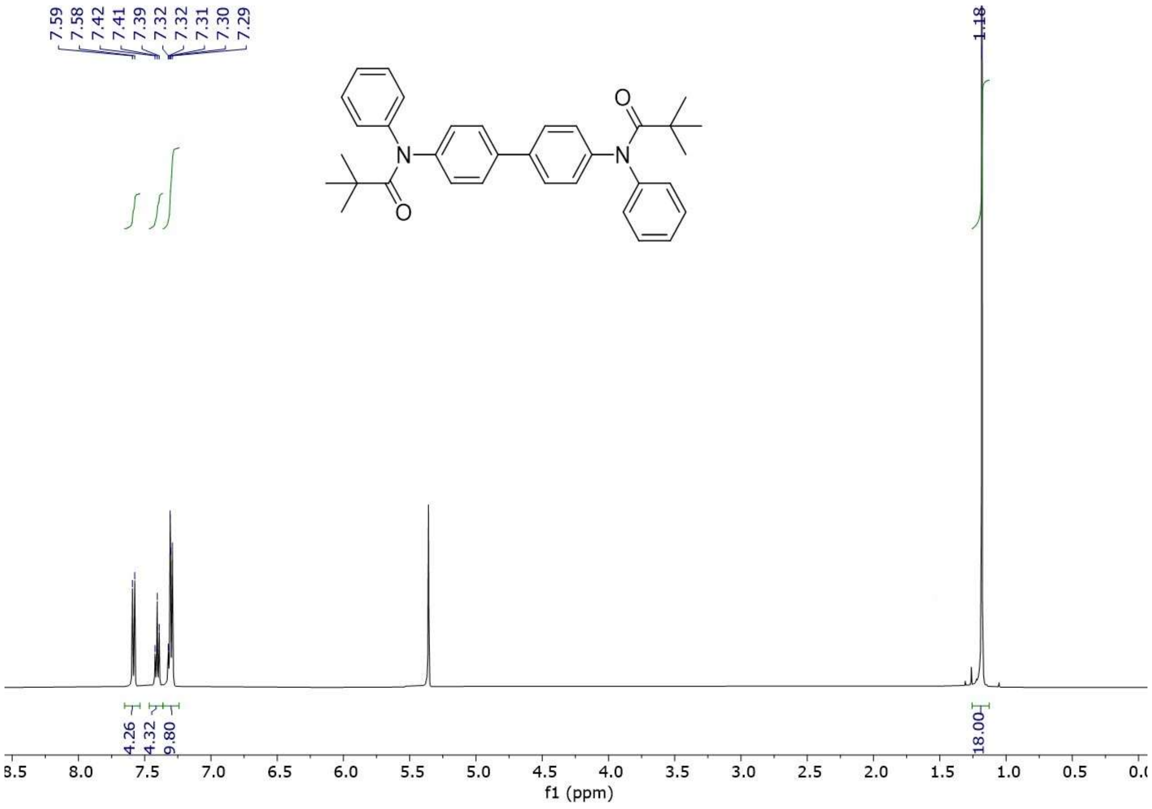

Figure **S38**. ^{1}H NMR spectrum of ***N*-[4-[4-(*N*-acetylanilino)phenyl]phenyl]-*N*-phenylpivalamide, 1p** in $CDCl_3$ (500 MHz).

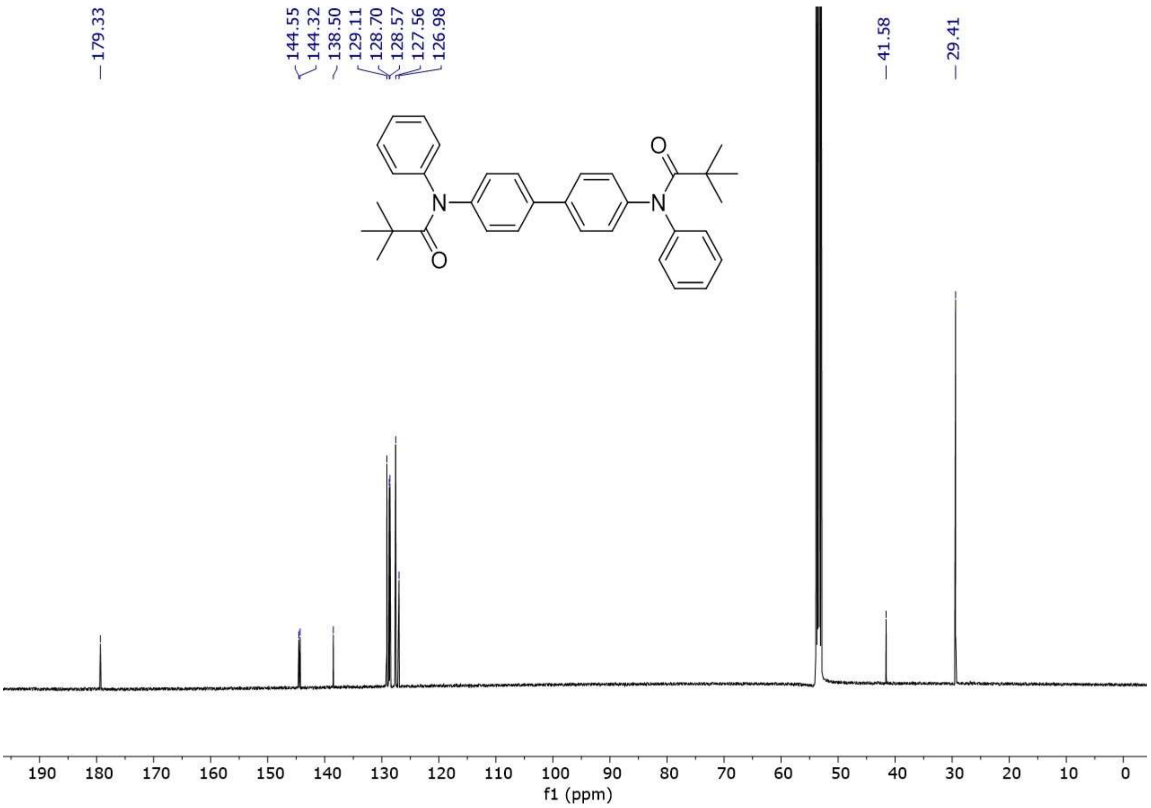

Figure **S39**. $^{13}C\{^{1}H\}$ NMR spectrum of ***N*-[4-[4-(*N*-acetylanilino)phenyl]phenyl]-*N*-phenylpivalamide, 1p** in $CDCl_3$ (126 MHz).

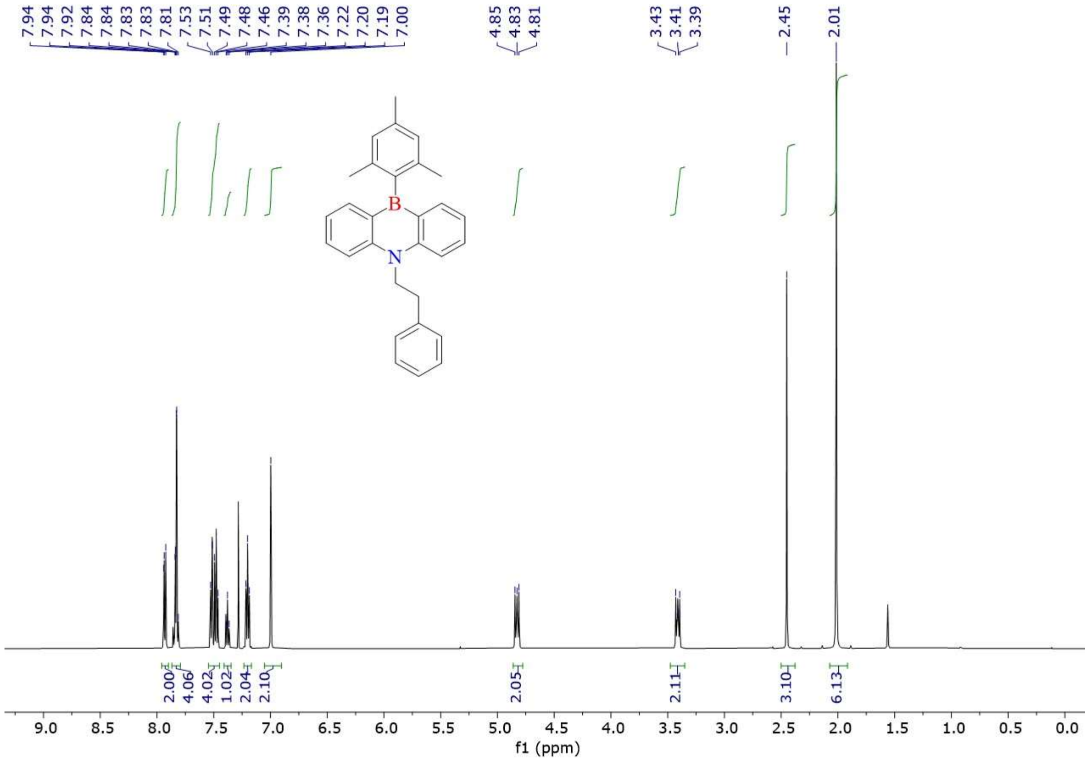

Figure **S40**. ^{1}H NMR spectrum of compound **4a** in $CDCl_3$ (500 MHz).

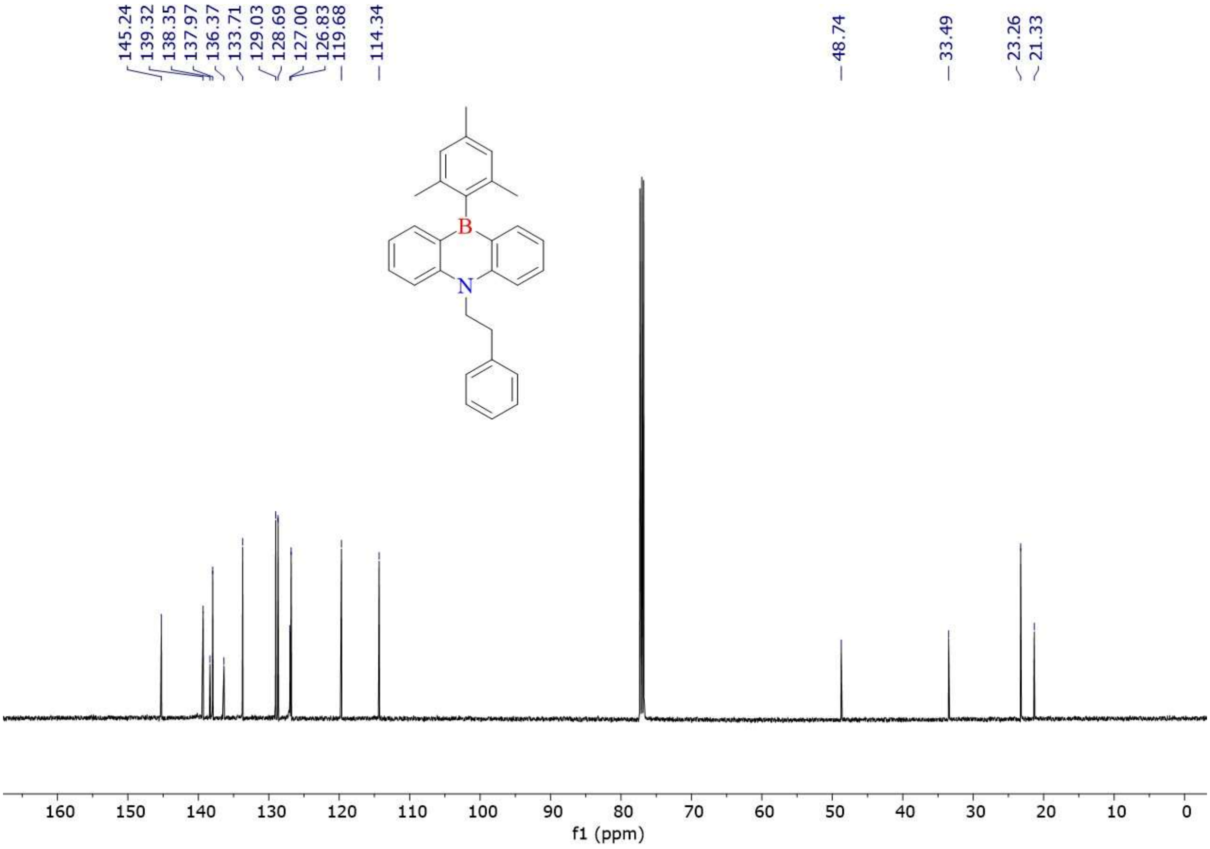

Figure **S41**. $^{13}C\{^{1}H\}$ NMR spectrum of compound **4a** in $CDCl_3$ (126 MHz).

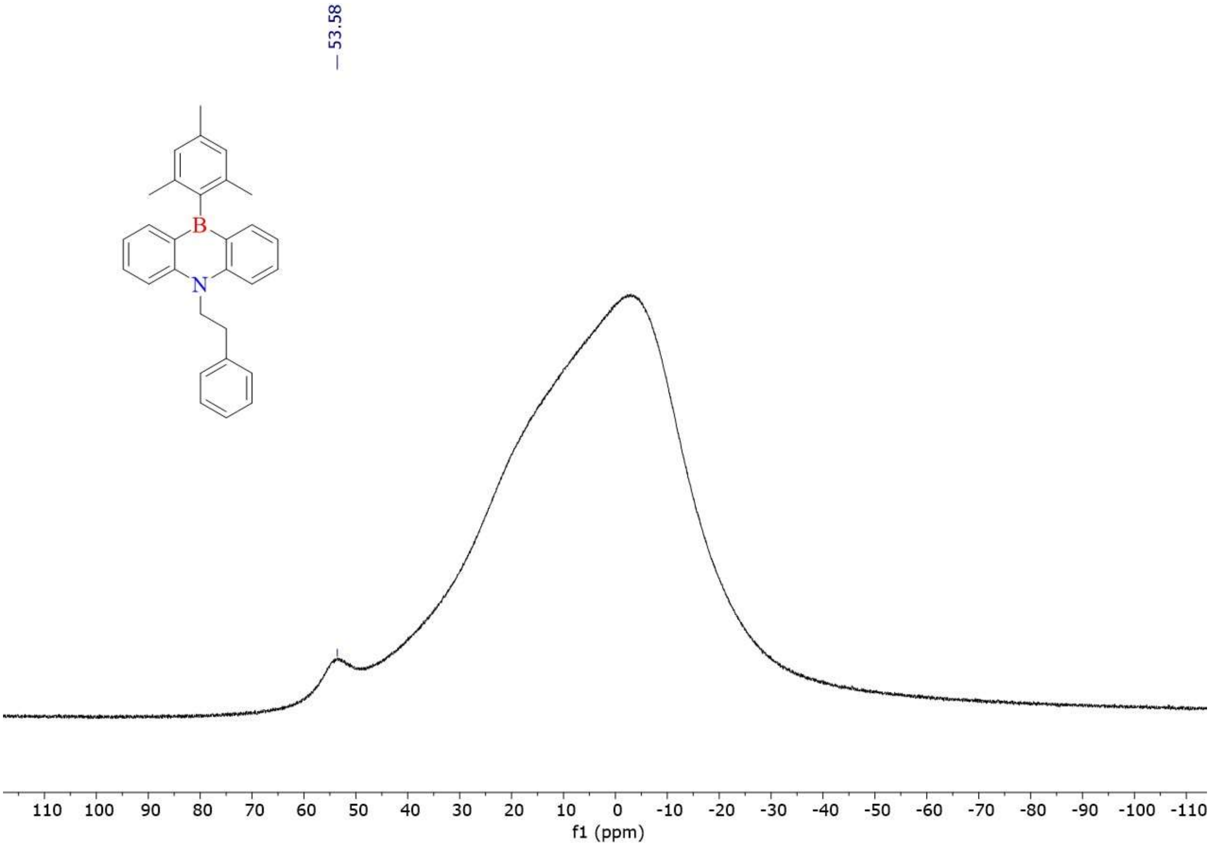

Figure **S42**. ^{11}B NMR spectrum of compound **4a** in $CDCl_3$ (160 MHz).

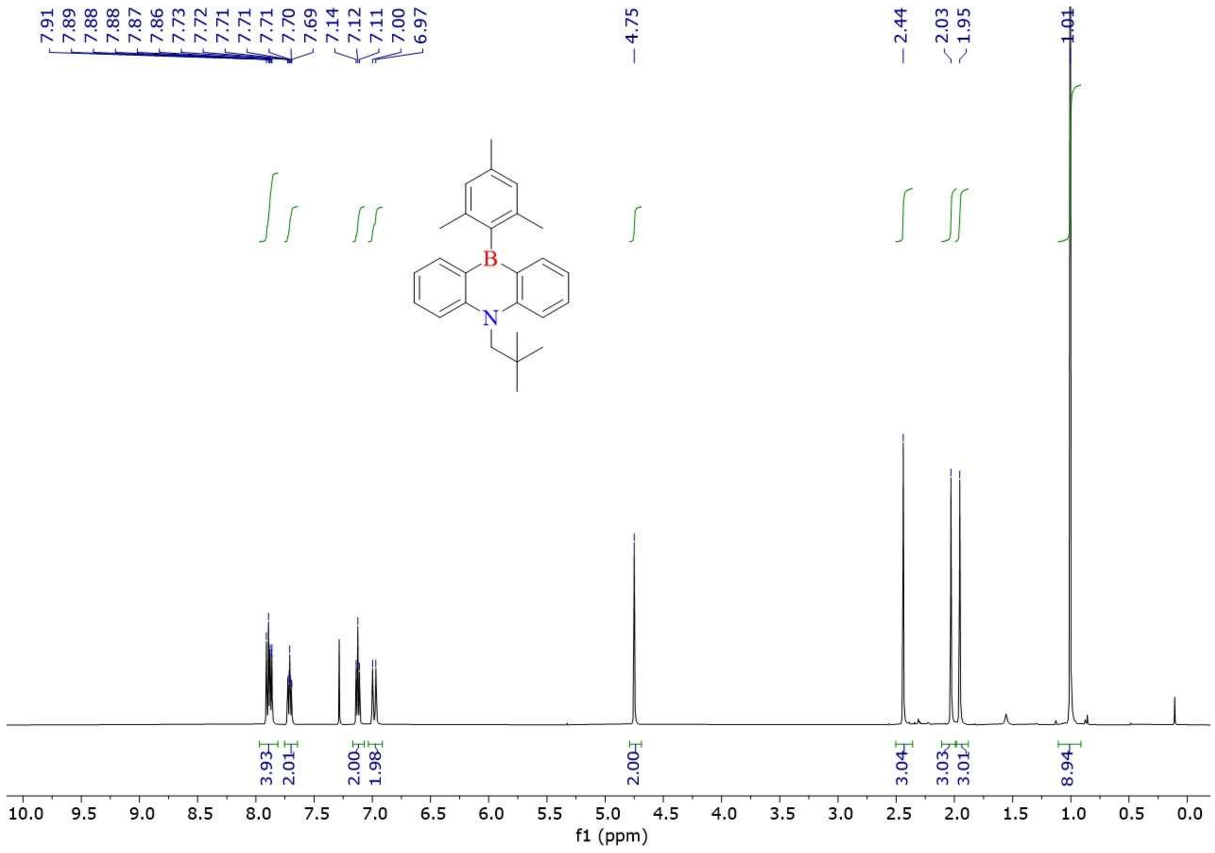

Figure **S43**. ^{1}H NMR spectrum of compound **4b** in $CDCl_3$ (500 MHz).

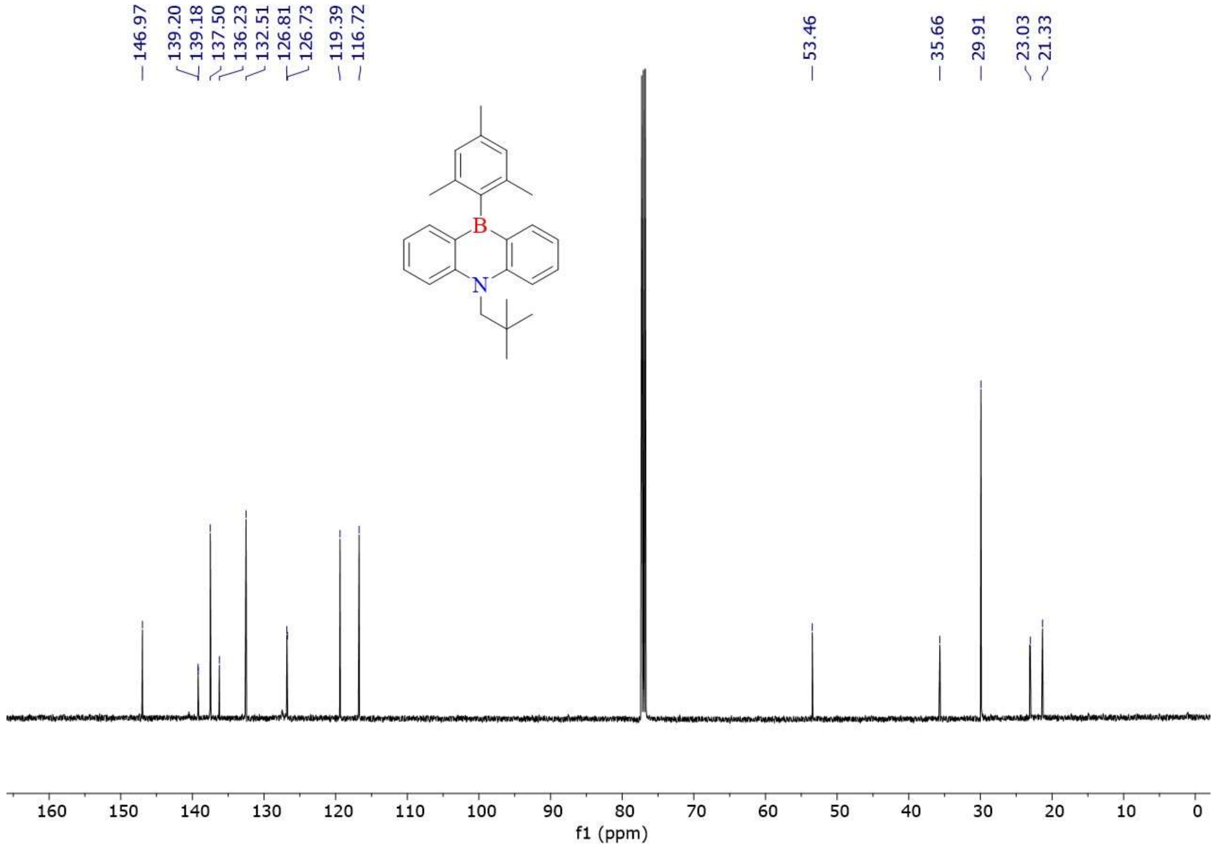

Figure **S44**. $^{13}C\{^{1}H\}$ NMR spectrum of compound **4a** in $CDCl_3$ (126 MHz).

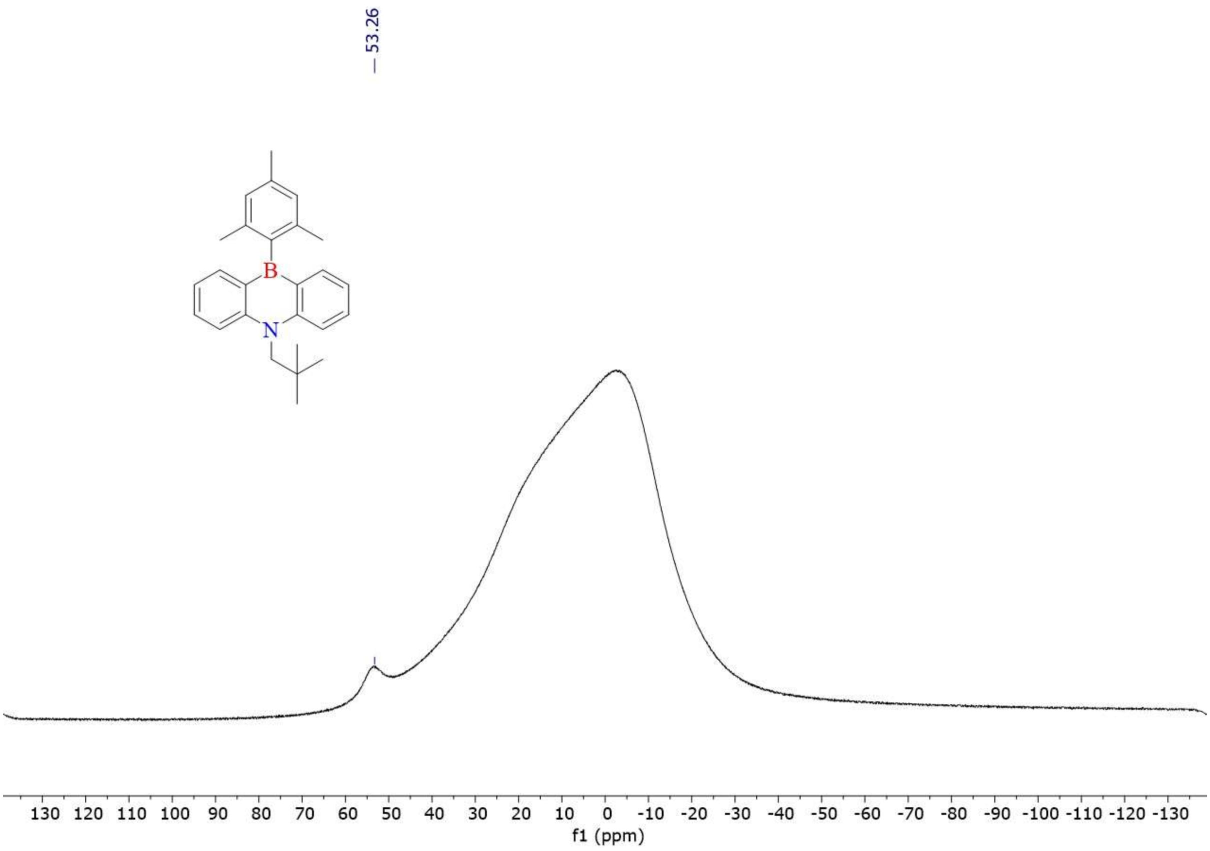

Figure **S45**. ^{11}B NMR spectrum of compound **4b** in $CDCl_3$ (160 MHz).

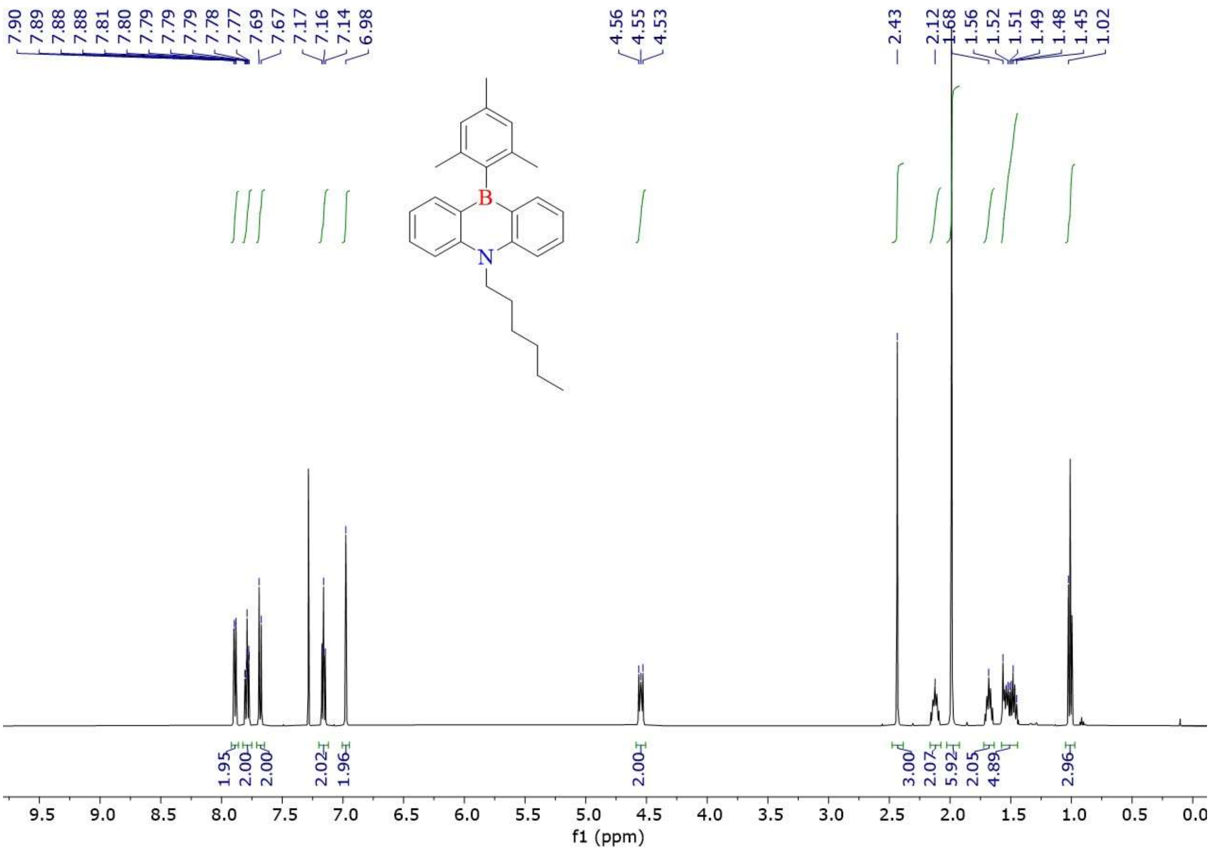

Figure **S46**. ^{1}H NMR spectrum of compound **4c** in $CDCl_3$ (500 MHz).

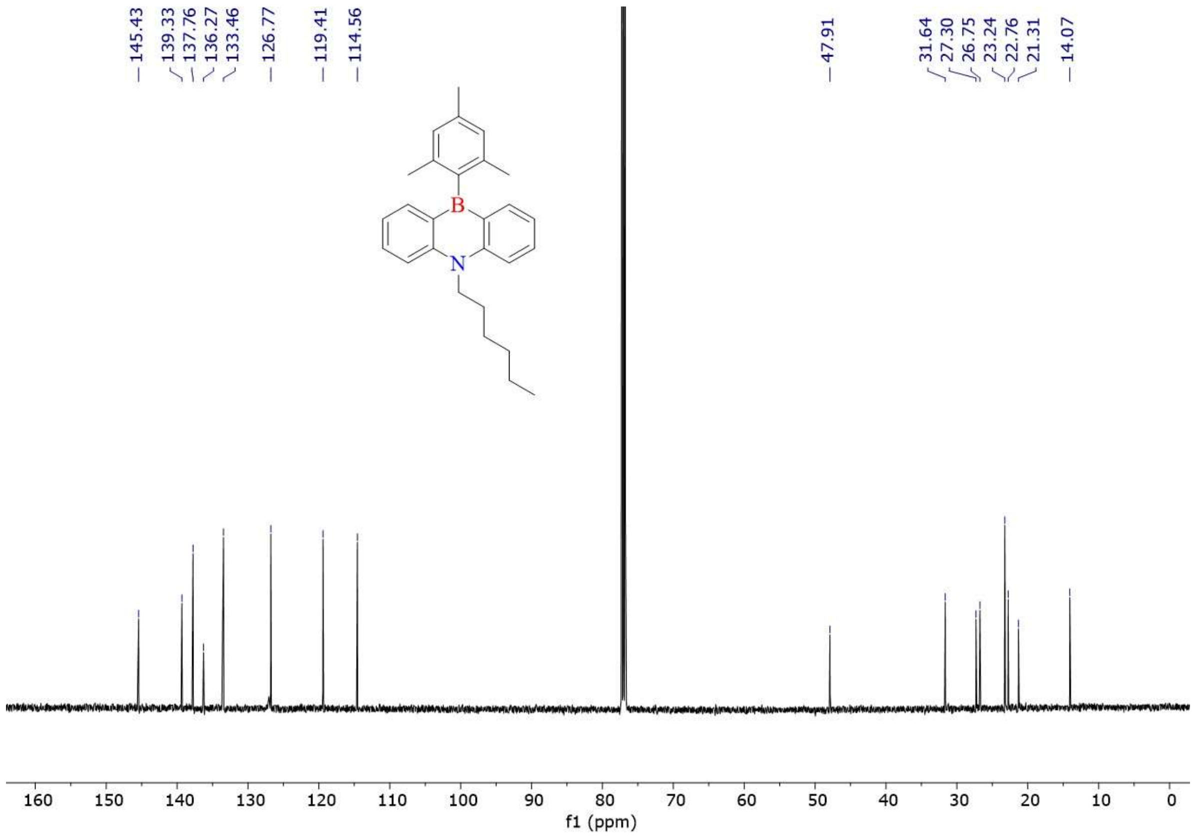

Figure **S47**. $^{13}C\{^{1}H\}$ NMR spectrum of compound **4c** in $CDCl_3$ (126 MHz).

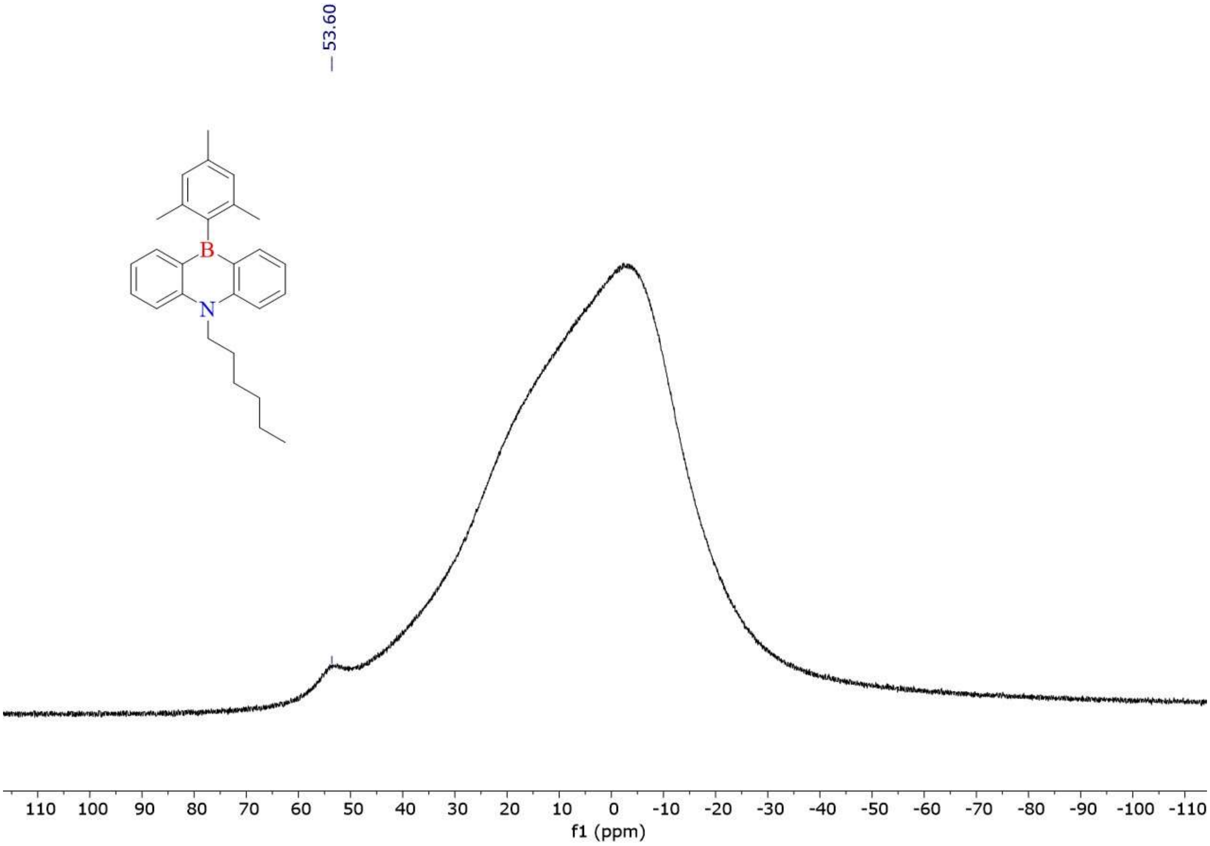

Figure **S48**. ^{11}B NMR spectrum of compound **4c** in $CDCl_3$ (160 MHz).

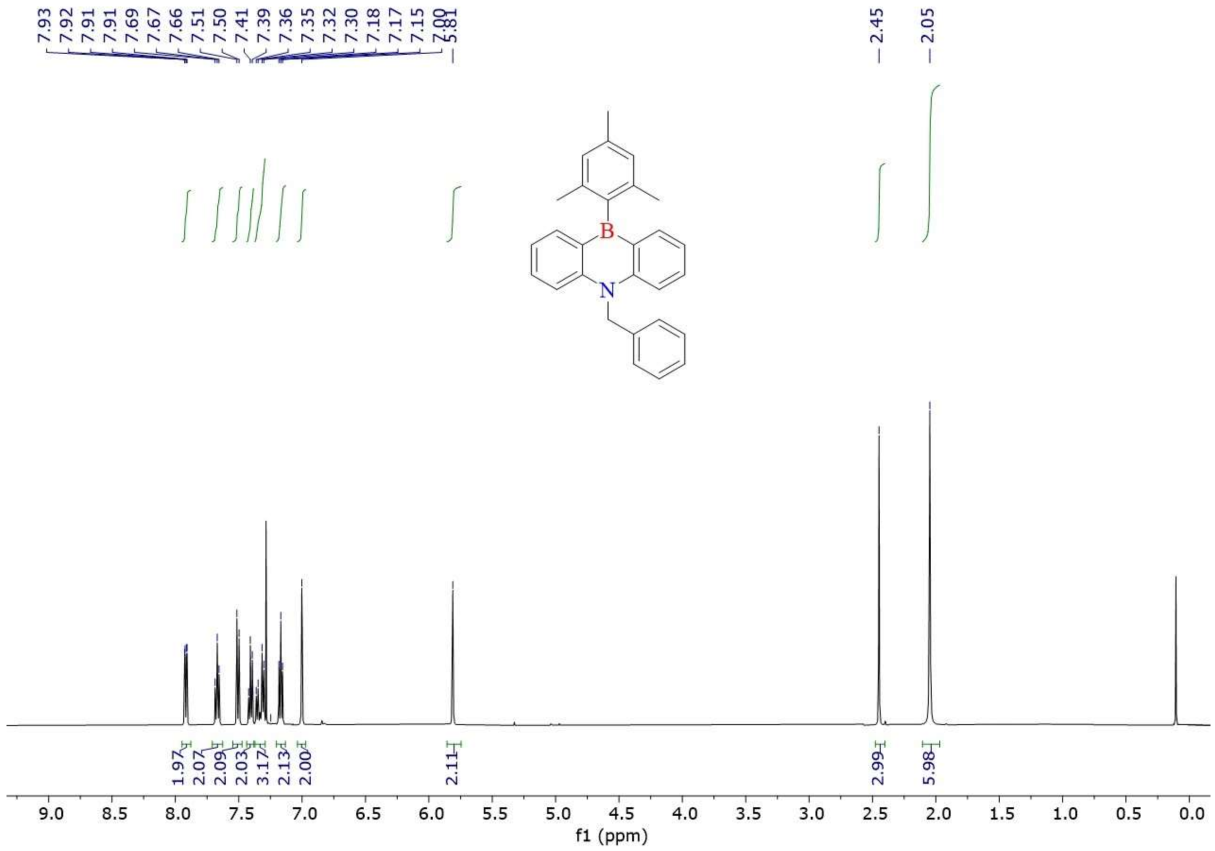

Figure **S49**. ^{1}H NMR spectrum of compound **4d** in $CDCl_3$ (500 MHz).

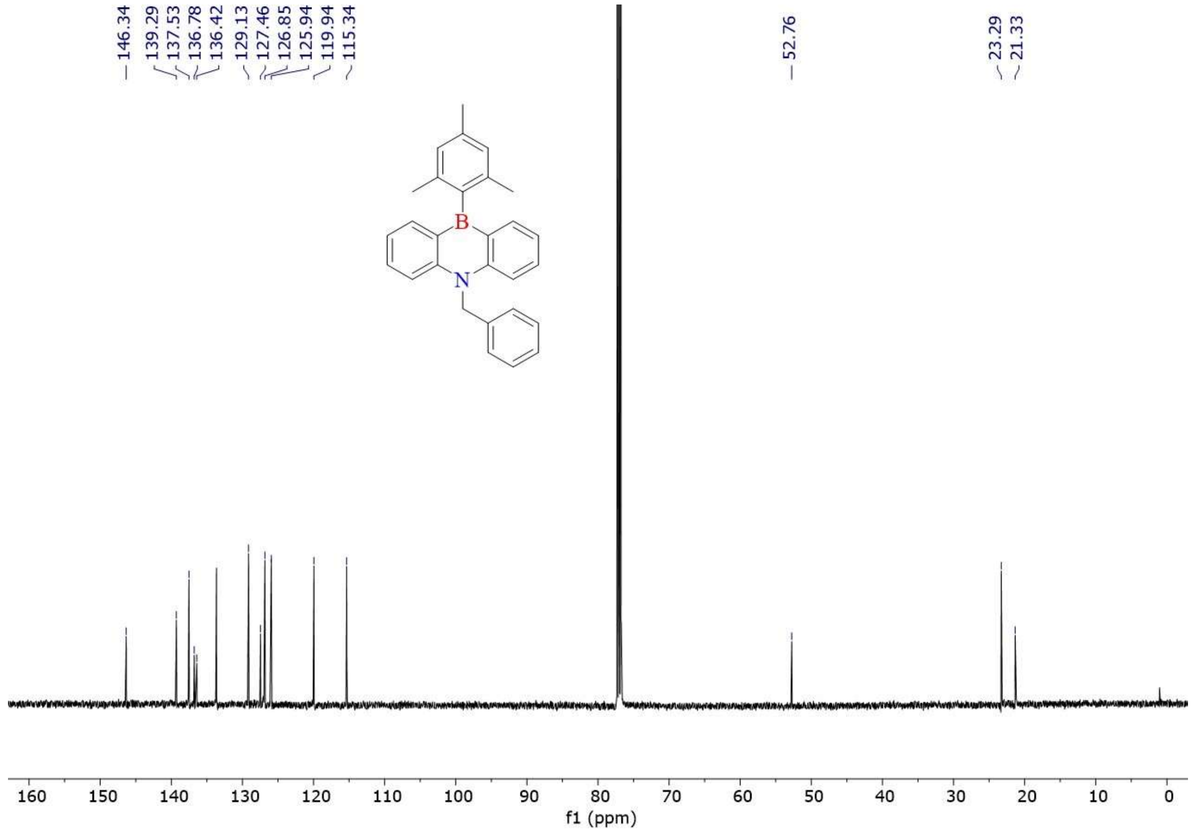

Figure **S50**. $^{13}C\{^{1}H\}$ NMR spectrum of compound **4d** in $CDCl_3$ (126 MHz).

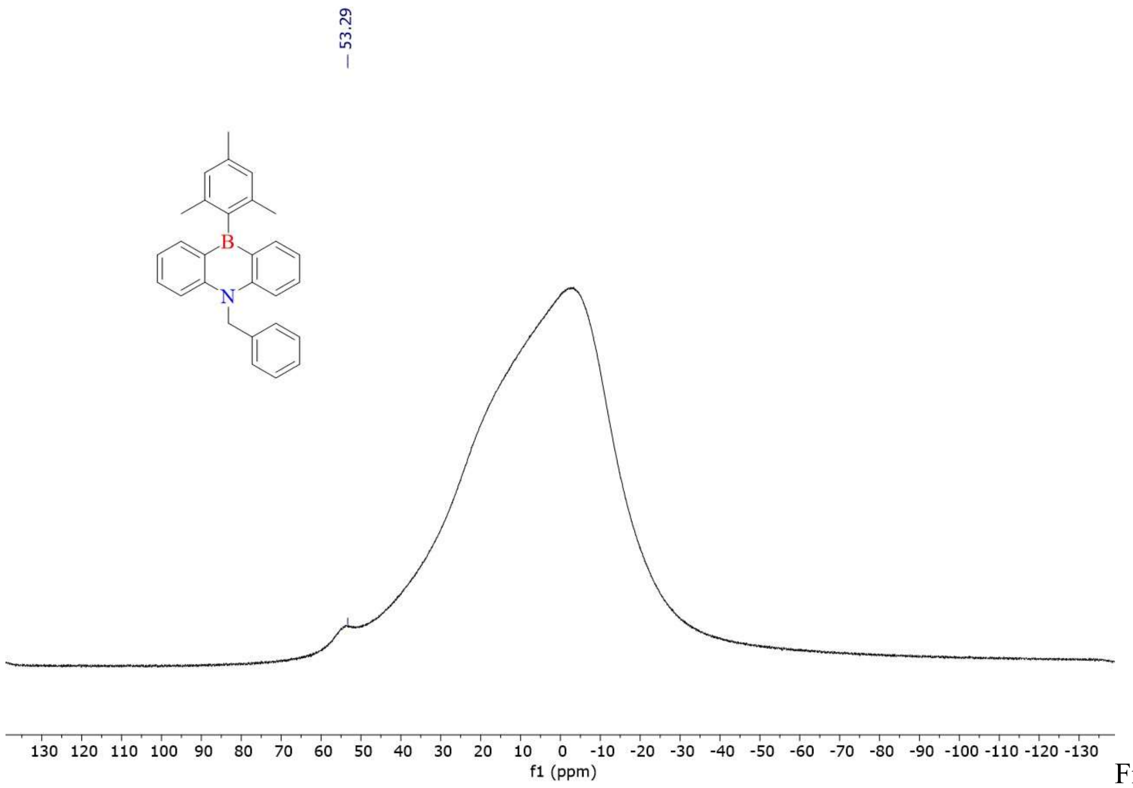

Figure **S51**. ^{11}B NMR spectrum of compound **4d** in $CDCl_3$ (160 MHz).

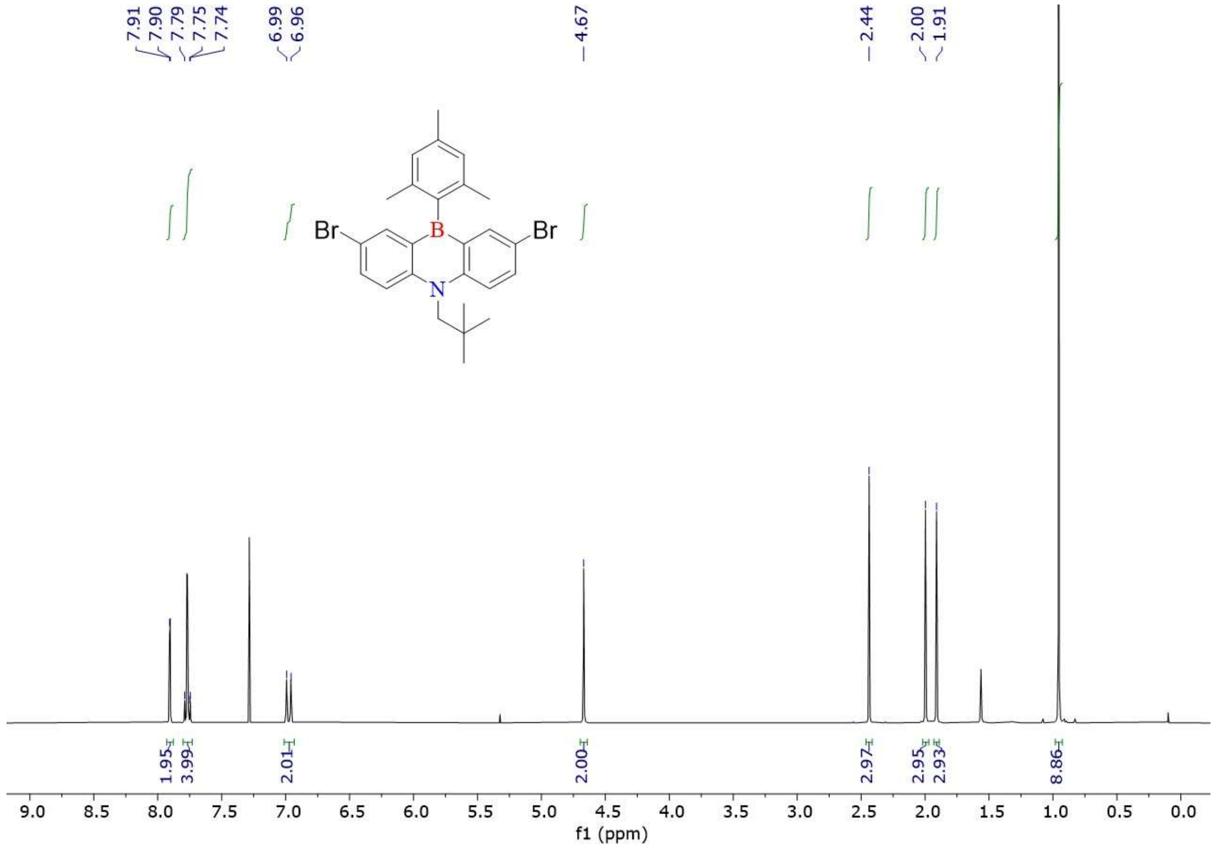

Figure **S52**. ^{1}H NMR spectrum of compound **4e** in $CDCl_3$ (500 MHz).

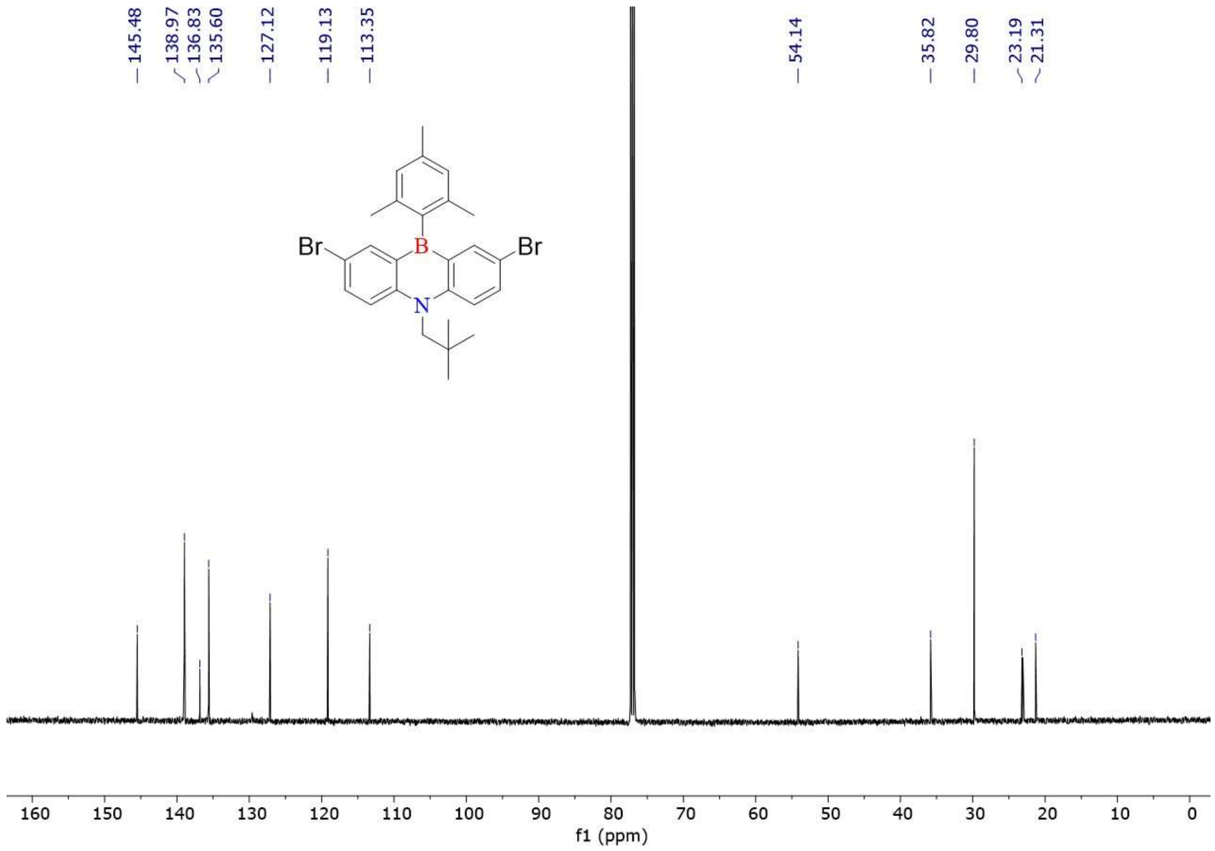

Figure **S53**. $^{13}C\{^{1}H\}$ NMR spectrum of compound **4e** in $CDCl_3$ (126 MHz).

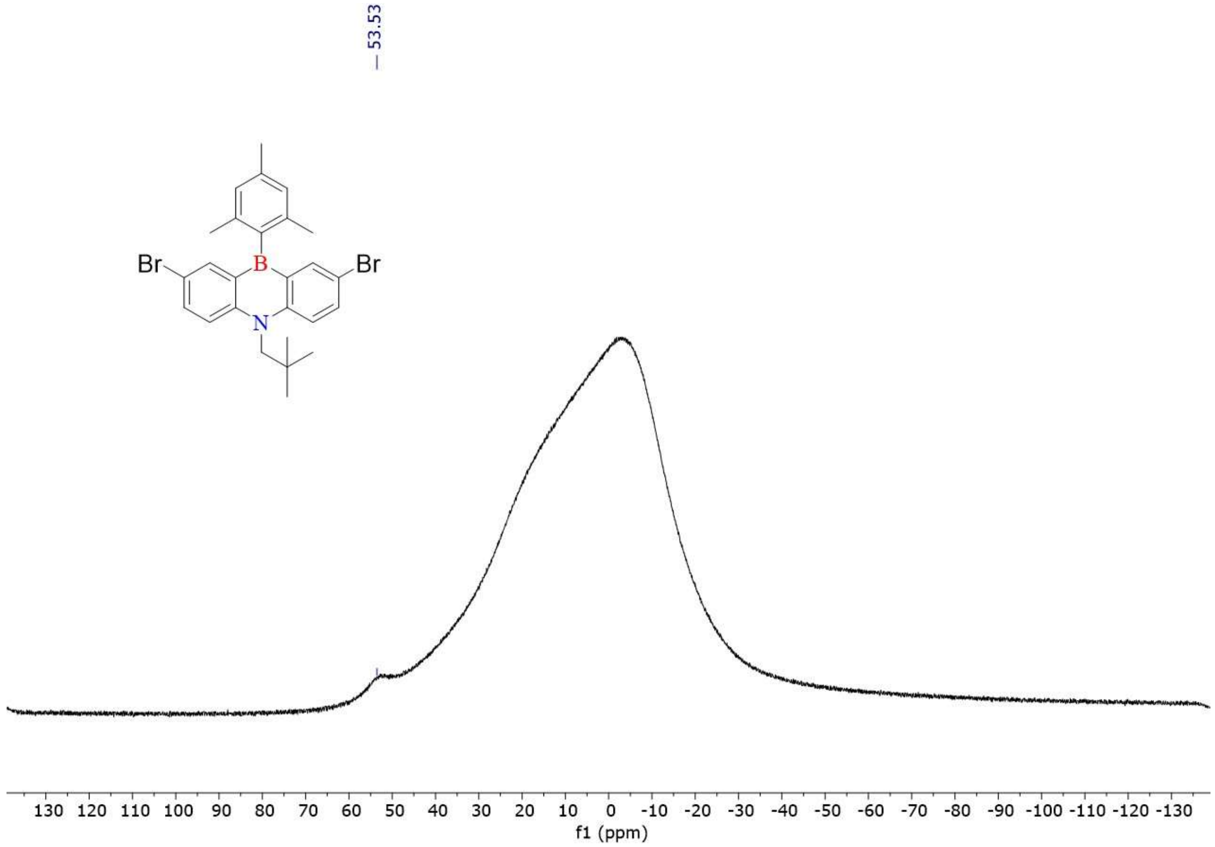

Figure **S54**. ^{11}B NMR spectrum of compound **4e** in $CDCl_3$ (160 MHz).

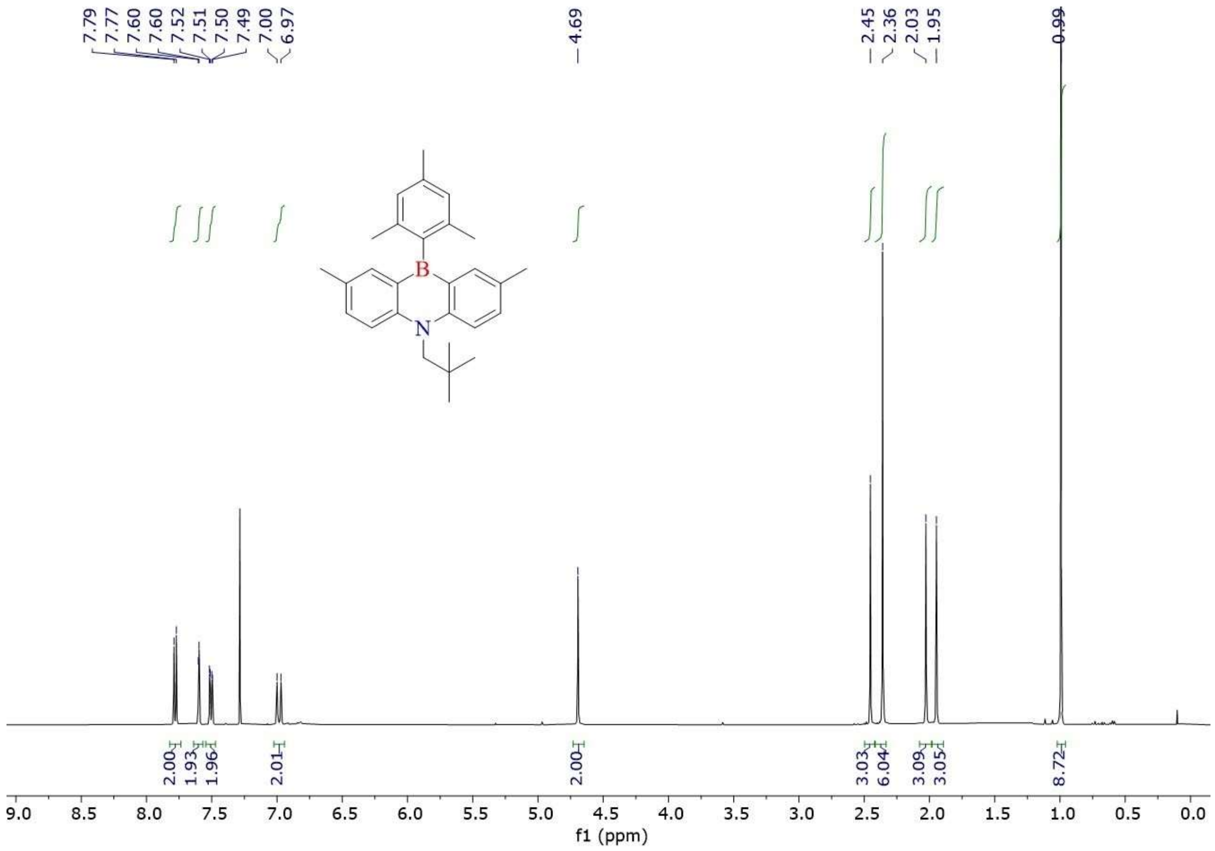

Figure **S55**. ^{1}H NMR spectrum of compound **4f** in $CDCl_3$ (500 MHz).

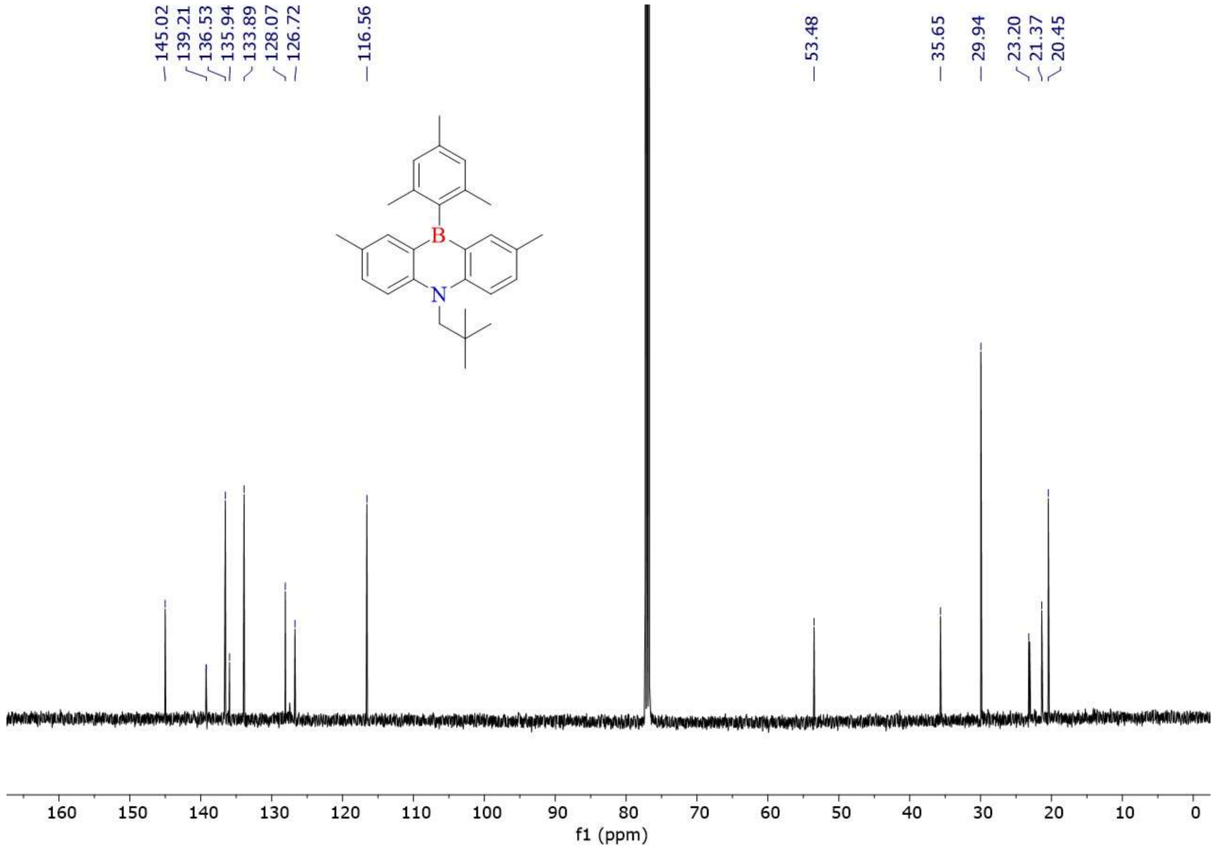

Figure **S56**. $^{13}C\{^{1}H\}$ NMR spectrum of compound **4f** in $CDCl_3$ (126 MHz).

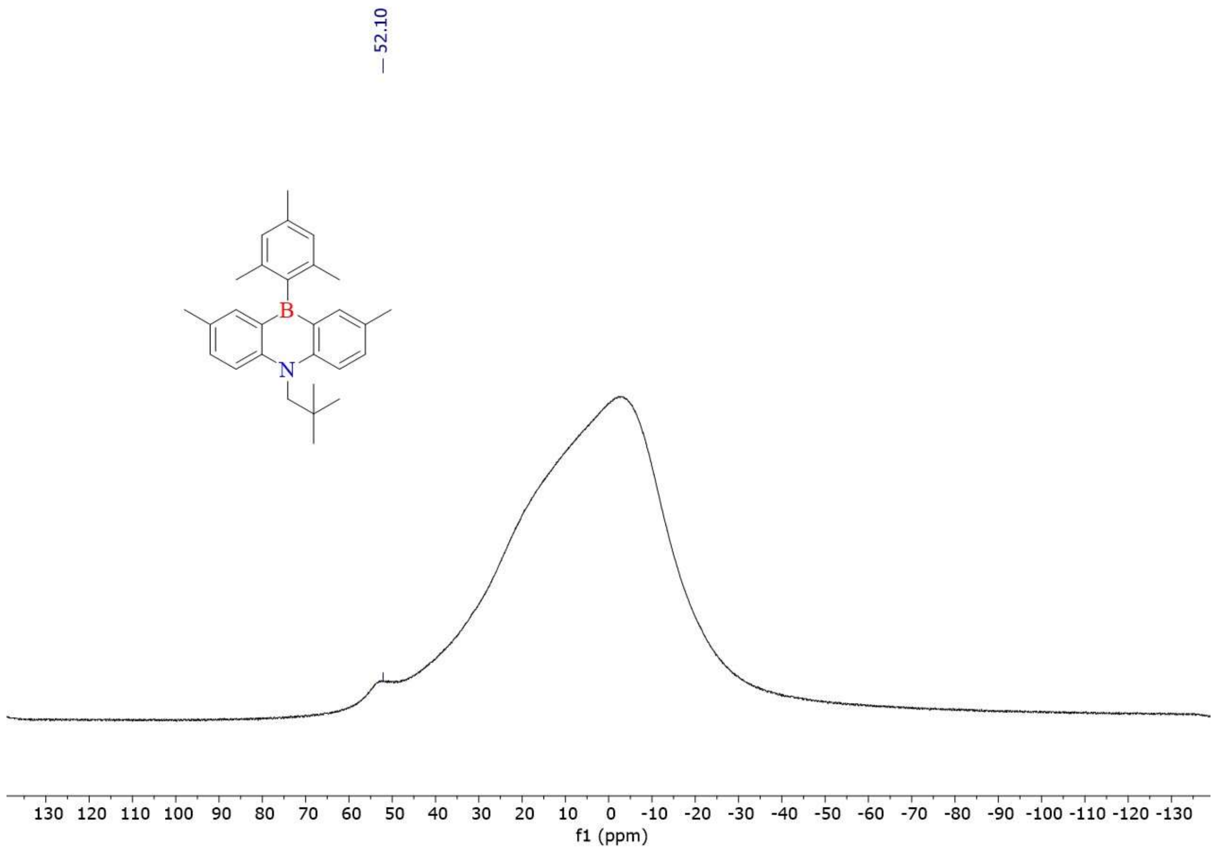

Figure **S57**. ^{11}B NMR spectrum of compound **4f** in $CDCl_3$ (160 MHz).

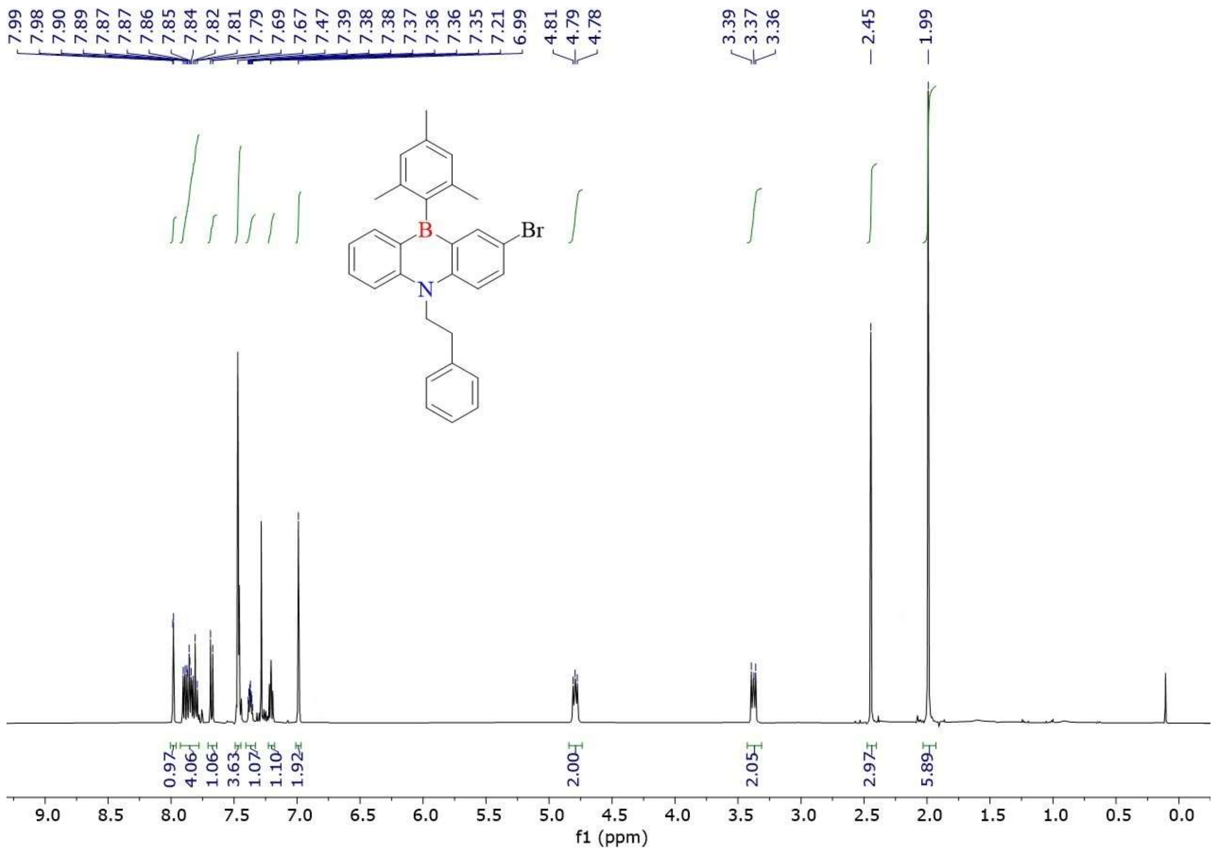

Figure **S58**. ^{1}H NMR spectrum of compound **4g** in $CDCl_3$ (500 MHz).

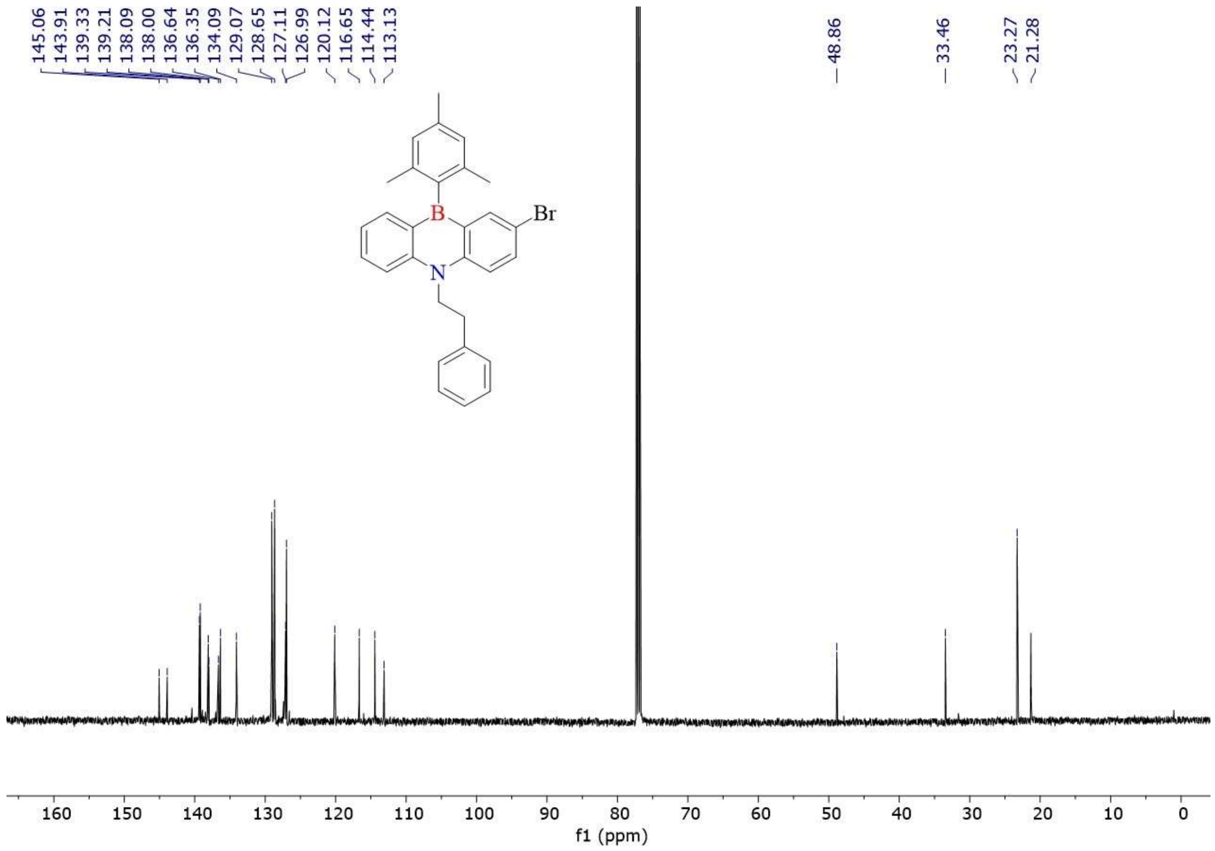

Figure **S59**. $^{13}C\{^{1}H\}$ NMR spectrum of compound **4g** in $CDCl_3$ (126 MHz).

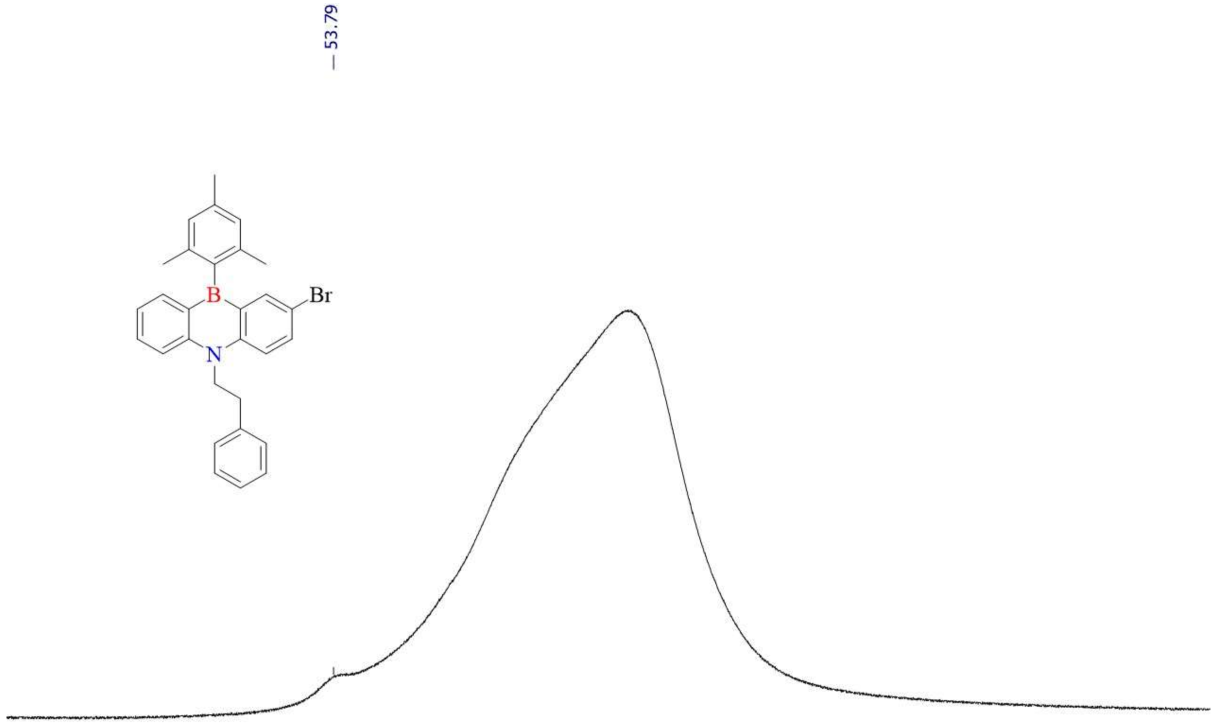

Figure **S60**. ^{11}B NMR spectrum of compound **4g** in $CDCl_3$ (160 MHz).

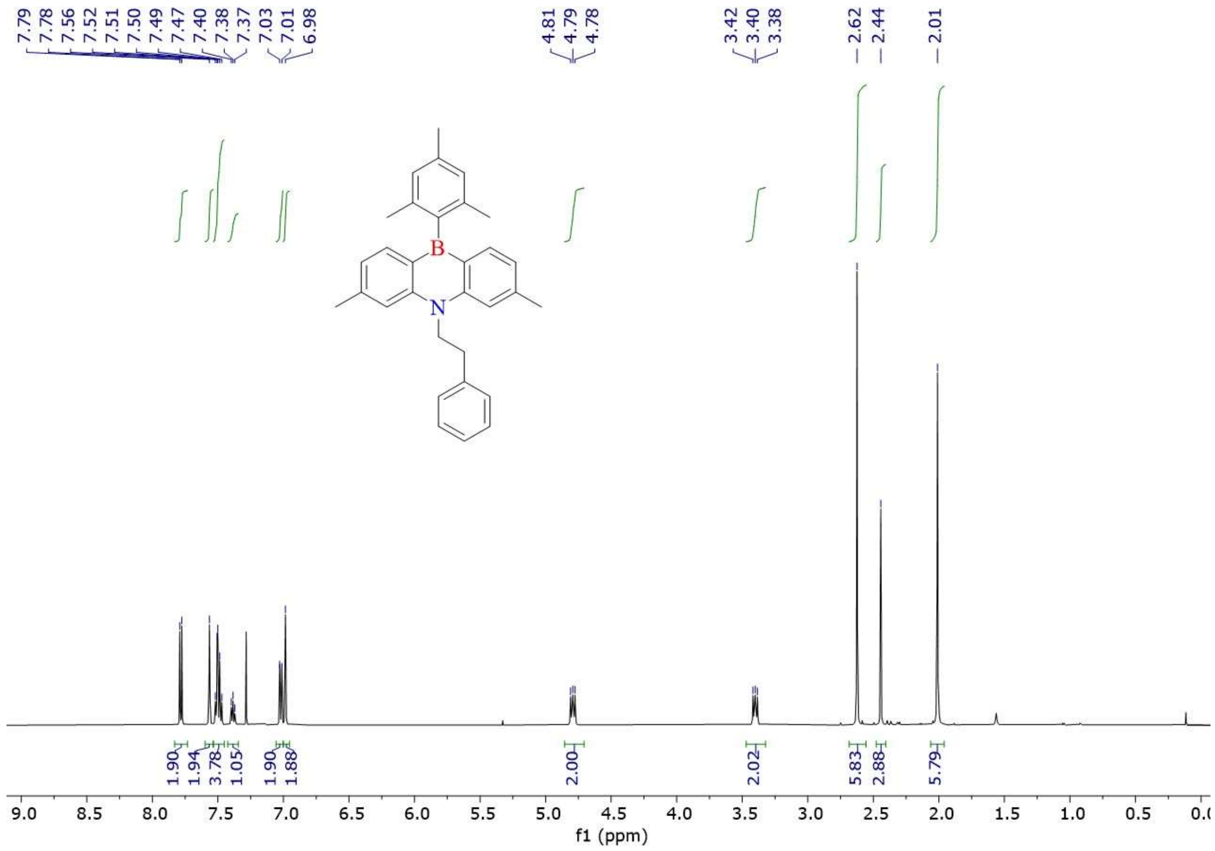

Figure **S61**. ^{1}H NMR spectrum of compound **4h** in $CDCl_3$ (500 MHz).

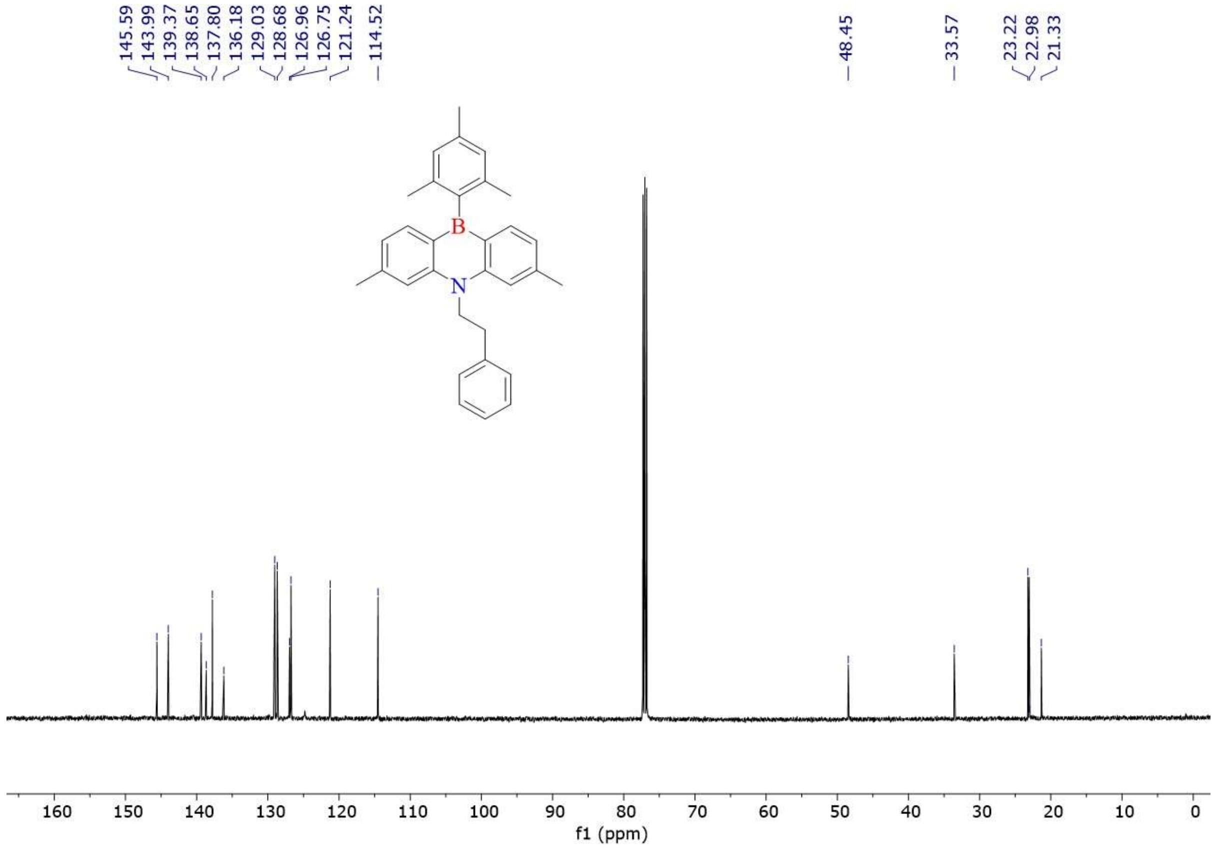

Figure **S62**. $^{13}C\{^{1}H\}$ NMR spectrum of compound **4h** in $CDCl_3$ (126 MHz).

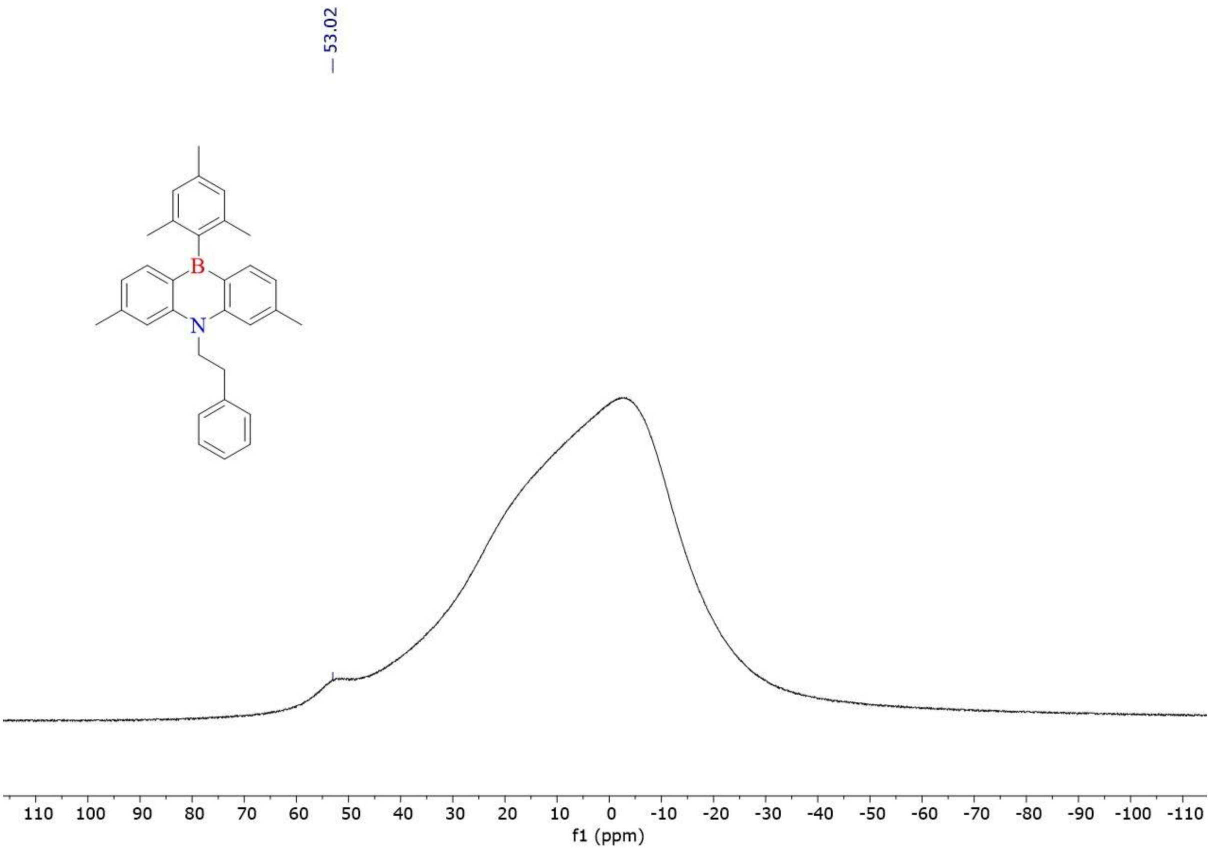

Figure **S63**. ^{11}B NMR spectrum of compound **4h** in $CDCl_3$ (160 MHz).

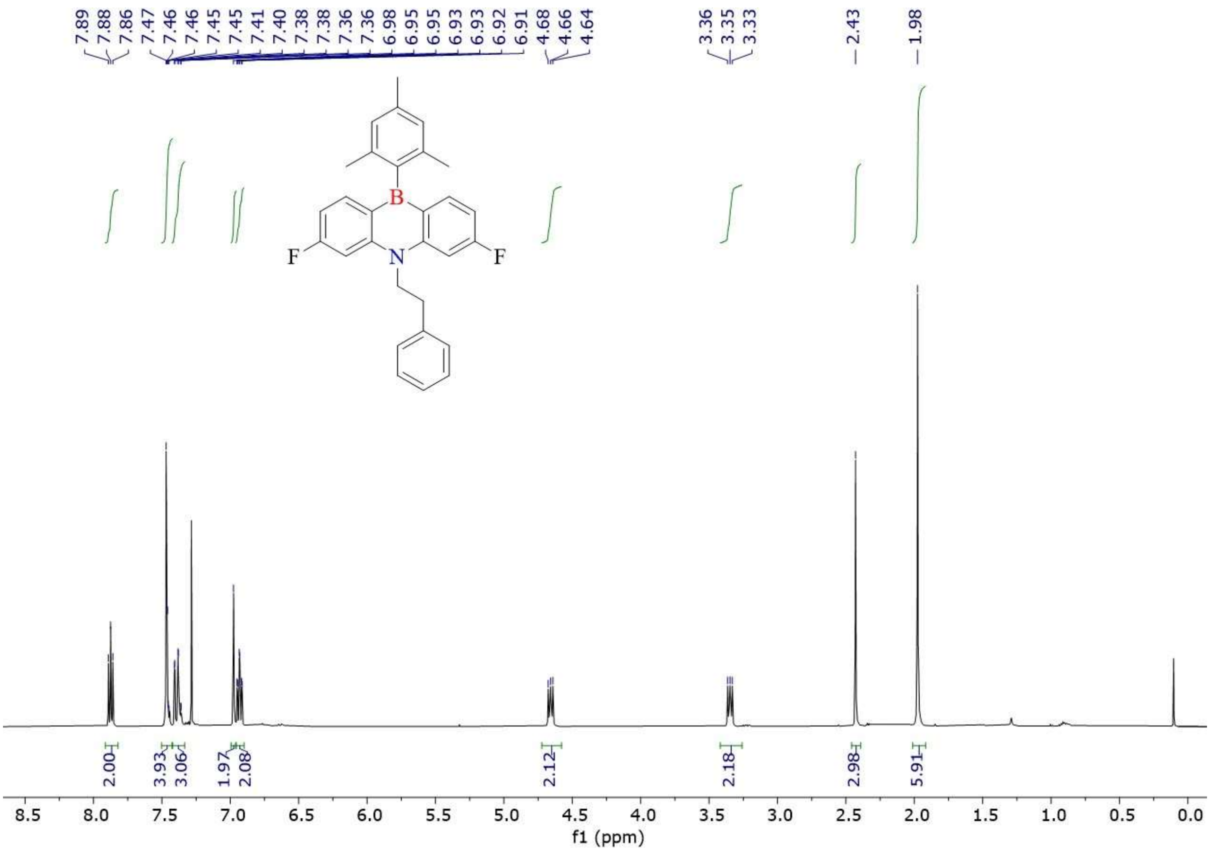

Figure **S64**. ^{1}H NMR spectrum of compound **4i** in $CDCl_3$ (500 MHz).

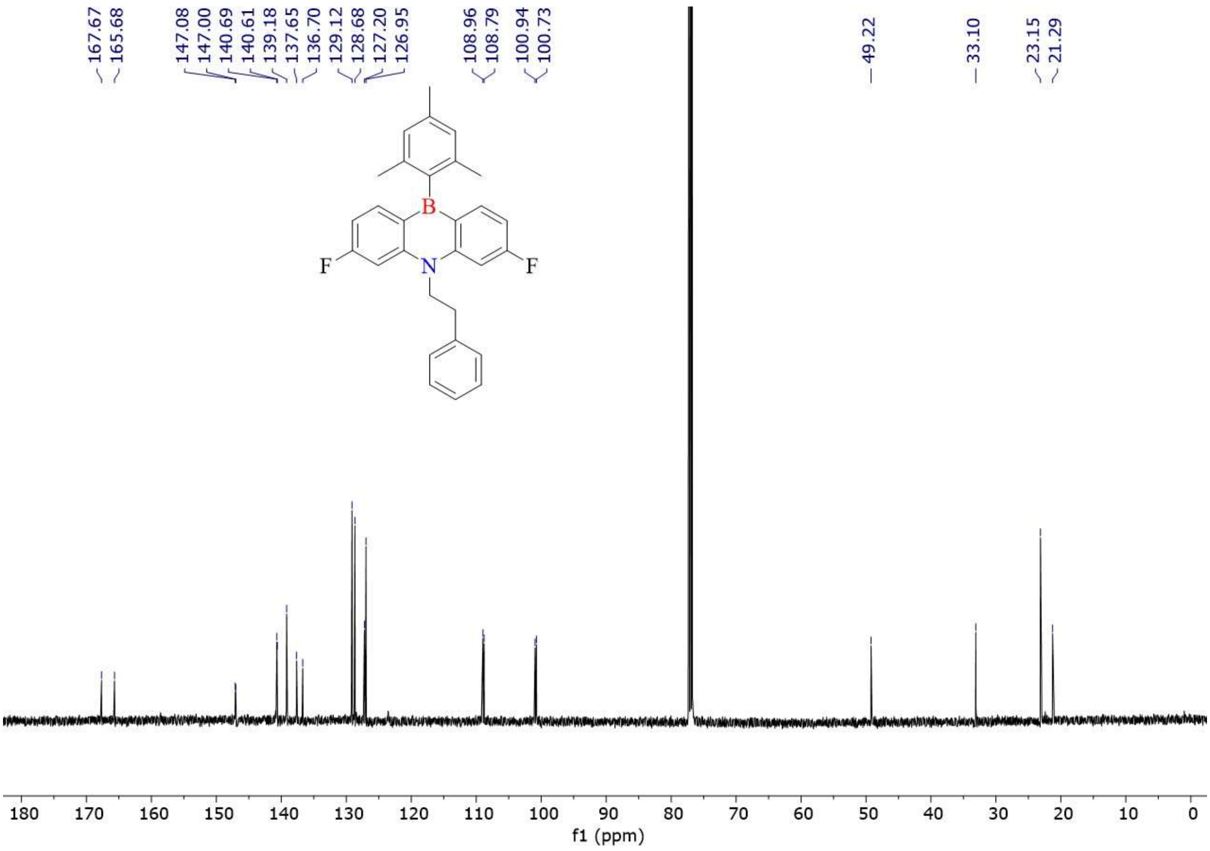

Figure **S65**. $^{13}C\{^{1}H\}$ NMR spectrum of compound **4i** in $CDCl_3$ (126 MHz).

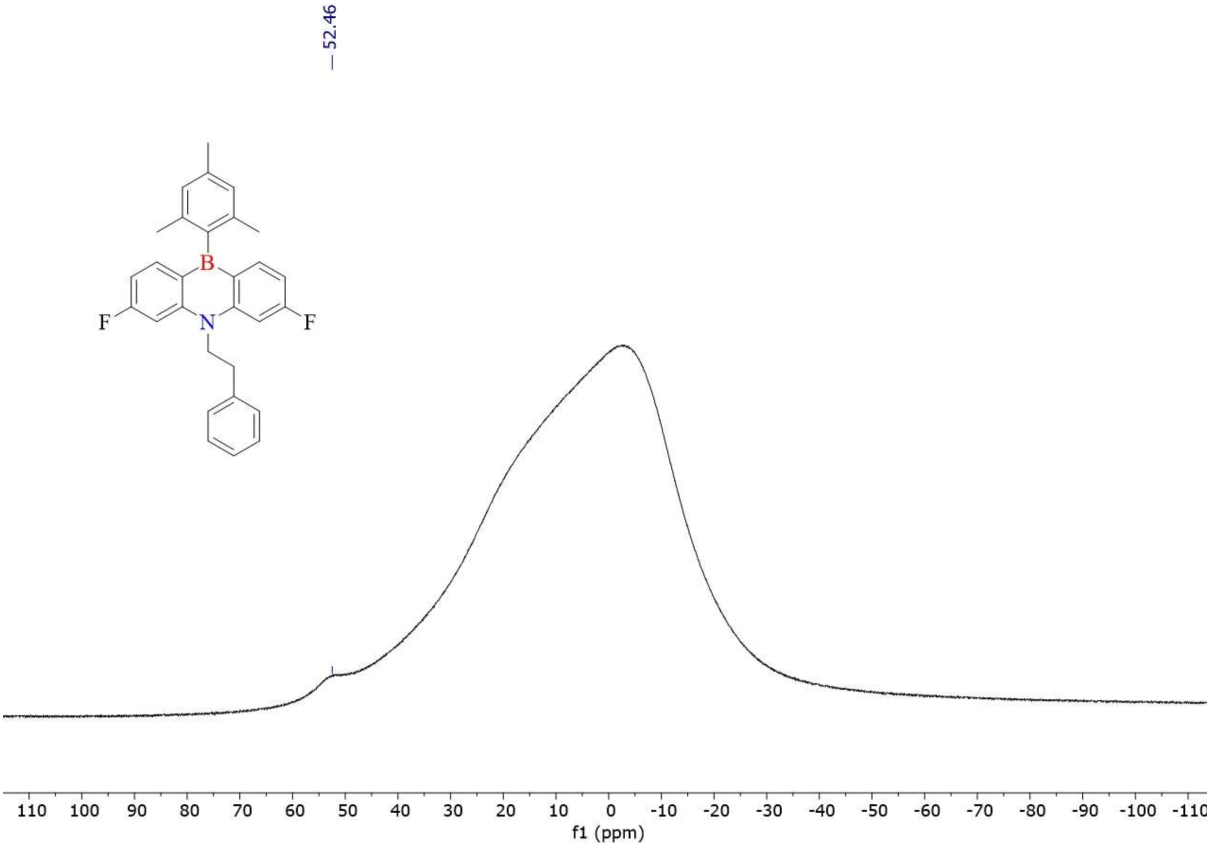

Figure **S66**. ^{11}B NMR spectrum of compound **4i** in $CDCl_3$ (160 MHz).

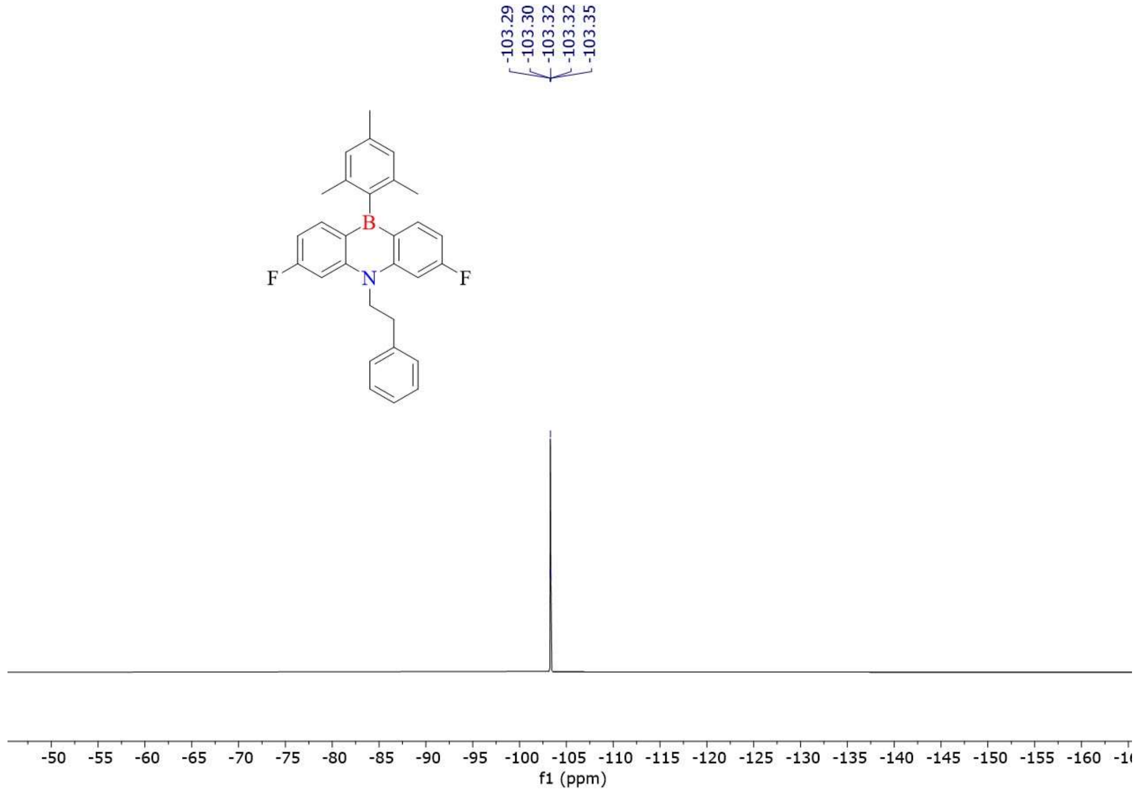

Figure **S67**. ^{19}F NMR spectrum of compound **4i** in $CDCl_3$ (471 MHz).

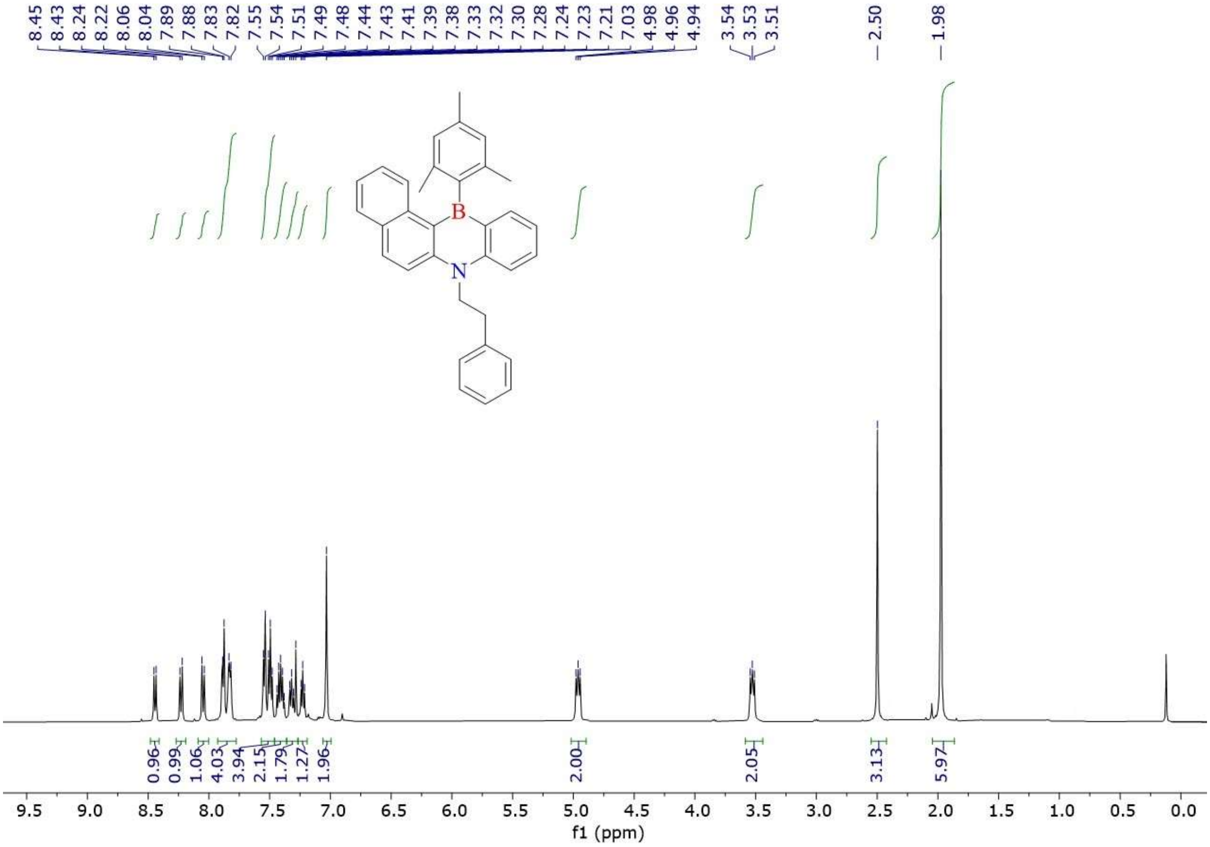

Figure **S68**. ^{1}H NMR spectrum of compound **4k-α** in $CDCl_3$ (500 MHz).

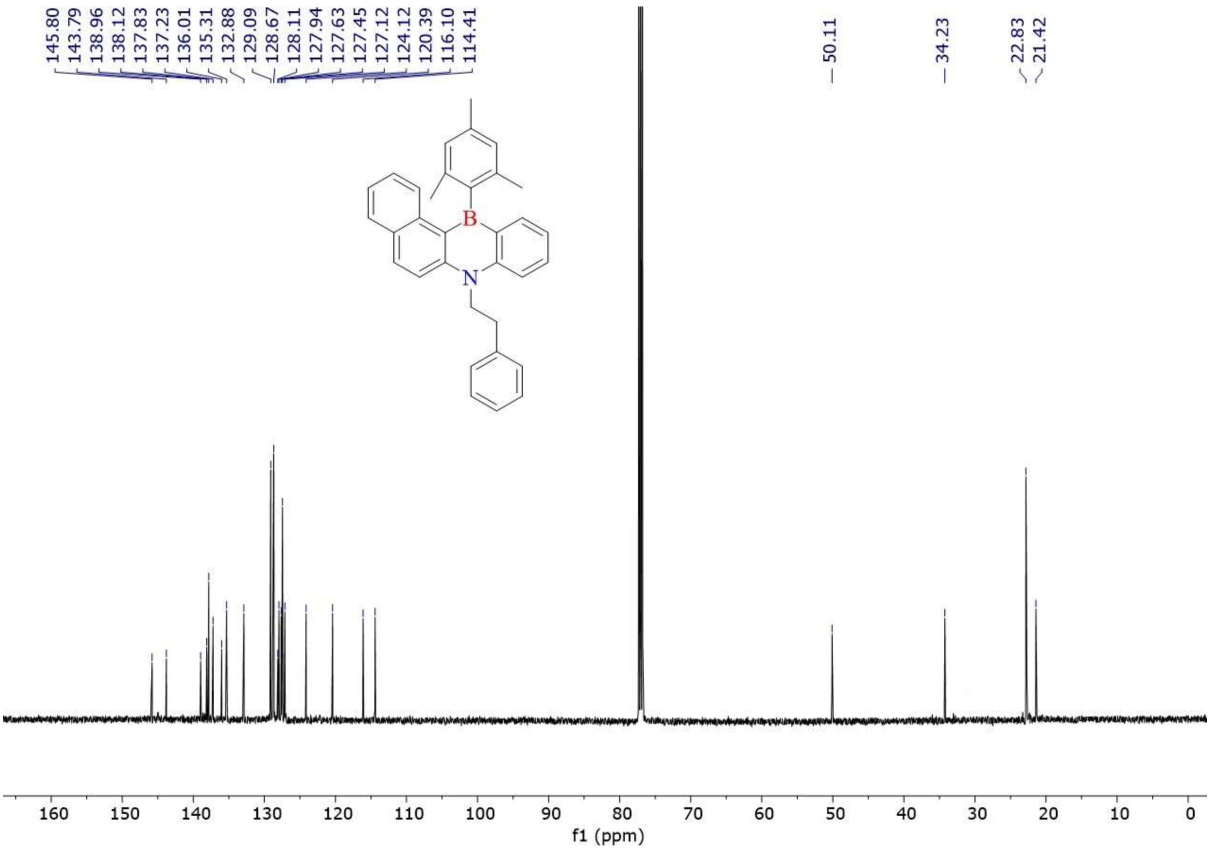

Figure **S69**. $^{13}C\{^{1}H\}$ NMR spectrum of compound **4k-α** in $CDCl_3$ (126 MHz).

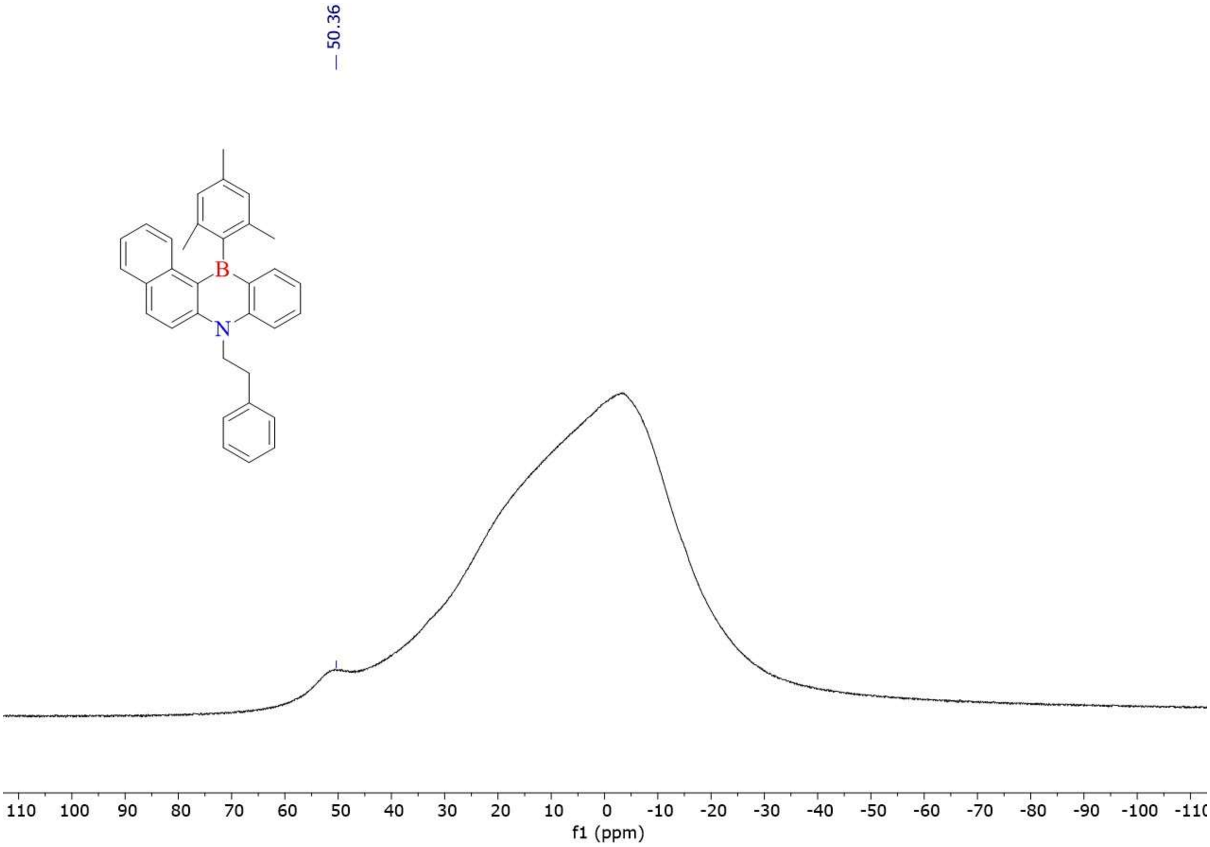

Figure **S70**. ^{11}B NMR spectrum of compound **4k-α** in $CDCl_3$ (160 MHz).

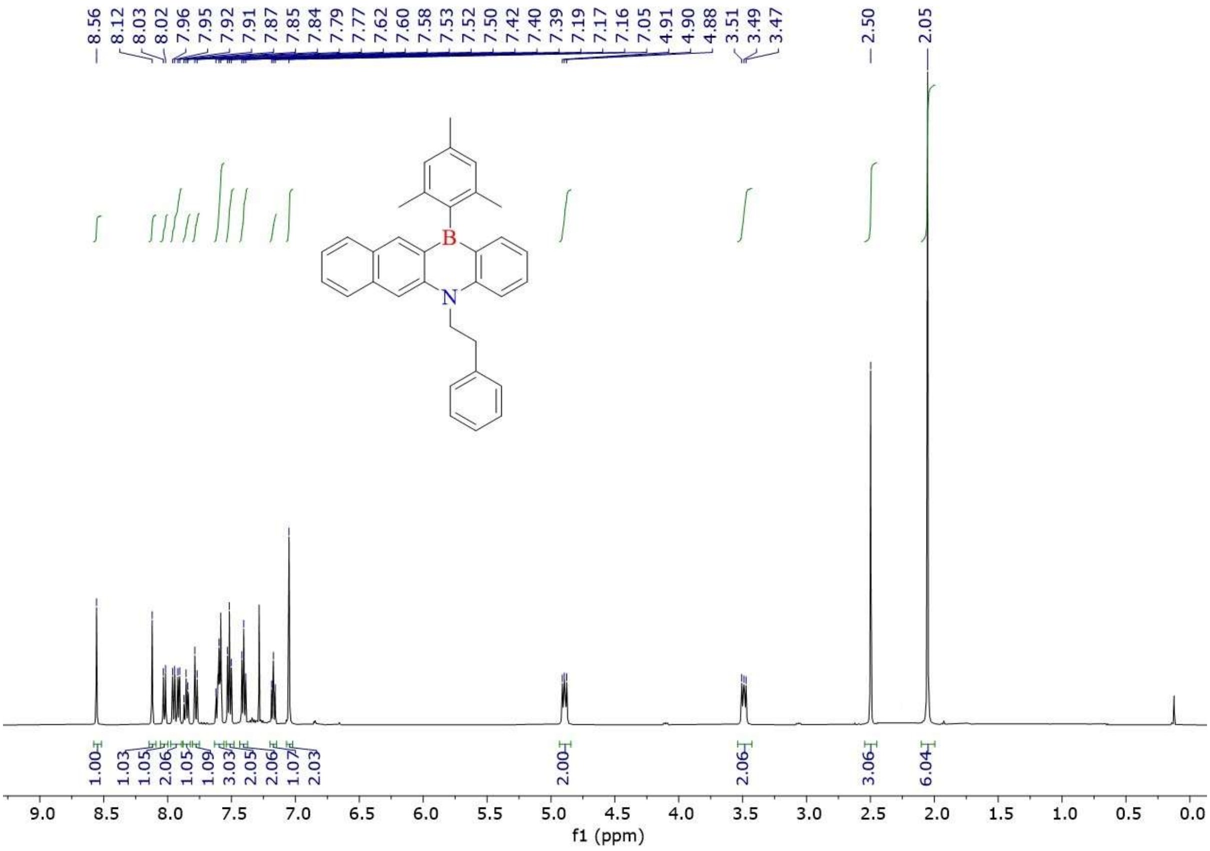

Figure **S71**. ^{1}H NMR spectrum of compound **4k-β** in $CDCl_3$ (500 MHz).

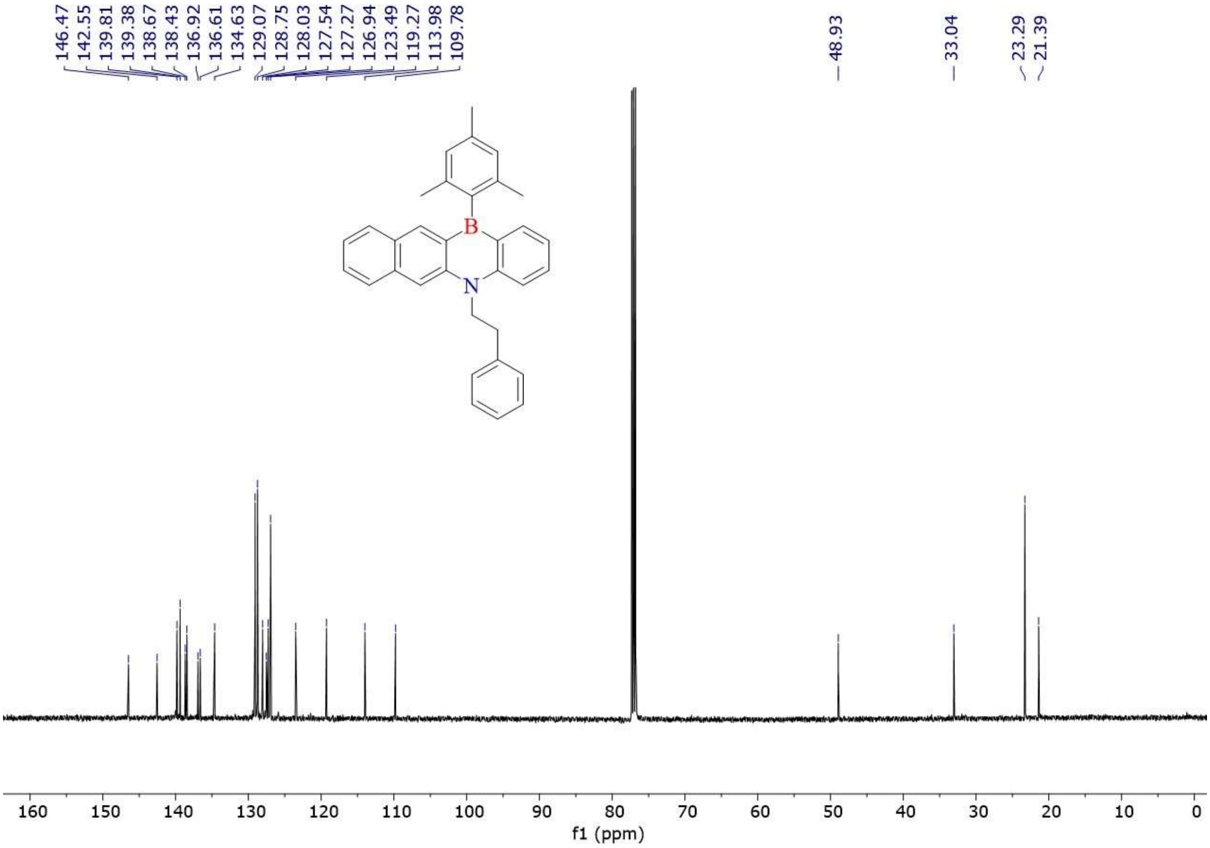

Figure **S72**. $^{13}C\{^{1}H\}$ NMR spectrum of compound **4k-β** in $CDCl_3$ (126 MHz).

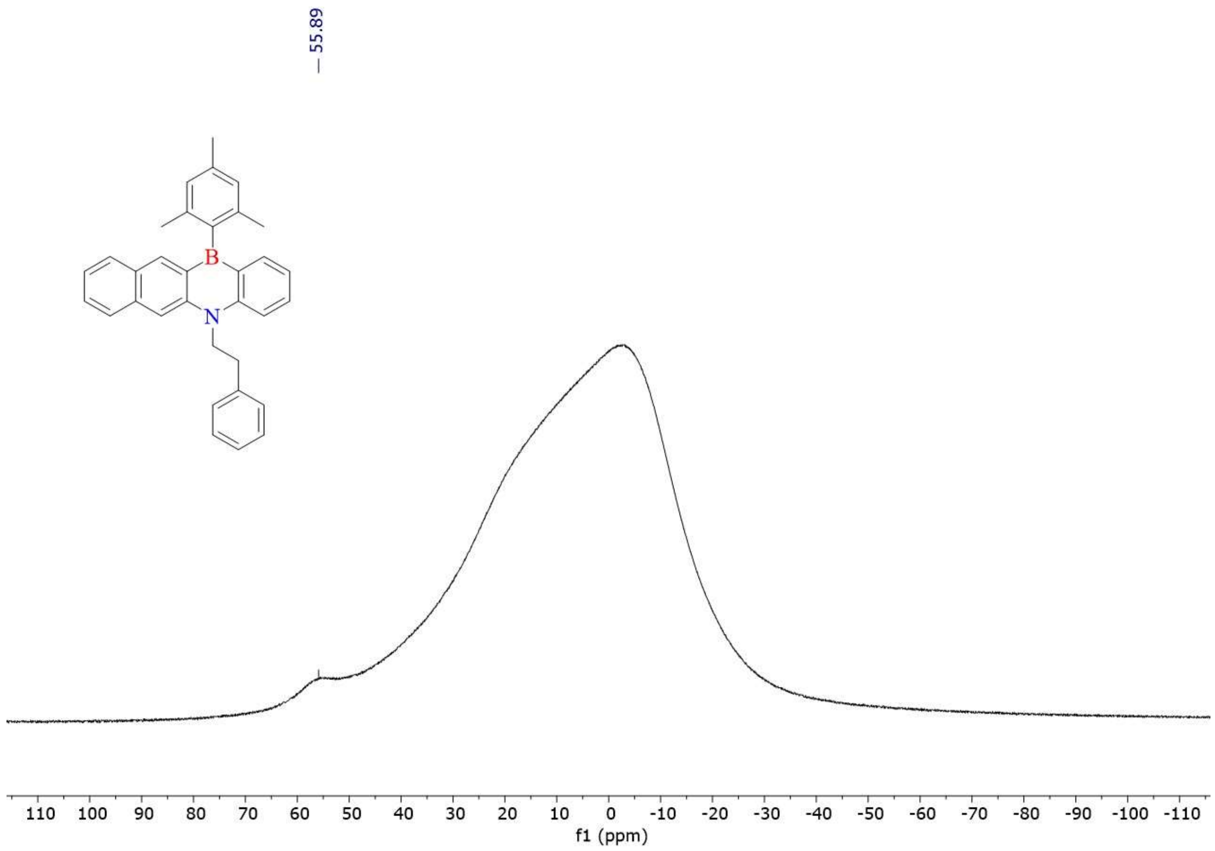

Figure **S73**. ^{11}B NMR spectrum of compound **4k-β** in $CDCl_3$ (160 MHz).

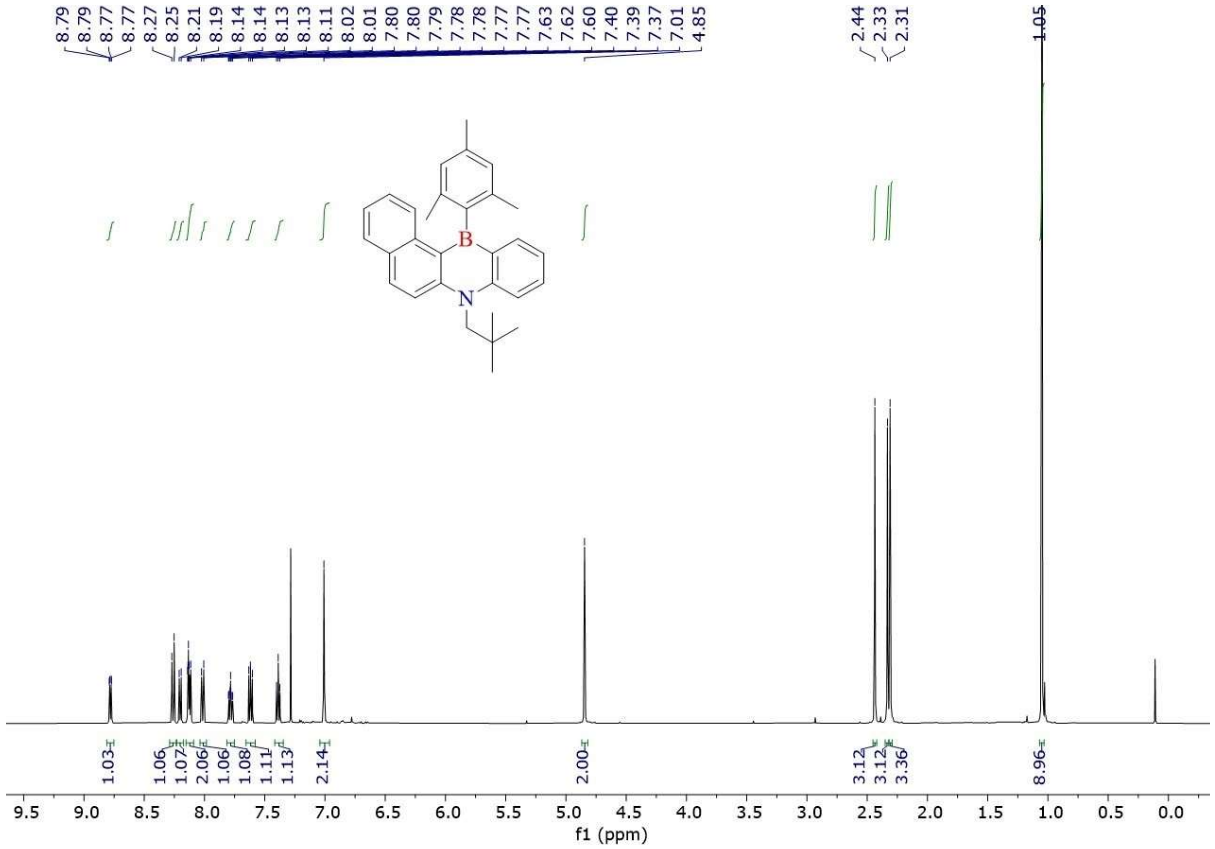

Figure **S74**. ^{1}H NMR spectrum of compound **4l** in $CDCl_3$ (500 MHz).

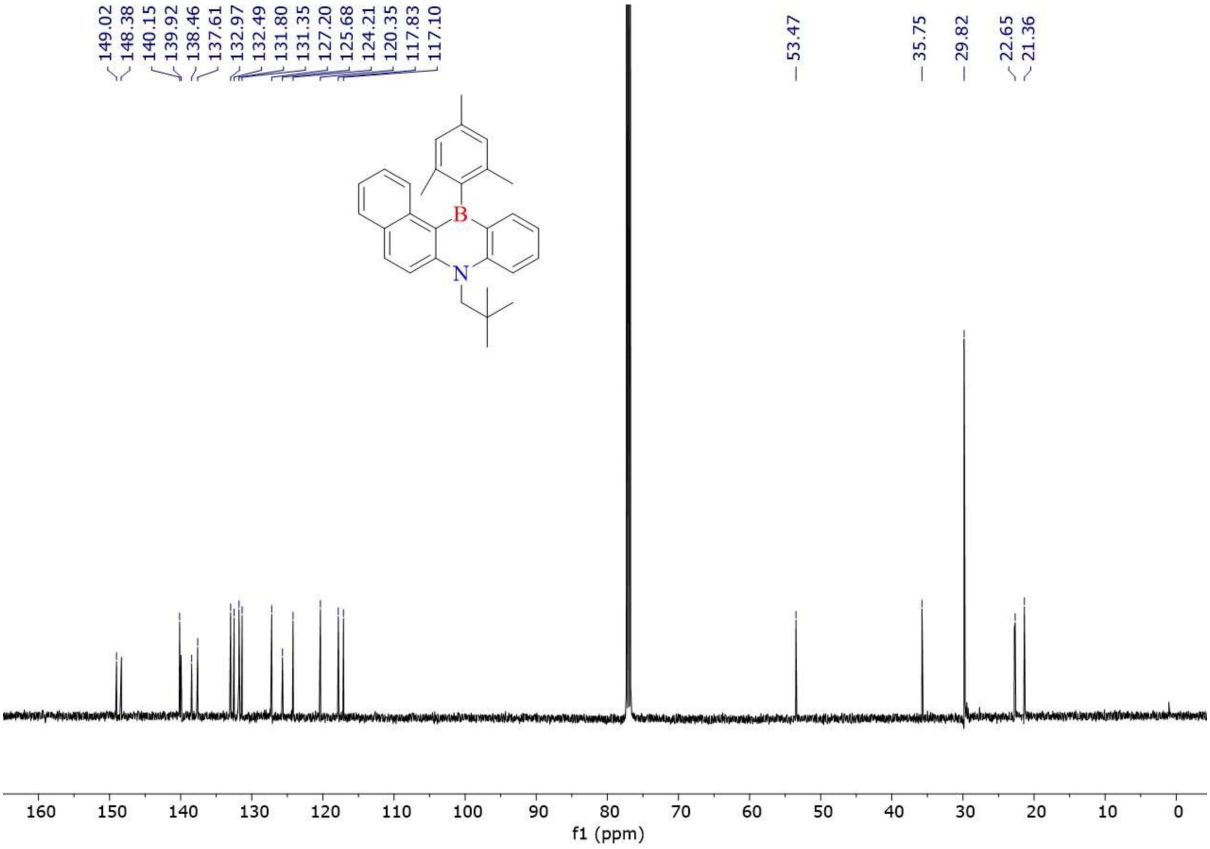

Figure **S75**. $^{13}C\{^{1}H\}$ NMR spectrum of compound **4l** in $CDCl_3$ (126 MHz).

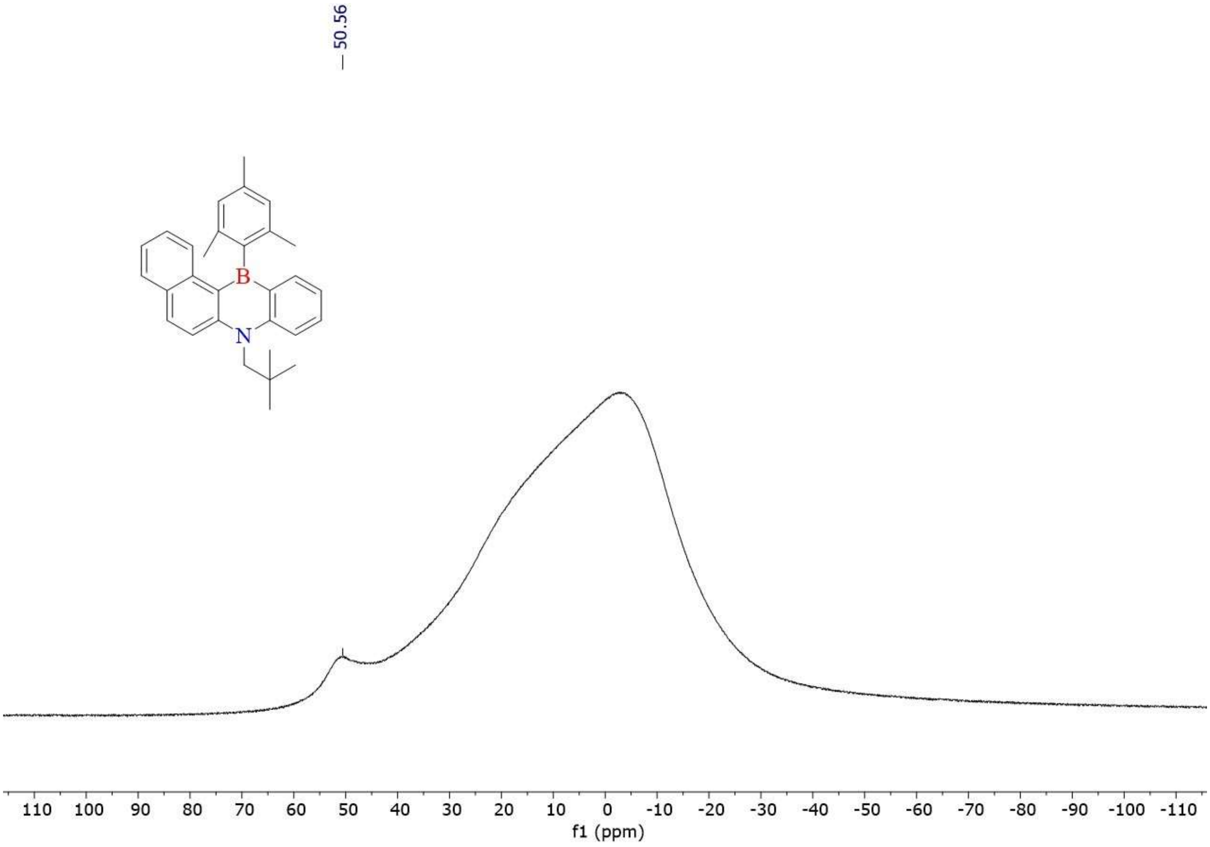

Figure **S76**. ^{11}B NMR spectrum of compound **4l** in $CDCl_3$ (160 MHz).

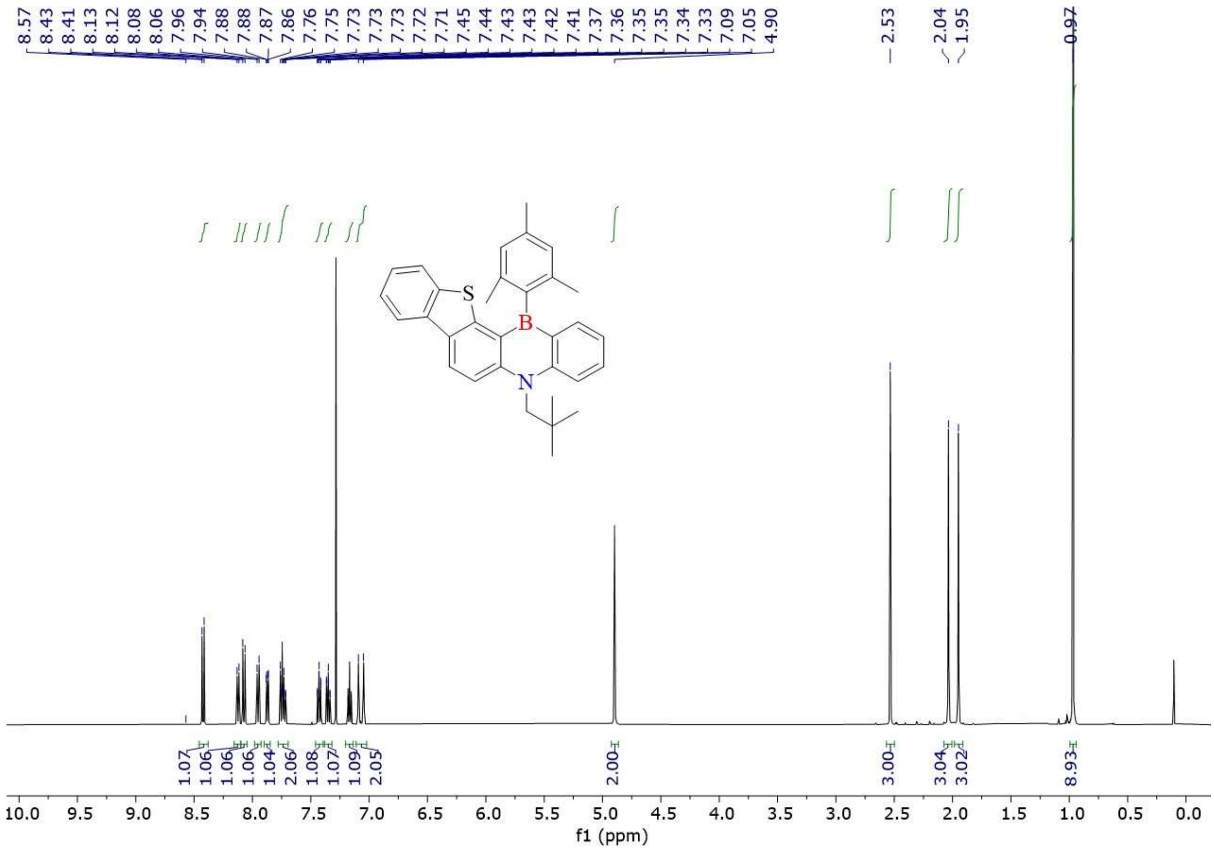

Figure **S77**. ^{1}H NMR spectrum of compound **4m-α** in $CDCl_3$ (500 MHz).

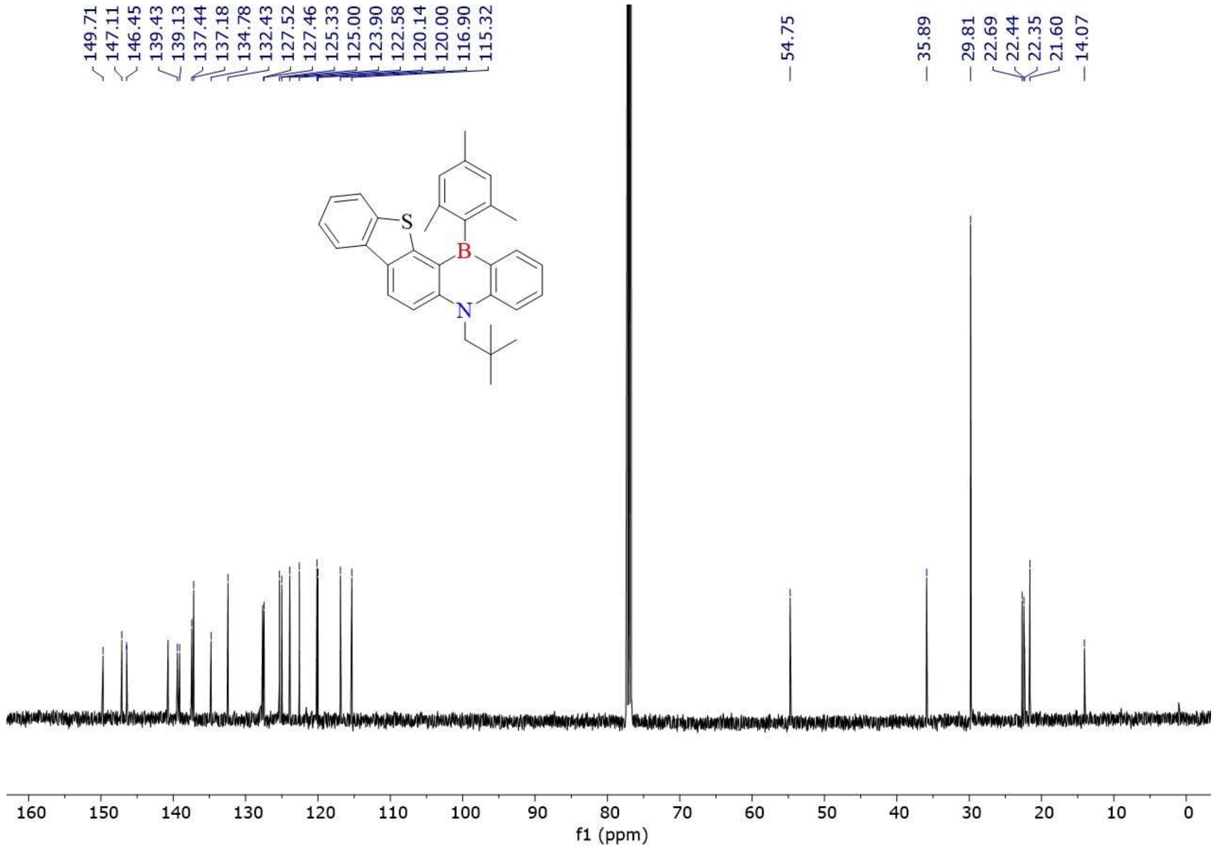

Figure **S78**. ^{13}C{^{1}H} NMR spectrum of compound **4m-α** in $CDCl_3$ (126 MHz).

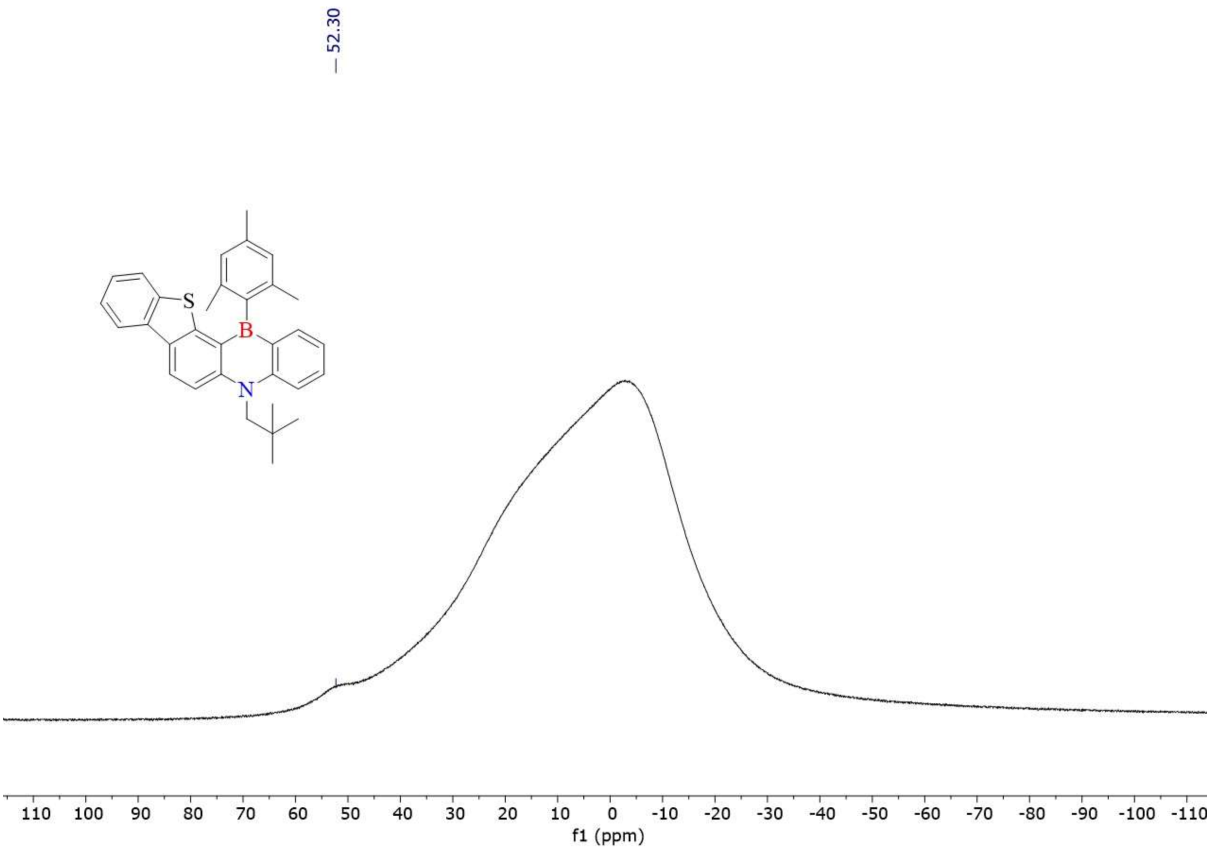

Figure **S79**. ^{11}B NMR spectrum of compound **4m-α** in $CDCl_3$ (160 MHz).

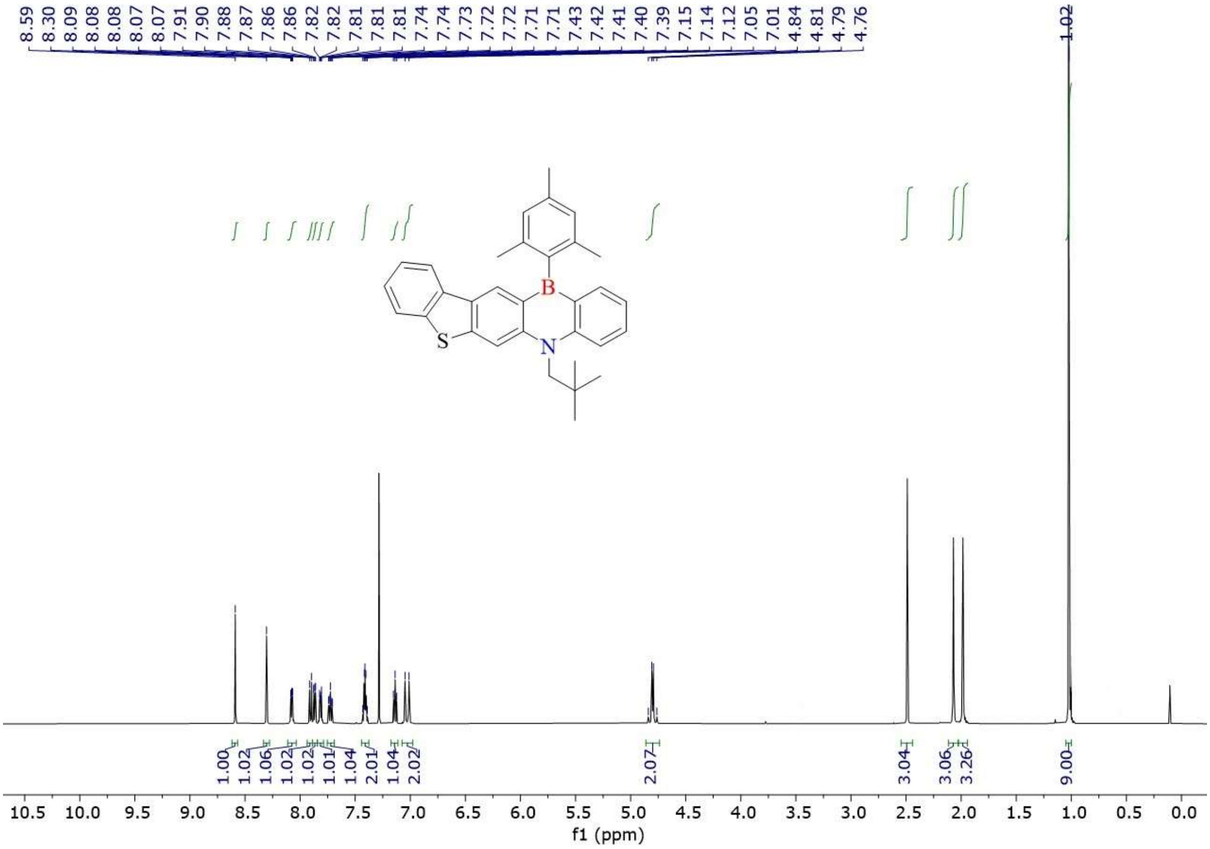

Figure **S80**. ^{1}H NMR spectrum of compound **4m-β** in $CDCl_3$ (500 MHz).

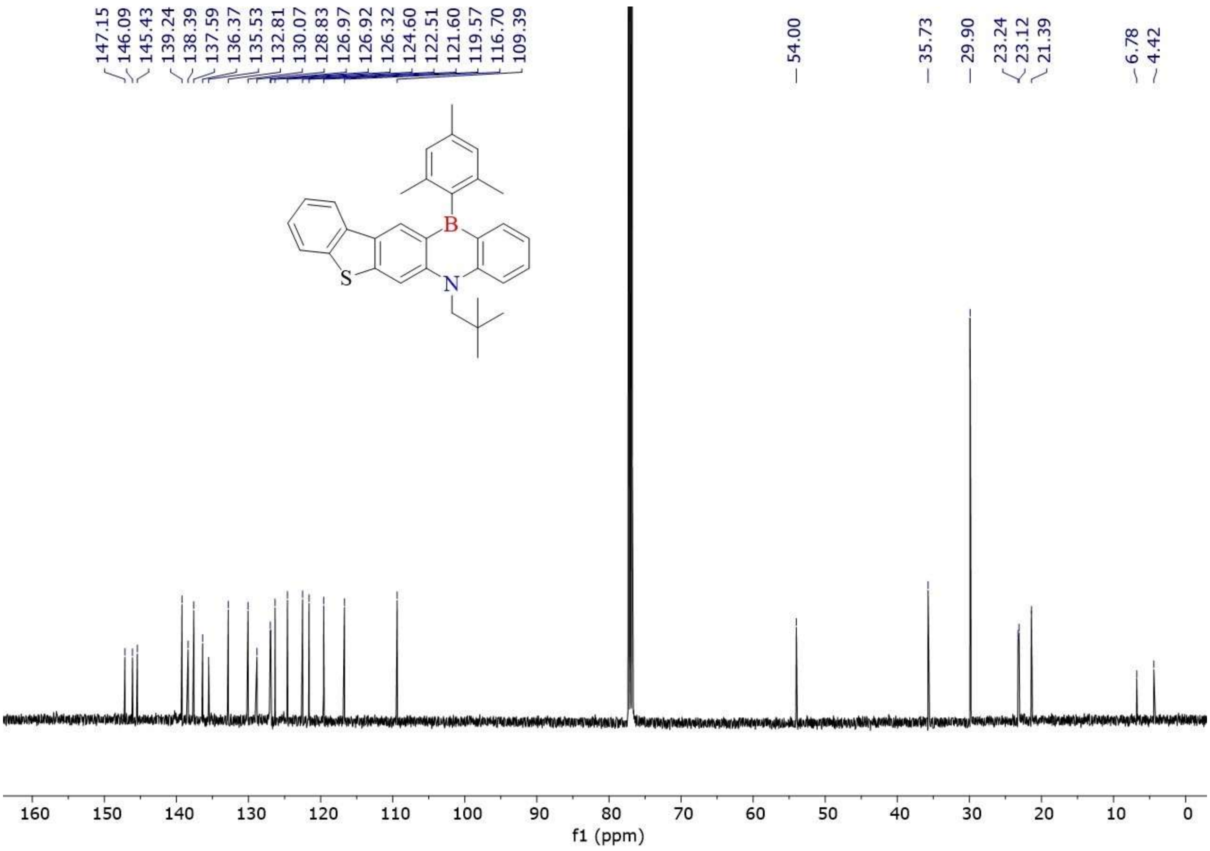

Figure **S81**. $^{13}C\{^1H\}$ NMR spectrum of compound **4m-β** in $CDCl_3$ (126 MHz).

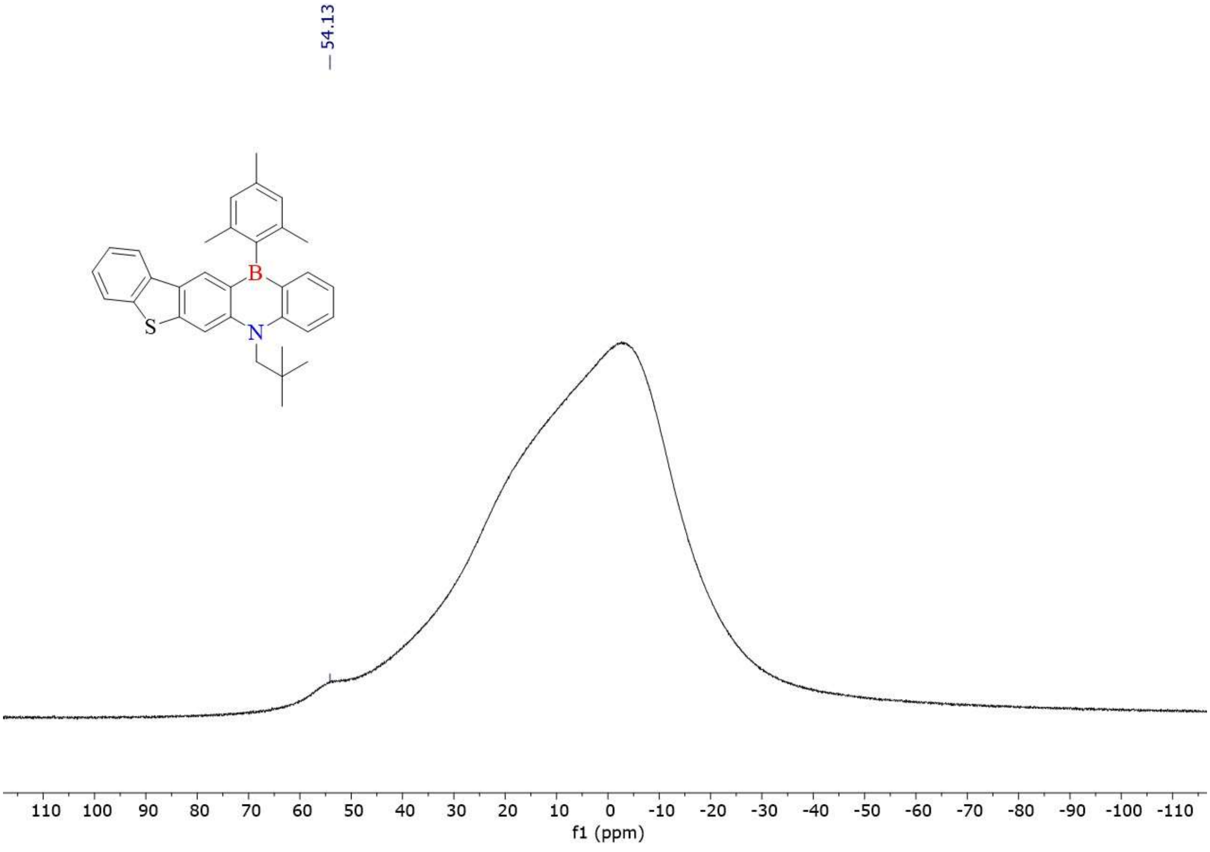

Figure **S82**. ^{11}B NMR spectrum of compound **4m-β** in $CDCl_3$ (160 MHz).

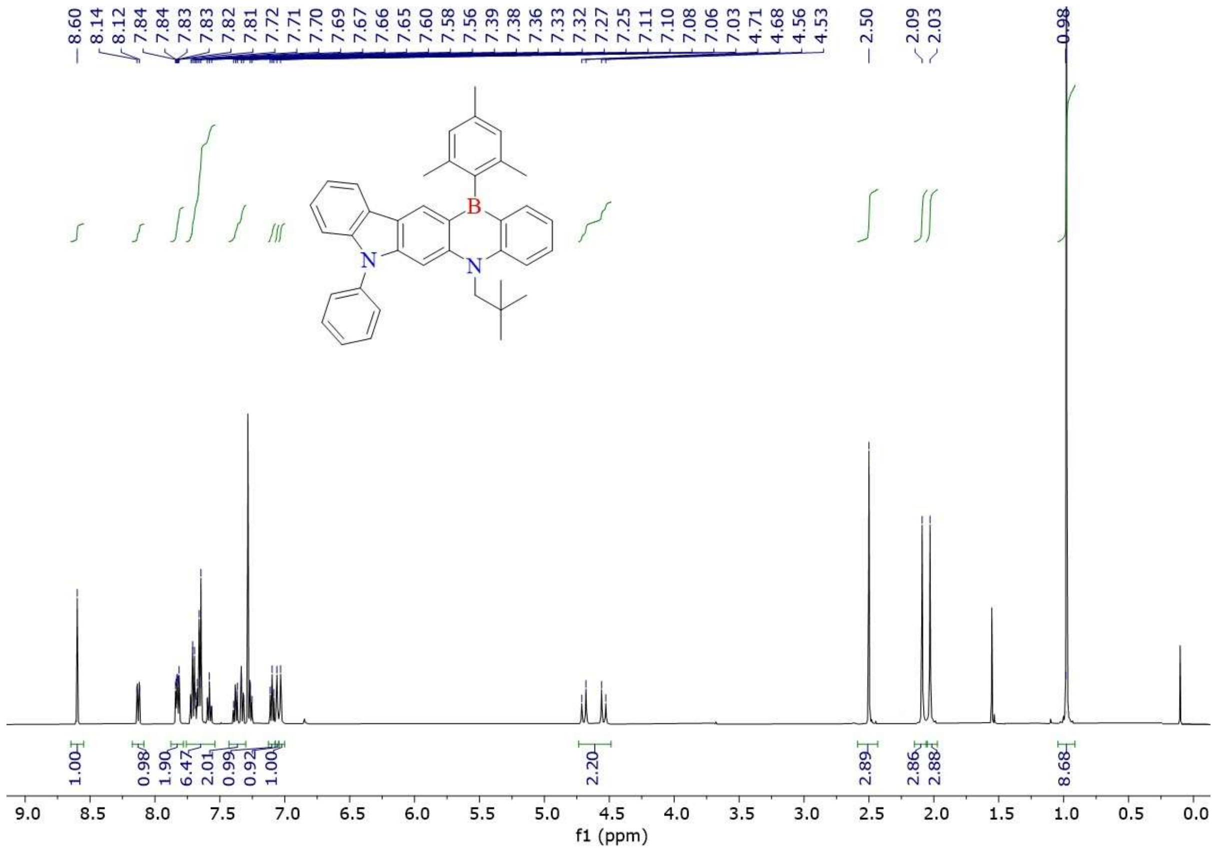

Figure **S83**. ^{1}H NMR spectrum of compound **4n** in $CDCl_3$ (500 MHz).

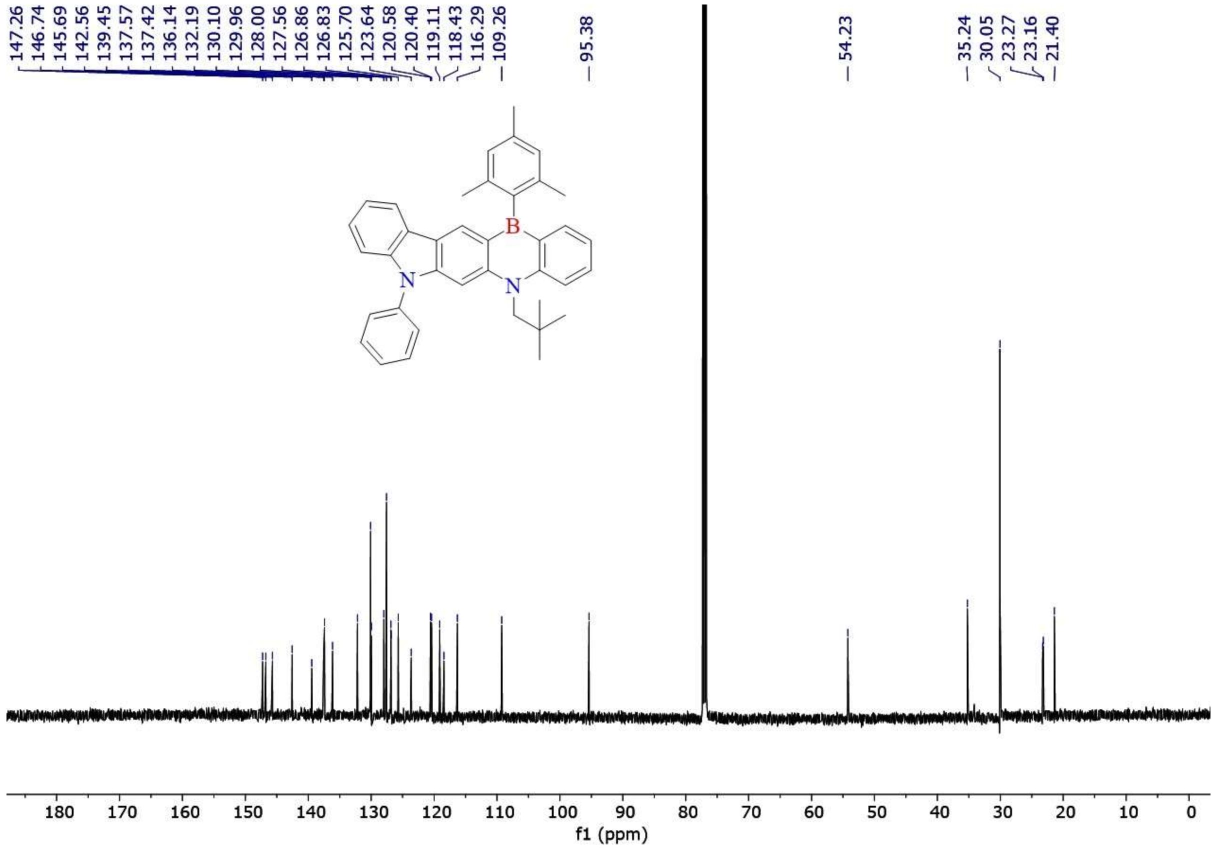

Figure **S84**. $^{13}C\{^1H\}$ NMR spectrum of compound **4n** in $CDCl_3$ (126 MHz).

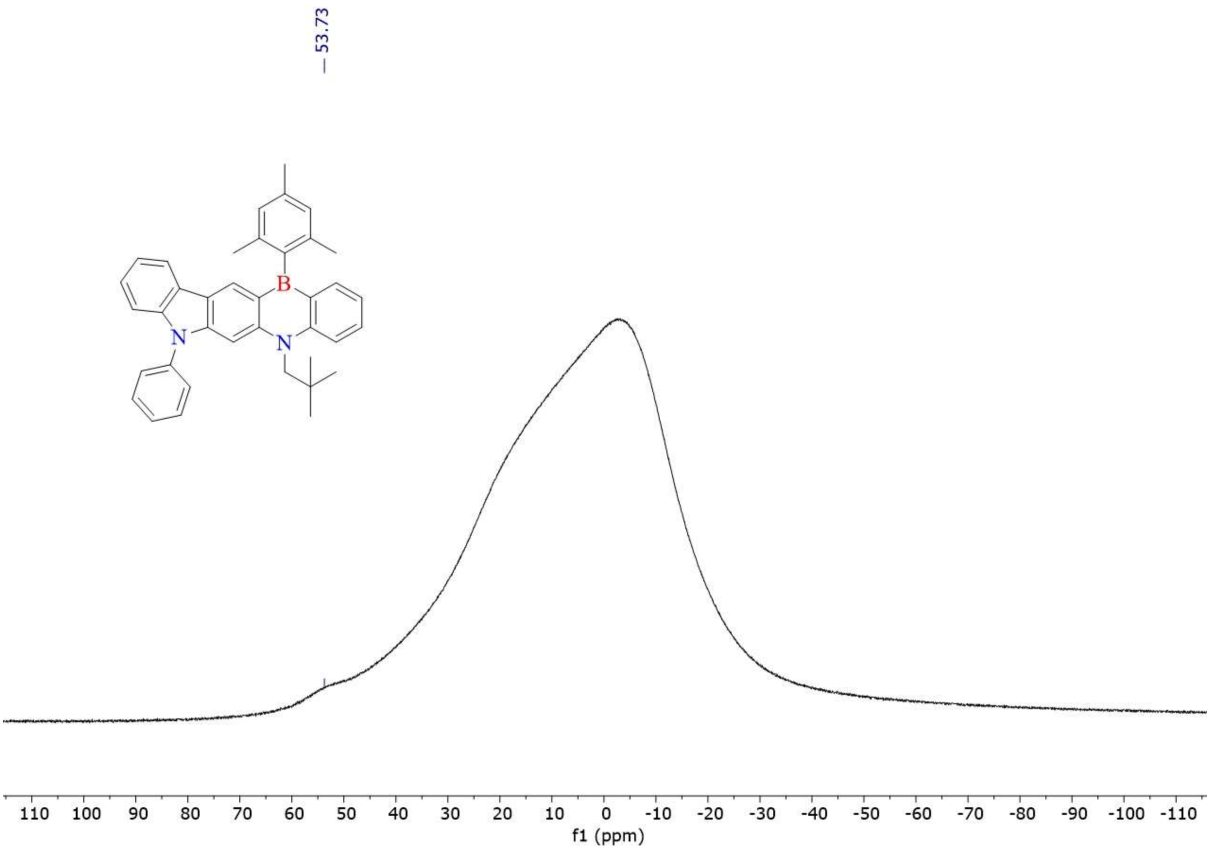

Figure **S85**. ^{11}B NMR spectrum of compound **4n** in $CDCl_3$ (160 MHz).

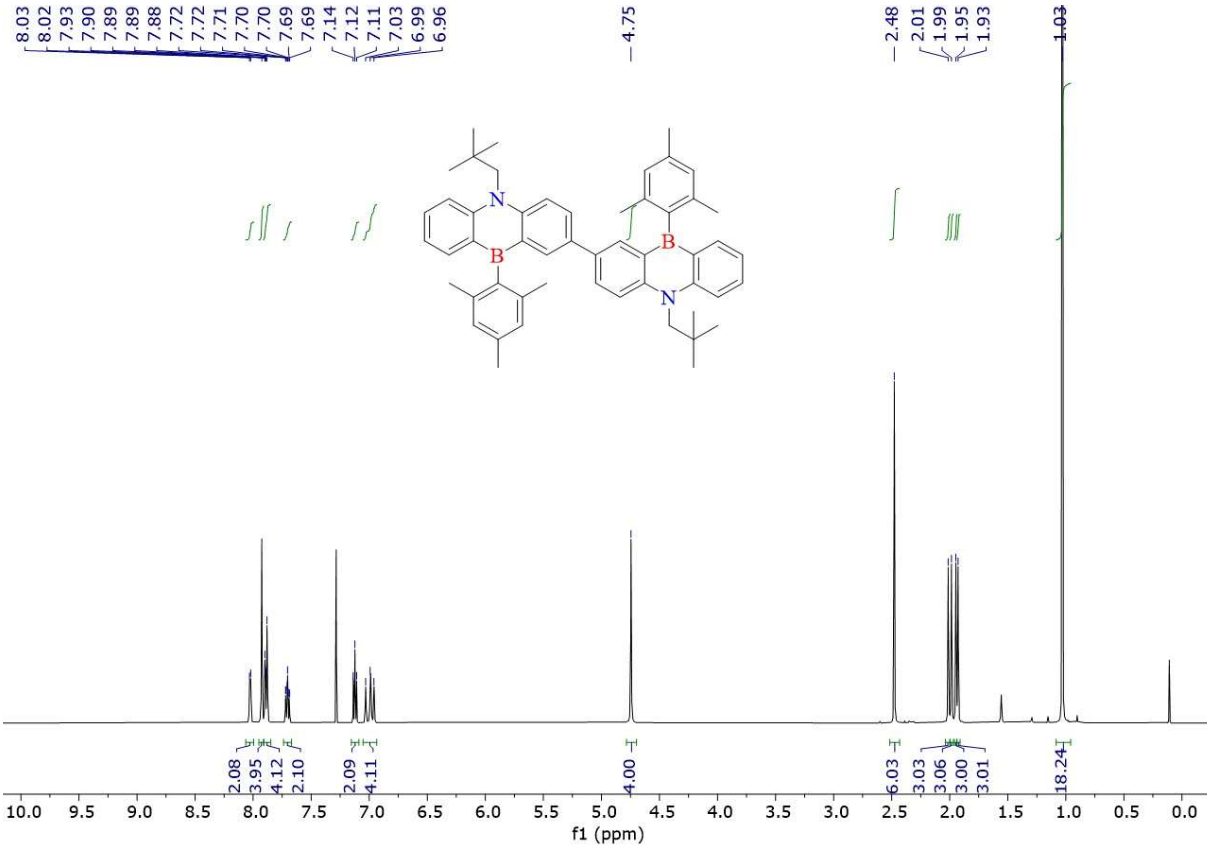

Figure **S86**. ^{1}H NMR spectrum of compound **4q** in $CDCl_3$ (500 MHz).

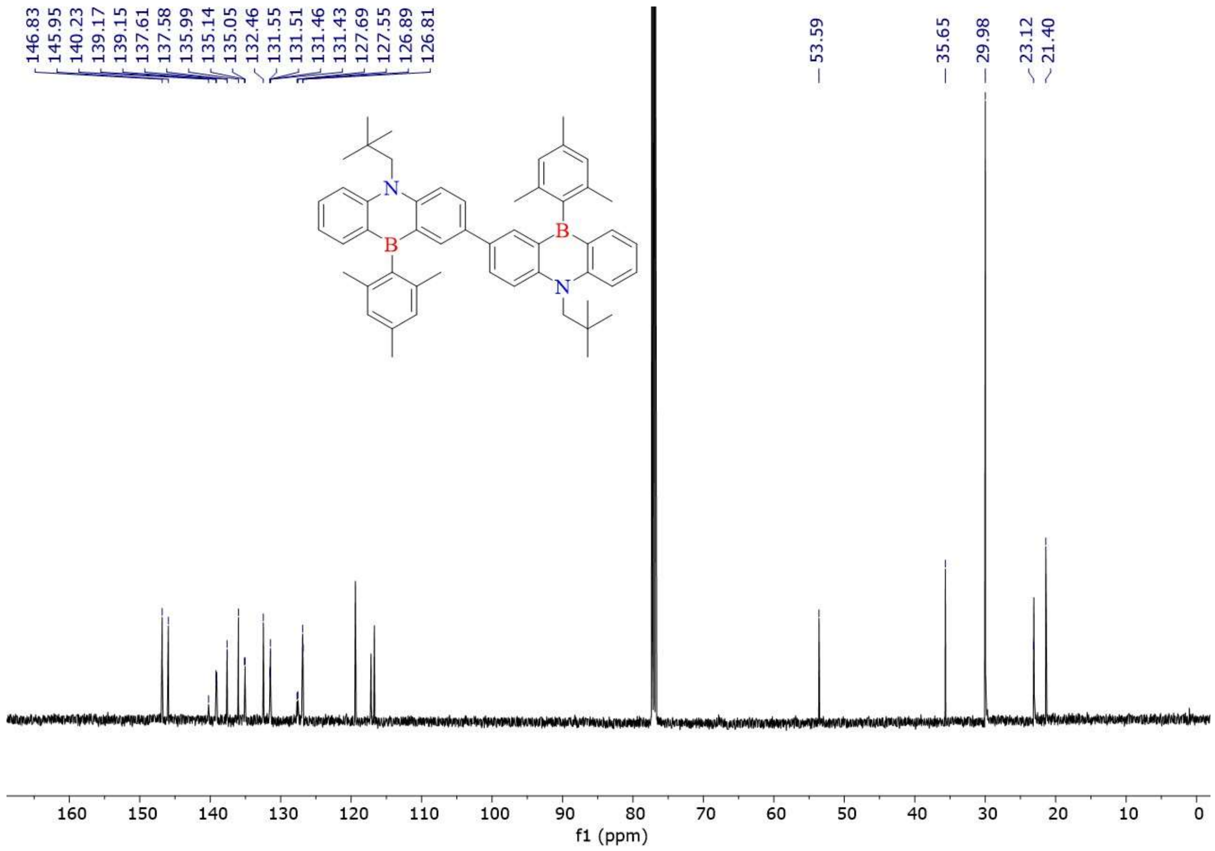

Figure **S87**. $^{13}C\{^1H\}$ NMR spectrum of compound **4q** in $CDCl_3$ (126 MHz).

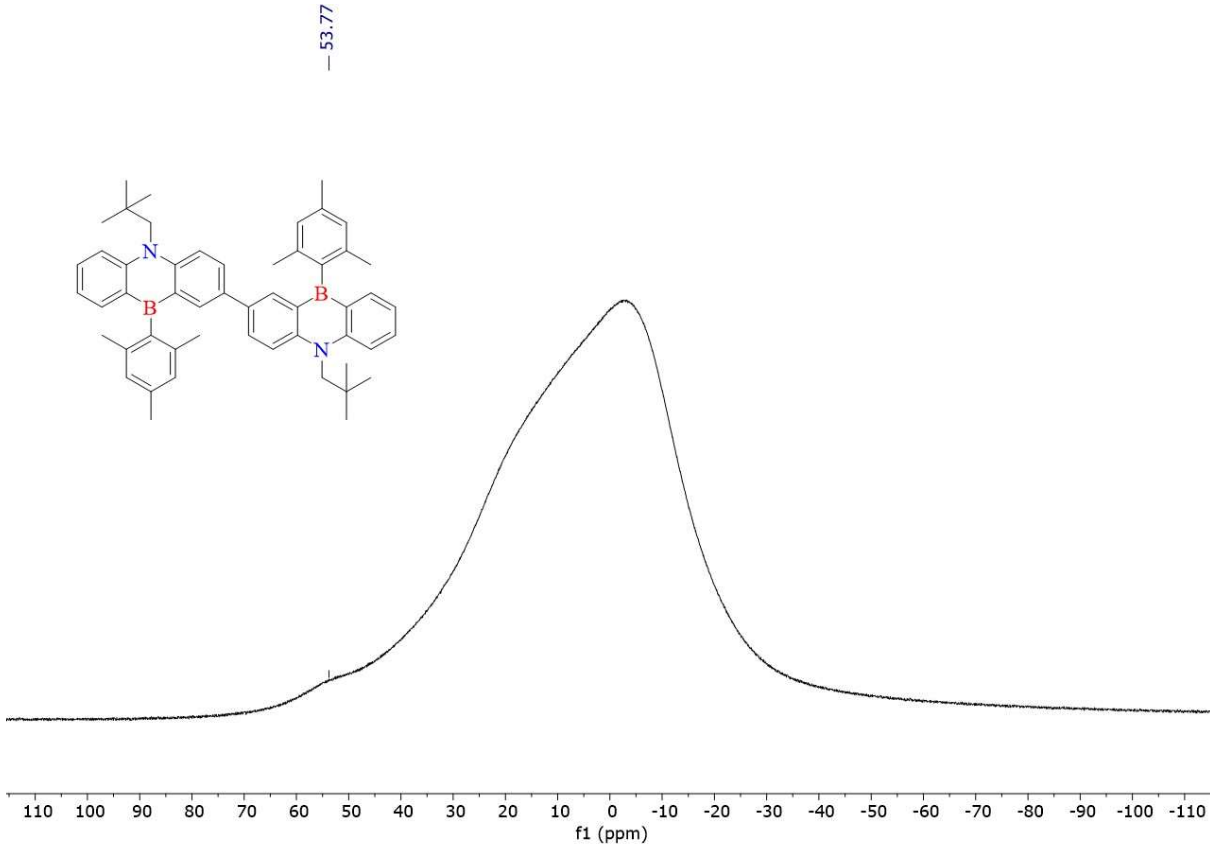

Figure **S88**. ^{11}B NMR spectrum of compound **4q** in $CDCl_3$ (160 MHz).

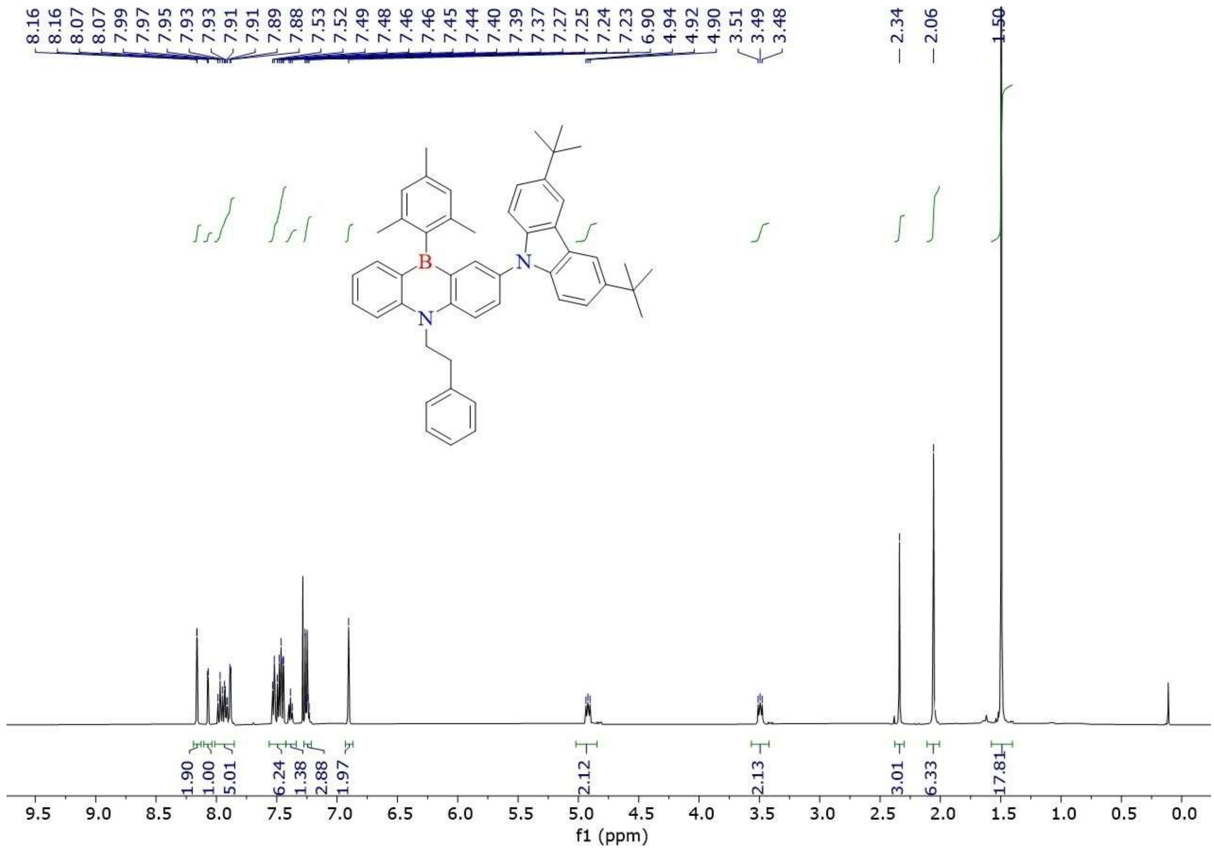

Figure **S89**. ^{1}H NMR spectrum of compound **5** in $CDCl_3$ (500 MHz).

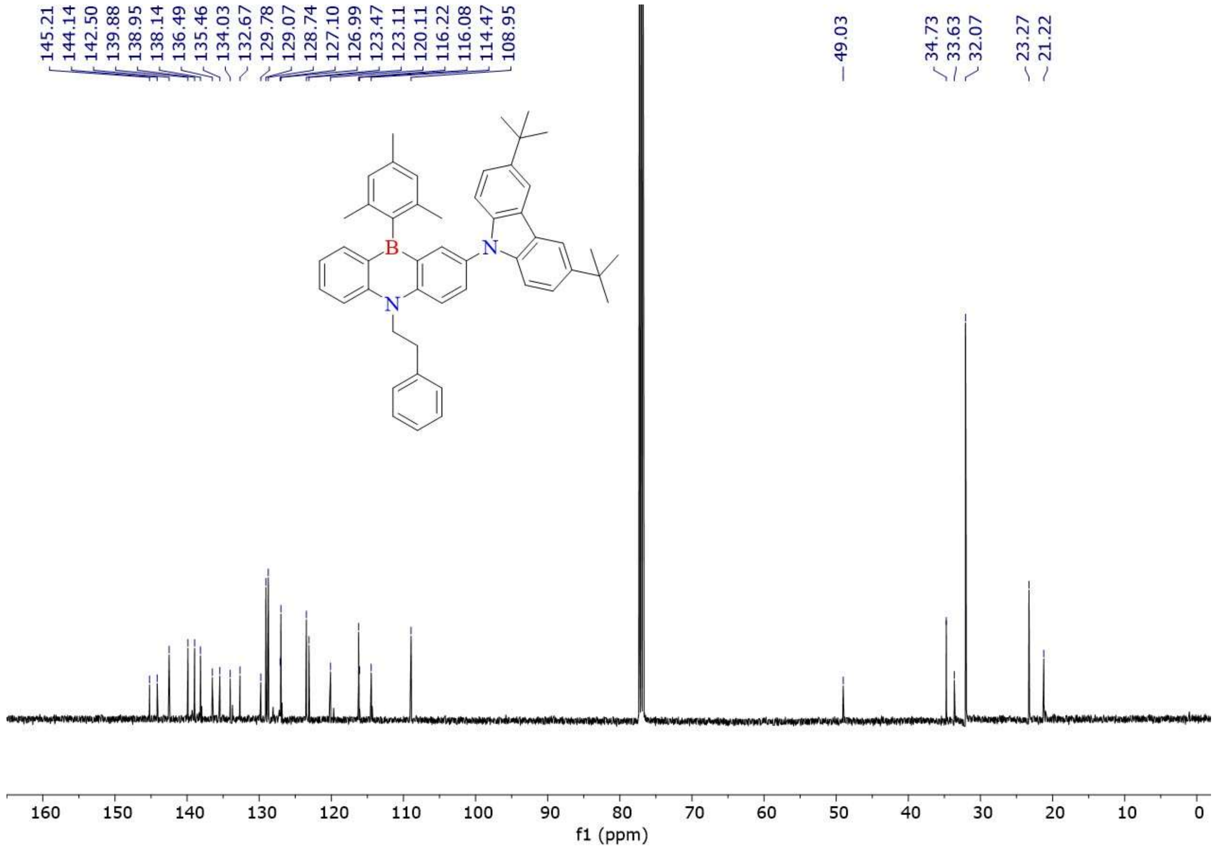

Figure **S90**. $^{13}C\{^1H\}$ NMR spectrum of compound **5** in $CDCl_3$ (126 MHz).

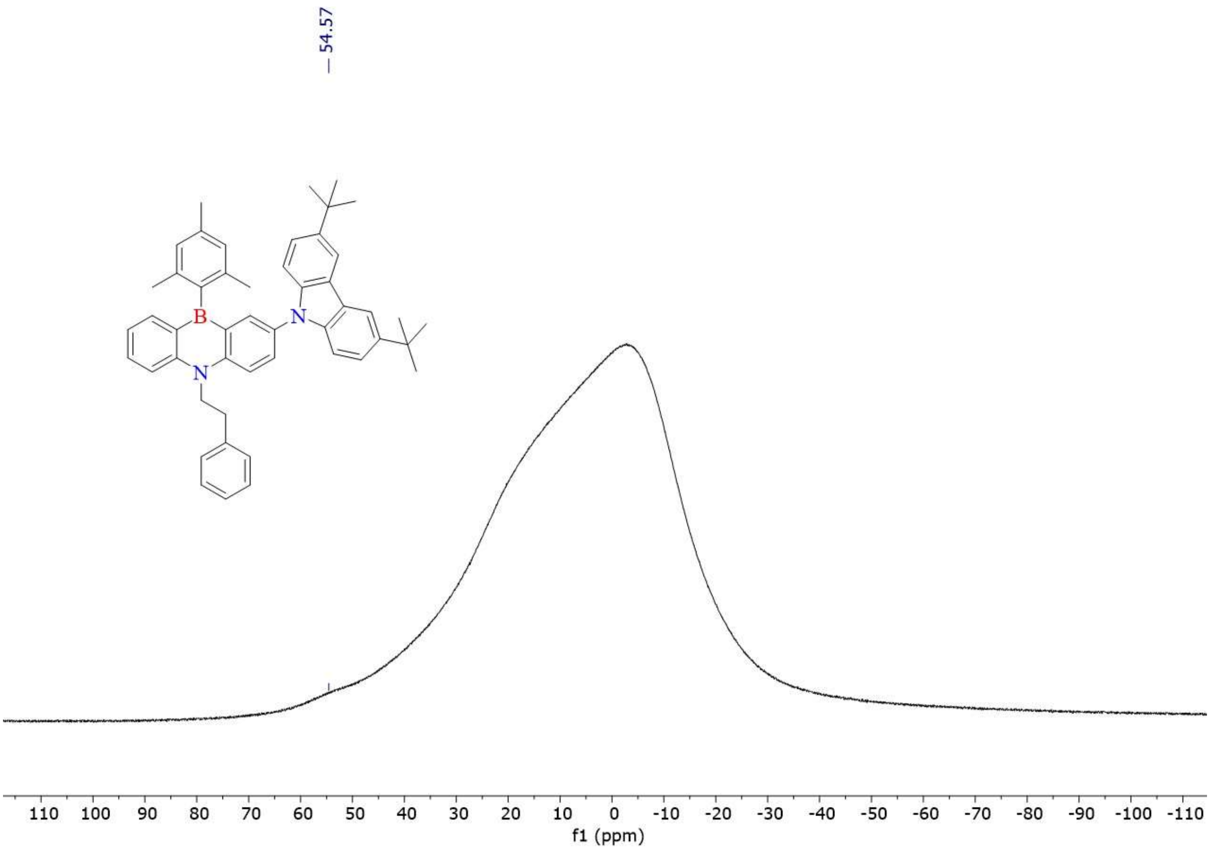

Figure **S91**. ^{11}B NMR spectrum of compound **5** in $CDCl_3$ (160 MHz).

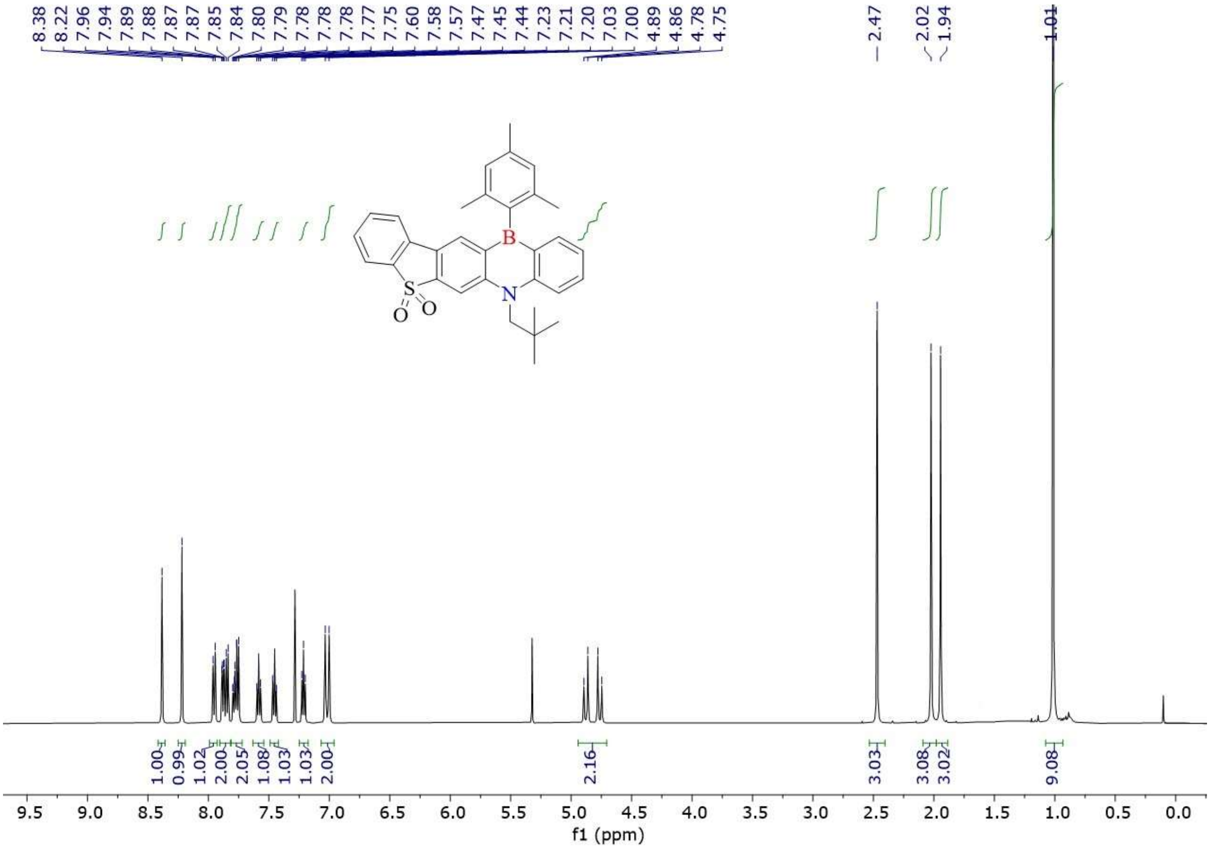

Figure **S92**. ^{1}H NMR spectrum of compound **6** in $CDCl_3$ (500 MHz).

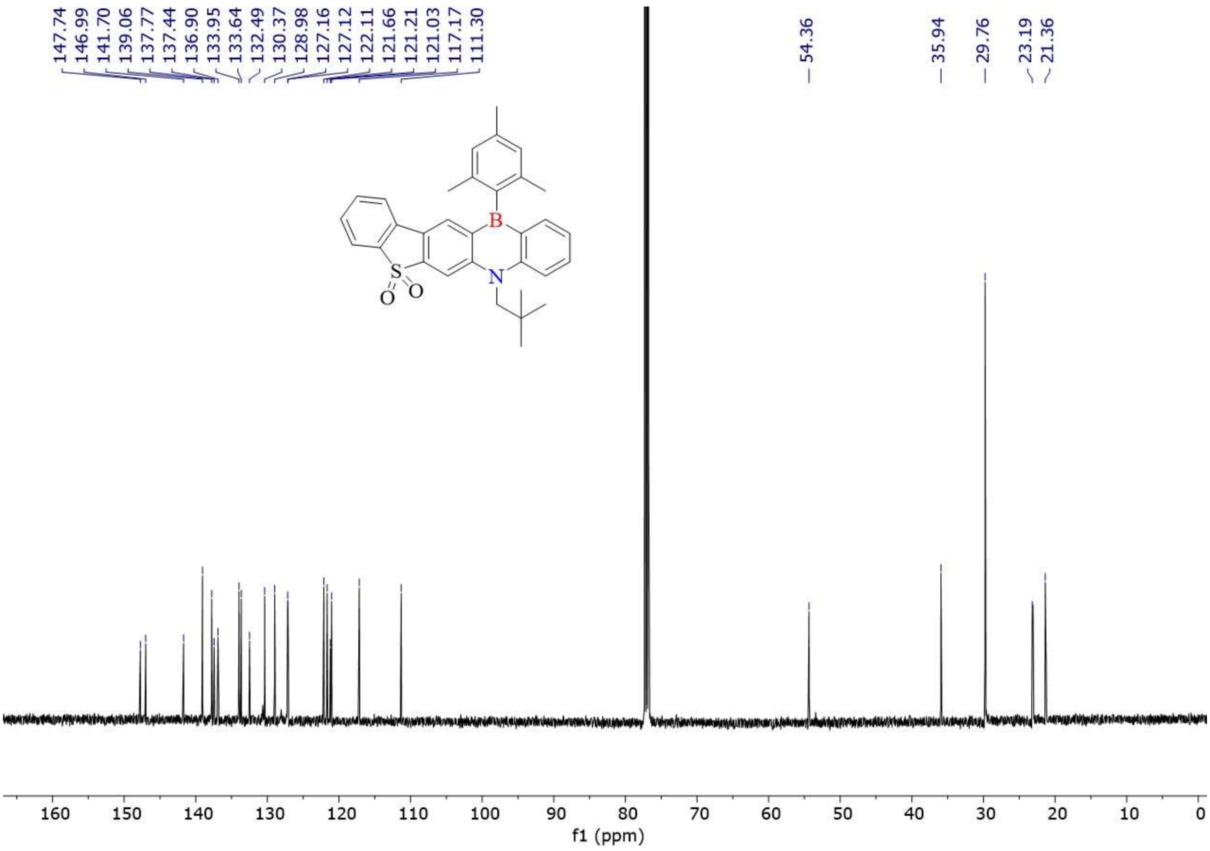

Figure **S93**. $^{13}C\{^{1}H\}$ NMR spectrum of compound 6 in $CDCl_3$ (126 MHz).

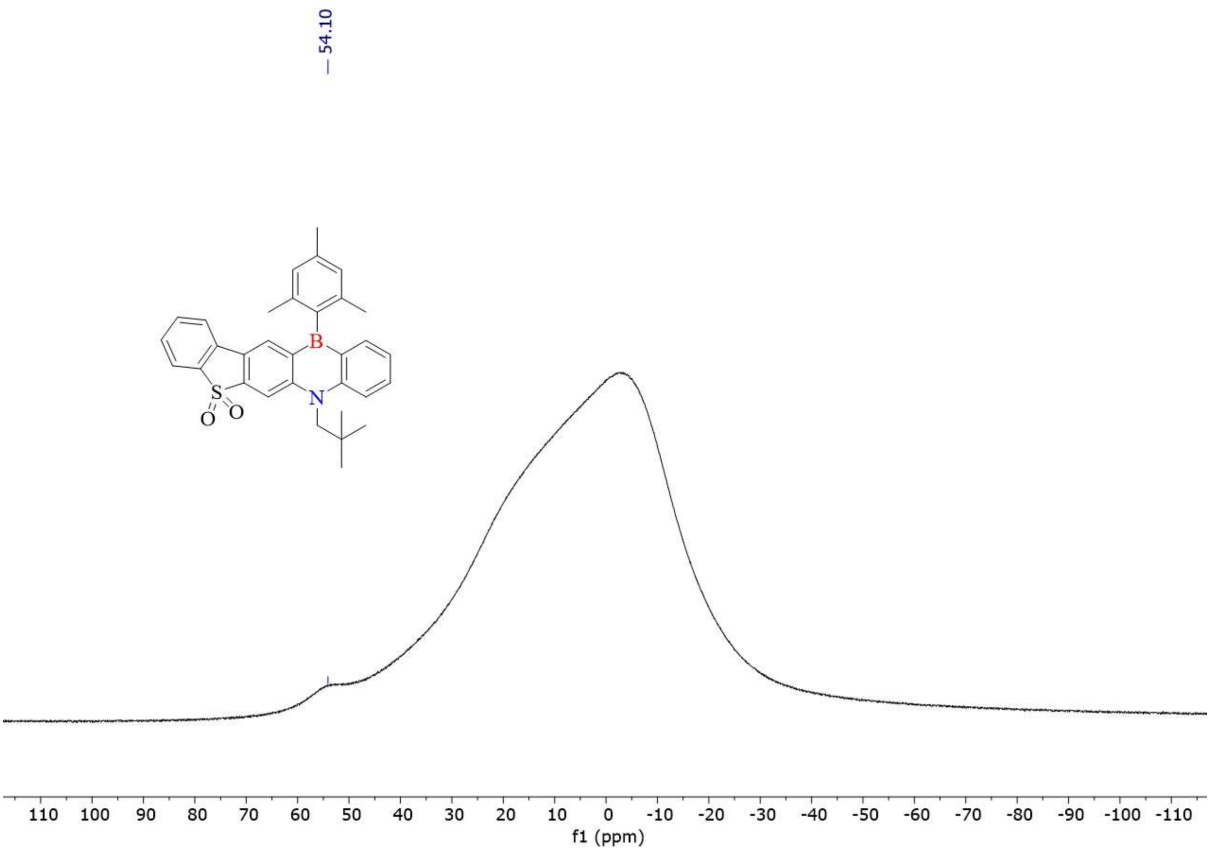

Figure **S94**. ^{11}B NMR spectrum of compound **6** in $CDCl_3$ (160 MHz).

S4. DFT Calculations

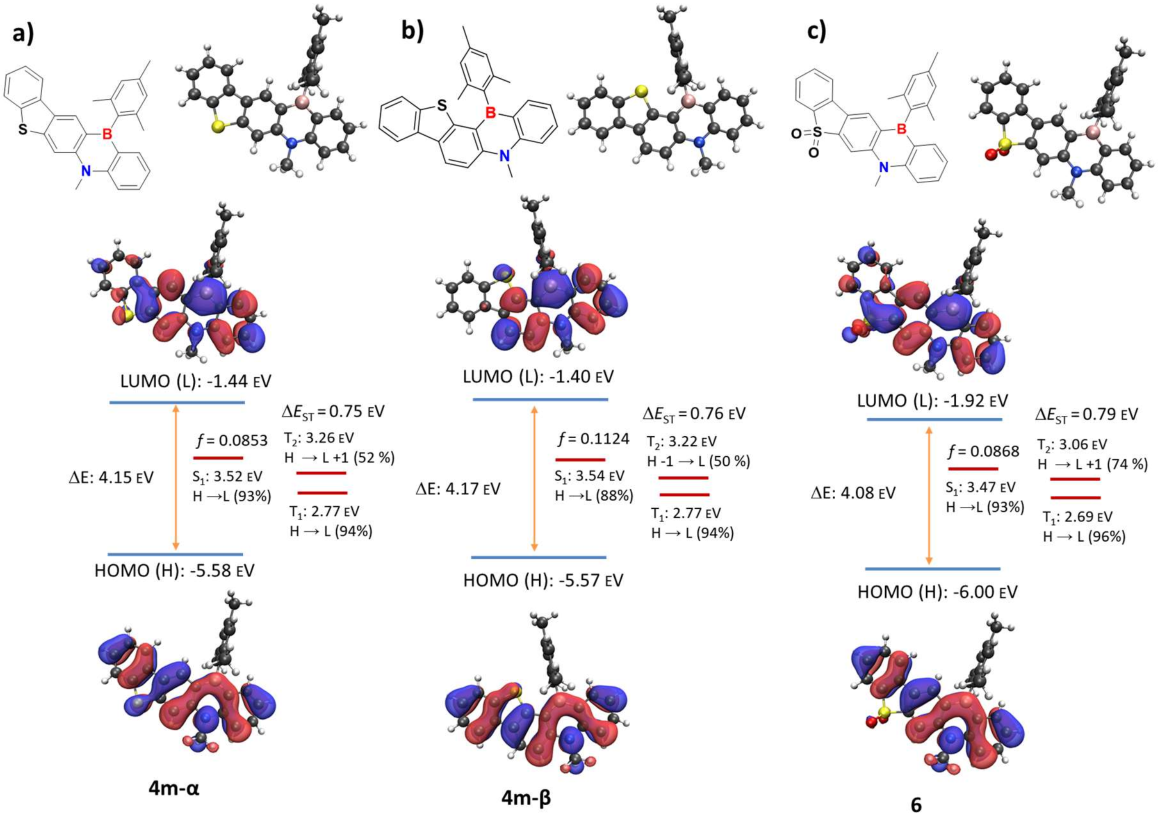

Figure **S95.** DFT-optimized geometries of a) **4m-α,** b) **4m-β** and c) **6** and their corresponding highest occupied molecular orbital (HOMO) and lowest unoccupied molecular orbital (LUMO) distributions (ISO value = 0.02), together with the transition energies for the relevant lowest singlet and triplet states calculated at the PBE0/6-31G(d,p) level in vacuum by using TDA-DFT methodology. *f* is the oscillator strength.

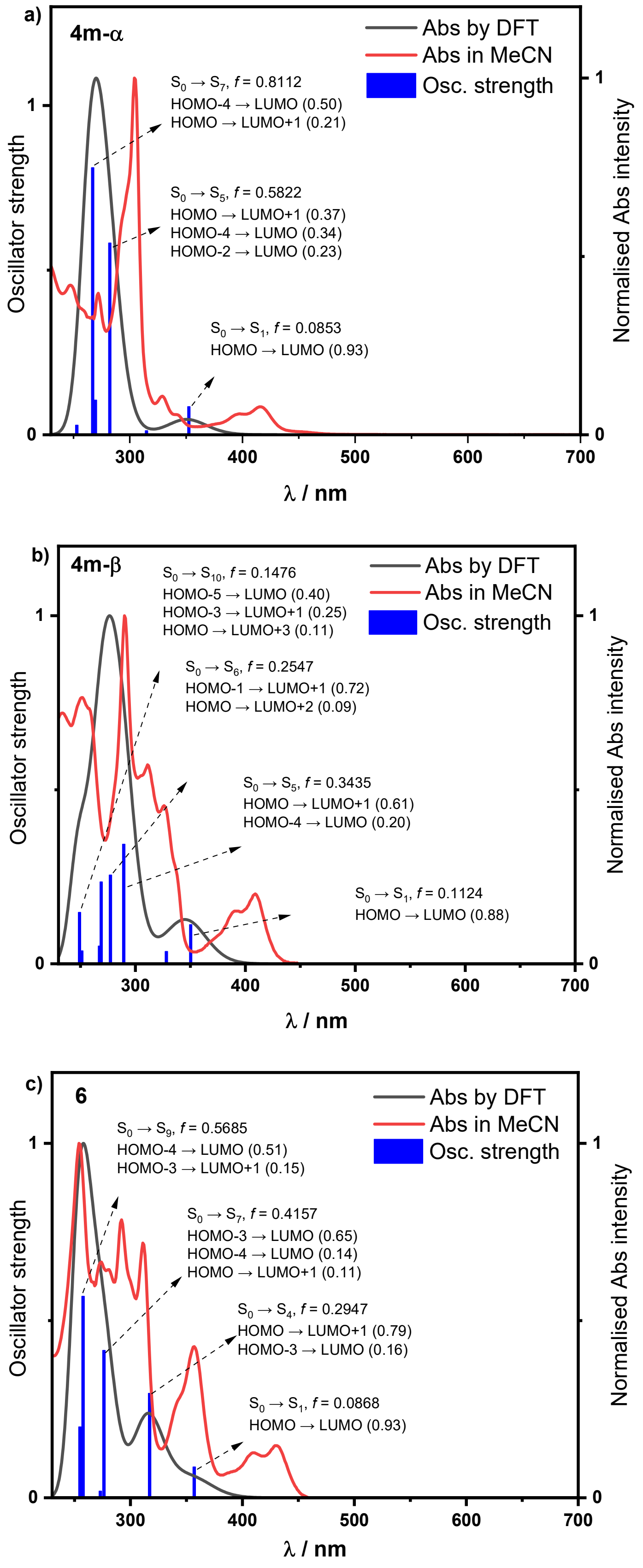

Figure S96. TDA-DFT simulation and experimental UV-Vis absorption in the gas phase of (a) **4m-α**, (b) **4m-β** and (c) **6**.

S5. Electrochemical Properties

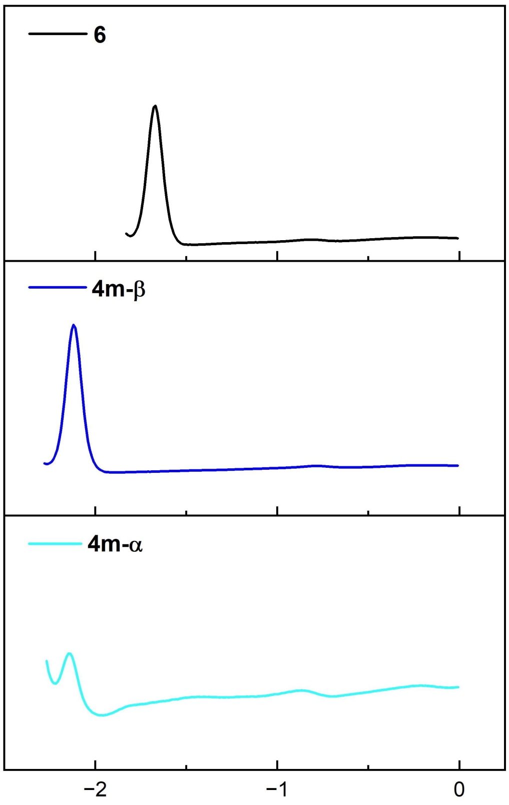

Figure S97. Differential pulse voltammetry (DPV) plots for the reduction processes for (a) **4m-α**, (b) **4m-β** and (c) **6**.

Table **S1**. Electrochemical data and HOMO-LUMO levels for **4m-α**, **4m-β** and **6**.

Compound	E_{red} / V[a]	LUMO / eV[b]
**4m-α**	-2.14	-2.28
**4m-β**	-2.12	-2.32
**6**	-1.67	-2.73

[a] E_{red} are cathodic peak potentials obtained from DPV using F_c/F_c^+ as the internal reference and referenced versus SCE (0.38V vs. SCE) in MeCN with 0.1 M [nBu$_4$N]PF$_6$ as the supporting electrolyte.[2] [b] $E_{LUMO} = -(E^{red} + 4.8)eV$,[3] where E^{red} is the cathodic peak potential calculated from DPV relative to F_c/F_c^+.

S6. Photophysical Properties

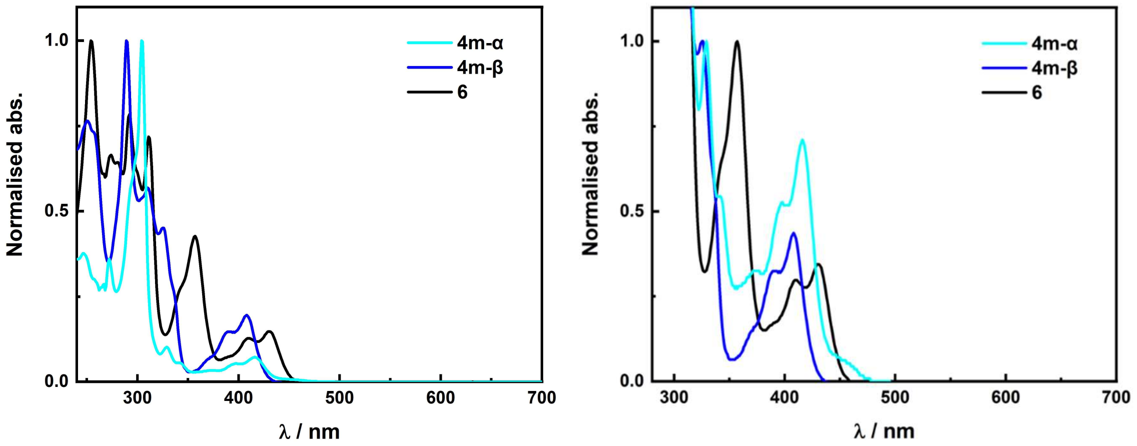

Figure **S98.** Normalised absorption plots of **4m-α**, **4m-β** and **6** measured in MeCN.

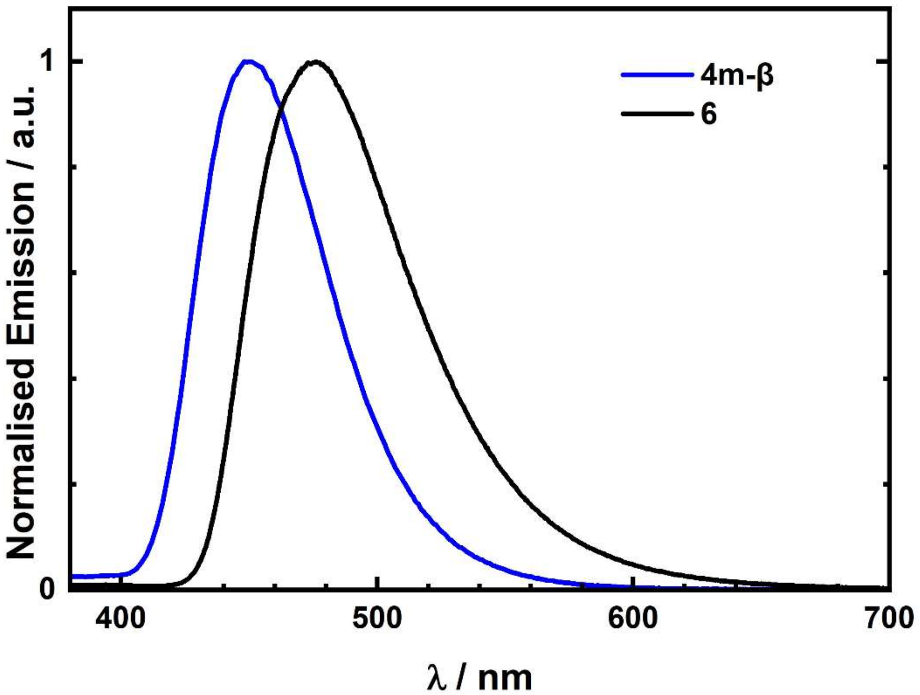

Figure **S99.** Normalised emission plot of **4m-β** (λ_{exc} = 330 nm) and **6** (λ_{exc} = 360 nm) measured in MeCN.

S7. References

(1) Connelly, N. G.; Geiger, W. E. Chemical Redox Agents for Organometallic Chemistry. *Chem. Rev.* **1996**, *96* (2), 877–910. https://doi.org/10.1021/cr940053x.

(2) Pavlishchuk, V. V; Addison, A. W. Conversion Constants for Redox Potentials Measured versus Different Reference Electrodes in Acetonitrile Solutions at 25°C. *Inorganica Chim. Acta* **2000**, *298* (1), 97–102. https://doi.org/https://doi.org/10.1016/S0020-1693(99)00407-7.

(3) Cardona, C. M.; Li, W.; Kaifer, A. E.; Stockdale, D.; Bazan, G. C. Electrochemical Considerations for Determining Absolute Frontier Orbital Energy Levels of Conjugated Polymers for Solar Cell Applications. *Adv. Mater.* **2011**, *23* (20), 2367–2371. https://doi.org/https://doi.org/10.1002/adma.201004554.

(4) Humphrey, W.; Dalke, A.; Schulten, K. VMD: Visual Molecular Dynamics. *J. Mol. Graph.* **1996**, *14* (1), 33–38. https://doi.org/https://doi.org/10.1016/0263-7855(96)00018-5.

(5) O'Boyle, N. M.; Hutchison, G. R. Cinfony – Combining Open Source Cheminformatics Toolkits behind a Common Interface. *Chem. Cent. J.* **2008**, *2* (1), 24. https://doi.org/10.1186/1752-153X-2-24.

(6) Hunter, J. D. Matplotlib: A 2D Graphics Environment. *Comput. Sci. Eng.* **2007**, *9* (3), 90–95. https://doi.org/10.1109/MCSE.2007.55.

(7) M. J. Frisch, G. W. Trucks, H. B. Schlegel, G. E. Scuseria, M. A. Robb, J. R. Cheeseman, G. Scalmani, V. Barone, G. A. Petersson, H. Nakatsuji, X. Li, M. Caricato, A. V. Marenich, J. Bloino, B. G. Janesko, R. Gomperts, B. Mennucci, H. P. Hratchian, J. V. Ortiz, A. F. Izmaylov, J. L. Sonnenberg, Williams, F. Ding, F. Lipparini, F. Egidi, J. Goings, B. Peng, A. Petrone, T. Henderson, D. Ranasinghe, V. G. Zakrzewski, J. Gao, N. Rega, G. Zheng, W. Liang, M. Hada, M. Ehara, K. Toyota, R. Fukuda, J. Hasegawa, M. Ishida, T. Nakajima, Y. Honda, O. Kitao, H. Nakai, T. Vreven, K. Throssell, J. A. Montgomery Jr., J. E. Peralta, F. Ogliaro, M. J. Bearpark, J. J. Heyd, E. N. Brothers, K. N. Kudin, V. N. Staroverov, T. A. Keith, R. Kobayashi, J. Normand, K. Raghavachari, A. P. Rendell, J. C. Burant, S. S. Iyengar, J. Tomasi, M. Cossi, J. M. Millam, M. Klene, C. Adamo, R. Cammi, J. W. Ochterski, R. L. Martin, K. Morokuma, O. Farkas, J. B. Foresman, D. J. Fox, Wallingford, CT **2016**.

(8) Adamo, C.; Barone, V. Toward Reliable Density Functional Methods without Adjustable Parameters: The PBE0 Model. *J. Chem. Phys.* **1999**, *110* (13), 6158–6170. https://doi.org/10.1063/1.478522.

(9) Petersson, G. A.; Tensfeldt, T. G.; Montgomery Jr., J. A. A Complete Basis Set Model

Chemistry. III. The Complete Basis Set-quadratic Configuration Interaction Family of Methods. *J. Chem. Phys.* **1991**, *94* (9), 6091–6101. https://doi.org/10.1063/1.460448.

(10) Grimme, S. Density Functional Calculations with Configuration Interaction for the Excited States of Molecules. *Chem. Phys. Lett.* **1996**, *259* (1), 128–137. https://doi.org/https://doi.org/10.1016/0009-2614(96)00722-1.

(11) Hirata, S.; Head-Gordon, M. Time-Dependent Density Functional Theory within the Tamm–Dancoff Approximation. *Chem. Phys. Lett.* **1999**, *314* (3), 291–299. https://doi.org/https://doi.org/10.1016/S0009-2614(99)01149-5.

(12) Yu, H.; Gao, B.; Hu, B.; Huang, H. Charge-Transfer Complex Promoted C–N Bond Activation for Ni-Catalyzed Carbonylation. *Org. Lett.* **2017**, *19* (13), 3520–3523. https://doi.org/10.1021/acs.orglett.7b01488.

(13) Pandey, G.; Koley, S.; Talukdar, R.; Sahani, P. K. Cross-Dehydrogenating Coupling of Aldehydes with Amines/R-OTBS Ethers by Visible-Light Photoredox Catalysis: Synthesis of Amides, Esters, and Ureas. *Org. Lett.* **2018**, *20* (18), 5861–5865. https://doi.org/10.1021/acs.orglett.8b02537.

(14) Feltenberger, J. B.; Hayashi, R.; Tang, Y.; Babiash, E. S. C.; Hsung, R. P. Enamide-Benzyne-[2 + 2] Cycloaddition: Stereoselective Tandem [2 + 2]−Pericyclic Ring-Opening−Intramolecular N-Tethered [4 + 2] Cycloadditions. *Org. Lett.* **2009**, *11* (16), 3666–3669. https://doi.org/10.1021/ol901434g.
